# Facile Access to Chelating CAArC‐Phosphine (CAArCPhos) Palladium Complexes

**DOI:** 10.1002/anie.202504316

**Published:** 2025-10-15

**Authors:** K. Georg Leistikow, Alexander Wingelstern, Philipp Rohrmann, Jonas F. Wunsch, Jan Werst, Margit Brückner, Frank Rominger, Matthias Rudolph, A. Stephen K. Hashmi

**Affiliations:** ^1^ Organisch‐Chemisches Institut Universität Heidelberg Im Neuenheimer Feld 270 69120 Heidelberg Germany

**Keywords:** CAArCs, C–H activation, Chelating carbene ligands, Directing groups, Palladium

## Abstract

In this article, we present a synthetic route to 4‐(diphenyl‐phosphino) isoindolium salts based on a new protecting group strategy. Key to success is the use of hemi‐aminal methyl ether precursors, which serve as base‐stable iminium salt equivalents that enable halogen‐metal exchange and subsequent functionalization. After removal of the protecting group, the catalytically active palladium(II) complexes are formed of phosphine isoindolium salts by a CMD‐like metalation process, producing the first examples of chelating cyclic amino(aryl) carbene complexes. Calculations on the mechanism support an acetate‐assisted intramolecular palladation of the cyclic amino(aryl) carbene‐phosphine (CAArCPhos) ligand enabled by the phosphine directing group through a six‐membered transition state.

## Graphical abstract

A protection group strategy using methoxyisoindolines as protected iminium equivalents was developed to allow late‐stage functionalization of 4‐bromo isoindoliumsalts, enabling the synthesis of 4‐(diphenylphosphino) isoindolium salts. The phosphine functionality acts as a directing group for the palladation of the cyclic amino(aryl) carbene‐phosphine (CAArCPhos) ligand by an acetate‐assisted concerted‐metalated deprotonation (CMD)‐like process.

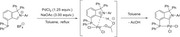

## Introduction

In recent years, ambiphilic singlet carbene ligands such as cyclic alkyl amino carbenes (CAACs) introduced by Bertrand,^[^ ^1^ ^]^ emerged as versatile ligands for applications in transition metal catalysis^[^ ^2^, ^3^, ^4^, ^5^, ^6^, ^7^, ^8^, ^9^, ^10^, ^11^, ^12^, ^13^, ^14^, ^15^, ^16^, ^17^, ^18^, ^19^, ^20^, ^21^, ^22^, ^23^, ^24^, ^25^, ^26^, ^27^, ^28^, ^29^ ^]^ main‐group element stabilization^[^ ^30^, ^31^, ^32^, ^33^, ^34^ ^]^ small‐molecule activation,^[^ ^35^, ^36^, ^37^, ^38^, ^39^, ^40^, ^41^, ^42^ ^]^ and lately as candidates for medicinal applications.^[^ ^43^ ^]^ In contrast, π‐extended cyclic (amino)aryl carbene (CAArC) ligands,^[^ ^44^, ^45^ ^]^ which possess an even more ambiphilic character, have received less attention, which can be attributed to the challenging metalation of amino(aryl) carbenes.^[^ ^45^, ^46^, ^47^, ^48^, ^49^, ^50^, ^51^, ^52^, ^53^, ^54^, ^55^, ^56^ ^]^ Nevertheless, CAArC ligands have already demonstrated their potential for catalysis^[^ ^44^ ^]^ and their potency as π‐chromophore ligand with potential applications in optoelectronics^[^ ^46^ ^]^ or lately even in photocatalysis.^[^ ^53^ ^]^

The introduction of coordinating groups into ambiphilic carbene ligand scaffolds allows to tune the character of carbene‐metal complexes as initial findings in catalysis^[^ ^57^ ^]^ and organometallic chemistry^[^ ^58^, ^59^, ^60^ ^]^ revealed for Lewis base‐functionalized CAACs (FunCAACs).^[^ ^61^ ^]^ However, the synthesis of Lewis base‐functionalized iminium salts is quite challenging. To begin with, the frequently used imine carbocation cyclization pathway^[^ ^1^, ^62^, ^63^ ^]^ towards CAAC/CAArC iminium salts suffers from the use of strong electrophiles, which prohibits the installation of many nucleophilic groups prior to the iminium cyclization. Another intrinsic problem is that due to a competing addition reaction^[^ ^47^ ^]^ or the direct formation of the reactive carbene accompanied by undesired side‐reactions^[^ ^47^, ^53^, ^56^, ^64^ ^]^ a late‐stage integration of nucleophilic groups into iminium salts is only tolerated under weakly basic conditions.^[^ ^61^ ^]^

Inspired by directed C–H activation chemistry, we considered to exploit the *peri*‐position of isoindolium salts as a rigid anchor for coordinating groups such as phosphines to facilitate the complexation of CAArCs. To protect the reactive iminium position in 4‐bromoisoindolium salts, 3‐methoxyisoindolines were regarded as ideal tool, which already demonstrated their synthetic potential as intermediates for counter anion exchange of isoindolium salts.^[^ ^47^ ^]^ Indeed, the base‐stable methoxy protecting group allowed hemi‐aminal methyl ethers to perform selective halogen‐metal‐exchange by reaction with *n*‐BuLi at low temperature. Trapping of the lithiated arene with diphenylphosphine chloride and subsequent deprotection under acidic conditions delivered phosphine isoindolium salts as first examples of chelating CAArC precursors.

## Results and Discussion

Our study started with the objective of synthesizing 4‐bromo‐isoindolium salts as platform for Lewis base‐functionalized isoindolium salts. Starting from 2,6‐dibromobenzaldehydes, several dibrominated imines **1a–j** could be prepared in yields ranging from 84% to 99%. Selective mono‐lithiation of imines **1** was enabled by the reaction with *n*‐BuLi in ether at −78 °C. In analogy to Bertrand's protocol,^[^ ^44^ ^]^ the lithiated species was reacted in situ with benzophenone and triflic anhydride to selectively afford 4‐bromo‐isoindolium salts

**Scheme 1:**
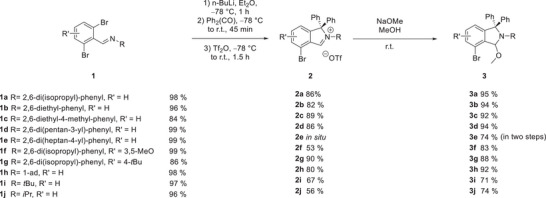
Synthesis of 4‐bromo 3‐methoxyisoindolines as protected iminium equivalents.

**2a–j** in moderate to high yields from 63% to 89%. The reaction was successful for *N‐*alkyl and *N‐*aryl‐substituted imines and even sterically demanding imines could be transformed into the corresponding iminium salt (see Scheme 1).^[^ ^47^ ^]^

The direct lithiation and functionalization of 4‐bromo‐isoindolium salts **2** was not effective and the iminium salts only decomposed under the applied conditions. Therefore, we introduced the methoxy protecting group by the reaction of isoindolium salts **2** with commercially available sodium methoxide solution in methanol in close analogy to Lorkowski et al.^[^ ^47^ ^]^ 4‐Bromo‐3‐methoxyisoindolines **3** were obtained as colorless to off‐white solids in high yields, which precipitated from methanol and could be purified by filtration (see Scheme 1). The masked isoindolium salts allowed us to functionalize the *peri‐*position by way of lithiation in the next step.

**Scheme 2:**
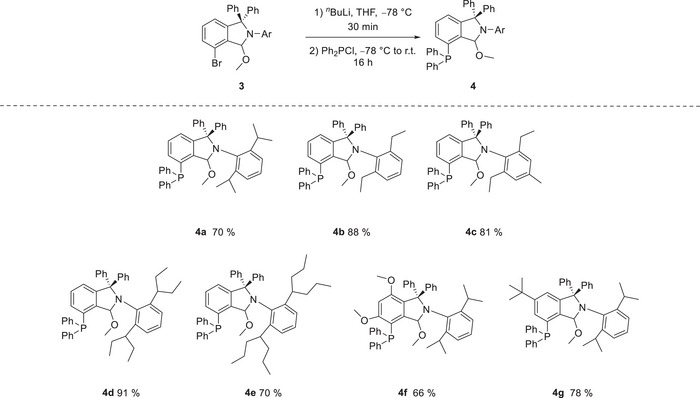
Halogen‐metal exchange and phosphinylation of *N*‐Aryl methoxyisoindolines.

In THF, halogen‐metal exchange of *N*‐aryl methoxyisoindolines **3** proceeded smoothly at −78 °C with *n*‐BuLi and subsequent trapping with diphenyl phosphine chloride afforded the corresponding phosphine‐substituted methoxyisoindolines **4a‐** **g** (see Scheme 2). After simple washing with diethyl ether, the hydrolysis‐sensitive products were obtained as colorless powders in good to high yields up to 91%. Single crystals of **4a**‐**4f** suitable for X‐ray diffractometry (see Supporting Information) gave proof for the desired products, which confirmed the stability of the protecting group.^[^ ^65^ ^]^

**Scheme 3:**
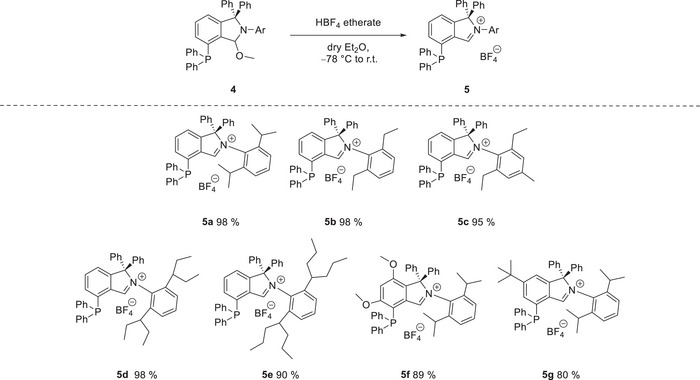
Synthesis of phosphine isoindolium salts by stoichiometric reaction of methoxyisoindolines with HBF_4_ etherate.

In contrast, *N*‐alkyl methoxyisoindolines **3** showed no selective reaction outcomes. We attribute this to the reduced stability of the protecting group in these species which aligns with their pronounced susceptibility towards hydrolysis in solution. As the final step in the synthesis of carbene‐precursors **5**, the methoxyisoindoline phosphines **4** were deprotected by stoichiometric reaction with HBF_4_ etherate, which required only short reaction times. The phosphine isoindolium salts **5** which precipitated from diethyl ether were obtained as orange to red solids in high yields up to 98% (see Scheme 3), which showed a characteristic doublet in the ^13^C NMR spectrum at 172.0–174.0 ppm with carbon‐phosphorus couplings of *^3^J_C‐P_* = 7.7–13.0 Hz. Crystals from **5a** suitable for single crystal X‐ray structure analysis were obtained from benzene, which confirmed the nature of the product and the reversibility of the iminium's protection/deprotection process (see Supporting Informaton for crystal structure).^[^ ^65^ ^]^

**Scheme 4:**
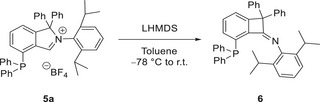
Ring contraction rearrangement of the free cyclic amino(aryl) carbene‐phosphine (CAArCPhos).

**Figure 1:**
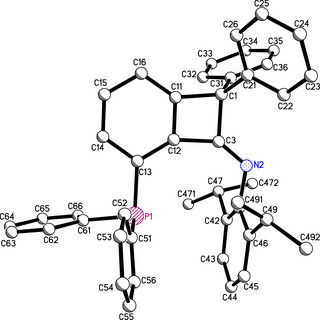
Selected bond distances [Å] and bond angles [°]: P1‐C13 1.835(3), C3‐C12 1.503(5), C1‐C11 1.536(5), C1‐C3 1.569(5), N2‐C3 1.260(4), C11‐C1‐C3 83.6(2), C12‐C3‐C1 90.0(3), C11‐C12‐C3 90.9(3).

In an endeavor to isolate the free carbene, phosphine isoindolium salt **5a** was reacted with lithium bis(trimethylsilyl)amide in toluene at low temperature. But, instead of the free carbene, the reaction in toluene afforded the ring‐contracted benzocyclobutenone‐imine phosphine **6** as revealed by X‐ray structure investigation (Figure 1, Scheme 4).^[^ ^65^ ^]^ The product could be isolated in a yield of 35%. A related ring‐contracting reaction was recently reported as a decomposition pathway of free, unsubstituted CAArCs.^[^ ^45^ ^]^

The reaction of phosphine isoindolium salts **5** with metal precursors and strong bases such as alkali bis(trimethylsilyl)amides was unselective and resulted in complex reaction mixtures. Recently, Lorkowski et al. reported the efficient use of sodium acetate as weak base for the metalation of CAACs^[^ ^66^ ^]^ and CAArCs^[^ ^45^ ^]^ by the “*sans carbène*” (without carbene) pathway. We figured if the metalation of the CAArC‐phosphine could be enabled by this alternative pathway in combination with a suitable metal precursor.

**Scheme 5:**
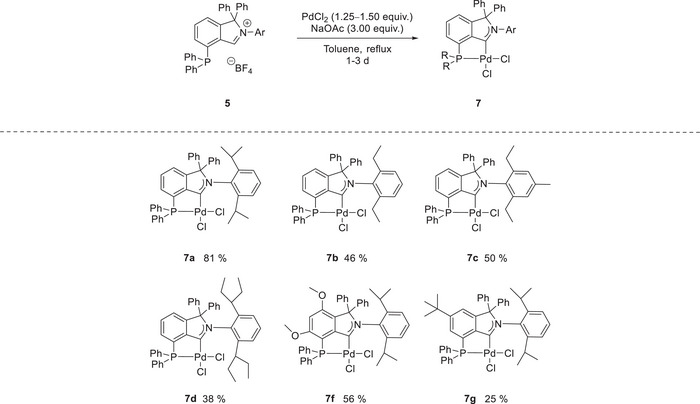
Directing group‐enabled palladation of phosphine‐isoindolium salts with sodium acetate.

Indeed, refluxing phosphine isoindolium salts **5** with palladium(II) chloride and sodium acetate in toluene afforded air‐stable square‐planar CAArCPhos palladium chloride complexes **7** in yields up to 81% (see Scheme 5). The palladium carbene complexes **7** show the carbene's characteristic downfield‐shifted doublet at *δ* = 203.5–212.2 ppm with a coupling constant of *^3^J_C‐P_ *= 5.0–5.3 Hz as observed by ^13^C NMR spectroscopy.

**Figure 2:**
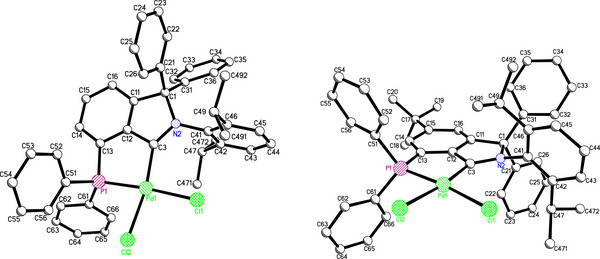
X‐ray structure of carbene‐palladium complexes **7a** and **7g**. Selected bond distances[Å] and bond angles[°] for **7a**: C3‐Pd1 2.003(4), Pd1‐P1 2.2383(11), Pd1‐Cl1 2.3402(12), Pd1‐Cl2 2.3535(11); N2‐C3‐C12 106.3(4), C3‐Pd1‐P1 86.61(12), C3‐Pd1‐Cl1 98.15(12), C3‐Pd1‐Cl2 169.82(12), P1‐Pd1‐Cl2 83.29(4), C13‐P1‐Pd1 102.78(14). Selected bond distances[Å] and bond angles[°] for **7g**: C3‐Pd1 1.994(6), Pd1‐P1 2.2434(16), Pd1‐Cl1 2.3511(16), Pd1‐Cl2 2.3515(16); N2‐C3‐C12 106.3(5), C3‐Pd1‐P1 85.79(17), C3‐Pd1‐Cl1 97.89(16), C3‐Pd1‐Cl2 170.05(17), P1‐Pd1‐Cl2 84.35(6), C13‐P1‐Pd1 102.9(2).

Single‐crystals of palladium‐CAArCPhos complex **7a** and **7g** could be obtained. The expected connectivity could be confirmed by single crystal X‐ray structure analysis, the latter revealing a significant geometric strain around the palladium center (Figure 2), which can be attributed to the *cis‐*coordination mode of the CAArCPhos‐ligand.^[^ ^65^ ^]^

The length of the carbene‐palladium bond of 2.003(4) Å is comparable to the bond length of *trans*‐^Me,Me^CAArC‐PdCl_2_‐triphenylphosphine complex, which was obtained by oxidative addition of tetrakis(triphenylphosphine)‐Pd(0) into a chloroisoindolium salt reported by Bertrand.^[^ ^62^ ^]^ The use of sodium or cesium pivalate or sodium trifluoroacetate bases did not provide any carbene product. Using our metalation conditions, the reaction of phosphine isoindolium salt **5a** with platinum(II) chloride to the platinum carbene **8a** was also successful, which was confirmed by X‐ray structure analysis of a single‐crystal obtained from a crude reaction mixture that could not be purified enough to afford an analytically pure sample (for X‐ray structure of **8a**, see Supporting Information).^[^ ^65^ ^]^

**Scheme 6:**
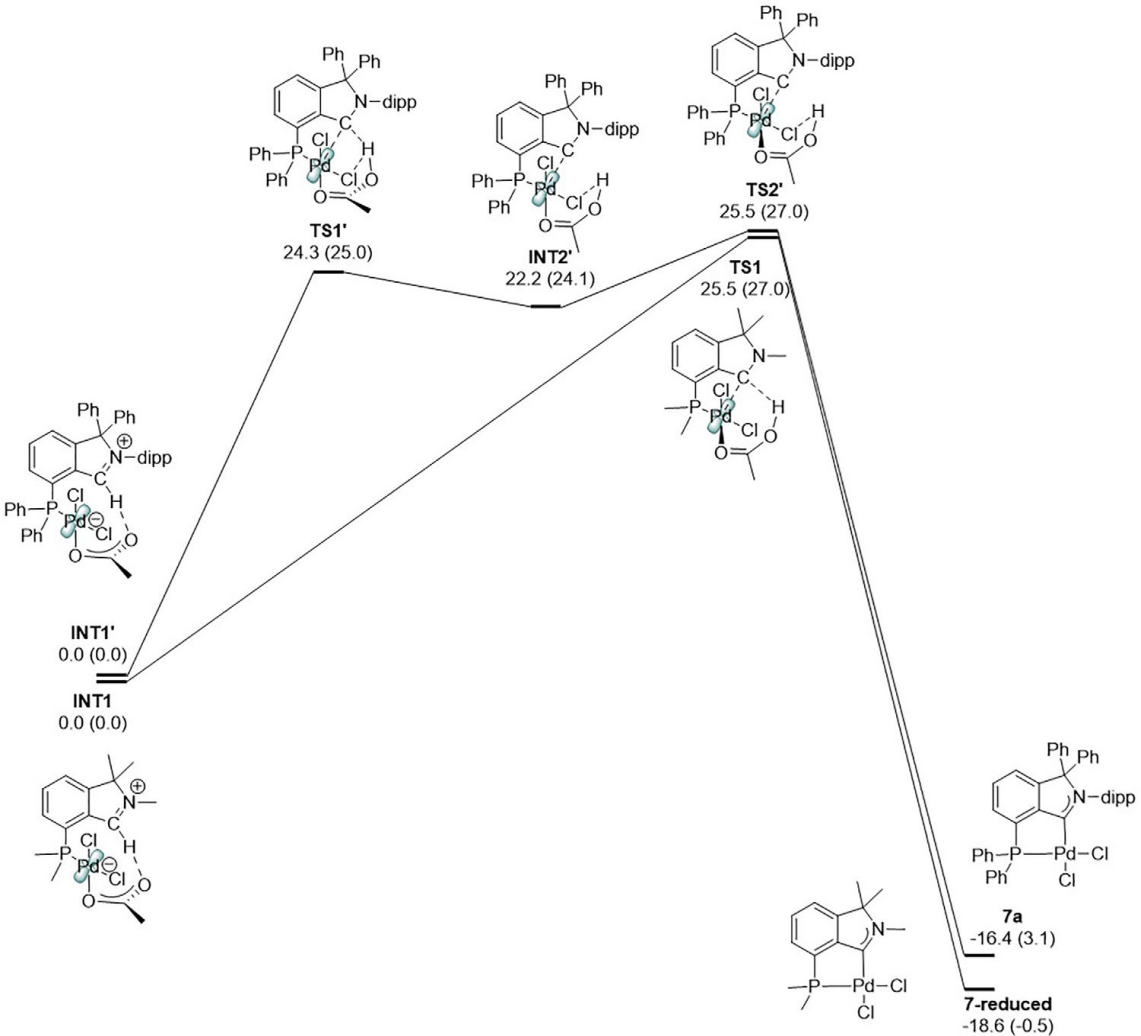
Calculated (wB97M‐D4, def2‐TZVP) reaction mechanism for the carbene formation and metalation of **5a** and a sterically reduced model. See Supporting Information for computational details and a discussion of bond lengths on the reaction pathway. Gibbs energies are given in kcal mol^−1^. Numbers in parentheses are electronic energies.

In terms of the palladation, the role of the phosphine directing group was quintessential to facilitate the process since no palladation reaction was observed for unsubstituted isoindolium salts under the same reaction conditions. Mechanistically, we believe that the reaction proceeds by an acetate‐assisted intramolecular palladation mechanism without intermediacy of the free carbene (in analogy to Lorkowski et al.^[^ ^45^ ^]^). This was supported by our DFT‐calculations (wB97M‐D4, def2‐TZVP, Scheme 6). We believe that the rationale behind the directing nature of the phosphine is the formation of a reactive iminium‐palladate complex, which is formed from the phosphine, palladium chloride, and one acetate ligand. By an intramolecular reaction, the iminium proton interacts with the basic acetate ligand on the palladium center (Scheme 6), which reduces the entropy of activation for the palladation.

**Scheme 7:**
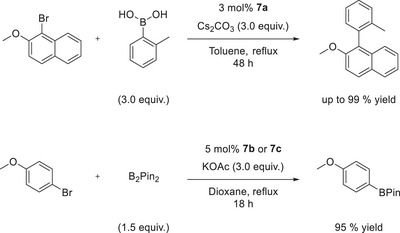
Catalytic activity of chelating CAArCPhos)PdCl_2_ complexes **7** as catalysts in classical cross‐coupling reactions.

In a sterically reduced model, we found a single transition state **TS1** for the CAArCPhos palladation with a reasonable Gibbs energy of 25.5 kcal/mol indicating a concerted metalation‐deprotonation (CMD) process which confirmed our rationale. The transfer of the carbene to the metal is accompanied by elimination of acetic acid.

Finally, we performed calculations with our model substance **5a** bearing all substituents which revealed that due to steric congestion the process is not a concerted process anymore. In that case, we found a flat energy region around intermediate **INT2’** flanked by the two transition states **TS1’** and **TS2’**. Although this is not a concerted mechanism, it is still intramolecular and has an activation Gibbs energy of 24.3 kcal mol^−1^, which confirmed favored carbene formation for fully substituted **5a** under the given conditions (for detailed discussions, see Supporting Information).

As part of our initial investigation, we tested the performance of palladium(II) CAArCPhos complexes **7** as catalysts in cross‐coupling reactions (see Scheme 7). Using 3 mol % of catalyst **7a**, the Suzuki–Miyaura reaction of 1‐bromo‐2‐methoxynaphthalene and *ortho*‐tolyl boronic acid afforded the tri‐*ortho* substituted biaryl in high yields up to 99%. The synthesis of tetra‐*ortho* substituted biaryls using 2,6‐dimethylphenyl boronic acid could not be achieved. Additionally, catalysts **7b** and **7c** showed activity in the Miyaura borylation of 4‐bromoanisole and bis(pinacolato)diboron.

## Conclusion

In conclusion, we have introduced a protecting group strategy for the synthesis of *peri*‐functionalized phosphine‐isoindolium salts using intermediate hemi‐aminal methyl ethers as base‐stable iminium equivalents. The CAArC‐phosphine (CAArCPhos) ligand precursors were metalated using palladium chloride and sodium acetate in refluxing toluene while for unsubstituted isoindolium salts no palladation could be observed. The strained palladium‐CAArCPhos complexes, which represent the first examples of chelating CAArC‐metal complexes showed catalytic activity in palladium‐catalyzed cross‐coupling reactions. The directing effect of the phosphine was confirmed by DFT‐calculations, which indicated an acetate‐assisted intramolecular palladation mechanism by the “*sans carbène*” pathway.^[^ ^45^ ^]^ We assume that the introduction of coordinating groups into the CAArC framework will help to facilitate the carbene metalation in future investigations. Our protecting group strategy should allow to expand the field of amino(aryl) carbene complexes to valuable chelate motives with accompanying new fields of applications.

## Supporting Information

The authors have cited additional references within the Supporting Information.^[^ ^67^, ^68^, ^69^, ^70^, ^71^, ^72^, ^73^, ^74^, ^75^, ^76^, ^77^, ^78^, ^79^, ^80^, ^81^, ^82^, ^83^ ^]^

## Conflict of Interests

The authors declare no conflict of interest.

## Supporting information



Supporting Information

## Data Availability

The data that support the findings of this study are available in the supplementary material of this article.
