# Supplementary material for: Facile Access to Chelating CAArC‐Phosphine (CAArCPhos) Palladium Complexes
Source: Angew Chem Int Ed Engl. 2025 Oct 15;64(48):e202504316. doi: 10.1002/anie.202504316 (PMC12643342; doi:10.1002/anie.202504316)
Supplement: Supplementary file 1 — Supporting Information [file ANIE-64-e202504316-s001.docx]

**Supporting Information to**

**“Facile access to chelating CAArC-phosphine (CAArCPhos) palladium complexes”**

# **General remarks**

**Chemical compunds** were purchased by industrial suppliers (abcr, Acros, Alfa Aeser, BLDPharm, carbolution, Merck, Sigma-Aldrich, thermofisher, TCI) and used without further purification unless stated otherwise. 2,6-Dibromobenzaldehyde was purchased from BLDPharm. The compounds 2,6-dibromo-3,5-dimethoxybenzaldehyde^[15]^ and 4-*tert*-butyl-2,6-dibromobenzaldehyde^[16]^ were synthesized according to known procedures. All experimental manipulations were performed under Argon atmosphere by using common Schlenk or glovebox techniques (unless specified otherwise). Anhydrous and oxygen-free solvents (acetonitrile, DCM, diethylether, methanol, THF, toluene and pentane) were dried by passing through Al_2_O_3_ columns employing a solvent purification system (*MB* *SPS-800-Benchtop* from *MBraun*). Chemical reactions were drawn with *ChemDraw Professional 22.2.0* by *PerkinElmer* and structure names were determined by the program.

**NMR measurements** were recorded by a Bruker Avance III 300, Bruker Avance DRX 300, Bruker Avance III 400, Bruker ​Avance III 500 or Bruker Avance III 600. ^1^H NMR and ^13^C NMR spectra were referenced to the residual proton or carbon signals of the deuterated solvent.^[17]^ All ^13^C NMR spectra were recorded as proton-decoupled spectra. The analysis of proton and carbon NMR signals was supported by two-dimensional NMR techniques (COSY, HSQC, HMBC), ^13^C-dept NMR spectra as well as phosphorus-decoupled ^13^C NMR spectra (if required). Chemical shift *δ* is given in ppm, relative to Si(CH_3_)_4_ (for ^1^H and ^13^C NMR spectroscopy), H_3_PO_4_ (for ^31^P spectroscopy). Multiplets in NMR spectra were abbreviated as follows: d for doublet, dd for doublet of doublet, t for triplet, dt for doublet of triplet, m for multiplet or sept for septet. As implied by ^13^C-dept NMR analysis, carbon signals were specified as Cq (quaternary), CH (tertiary), CH_2_ (secondary) or CH_3_ (primary). NMR-spectroscopic measurements were processed by *MestReNova 14.2.1* by *Mestrelab Research* and *TopSpin 3.7.0* by *Bruker*. The spectra were edited by *MestReNova 14.2.1* by *Mestrelab Research*.

**Mass spectrometry (MS) and high-resolution mass spectrometry (HR/MS) spectra** were recorded at the mass spectrometry facility of the department of organic chemistry under supervision of Dr. J. Gross on the following spectrometers: JEOL AccuTOF GCx (EI), Bruker ApexQe hybrid 9.4 T FT-ICR (ESI, MALDI, DART), Finnigan LCQ (ESI), Bruker Autoflex Speed MALDI-TOF (MALDI) and Bruker timsTOFfleX (ESI, MALDI).

**X-ray crystallographic measurements** were performed by the chemistry department under the supervision of Dr. F. Rominger. For crystallographic analysis, the instruments *Bruker APEX II Quazar* containing a Mo-micro source or *Stoe Stadivari* with Cu-micro source and *Pilatus* detector were used. X-ray crystallographic measurements were edited with *Mercury 4.2.0* by *CCDC*. Space-group and crystal structure determination was supported by *SHELXT*.^[18]^ Crystal structure refinement was performed by *SHELXL-2019/2.*^[19]^ The programs *APEX*, *APEX2*, *SMART*, *SAINT*, *SAINT-Plus* by *Bruker* were used.^[20]^

**IR spectroscopic measurements** were collected using a *Bruker Lumos* instrument with a *Germanium ATR-Crystal*.

**Melting points** (m.p.) were measured in open glass capillaries on a Stuart SMP10 melting point apparatus.

## **Synthetic procedures**

**GP 1. Synthesis of *N*-aryl 2,6-dibromophenyl imines (1a)**–**(1e)**

In 250 mL dry toluene, 2,6-dibromobenzaldehyde (25.8 g, 97.8 mmol, 1.00 equiv.) and the appropriate aniline (97.8 mmol, 1.00 equiv.) were dissolved and molecular sieves (4Å) were added. The reaction mixture was stirred for 2 d at 100 °C. The suspension was filtered over a MgSO_4_ plug to remove residual molecular sieves and the product was eluted with DCM or toluene. The solvent was removed *in vacuo* to obtain the product as pure compound.

*Note*: Imine synthesis of (**1d**) and (**1e**) was performed on a smaller scale due to economic reasons.

**GP 2. Synthesis of *N*-alkyl 2,6-dibromophenyl imines (1h)**–**(1j)**

Imine synthesis was performed according to the general procedure by Morales et al.^[21]^ 2,6-dibromobenzaldehyde (3.96 g, 15.0 mmol, 1.00 equiv.) was dissolved in 30 mL dry DCM with molecular sieves (4Å). Pyrrolidine catalyst (0.12 mL, 107 mg, 1.50 mmol, 0.10 equiv.) and the corresponding amine (15.0 mmol, 1.00 equiv.) were added and the reaction mixture was stirred under reflux for 2 d. The suspension was filtrated over a magnesium sulfate plug and the product was eluted with DCM. The solvent of the filtrate was concentrated *in vacuo* to yield the imines.

**1-(2,6-dibromophenyl)-*N*-(2,6-diisopropylphenyl)methanimine (1a)**

According to **GP 1**. The product was eluted with DCM and the solvent was removed *in vacuo* to obtain the product as a yellow viscous oil. Yield: 98 % (40.4 g, 95.5 mmol).

**^1^H NMR** (500.22 MHz, CDCl_3_, 300 K): *δ* [ppm] = 8.38 (s, 1H), 7.68 (d, *J* = 8.0 Hz, 2H), 7.23–7.11 (m, 4H), 3.26 (sept, *J* = 6.9 Hz, 2H), 1.21 (d, *J* = 6.9 Hz, 12H).

**13C{1H} NMR** (125.78 MHz, CDCl_3_, 300 K): *δ*[ppm] = 161.6 (s, CH, 1C), 148.5 (s, Cq, 1C), 137.8 (s, Cq, 2C), 134.6 (s, Cq, 1C), 133.4 (s, CH, 2C), 131.6 (s, CH, 1C), 124.8 (s, CH, 1C), 124.2 (s, Cq, 2C), 123.3 (s, CH, 2C), 27.8 (s, CH, 2C), 24.2 (s, CH_3_, 4C).

**IR (ATR):** ν[cm^-1^] = 3063, 2961, 2926, 2867, 1634, 1573, 1549, 1459, 1430, 1382, 1362, 1324, 1255, 1194, 1171, 1099, 1041, 930, 881, 797, 775, 756, 726, 716.

**Mass spectrometry (MS-EI, +)**: *calcd.* C_19_H_21_Br_2_N: 421.0035, *found:* 421.0038.

**1-(2,6-dibromophenyl)-*N*-(2,6-diethylphenyl)methanimine (1b)**

According to **GP 1**. The product was eluted with toluene and the solvent was removed *in vacuo* to obtain the product as a yellow solid. Yield: 96 % (37.2 g, 94.0 mmol).

**^1^H NMR** (400.33 MHz, CDCl_3_, 300 K): *δ* [ppm] = 8.42 (s, 1H), 7.67 (d, *J* = 8.0 Hz, 2H), 7.16–7.05 (m, 4H), 2.67 (q, *J* = 7.5 Hz, 4H), 1.20 (t, *J* = 7.5 Hz, 6H).

**13C{1H} NMR** (100.66 MHz, CDCl_3_, 300 K): *δ*[ppm] = 161.8 (s, CH, 1C), 149.9 (s, Cq, 1C), 134.7 (s, Cq, 1C), 133.4 (s, CH, 2C), 133.1 (s, Cq, 2C), 131.6 (s, CH, 1C), 126.4 (s, CH, 2C), 124.6 (s, CH, 1C), 124.2 (s, Cq, 2C), 24.9 (s, CH_2_, 2C), 15.3 (s, CH_3_, 2C).

**IR (ATR):** ν[cm^-1^] = 3074, 2969, 2940, 2862, 1928, 1858, 1630, 1576, 1552, 1469, 1449, 1424, 1367, 1343, 1189, 1172, 1105, 1059, 984, 904, 886, 866, 823, 806, 768, 751, 731, 714, 691.

**Mass spectrometry (MS-EI, +)**: *calcd.* C_17_H_17_Br_2_N: 392.9722, *found:* 392.9709.

**m.p.** [°C]: 75.

**1-(2,6-dibromophenyl)-*N*-(2,6-diethyl-4-methylphenyl)methanimine (1c)**

According to **GP 1**. The product was eluted with DCM and the solvent was removed *in vacuo* to obtain the title compound as a yellow solid. Yield: 84 % (34.5 g, 84.3 mmol).

**^1^H NMR** (400.33 MHz, CDCl_3_, 300 K): *δ* [ppm] = 8.40 (s, 1H), 7.67 (d, *J* = 8.1 Hz, 2H), 7.13 (t, *J* = 8.1 Hz, 1H), 6.95 (s, 2H), 2.65 (q, *J* = 7.5 Hz, 4H), 2.35 (s, 3H), 1.19 (t, *J* = 7.5 Hz, 6H).

**13C{1H} NMR** (100.66 MHz, CDCl_3_, 300 K): *δ*[ppm] = 161.8 (s, CH, 1C), 147.5 (s, Cq, 1C), 134.9 (s, Cq, 1C), 133.9 (s, Cq, 1C), 133.3 (s, CH, 2C), 133.2 (s, Cq, 2C), 131.5 (s, CH, 1C), 127.2 (s, CH, 2C), 124.1 (s, Cq, 2C), 24.9 (s, CH_2_, 2C), 21.1 (s, CH_3_, 1C), 15.5 (s, CH_3_, 2C).

**IR (ATR):** ν[cm^-1^] = 2959, 2928, 2872, 1870, 1731, 1633, 1575, 1551, 1460, 1421, 1369, 1335, 1264, 1198, 1185, 1137, 1064, 979, 886, 852, 770, 719, 641.

**Mass spectrometry (MS-EI, +)**: *calcd.* C_18_H_19_Br_2_N: 406.9879, *found:* 406.9892.

**m.p.** [°C]: 95.

**1-(2,6-dibromophenyl)-*N*-(2,6-diethylphenyl)methanimine (1d)**

According to **GP 1**. In 75 mL dry toluene, 2,6-dibromobenzaldehyde (5.42 g, 20.6 mmol, 1.00 equiv.) and 2,6-di(pentan-3-yl)aniline (5.05 g, 20.6 mmol, 1.00 equiv.) were dissolved and molecular sieves (4Å) were added. The reaction mixture was stirred for 2 d at 100 °C. The suspension was filtered over a MgSO_4_ plug and the product was eluted with DCM. The solvent was removed *in vacuo* and the product was obtained as yellow oil. Yellow needles that spontaneously crystallized from the oil could be analyzed by X-Ray diffractometry. Yield: 99 % (9.75 g, 20.4 mmol).

**^1^H NMR** (300.51 MHz, CD_2_Cl_2_, 300 K): *δ* [ppm] = 8.35 (s, 1H), 7.70 (d, *J* = 8.0 Hz, 2H), 7.19–7.05 (m, 4H), 2.90–2.77 (m, 2H), 1.73–1.46 (m, 8H), 0.79 (t, *J* = 7.4 Hz, 12H).

**13C{1H} NMR** (100.66 MHz, CD_2_Cl_2_, 300 K): *δ*[ppm] = 161.5 (s, CH, 1C), 152.0 (s, Cq, 1C), 135.0 (s, Cq, 2C), 134.4 (s, Cq, 1C), 133.9 (s, CH, 2C), 131.9 (s, CH, 1C), 124.7 (s, Cq, 2C), 124.7 (s, CH, 1C), 124.2 (s, CH, 2C), 41.8 (s, CH, 2C), 29.2 (s, CH_2_, 4C), 12.2 (s, CH_3_, 4C).

**IR (ATR):** ν[cm^-1^] = 3059, 2960, 2930, 2871, 1923, 1860, 1792, 1641, 1573, 1548, 1461, 1444, 1429, 1377, 1344, 1259, 1213, 1193, 1170, 1145, 1102, 1039, 968, 879, 799, 773, 754, 725.

**Mass spectrometry (MS-EI, +)**: *calcd.* C_23_H_29_Br_2_N: 477.0661, *found:* 477.0652.

***N*-(2,6-di(heptan-4-yl)phenyl)-1-(2,6-dibromophenyl)methanimine (1e)**

In 50 mL dry toluene, 2,6-dibromobenzaldehyde (4.17 g, 15.8 mmol, 1.00 equiv.) and 2,6-di(heptan-4-yl)aniline (4.81 g, 15.8 mmol, 1.00 equiv.) were dissolved and molecular sieves (4Å) were added. The reaction mixture was stirred for 2 d at 100 °C. The suspension was filtered over a MgSO_4_ plug and the product was eluted with DCM. The solvent was removed *in vacuo* and the product was obtained as yellow-orange oil. Yield: 99 % (8.39 g, 15.7 mmol).

**^1^H NMR** (400.33 MHz, CDCl_3_, 300 K): *δ* [ppm] = 8.36 (s, 1H), 7.67 (d, *J* = 8.1 Hz, 2H), 7.14–7.05 (m, 4H), 2.97–2.89 (m, 2H), 1.60–1.46 (m, 8H), 1.30–1.10 (m, 8H), 0.80 (t, *J* = 7.3 Hz, 12H).

**13C{1H} NMR** (100.66 MHz, CDCl_3_, 300 K): *δ*[ppm] = 160.9 (s, CH, 1C), 151.2 (s, Cq, 1C), 135.3 (s, Cq, 2C), 133.7 (s, CH, 2C), 133.5 (s, Cq, 1C), 131.5 (s, CH, 1C), 124.8 (s, Cq, 2C), 124.3 (s, CH, 1C), 123.9 (s, CH, 2C), 39.3 (s, CH_2_, 4C), 38.3 (s, CH, 2C), 20.8 (s, CH_2_, 4C), 14.6 (s, CH_3_, 4C).

**IR (ATR):** ν[cm^-1^] = 3059, 2955, 2929, 2869, 1635, 1573, 1549, 1446, 1430, 1377, 1260, 1211, 1193, 1169, 1145, 1065, 959, 880, 797, 774, 757, 725.

**Mass spectrometry (MS-EI, +)**: *calcd.* C_27_H_37_Br_2_N: 533.1287, *found:* 533.1278.

**1-(2,6-dibromo-3,5-dimethoxyphenyl)-*N*-(2,6-diisopropylphenyl)methanimine (1f)**

In analogy to **GP1**. 2,6-dibromo-3,5-dimethoxybenzaldehyde (25.9 g, 80.0 mmol, 1.00 equiv.) and 2,6-diisopropylaniline (80.0 mmol, 1.00 equiv.) were dissolved in 200 mL dry toluene and molecular sieves (4Å) were added. The reaction was stirred for 2 d at 100 °C. The suspension was filtered over a MgSO_4_ plug to remove residual molecular sieves and the product was eluted with DCM. The solvent was removed *in vacuo* to obtain the product as yellow solid in quantitative yield (38.7 g, 80.0 mmol).

**^1^H NMR** (400.33 MHz, CD_2_Cl_2_, 300 K): *δ* [ppm] = 8.34 (s, 1H), 7.22–7.18 (m, 2H), 7.16–7.11 (m, 1H), 6.68 (s, 1H), 3.97 (s, 6H), 3.32 (sept, *J* = 6.8 Hz, 2H), 1.21 (d, *J* = 6.8 Hz, 12H).

**13C{1H} NMR** (100.66 MHz, CD_2_Cl_2_, 300 K): *δ*[ppm] = 162.3 (s, CH, 1C), 157.0 (s, Cq, 2C), 148.7 (s, Cq, 1C), 138.3 (s, Cq, 2C), 136.9 (s, Cq, 1C), 125.1 (s, CH, 1C), 123.5 (s, CH, 2C), 104.2 (s, Cq, 2C), 98.5 (s, CH, 1C), 57.3 (s, CH_3_, 2C), 28.0 (s, CH, 2C), 24.3 (s, CH_3_, 4C).

**IR (ATR):** ν[cm^-1^] = 2958, 2867, 1702, 1642, 1569, 1466, 1456, 1421, 1360, 1336, 1209, 1177, 1099, 1059, 955, 848, 810, 756, 739, 685.

**Mass spectrometry (MS-EI, +)**: *calcd.* C_21_H_25_Br_2_NO_2_: 481.0247, *found*: 481.0257.

**m.p.** [°C]: 88.

**1-(2,6-dibromo-4-((*tert*-butyl)phenyl)-*N*-(2,6-diisopropylphenyl)methanimine (1g)**

In analogy to **GP1**. 4-*tert*-butyl-2,6-dibromobenzaldehyde (25.6 g, 80.0 mmol, 1.00 equiv.) and 2,6-diisopropylaniline (80.0 mmol, 1.00 equiv.) were dissolved in 200 mL dry toluene and molecular sieves (4Å) were added. The reaction was stirred for 2 d at 100 °C. The suspension was filtered over a MgSO_4_ plug to remove residual molecular sieves and the product was eluted with DCM. The solvent was removed *in vacuo* to give the product as yellow solid in 86 % yield
(33.0 g, 68.8 mmol).

**^1^H NMR** (400.33 MHz, CD_2_Cl_2_, 300 K): *δ* [ppm] = 8.36 (s, 1H), 7.71 (s, 2H), 7.21–7.10 (m, 3H), 3.22 (sept, *J* = 6.9 Hz, 2H), 1.35 (s, 9H), 1.20 (d, *J* = 6.9 Hz, 12H).

**13C{1H} NMR** (100.66 MHz, CD_2_Cl_2_, 300 K): *δ*[ppm] = 161.9 (s, CH, 1C), 156.4 (s, Cq, 1C), 149.1 (s, Cq, 1C), 138.1 (s, Cq, 2C), 131.9 (s, Cq, 1C), 131.9 (s, CH, 2C), 125.0 (s, CH, 1C), 124.1 (s, Cq, 2C), 123.5 (s, CH, 2C), 35.4 (s, Cq, 1C), 31.0 (s, CH_3_, 3C), 28.1 (s, CH, 2C), 24.1 (s, CH_3_, 4C).

**IR (ATR):** ν[cm^-1^] = 2962, 2926, 2868, 1639, 1587, 1520, 1459, 1383, 1320, 1261, 1230, 1198, 1178, 1108, 1059, 1042, 932, 899, 875, 797, 766, 746, 730, 706, 637.

**Mass spectrometry (MS-ESI, +)**: *calcd.* for [M+H]^+^ C_23_H_30_Br_2_N: 478.0740, *found*: 478.0735.

**m.p.** [°C]: 120 °C.

***N*-(adamantan-1-yl)-1-(2,6-dibromophenyl)methanimine (1h)**

According to **GP2**. Imine **(1h)** was obtained as orange solid. Yield: 98 % (5.84 g, 14.7 mmol). Colorless single crystals were obtained from DCM.

**^1^H NMR** (400.33 MHz, CD_2_Cl_2_, 300 K): *δ* [ppm] = 8.18 (s, 1H), 7.56 (d, *J* = 8.0 Hz, 2H), 7.06 (t, *J* = 8.0 Hz, 1H), 2.22–2.15 (m, 3H), 1.90–1.86 (m, 5H), 1.82–1.70 (m, 7H).

**13C{1H} NMR** (100.66 MHz, CD_2_Cl_2_, 300 K): *δ*[ppm] = 154.3 (s, CH, 1C), 138.0 (s, Cq, 1C), 132.6 (s, CH, 2C), 130.8 (s, CH, 1C), 123.4 (s, Cq, 2C), 59.5 (s, Cq, 1C), 43.1 (s, CH_2_, 3C), 37.0 (s, CH_2_, 3C), 30.1 (s, CH, 3C).

**IR (ATR):** ν[cm^-1^] = 3056, 2903, 2844, 1653, 1575, 1547, 1451, 1419, 1367, 1342, 1307, 1261, 1190, 1142, 1117, 1089, 1063, 1041, 980, 913, 866, 813, 776, 720, 662.

**Mass spectrometry (MS-EI, +)**: *calcd.* C_17_H_19_Br_2_N: 394.9879, *found:* 394.9870.

**m.p.** [°C]: 95.

***N-tert-*butyl-1-(2,6-dibromophenyl)methanimine (1i)**

According to **GP2**. Imine **(1i)** was obtained as orange to red liquid. Yield: 97 % (4.63 g,
14.5 mmol).

**^1^H NMR** (400.33 MHz, CD_2_Cl_2_, 300 K): *δ* [ppm] = 8.19 (s, 1H), 7.57 (d, *J* = 8.0 Hz, 2H), 7.07 (t, *J* = 8.0 Hz, 1H), 1.34 (s, 9H).

**13C{1H} NMR** (100.66 MHz, CD_2_Cl_2_, 300 K): *δ*[ppm] = 154.6 (s, CH, 1C), 137.9 (s, Cq, 1C), 132.6 (s, CH, 2C), 130.9 (s, CH, 1C), 123.3 (s, Cq, 2C), 59.1 (s, Cq, 1C), 29.4 (s, CH_3_, 3C).

**IR (ATR):** ν[cm^-1^] = 2968, 2929, 2901, 2867, 1932, 1860, 1788, 1714, 1654, 1574, 1549, 1473, 1423, 1388, 1365, 1204, 1189, 1146, 1062, 937, 907, 772, 720.

**Mass spectrometry (MS-EI, +)**: *calcd.* C_11_H_13_Br_2_N: 316.9409, *found:* 316.9387.

**1-(2,6-dibromophenyl)-*N*-isopropylmethanimine (1j)**

According to **GP2**. Imine **(1j)** was obtained as orange liquid. Yield: 96 % (4.39 g,
14.4 mmol).

**^1^H NMR** (400.33 MHz, CD_2_Cl_2_, 300 K): *δ* [ppm] = 8.26 (s, 1H), 7.58 (d, *J* = 8.1 Hz, 2H), 7.08 (t, *J* = 8.0 Hz, 1H), 3.66 (sept, *J* = 6.2 Hz, 1H), 1.29 (d, *J* = 6.2 Hz, 6H).

**13C{1H} NMR** (100.66 MHz, CD_2_Cl_2_, 300 K): *δ*[ppm] = 156.8 (s, CH, 1C), 137.2 (s, Cq, 1C), 132.7 (s, CH, 2C), 131.1 (s, CH, 1C), 123.4 (s, Cq, 2C), 62.7 (s, CH, 1C), 24.0 (s, CH_3_, 2C).

**IR (ATR):** ν[cm^-1^] = 2968, 2926, 2865, 2603, 1652, 1573, 1550, 1464, 1423, 1381, 1362, 1314, 1259, 1190, 1146, 1063, 938, 887, 773, 722, 640.

**Mass spectrometry (MS-EI, +)**: *calcd.* C_10_H_11_Br_2_N: 302.9253, *found:* 302.9257.

**GP 3. Synthesis of *N*-aryl-4-bromo-isoindolium salts (2a)** – **(2g)**

In analogy to Rao et al.^[6a+b]^, *N*-aryl imine (**1**) (24.0 mmol, 1.00 equiv.) was dissolved/suspended in anhydrous diethyl ether (350 mL) and the suspension was cooled to
–78 °C. *n*-BuLi (9.56 mL, 2.5 M in hexanes, 1.53 g, 24.0 mmol, 1.00 equiv.) was added dropwise over 5 mins and the mixture was stirred for 1 h at –­­­­­­­­­78 °C. Then, a solution of benzophenone (14.3 mL, 1.75 M in diethyl ether, 4.57 g, 25.0 mmol, 1.05 equiv.) was added at –78 °C. The resulting mixture was allowed to warm to rt and was stirred for 45 min at rt. After cooling to –78 °C again, trifluoromethanesulfonic anhydride (4.22 mL, 7.08 g, 25.0 mmol, 1.05 equiv.) was added dropwise over 20 mins. The resulting mixture was allowed to warm to rt and was stirred for another 1 h at rt. The precipitate was filtered off and washed with diethylether (5 x 20 mL). The product was extracted with chloroform and the solvent was removed under reduced pressure to give the title compounds.

Note: The synthesis of isoindolium salt **(2d)** was performed at 7.14 mmol scale (see procedure) starting with imine (**1d**) under the same conditions as the other isoindolium salts.

**GP 4: Synthesis of *N*-alkyl-4-bromo-isoindolium salts (2h)** – **(2j)**

A solution or suspension of *N-*alkyl imine (14.7 mmol, 1.00 equiv.) in diethyl ether (150 mL) was cooled to –­­­­­­­­­78 °C and *n-*BuLi (5.88 mL, 2.5 M in hexanes, 0.94 g, 14.7 mmol, 1.00 equiv.) was added dropwise over 5 mins. Then, the reaction mixture was stirred for 1 h at –­­­­­­­­­78 °C. After that, benzophenone (8.82 mL, 1.75 M in diethyl ether, 2.81 g, 15.4 mmol, 1.05 equiv.) was added at –78 °C. The mixture was allowed to warm to rt and stir for 45 mins at rt. At –78 °C, trifluoromethanesulfonic anhydride (2.59 mL, 4.36 g, 15.4 mmol, 1.05 equiv.) was added dropwise over 15 mins. Allowing the reaction to warm to rt again, the mixture was stirred for
1 h at rt. The product precipitated from the solution and was filtered off and washed with diethyl ether (5 x 15 mL). The precipitate was extracted with chloroform and the solvent was removed *in vacuo* to yield the title compounds.

**4-bromo-2-(2,6-diisopropylphenyl)-1,1-diphenyl-1*H*-isoindol-2-ium triflate (2a)**

According to **GP3**. The isoindolium salt **(2a)** was obtained as beige to brown solid. Yield:
86 % (13.6 g, 20.7 mmol). Brown single crystals of **(2a)** were obtained by slow diffusion from DCM.

**1H NMR** (400.33 MHz, CDCl_3_, 300 K): *δ*[ppm] = 9.69 (s, 1H), 7.91 (d, *J* = 8.0 Hz, 1H), 7.83 (t,
*J* = 8.0 Hz, 1H), 7.46−7.36 (m, 3H), 7.33−7.27 (m, 4H), 7.18 (d, *J* = 7.6 Hz, 1H), 7.12 (d, *J* = 7.8 Hz, 2H), 7.08−7.02 (m, 4H), 2.13 (sept, *J* = 6.6 Hz, 2H), 1.06 (d, *J* = 6.6 Hz, 6H), 0.22 (d, *J* = 6.6 Hz, 6H).

**13C{1H} NMR** (100.66 MHz, CDCl_3_, 300 K): *δ*[ppm] = 175.7 (s, CH, 1C), 157.2 (s, Cq, 1C), 146.7 (s, Cq, 2C), 139.3 (s, CH, 1C), 134.7 (s, CH, 1C), 133.1 (s, Cq, 1C), 132.3 (s, CH, 1C), 130.8 (s, CH, 2C), 130.7 (s, Cq, 1C), 130.2 (s, CH, 4C), 129.3 (s, CH, 4C), 125.5 (s, CH, 2C), 125.3 (s, CH, 1C), 125.1 (s, Cq, 1C), 120.8 (q, *J* = 321 Hz, CF_3_, 1C), 97.5 (s, Cq, 1C), 30.6 (s, CH, 2C), 26.1 (s, CH_3_, 2C), 21.9 (s, CH_3_, 2C). *1 quaternary carbon signal (2C) could not be detected.*

**IR (ATR):** ν[cm^-1^] = 2970, 2931, 2867, 1597, 1542, 1492, 1450, 1355, 1260, 1220, 1153, 1028, 935, 813, 798, 770, 754, 738, 704, 671, 634.

**Mass spectrometry (MS-ESI, +)**: *calcd.* for [M–OTf]^+^ C_32_H_31_BrN^+^ : 508.1634, *found:* 508.1631.

**m.p.** [°C]: 245.

**4-bromo-2-(2,6-diethylphenyl)-1,1-diphenyl-1*H*-isoindol-2-ium triflate (2b)**

According to **GP3**. The title compound **(2b)** was obtained as colorless to pale yellow powder. Colorless crystals were obtained by slow diffusion from DCM. Yield: 82 % (12.4 g, 18.9 mmol).

**1H NMR** (600.24 MHz, CDCl_3_, 300 K): *δ*[ppm] = 9.79 (s, 1H), 7.92 (d, *J* = 7.9 Hz, 1H), 7.82 (t,
*J* = 7.9 Hz, 1H), 7.44−7.40 (m, 2H), 7.38 (t, *J* = 7.8 Hz, 1H), 7.29 (t, *J* = 7.9 Hz, 4H), 7.21 (d,
*J* = 7.8 Hz, 1H), 7.13−7.08 (m, 4H), 7.06 (d, *J* = 7.9 Hz, 2H), 2.05−1.97 (m, 2H), 1.58−1.50 (m, 2H), 0.69 (t, *J* = 7.5 Hz, 6H).

**13C{1H} NMR** (150.93 MHz, CDCl_3_, 300 K): *δ*[ppm] = 176.2 (s, CH, 1C), 157.2 (s, Cq, 1C), 141.9 (s, Cq, 2C), 138.9 (s, CH, 1C), 134.7 (s, CH, 1C), 134.5 (s, Cq, 1C), 131.8 (s, CH, 1C), 131.1 (s, Cq, 1C), 130.9 (s, CH, 2C), 130.5 (s, Cq, 2C), 130.3 (s, CH, 4C), 129.1 (s, CH, 4C), 127.3 (s, CH, 2C), 125.2 (s, Cq, 1C), 125.0 (s, CH, 1C), 120.8 (q, *J* = 321 Hz, CF_3_, 1C), 97.2 (s, Cq, 1C), 25.0 (s, CH_2_, 2C), 14.1 (s, CH_3_, 2C).

**IR (ATR):** ν[cm^-1^] = 3082, 3048, 2961, 2931, 2873, 1601, 1547, 1494, 1454, 1363, 1271, 1259, 1223, 1154, 1030, 802, 777, 765, 754, 742, 706, 671, 655, 636.

**Mass spectrometry (MS-ESI, +)**: *calcd.* for [M–OTf]^+^ C_30_H_27_BrN^+^: 480.1321, *found:* 480.1323.

**m.p.** [°C]: 231.

**4-bromo-2-(2,6-diethyl-4-methylphenyl)-1,1-diphenyl-1*H*-isoindol-2-ium triflate (2c)**

According to **GP3**. The product **(2c)** was obtained as pale yellow foam. Yield: 89 % (13.8 g, 21.4 mmol).

**1H NMR** (600.24 MHz, CDCl_3_, 300 K): *δ*[ppm] = 9.73 (s, 1H), 7.92 (d, *J* = 8.0 Hz, *J* = 0.8 Hz, 1H), 7.81 (t, *J* = 7.8 Hz, 1H), 7.44−7.36 (m, 2H), 7.29 (t, *J* = 7.9 Hz, 4H), 7.20 (d, *J* = 7.8 Hz, 1H), 7.13−7.09 (m, 4H), 6.85 (s, 2H), 2.31 (s, 3H), 2.00−1.87 (m, 2H), 1.55−1.45 (m, 2H), 0.68 (t, *J* = 7.5 Hz, 6H).

**13C{1H} NMR** (150.93 MHz, CDCl_3_, 300 K): *δ*[ppm] = 176.1 (s, CH, 1C), 157.2 (s, Cq, 1C), 142.0 (s, Cq, 1C), 141.5 (s, Cq, 2C), 138.8 (s, CH, 1C), 134.7 (s, CH, 1C), 132.2 (s, Cq, 1C), 131.2 (s, Cq, 1C), 130.8 (s, CH, 2C), 130.7 (s, Cq, 2C), 130.3 (s, CH, 4C), 129.1 (s, CH, 4C), 128.1 (s, CH, 2C), 125.1 (s, Cq, 1C), 125.0 (s, CH, 1C), 120.9 (q, *J* = 321 Hz, CF_3_, 1C), 97.1 (s, Cq, 1C), 24.9 (s, CH_2_, 2C), 21.4 (s, CH_3_, 1C), 14.1 (s, CH_3_, 2C).

**IR (ATR):** ν[cm^-1^] = 2968, 2934, 2879, 1605, 1543, 1492, 1453, 1358, 1274, 1247, 1222, 1169, 1023, 867, 803, 758, 743, 707, 674, 659, 632.

**Mass spectrometry (MS-ESI, +)**: *calcd.* for [M–OTf]^+^ C_31_H_29_BrN^+^: 494.1478, *found:* 494.1477.

**m.p.** [°C]: 186.

**4-bromo-2-(2,6-di(pentan-3-yl)phenyl)-1,1-diphenyl-1*H*-isoindol-2-ium triflate (2d)**

According to **GP3** (at 20.2 mmol scale). The pale yellow precipitate was filtered off and washed with diethylether (5 x 20 mL) and extracted with chloroform. The isoindolium salt **(2d)** was obtained as pale yellow foam. Yellow crystals were obtained by slow diffusion from DCM. Yield: 86 % (12.4 g, 17.3 mmol).

**1H NMR** (400.33 MHz, CDCl_3_, 300 K): *δ*[ppm] = 9.44 (s, 1H), 8.02−7.90 (m, 2H), 7.47 (t, *J* = 7.8 Hz, 1H), 7.44−7.30 (m, 6H), 7.22 (d, *J* = 7.5 Hz, 1H), 7.08 (d, *J* = 7.8 Hz, 2H), 7.06−6.99 (m, 4H), 1.87−1.78 (m, 2H), 1.64−1.52 (m, 2H), 1.41−1.28 (m, 2H), 1.19−1.07 (m, 2H), 0.72 (t,
*J* = 7.4 Hz, 6H), 0.33 (t, *J* = 7.4 Hz, 6H), 0.20−0.06 (m, 2H).

**13C{1H} NMR** (100.66 MHz, CDCl_3_, 300 K): *δ*[ppm] = 175.0 (s, CH, 1C), 157.4 (s, Cq, 1C), 144.7 (s, Cq, 2C), 140.5 (s, CH, 1C), 135.4 (s, Cq, 1C), 135.0 (s, CH, 1C), 132.0 (s, CH, 1C), 130.9 (s, CH, 2C), 130.8 (s, Cq, 2C), 130.1 (s, Cq, 1C), 130.0 (s, CH, 4C), 129.7 (s, CH, 4C), 126.7 (s, CH, 2C), 125.8 (s, CH, 1C), 124.9 (s, Cq, 1C), 121.0 (q, *J* = 321 Hz, CF_3_, 1C), 98.2 (s, Cq, 1C), 43.1 (s, CH, 2C), 28.7 (s, CH_2_, 2C), 26.7 (s, CH_2_, 2C), 12.1 (s, CH_3_, 2C), 11.2 (s, CH_3_, 2C).

**IR (ATR):** ν[cm^-1^] = 3072, 2968, 2936, 2876, 1599, 1537, 1493, 1454, 1361, 1255, 1222, 1150, 1030, 800, 768, 753, 735, 704, 658, 635.

**Mass spectrometry (MS-ESI, +)**: *calcd.* for [M–OTf]^+^ C_36_H_39_BrN^+^: 566.2240, *found:* 566.2244.

**m.p.** [°C]: 215 (decomp.).

**4-bromo-2-(2,6-diisopropylphenyl)-5,7-dimethoxy-1,1-diphenyl-1*H*-isoindol-2-ium triflate (2f)**

According to **GP3**. The product **(2f)** was obtained as yellow to brown solid. Yield: 53 %
(9.14 g, 12.7 mmol).

**^1^H NMR** (400.33 MHz, CD_2_Cl_2_, 300 K): *δ* [ppm] = 9.38 (s, 1H), 7.50 (t, *J* = 7.9 Hz, 1H), 7.44 (t, *J* = 7.5 Hz, 2H), 7.35–7.29 (m, 4H), 7.18 (d, *J* = 7.9 Hz, 2H), 7.13 (s, 1H), 7.06–7.00 (m, 4H), 4.13 (s, 3H), 3.61 (s, 3H), 2.08 (sept, *J* = 6.7 Hz, 2H), 1.03 (d, *J* = 6.8 Hz, 6H), 0.30 (d, *J* =
6.7 Hz, 6H).

**13C{1H} NMR** (100.66 MHz, CD_2_Cl_2_, 300 K): *δ*[ppm] = 174.6 (s, CH, 1C), 160.8 (s, Cq, 1C), 155.3 (s, Cq, 1C), 146.8 (s, Cq, 2C), 136.0 (s, Cq, 1C), 133.4 (s, Cq, 1C), 132.9 (s, CH, 1C), 131.6 (s, Cq, 1C), 131.0 (s, CH, 2C), 130.8 (bs, CH, 4C), 129.6 (s, Cq, 2C), 129.1 (s, CH, 4C), 125.9 (s, CH, 2C), 107.7 (s, CH, 1C), 103.0 (s, Cq, 1C), 98.8 (s, Cq, 1C), 78.0 (s, Cq, 1C), 58.3 (s, CH_3_, 1C), 57.3 (s, CH_3_, 1C), 30.9 (s, CH, 2C), 26.3 (s, CH_3_, 2C), 21.8 (s, CH_3_, 2C).

**IR (ATR):** ν[cm^-1^] = 3077, 2974, 2959, 1560, 1491, 1471, 1450, 1438, 1361, 1322, 1281, 1250, 1222, 1151, 1106, 1085, 1029, 973, 935, 863, 808, 773, 700, 736, 700, 658, 634.

**Mass spectrometry (MS-ESI, +)**: *calcd.* for [M–OTf]^+^ C_34_H_35_BrNO_2_^+^: 568.1846, *found*: 568.1848.

**m.p.** [°C]: 239 (decomp.).

**4-bromo-6-(*tert*-butyl)-2-(2,6-diisopropylphenyl)-1,1-diphenyl-1*H*-isoindol-2-ium triflate (2g)**

According to **GP3**. The product **(2g)** was obtained as pale yellow solid. Yield: 90 % (13.8 g, 21.4 mmol).

**^1^H NMR** (400.33 MHz, CD_2_Cl_2_, 300 K): *δ* [ppm] = 9.51 (s, 1H), 8.03 (d, *J* = 1.2 Hz, 1H), 7.53–7.43 (m, 3H), 7.38–7.32 (m, 4H), 7.22–7.17 (m, 3H), 7.07–7.02 (m, 4H), 2.10 (sept, *J* = 6.7 Hz, 2H), 1.34 (s, 9H), 1.06 (d, *J* = 6.8 Hz, 6H), 0.27 (d, *J* = 6.7 Hz, 6H).

**13C{1H} NMR** (100.66 MHz, CD_2_Cl_2_, 300 K): *δ*[ppm] = 174.1 (s, CH, 1C), 166.6 (s, Cq, 1C), 158.1 (s, Cq, 1C), 146.9 (s, Cq, 2C), 133.3 (s, Cq, 1C), 132.9 (s, CH, 1C), 132.8 (s, CH, 1C), 131.2 (s, CH, 2C), 131.0 (s, Cq, 2C), 130.4 (s, CH, 4C), 129.6 (s, CH, 4C), 128.7 (s, Cq, 1C), 125.9 (s, CH, 2C), 124.6 (s, Cq, 1C), 123.2 (s, CH, 1C), 121.3 (q, *J* = 321 Hz, CF_3_, 1C), 98.0 (s, Cq, 1C), 37.1 (s, Cq, 1C), 30.9 (s, CH_3_, 3C), 30.9 (s, CH, 2C), 26.2 (s, CH_3_, 2C), 21.9 (s, CH_3_, 2C).

**IR (ATR):** ν[cm^-1^] = 2966, 2931, 2872, 1602, 1528, 1454, 1404, 1367, 1255, 1224, 1146, 1090, 1030, 934, 880, 810, 753, 703, 651, 635.

**Mass spectrometry (MS-ESI, +)**: *calcd.* for [M–OTf]^+^ C_36_H_39_BrN^+^: 564.2268, *found*: 564.2260.

**m.p.** [°C]: 250 (decomp.).

**2-(adamantan-1-yl)-4-bromo-1,1-diphenyl-1*H*-isoindol-2-ium triflate (2h)**

According to **GP4**. Isoindolium salt **(2h)** was obtained as yellow solid. Colorless crystals were obtained by slow diffusion from DCM. Yield: 80 % (7.40 g, 11.7 mmol).

**1H NMR** (400.33 MHz, CDCl_3_, 300 K): *δ*[ppm] = 10.15 (s, 1H), 7.62 (d, *J* = 7.2 Hz, 1H), 7.52−7.44 (m, 11H), 7.04 (d, *J* = 7.7 Hz, 1H), 2.13−2.04 (m, 9H), 1.58−1.50 (m, 6H).

**13C{1H} NMR** (100.66 MHz, CDCl_3_, 300 K): *δ*[ppm] = 171.5 (s, CH, 1C), 159.7 (s, Cq, 1C), 138.5 (s, CH, 1C), 133.8 (s, CH, 1C), 132.4 (s, Cq, 2C), 130.7 (s, CH, 2C), 130.1 (s, Cq, 1C), 129.7 (s, CH, 4C), 128.7 (s, CH, 4C), 123.5 (s, Cq, 1C), 121.8 (s, CH, 1C), 120.9 (q, *J* =
321 Hz, CF_3_, 1C), 93.3 (s, Cq, 1C), 72.9 (s, Cq, 1C), 42.5 (s, CH_2_, 3C), 34.9 (s, CH_2_, 3C), 30.2 (s, CH, 3C).

**IR (ATR):** ν[cm^-1^] = 3068, 2915, 2855, 1695, 1600, 1544, 1494, 1454, 1360, 1347, 1255, 1222, 1156, 1058, 1028, 794, 758, 706, 656, 635.

**Mass spectrometry (MS-ESI, +)**: *calcd.* for [M–OTf]^+^ C_30_H_29_BrN^+^: 482.1478, *found:* 482.1479.

**m.p.** [°C]: 199.

**4-bromo-2-(tert-butyl)-1,1-diphenyl-1*H*-isoindol-2-ium triflate (2i)**

According to **GP4**. Colorless crystals were obtained by slow diffusion from DCM. The title compound was obtained as colorless solid. Yield: 67 % (5.46 g, 9.85 mmol).

**1H NMR** (400.33 MHz, CD_3_CN, 300 K): *δ*[ppm] = 9.68 (s, 1H), 7.79 (d, *J* = 8.0 Hz, 1H), 7.61 (t, *J* = 8.0 Hz, 1H), 7.56−7.45 (m, 10H), 7.26 (d, *J* = 8.0 Hz, 1H), 1.43 (s, 9H).

**13C{1H} NMR** (100.66 MHz, CD_3_CN, 300 K): *δ*[ppm] = 173.1 (s, CH, 1C), 160.8 (s, Cq, 1C), 139.8 (s, CH, 1C), 134.6 (s, CH, 1C), 133.3 (s, Cq, 2C), 131.4 (s, CH, 2C), 130.8 (s, Cq, 1C), 130.3 (s, CH, 4C), 129.8 (s, CH, 4C), 123.2 (s, CH, 1C), 123.1 (s, Cq, 1C), 121.8 (q, *J* = 320 Hz, CF_3_, 1C), 94.2 (s, Cq, 1C), 70.8 (s, Cq, 1C), 30.8 (s, CH_3_, 3C).

**IR (ATR):** ν[cm^-1^] = 3073, 2996, 2953, 1598, 1547, 1496, 1456, 1411, 1384, 1360, 1288, 1251, 1231, 1174, 1049, 1006, 978, 935, 884, 794, 764, 740, 704, 675, 646, 636.

**Mass spectrometry (MS-ESI, +)**: *calcd.* for [M–OTf]^+^ C_24_H_23_BrN^+^: 404.1008, *found:* 404.1010.

**m.p.** [°C]: 153.

**4-bromo-2-isopropyl-1,1-diphenyl-1*H*-isoindol-2-ium triflate (2j)**

According to **GP4**. Colorless crystals were obtained by slow diffusion from DCM. Isoindolium salt **(2j)** was obtained as colorless to pale yellow solid. Yield: 56 % (4.45 g, 8.23 mmol).

**1H NMR** (400.33 MHz, CD_3_CN, 300 K): *δ*[ppm] = 9.65 (s, 1H), 7.87 (dd, *J* = 8.0 Hz, *J* = 0.6 Hz, 1H), 7.71 (t, *J* = 8.0 Hz, 1H), 7.58−7.43 (m, 7H), 7.33−7.29 (m, 4H), 4.39 (sept, *J* = 6.6 Hz, 1H), 1.24 (d, *J* = 6.6 Hz, 6H).

**13C{1H} NMR** (100.66 MHz, CD_3_CN, 300 K): *δ*[ppm] = 170.4 (s, CH, 1C), 157.0 (s, Cq, 1C), 139.3 (s, CH, 1C), 134.9 (s, CH, 1C), 134.3 (s, Cq, 2C), 132.6 (s, Cq, 1C), 131.6 (s, CH, 2C), 130.6 (s, CH, 4C), 129.3 (s, CH, 4C), 124.1 (s, CH, 1C), 123.4 (s, Cq, 1C), 121.7 (q, *J* =
320 Hz, CF_3_, 1C), 93.6 (s, Cq, 1C), 55.8 (s, CH, 1C), 23.4 (s, CH_3_, 2C).

**IR (ATR):** ν[cm^-1^] = 1609, 1566, 1494, 1454, 1402, 1283, 1252, 1232, 1175, 1157, 1107, 1051, 982, 795, 763, 742, 718, 701, 637.

**Mass spectrometry (MS-ESI, +)**: *calcd.* for [M–OTf]^+^ C_23_H_21_BrN^+^: 390.0852, *found:* 390.0853.

**m.p.** [°C]: 182 (decomp.).

**GP 5. Synthesis of 4-bromo-3-methoxyisoindolines (3)**

According to an adapted procedure of Lorkowski et al.^[7b]^, the isoindolium salt (**3**) (1.00 equiv.) was dissolved/suspended in dry methanol (0.4 M) and an excess of sodium methoxide (5.0 M in MeOH, 4.00 equiv.) was added at rt. The suspension was stirred for 18 h at rt and the solid was filtered off. The methoxyisoindoline was washed with dry methanol and dried *in vacuo*overnight*.*

Note: *N*-alkyl methoxyisoindolines were measured in dry NMR solvents due to its sensitivity towards hydrolysis.

**4-bromo-2-(2,6-diisopropylphenyl)-3-methoxy-1,1-diphenylisoindoline (3a)**

According to **GP5** (at 26.4 mmol scale), the crude product was washed with dry methanol (5 x 15 mL). The title compound was obtained as colorless to beige solid. Yield: 95 % (13.5 g, 25.0 mmol).

**1H NMR** (300.51 MHz, CDCl_3_, 295 K): *δ* [ppm] = 7.54–7.40 (m, 3H), 7.21–7.02 (m, 9H), 6.97–6.75 (m, 4H), 5.92 (s, 1H), 3.87 (sept, *J* = 6.8 Hz, 1H), 3.57 (s, 3H), 1.99 (sept, *J* = 6.8 Hz, 1H), 1.16 (d, *J* = 6.8 Hz, 3H), 1.12 (d, *J* = 6.8 Hz, 3H), 0.47 (d, *J* = 6.7 Hz, 3H), –0.06 (d, *J* = 6.7 Hz, 3H).

**13C{1H} NMR** (125.78 MHz, CDCl_3_, 300 K): *δ*[ppm] = 151.3 (s, Cq, 1C), 149.7 (s, Cq, 1C), 148.9 (s, Cq, 1C), 148.3 (s, Cq, 1C), 141.8 (s, Cq, 1C), 138.0 (s, Cq, 1C), 137.7 (s, Cq, 1C), 131.0 (s, CH, 1C), 130.6 (bs, CH, 2C), 130.1 (s, CH, 1C), 129.4 (bs, CH, 2C), 127.5 (s, CH, 2C), 127.4 (s, CH, 1C), 127.3 (bs, CH, 2C), 127.3 (s, CH, 1C), 126.6 (s, CH, 1C), 125.7 (s, CH, 1C), 124.4 (s, CH, 1C), 124.3 (s, CH, 1C), 119.3 (s, Cq, 1C), 102.6 (s, CH, 1C), 82.8 (s, Cq, 1C), 59.0 (s, CH_3_, 1C), 28.9 (s, CH, 2C), 26.3 (s, CH_3_, 1C), 24.9 (s, CH_3_, 1C), 23.4 (s, CH_3_, 1C), 23.3 (s, CH_3_, 1C).

**IR (ATR):** ν[cm^-1^] = 3055, 2961, 2927, 2867, 2826, 1595, 1580, 1493, 1444, 1382, 1362, 1326, 1254, 1222, 1172, 1135, 1114, 1062, 994, 962, 912, 898, 814, 782, 767, 755, 730, 702, 637.

**Mass spectrometry (MS-EI, +)**: *calcd.* C_33_H_34_BrNO: 541.1798, *found:* 541.1803.

**m.p.** [°C]: 207 (decomp.).

**4-bromo-2-(2,6-diethylphenyl)-3-methoxy-1,1-diphenylisoindoline (3b)**

According to **GP5** (at 19.0 mmol scale), the crude product was washed with dry methanol (5 x 11 mL). The product was obtained as colorless powder. Yield: 94 % (9.06 g, 17.7 mmol).

**1H NMR** (400.33 MHz, CD_2_Cl_2_, 300 K): *δ*[ppm] = 7.70 (d, *J* = 7.7 Hz, 2H), 7.53 (d, *J* = 7.8 Hz, 1H), 7.29–7.10 (m, 5H), 7.07–6.97 (m, 4H), 6.94 (d, *J* = 7.7 Hz, 1H), 6.87 (d, *J* = 7.5 Hz, 2H), 6.74 (dd, *J* = 6.6 Hz, *J* = 2.4 Hz, 1H), 5.76 (s, 1H), 3.55 (s, 3H), 3.06–2.90 (m, 1H), 2.75–2.58 (m, 1H), 1.94–1.76 (m, 1H), 1.50–1.36 (m, 1H), 0.98 (t, *J* = 7.6 Hz, 3H), 0.80 (t, *J* = 7.6 Hz, 3H).

**13C{1H} NMR** (100.66 MHz, CD_2_Cl_2_, 300 K): *δ*[ppm] = 150.3 (s, Cq, 1C), 148.8 (s, Cq, 1C), 146.5 (s, Cq, 1C), 144.7 (s, Cq, 1C), 144.3 (s, Cq, 1C), 138.5 (s, Cq, 1C), 138.2 (s, Cq, 1C), 131.4 (s, CH, 1C), 131.0 (s, CH, 2C), 130.6 (s, CH, 1C), 129.4 (s, CH, 2C), 127.9 (s, CH, 2C), 127.5 (s, CH, 1C), 126.9 (s, CH, 5C), 126.2 (s, CH, 1C), 125.4 (s, CH, 1C), 119.7 (s, Cq, 1C), 103.1 (s, CH, 1C), 83.8 (s, Cq, 1C), 58.1 (s, CH_3_, 1C), 26.6 (s, CH_2_, 1C), 25.1 (s, CH_2_, 1C), 15.2 (s, CH_3_, 1C), 14.3 (s, CH_3_, 1C).

**IR (ATR):** ν[cm^-1^] = 3054, 2955, 2930, 2873, 2821, 1597, 1578, 1492, 1446, 1364, 1334, 1252, 1176, 1134, 1060, 1034, 995, 961, 934, 912, 900, 815, 782, 756, 742, 732, 703, 636.

**Mass spectrometry (MS-EI, +)**: *calcd.* C_31_H_30_BrNO: 511.1505, *found:* 511.1502.

**m.p.** [°C]: 162 (decomp.).

**4-bromo-2-(2,6-diethyl-4-methylphenyl)-3-methoxy-1,1-diphenylisoindoline (3c)**

According to **GP5** (at 18.3 mmol scale), the crude product was washed with dry methanol (5 x 10 mL). The product was obtained as colorless powder. Yield: 92 % (8.51 g, 16.2 mmol).

**1H NMR** (400.33 MHz, CD_2_Cl_2_, 300 K): *δ*[ppm] = 7.70–7.59 (m, 2H), 7.50 (dd, *J* = 7.8 Hz, *J* = 0.9 Hz, 1H), 7.26–7.09 (m, 5H), 7.02–6.96 (m, 2H), 6.90 (dd, *J* = 7.8 Hz, *J* = 0.9 Hz, 1H), 6.86–6.80 (m, 3H), 6.52 (d, *J* = 2.2 Hz, 1H), 5.68 (s, 1H), 3.51 (s, 3H), 2.95–2.82 (m, 1H), 2.67–2.54 (m, 1H), 2.19 (s, 3H), 1.85–1.72 (m, 1H), 1.40–1.27 (m, 1H), 0.92 (t, *J* = 7.6 Hz, 3H), 0.74 (t, *J* = 7.5 Hz, 3H).

**13C{1H} NMR** (100.66 MHz, CD_2_Cl_2_, 300 K): *δ*[ppm] = 150.4 (s, Cq, 1C), 149.0 (s, Cq, 1C), 146.0 (s, Cq, 1C), 143.8 (s, Cq, 1C), 142.0 (s, Cq, 1C), 138.6 (s, Cq, 1C), 138.3 (s, Cq, 1C), 136.2 (s, Cq, 1C), 131.3 (s, CH, 1C), 131.0 (s, CH, 2C), 130.6 (s, CH, 1C), 129.4 (s, CH, 2C), 127.9 (s, CH, 2C), 127.5 (s, CH, 1C), 127.4 (s, CH, 1C), 127.1 (s, CH, 1C), 126.9 (s, CH, 2C), 126.8 (s, CH, 1C), 125.4 (s, CH, 1C), 119.6 (s, Cq, 1C), 103.1 (s, CH, 1C), 83.6 (s, Cq, 1C), 58.0 (s, CH_3_, 1C), 26.5 (s, CH_2_, 1C), 25.0 (s, CH_2_, 1C), 21.2 (s, CH_3_, 1C), 15.2 (s, CH_3_, 1C), 14.3 (s, CH_3_, 1C).

**IR (ATR):** ν[cm^-1^] = 3060, 2992, 2956, 2928, 2873, 2820, 1598, 1578, 1493, 1446, 1362, 1334, 1231, 1212, 1176, 1159, 1130, 1059, 987, 960, 935, 918, 890, 861, 785, 756, 735, 708, 638.

**Mass spectrometry (MS-EI, +)**: *calcd.* C_32_H_32_BrNO: 525.1662, *found:* 525.1630.

**m.p.** [°C]: 171 (decomp.).

**4-bromo-2-(2,6-di(pentan-3-yl)phenyl)-3-methoxy-1,1-diphenylisoindoline (3d)**

According to **GP5** (at 16.5 mmol scale), the crude product was washed with dry methanol (5 x 15 mL). The product was obtained as colorless powder. Yield: 94 % (9.25 g, 15.5 mmol).

**1H NMR** (400.33 MHz, CD_2_Cl_2_, 300 K): *δ*[ppm] = 7.58–7.42 (m, 3H), 7.27–6.81 (m, 12H), 6.76 (dd, *J* = 7.6 Hz, *J* = 1.8 Hz, 1H), 5.93 (s, 1H), 3.55 (s, 3H), 3.48–3.39 (m, 1H), 1.85–1.73 (m, 1H), 1.68–1.51 (m, 4H), 1.32–1.09 (m, 2H), 0.96 (t, *J* = 7.4 Hz, 3H), 0.90 (t, *J* = 7.5 Hz, 3H), 0.84–0.71 (m, 1H), 0.49 (t, *J* = 7.5 Hz, 3H), 0.39 (t, *J* = 7.4 Hz, 3H), –0.64 to –0.77 (m, 1H).

**13C{1H} NMR** (100.66 MHz, CD_2_Cl_2_, 300 K): *δ*[ppm] = 150.3 (s, Cq, 1C), 150.1 (s, Cq, 1C), 149.7 (s, Cq, 1C), 149.0 (s, Cq, 1C), 143.2 (s, Cq, 1C), 138.4 (s, Cq, 1C), 138.3 (s, Cq, 1C), 131.3 (bs, CH, 1C), 131.3 (s, CH, 1C), 130.2 (s, CH, 1C), 129.4 (s, CH, 2C), 128.0 (s, CH, 2C), 127.7 (s, CH, 1C), 127.6 (bs, CH, 2C), 126.8 (s, CH, 1C), 126.5 (s, CH, 1C), 126.1 (s, CH, 1C), 125.9 (s, CH, 1C), 125.9 (s, CH, 1C), 119.4 (s, Cq, 1C), 103.7 (s, CH, 1C), 83.2 (s, Cq, 1C), 59.5 (s, CH_3_, 1C), 42.4 (s, CH, 1C), 40.4 (s, CH, 1C), 29.6 (s, CH_2_, 1C), 29.5 (s, CH_2_, 1C), 27.4 (s, CH_2_, 1C), 25.5 (s, CH_2_, 1C), 13.7 (s, CH_3_, 1C), 13.3 (s, CH_3_, 1C), 11.2 (s, CH_3_, 1C), 10.8 (s, CH_3_, 1C). *One tertiary carbon signal (1C) is missing.*

**IR (ATR):** ν[cm^-1^] = 3061, 2963, 2934, 2873, 2826, 1595, 1578, 1494, 1444, 1374, 1324, 1245, 1218, 1171, 1134, 1109, 1054, 994, 962, 911, 898, 806, 788, 779, 768, 757, 733, 716, 702, 638.

**Mass spectrometry (MS-EI, +)**: *calcd.* C_37_H_42_BrNO: 595.2444, *found:* 595.2439.

**m.p.** [°C]: 193 (decomp.).

**4-bromo-2-(2,6-diisopropylphenyl)-3,5,7-trimethoxy-1,1-diphenylisoindoline (3f)**

According to **GP5** (at 20.9 mmol scale), the crude product was washed with dry methanol (5 x 10 mL). The product was obtained as colorless powder. Yield: 83 % (10.4 g, 17.3 mmol).

**^1^H NMR** (400.33 MHz, CDCl_3_, 300 K): *δ* [ppm] = 7.54 (bs, 2H), 7.21–6.65 (m, 11H), 6.43 (s, 1H), 5.88 (s, 1H), 3.93 (s, 3H), 3.80 (sept, *J* = 6.9 Hz, 1H), 3.54 (s, 3H), 3.34 (s, 3H), 2.01 (sept, *J* = 6.7 Hz, 1H), 1.12 (d, *J* = 6.9 Hz, 3H), 1.11 (d, *J* = 6.9 Hz, 3H), 0.43 (d, *J* = 6.9 Hz, 3H), –0.04 (d, *J* = 6.9 Hz, 3H).

**13C{1H} NMR** (100.66 MHz, CDCl_3_, 300 K): *δ*[ppm] = 157.4 (s, Cq, 1C), 154.4 (s, Cq, 1C), 151.5 (s, Cq, 1C), 149.1 (s, Cq, 1C), 146.2 (s, Cq, 1C), 141.8 (s, Cq, 1C), 140.4 (s, Cq, 1C), 136.2 (s, Cq, 1C), 131.0 (bs, CH, 2C), 130.7 (s, CH, 2C), 130.4 (s, Cq, 1C), 127.3 (s, CH, 1C), 126.8 (s, CH, 1C), 126.7 (bs, CH, 2C), 126.5 (bs, CH, 2C), 126.0 (s, CH, 1C), 124.3 (s, CH, 1C), 124.3 (s, CH, 1C), 103.0 (s, CH, 1C), 99.1 (s, Cq, 1C), 98.8 (s, CH, 1C), 82.1 (s, Cq, 1C), 59.0 (s, CH_3_, 1C), 57.0 (s, CH_3_, 1C), 56.0 (s, CH_3_, 1C), 29.0 (s, CH, 1C), 28.9 (s, CH, 1C), 26.4 (s, CH_3_, 1C), 25.0 (s, CH_3_, 1C), 23.4 (s, CH_3_, 1C), 23.2 (s, CH_3_, 1C).

**IR (ATR):** ν[cm^-1^] = 3059, 2965, 2867, 1694, 1593, 1483, 1450, 1382, 1341, 1256, 1215, 1174, 1145, 1104, 1083, 1050, 995, 969, 922, 899, 861, 820, 773, 751, 732, 700, 624.

**Mass spectrometry (MS-ESI, +)**: *calcd.* for [M–OMe]^+^ C_34_H_35_BrNO_2_^+^: 568.1846, *found*: 568.1848.

**m.p.** [°C]: 225 (decomp.).

**4-bromo-6-(*tert*-butyl)-2-(2,6-diisopropylphenyl)-3-methoxy-1,1-diphenylisoindoline (3g)**

According to **GP5** (at 14.7 mmol scale), the crude product was washed with dry methanol (5 x 10 mL). The product was obtained as colorless powder. Yield: 88 % (7.71 g, 12.9 mmol).

**^1^H NMR** (400.33 MHz, CD_2_Cl_2_, 300 K): *δ* [ppm] = 7.58–7.45 (m, 3H), 7.22–7.02 (m, 8H), 6.93–6.73 (m, 4H), 5.86 (s, 1H), 3.89 (sept, *J* = 6.7 Hz, 1H), 3.54 (s, 3H), 2.01 (sept, *J* = 6.7 Hz, 1H), 1.23 (s, 9H), 1.17 (d, *J* = 6.8 Hz, 3H), 1.12 (d, *J* = 6.8 Hz, 3H), 0.51 (d, *J* = 6.8 Hz, 3H), –0.04 (d, *J* = 6.8 Hz, 3H).

**13C{1H} NMR** (100.66 MHz, CD_2_Cl_2_, 300 K): *δ*[ppm] = 154.3 (s, Cq, 1C), 151.7 (s, Cq, 1C), 149.8 (s, Cq, 1C), 149.4 (s, Cq, 1C), 149.0 (s, Cq, 1C), 142.6 (s, Cq, 1C), 138.4 (s, Cq, 1C), 135.9 (s, Cq, 1C), 131.0 (bs, CH, 2C), 129.6 (s, CH, 2C), 128.8 (s, CH, 1C), 127.7 (s, CH, 2C), 127.6 (m, CH, 3C), 127.4 (s, CH, 1C), 126.7 (s, CH, 1C), 124.7 (s, CH, 1C), 124.6 (s, CH, 1C), 123.0 (s, CH, 1C), 119.0 (s, Cq, 1C), 102.9 (s, CH, 1C), 83.3 (s, Cq, 1C), 59.0 (s, CH_3_, 1C), 35.3 (s, Cq, 1C), 31.4 (s, CH_3_, 3C), 29.2 (s, CH, 2C), 26.3 (s, CH_3_, 1C), 25.0 (s, CH_3_, 1C), 23.6 (s, CH_3_, 1C), 23.5 (s, CH_3_, 1C).

**IR (ATR):** ν[cm^-1^] = 3055, 2963, 2928, 2868, 2826, 1609, 1570, 1494, 1449, 1401, 1361, 1333, 1274, 1254, 1223, 1171, 1149, 1118, 1105, 1086, 1052, 1004, 973, 927, 927, 905, 875, 844, 813, 777, 755, 734, 703, 646, 621.

**Mass spectrometry (MS-ESI, +)**: *calcd.* for [M–OMe]^+^ C_36_H_39_BrN^+^: 564.2268, *found*: 564.2259.

**m.p.** [°C]: 212 (decomp.).

**2-(adamantan-1-yl)-4-bromo-3-methoxy-1,1-diphenylisoindoline (3h)**

According to **GP5** (at 10.0 mmol scale), the crude product was washed with dry methanol (5 x 6 mL). The product was obtained as colorless powder. Yield: 92 % (4.73 g, 9.2 mmol).

**1H NMR** (400.33 MHz, CD_2_Cl_2_, 300 K): *δ*[ppm] = 7.95–7.87 (m, 2H), 7.42–7.16 (m, 9H), 7.02 (t, *J* = 7.8 Hz, 1H), 6.81 (dd, *J* = 7.8 Hz, *J* = 0.9 Hz, 1H), 6.43 (s, 1H), 3.25 (s, 3H), 1.90–1.64 (m, 9H), 1.52–1.41 (m, 6H).

**13C{1H} NMR** (100.66 MHz, CD_2_Cl_2_, 300 K): *δ*[ppm] = 153.6 (s, Cq, 1C), 146.7 (s, Cq, 1C), 144.9 (s, Cq, 1C), 136.7 (s, Cq, 1C), 131.0 (s, CH, 1C), 130.8 (s, CH, 2C), 130.7 (s, CH, 1C), 130.6 (s, CH, 2C), 127.6 (s, CH, 2C), 127.6 (s, CH, 2C), 127.3 (s, CH, 1C), 127.2 (s, CH, 1C), 123.3 (s, CH, 1C), 117.8 (s, Cq, 1C), 94.1 (s, CH, 1C), 78.9 (s, Cq, 1C), 57.2 (s, Cq, 1C), 52.4 (s, CH_3_, 1C), 42.3 (s, CH_2_, 3C), 36.8 (s, CH_2_, 3C), 30.6 (s, CH, 3C).

**IR (ATR):** ν[cm^-1^] = 3083, 3058, 2988, 2897, 2845, 2822, 1582, 1490, 1448, 1360, 1334, 1308, 1210, 1198, 1173, 1130, 1102, 1055, 1040, 975, 955, 908, 872, 822, 780, 758, 736, 718, 704, 649, 630, 610.

**Mass spectrometry (MS-EI, +)**: *calcd.* C_31_H_32_BrNO: 513.1662, *found:* 513.1658.

**m.p.** [°C]: 194 (decomp.).

**4-bromo-2-(tert-butyl)-3-methoxy-1,1-diphenylisoindoline (3i)**

According to **GP5** (at 10.0 mmol scale), the crude product was washed with dry methanol (5 x 6 mL). The product was obtained as yellow powder. Yield: 71 % (3.10 g, 7.1 mmol).

**1H NMR** (400.33 MHz, CD_2_Cl_2_, 300 K): *δ*[ppm] = 7.92–7.85 (m, 2H), 7.42–7.17 (m, 9H), 7.04 (t, *J* = 7.8 Hz, 1H), 6.88 (d, *J* = 7.8 Hz, 1H), 6.33 (s, 1H), 3.25 (s, 3H), 1.06 (s, 9H).

**13C{1H} NMR** (100.66 MHz, CD_2_Cl_2_, 300 K): *δ*[ppm] = 153.4 (s, Cq, 1C), 146.0 (s, Cq, 1C), 144. 8 (s, Cq, 1C), 136.8 (s, Cq, 1C), 131.2 (s, CH, 1C), 130.8 (s, CH, 1C), 130.7 (s, CH, 2C), 130.6 (s, CH, 2C), 127.7 (s, CH, 2C), 127.7 (s, CH, 2C), 127.4 (s, CH, 1C), 127.3 (s, CH, 1C), 123.4 (s, CH, 1C), 117.9 (s, Cq, 1C), 95.3 (s, CH, 1C), 79.2 (s, Cq, 1C), 55.2 (s, Cq, 1C), 52.2 (s, CH_3_, 1C), 30.8 (s, CH_3_, 3C).

**IR (ATR):** ν[cm^-1^] = 3087, 3059, 3022, 2965, 2876, 2822, 1583, 1493, 1448, 1397, 1370, 1356, 1327, 1299, 1255, 1211, 1176, 1131, 1106, 1084, 1051, 1031, 1015, 957, 911, 875, 785, 766, 736, 707, 647, 632.

**Mass spectrometry (MS-ESI, +)**: *calcd.* as [M–OMe]^+^ C_24_H_23_BrN^+^: 404.1008, *found:* 404.1006.

**m.p.** [°C]: 141 (decomp.).

**4-bromo-2-isopropyl-3-methoxy-1,1-diphenylisoindoline (3j)**

According to **GP5** (at 10.0 mmol scale), the crude product was washed with dry methanol (5 x 6 mL). The product was obtained as red-brownish powder. Yield: 74 % (3.12 g, 7.4 mmol).

**1H NMR** (400.33 MHz, CD_2_Cl_2_, 300 K): *δ*[ppm] = 7.60–7.55 (m, 2H), 7.47 (d, *J* = 7.7 Hz, 1H), 7.41–7.31 (m, 3H), 7.28–7.20 (m, 3H), 7.15 (t, *J* = 7.7 Hz, 1H), 7.08–6.99 (m, 3H), 6.25 (s, 1H), 3.35 (sept, *J* = 6.7 Hz, 1H), 3.04 (s, 3H), 1.02 (d, *J* = 6.7 Hz, 3H), 0.83 (d, *J* = 6.7 Hz, 3H).

**13C{1H} NMR** (100.66 MHz, CD_2_Cl_2_, 300 K): *δ*[ppm] = 150.3 (s, Cq, 1C), 145.8 (s, Cq, 1C), 144.9 (s, Cq, 1C), 137.3 (s, Cq, 1C), 131.6 (s, CH, 1C), 130.8 (s, CH, 1C), 129.2 (s, CH, 2C), 129.1 (s, CH, 2C), 128.3 (s, CH, 4C), 127.6 (s, CH, 1C), 127.4 (s, CH, 1C), 123.8 (s, CH, 1C), 118.8 (s, Cq, 1C), 91.6 (s, CH, 1C), 79.3 (s, Cq, 1C), 49.5 (s, CH_3_, 1C), 46.0 (s, CH, 1C), 24.0 (s, CH_3_, 1C), 18.6 (s, CH_3_, 1C).

**IR (ATR):** ν[cm^-1^] = 3056, 3027, 2973, 2937, 2866, 1597, 1579, 1489, 1446, 1386, 1367, 1327, 1304, 1240, 1206, 1191, 1156, 1126, 1070, 1032, 968, 922, 897, 865, 783, 765, 736, 715, 702, 656, 647, 632.

**Mass spectrometry (MS-ESI, +)**: *calcd.* as [M–OMe] ^+^ C_23_H_21_BrN^+^: 390.0852, *found:* 390.0852.

**m.p.** [°C]: 131 (decomp.).

**4-bromo-2-(2,6-di(heptan-4-yl)phenyl)-3-methoxy-1,1-diphenylisoindoline (3e)**

The synthesis of methoxyisoindoline (**3e**) was performed by a *one-pot* reaction starting from the imine. Imine (**1e**) (6.62 g, 12.4 mmol, 1.00 equiv.) was dissolved in 250 mL dry diethylether and at –­­­­­­­­­78 °C *n*-BuLi (4.95 mL, 2.5 M in hexane, 0.79 g, 12.4 mmol, 1.00 equiv.) was added dropwise to the solution. The red-orange solution was stirred for 1 h at –­­­­­­­­­78 °C and then benzophenone (7.42 mL, 1.75 M in diethylether, 2.37 g, 13.0 mmol, 1.05 equiv.) was added dropwise at –­­­­­­­­­78 °C. The reaction was allowed to warm to rt and was stirred for 45 mins at rt. To the pale yellow to brown solution, trifluoromethanesulfonic anhydride (2.18 mL, 3.66 g,
13.0 mmol, 1.05 equiv.) was added carefully over 15 mins at –­­­­­­­­­78 °C. Again, the reaction was allowed to reach rt and stirred for 16 h. Next, the solvent was removed under reduced pressure and was replaced with dry methanol (100 mL). Then, sodium methoxide (9.89 mL, 5.0 M in MeOH, 2.67 g, 49.5 mmol, 4.00 equiv.) was added in one portion at rt and the reaction was stirred for 18 h. Finally, the product precipitated from the solution as pale-yellow solid which was filtered off and washed with methanol (5 x 10 mL). The product was dried *in vacuo* and could be obtained as pale-yellow powder. Yield: 74 % in 2 steps (5.92 g,
9.07 mmol).

**1H NMR** (400.33 MHz, CD_2_Cl_2_, 300 K): *δ*[ppm] = 7.51 (d, *J* = 7.8 Hz, 1H), 7.49–7.39 (m, 2H), 7.22–6.93 (m, 11H), 6.84 (dd, *J* = 7.7 Hz, *J* = 1.0 Hz, 1H), 6.78 (dd, *J* = 7.5 Hz, *J* = 1.9 Hz, 1H), 5.86 (s, 1H), 3.54 (s, 3H), 3.52–3.47 (m, 1H), 1.76–1.64 (m, 1H), 1.61–1.14 (m, 10H), 1.09–0.99 (m, 1H), 0.95–0.72 (m, 8H), 0.67–0.52 (m, 8H), –0.62 to –0.72 (m, 1H).

**13C{1H} NMR** (100.66 MHz, CD_2_Cl_2_, 300 K): *δ*[ppm] = 150.4 (s, Cq, 1C), 150.4 (s, Cq, 1C), 150.1 (s, Cq, 1C), 149.6 (s, Cq, 1C), 142.6 (s, Cq, 1C), 138.3 (s, Cq, 1C), 138.1 (s, Cq, 1C), 131.4 (s, CH, 1C), 130.2 (s, CH, 1C), 129.4 (bs, CH, 2C), 128.0 (s, CH, 2C), 127.6 (s, CH, 1C), 127.5 (bs, CH, 2C), 126.8 (s, CH, 1C), 126.6 (s, CH, 1C), 126.0 (s, CH, 1C), 126.0 (s, CH, 1C), 125.9 (s, CH, 1C), 119.5 (s, Cq, 1C), 103.7 (s, CH, 1C), 83.1 (s, Cq, 1C), 59.6 (s, CH_3_, 1C), 41.4 (s, CH_2_, 1C), 40.4 (s, CH, 1C), 40.0 (s, CH_2_, 1C), 38.9 (s, CH, 1C), 38.7 (s, CH_2_, 1C), 36.8 (s, CH_2_, 1C), 22.9 (s, CH_2_, 1C), 22.0 (s, CH_2_, 1C), 20.3 (s, CH_2_, 1C), 20.2 (s, CH_2_, 1C), 15.4 (s, CH_3_, 1C), 15.0 (s, CH_3_, 1C), 14.9 (s, CH_3_, 1C), 14.6 (s, CH_3_, 1C). *One tertiary carbon signal (2C) could not be detected.*

**IR (ATR):** ν[cm^-1^] = 2956, 2929, 2870, 1595, 1578, 1492, 1448, 1375, 1331, 1256, 1219, 1188, 1134, 1052, 994, 962, 930, 915, 898, 812, 783, 757, 731, 704, 638, 609.

**Mass spectrometry (MS-ESI, +)**: *calcd.* as [M–OMe]^+^ C_40_H_47_BrN^+^: 622.2866, *found:* 622.2877.

**m.p.** [°C]: 148 (decomp.).

**GP 6. Synthesis of 4-(diphenylphosphino)-3-methoxy-1,1-diphenylisoindoline (4)**

*N*-Aryl 4-bromo-3-methoxyisoindoline (**4**) (12.0 mmol, 1.00 equiv.) was dissolved in degassed THF (120 mL, 0.1 M) and the solution was cooled to –78 °C. At this temperature, *n*-BuLi (2.5 M in hexanes, 4.80 mL, 0.77 g, 1.00 equiv.) was added dropwise over 10 mins and the reaction mixture was stirred for 30 mins. Then, a solution of diphenyl phosphine chloride (1.0 M in THF, 12.0 mL, 2.65 g, 1.00 equiv.) was added dropwise and the reaction was allowed to slowly warm to rt for 16 h in a dry ice/acetone bath. The solvent was removed *in vacuo* to afford the crude phosphines which were then suspended in degassed diethylether (~20–30 mL) and filtrated under argon. The solid was washed with diethyl ether (1 x 20 mL, 4 x 5 mL) and dried *in vacuo* to afford the 3-methoxyisoindoline phosphine as colorless solids.

**2-(2,6-diisopropylphenyl)-4-(diphenylphosphino)-3-methoxy-1,1-diphenylisoindoline (4a)**

According to **GP6**. Alternatively, the light-brown crude product can be purified by washing with HPLC grade acetone (12 x 7 mL) under ambient atmosphere. The product was obtained as a colorless solid. Yield: 73 % (5.66 g, 8.76 mmol). Colorless crystals of **(4a)** were obtained by slow diffusion from chloroform.

**^1^H NMR** (400.33 MHz, CD_2_Cl_2_, 300 K): *δ*[ppm] = 7.47 (d, *J* = 6.7 Hz, 2H), 7.41–7.29 (m, 10H), 7.26 (t, *J* = 7.6 Hz, 1H), 7.22–6.94 (m, 10H), 6.92–6.84 (m, 2H), 6.77 (d, *J* = 6.7 Hz, 1H), 6.18 (s, 1H), 3.86 (sept, *J* = 6.7 Hz, 1H), 3.33 (s, 3H), 2.01 (sept, *J* = 6.7 Hz, 1H), 1.13 (d, *J* =
6.7 Hz, 3H), 0.95 (d, *J* = 6.7 Hz, 3H), 0.47 (d, *J* = 6.7 Hz, 3H), –0.07 (d, *J* = 6.7 Hz, 3H).

**^13^C{^1^H} NMR** (100.66 MHz, CD_2_Cl_2_, 300 K) *δ*[ppm] = 151.6 (s, Cq, 1C), 149.7 (s, Cq, 1C), 149.4 (s, Cq, 1C), 148.0 (d, *J* = 6.9 Hz, Cq, 1C), 143.8 (d, *J* = 28.7 Hz, Cq, 1C), 142.9 (s, Cq, 1C), 138.6 (s, Cq, 1C), 138.4 (d, *J* = 11.4 Hz, Cq, 1C), 137.4 (d, *J* = 11.1 Hz, Cq, 1C), 134.5 (d, *J* = 17.6 Hz, Cq, 1C), 134.2 (d, *J* = 20.2 Hz, CH, 2C), 134.0 (d, *J* = 19.6 Hz, CH, 2C), 133.4 (d, *J* = 2.9 Hz, CH, 1C), 131.2 (bs, CH, 2C), 129.7 (s, CH, 2C), 129.1 (s, CH, 1C), 128.9 (d, *J* = 6.8 Hz, CH, 2C), 128.8 (bs, CH, 2C), 128.7 (d, *J* = 6.9 Hz, CH, 2C), 127.7 (s, CH, 1C), 127.5 (s, CH, 2C), 127.5 (bs, CH, 2C), 127.4 (s, CH, 1C), 127.4 (s, CH, 1C), 126.6 (s, CH, 1C), 124.7 (s, CH, 1C), 124.5 (s, CH, 1C), 102.7 (d, *J* = 8.3 Hz, CH, 1C), 82.8 (s, Cq, 1C), 57.9 (d, *J* = 5.3 Hz, CH_3_, 1C), 29.2 (s, CH, 1C), 29.2 (s, CH, 1C), 26.2 (s, CH_3_, 1C), 25.0 (s, CH_3_, 1C), 23.6 (s, CH_3_, 1C), 23.5 (s, CH_3_, 1C).

**^31^P NMR** (121.49 MHz, CD_2_Cl_2_, 298 K): *δ* [ppm] = –15.4 (s).

**IR (ATR):** ν[cm^-1^] = 3054, 2961, 2866, 2820, 1584, 1493, 1476, 1459, 1434, 1383, 1361, 1325, 1253, 1174, 1068, 965, 899, 811, 798, 768, 743, 696, 633.

**Mass spectrometry (MS-ESI, +)**: *calcd.* as [M–OMe]^+^ C_44_H_41_NP^+^: 614.2971, *found:* 614.2981.

**m.p.** [°C]: 195 (decomp.).

**2-(2,6-diethylphenyl)-4-(diphenylphosphino)-3-methoxy-1,1-diphenylisoindoline (4b)**

According to **GP6**. The product was obtained as a colorless solid. Yield: 88 % (6.55 g, 10.6 mmol). The filtrate was cooled to –40 °C to give colorless crystals of **(4b)** suitable for X-Ray crystallography analysis.

**1H NMR** (600.24 MHz, CD_2_Cl_2_, 300 K): *δ*[ppm] = 7.62 (d, *J* = 7.6 Hz, 2H), 7.41–7.31 (m, 10H), 7.26–7.20 (m, 2H), 7.15 (tt, *J* = 7.3 Hz, *J* = 1.3 Hz, 1H), 7.10 (tt, *J* = 7.3 Hz, *J* = 1.3 Hz, 1H), 7.00–6.91 (m, 6H), 6.86 (bd, *J* = 7.5 Hz, 2H), 6.65 (dd, *J* = 6.7 Hz, *J* = 2.6 Hz, 1H), 5.78 (s, 1H), 3.28 (s, 3H), 2.96–2.86 (m, 1H), 2.66–2.54 (m, 1H), 1.65–1.56 (m, 1H), 1.34–1.26 (m, 1H), 0.89 (t, *J* = 7.5 Hz, 3H), 0.58 (t, *J* = 7.5 Hz, 3H).

**13C{1H} NMR** (150.93 MHz, CD_2_Cl_2_, 300 K): *δ*[ppm] = 149.4 (s, Cq, 1C), 148.2 (d, *J* = 5.9 Hz, Cq, 1C), 146.4 (s, Cq, 1C), 144.9 (s, Cq, 1C), 144.2 (s, Cq, 1C), 142.9 (d, *J* = 26.3 Hz, Cq, 1C), 138.5 (s, Cq, 1C), 137.8 (d, *J* = 11.7 Hz, Cq, 1C), 137.1 (d, *J* = 11.6 Hz, Cq, 1C), 135.2 (d, *J* = 18.3 Hz, Cq, 1C), 134.5 (d, *J* = 20.5 Hz, CH, 2C), 134.3 (d, *J* = 20.1 Hz, CH, 2C), 132.7 (s, CH, 1C), 131.1 (bs, CH, 2C), 129.4 (bs, CH, 2C), 129.2 (s, CH, 1C), 129.0 (s, CH, 1C), 129.0 (s, CH, 1C), 128.9 (d, *J* = 7.4 Hz, CH, 2C), 128.7 (d, *J* = 7.1 Hz, CH, 2C), 127.7 (bs, CH, 2C), 127.3 (s, CH, 1C), 126.8 (bs, CH, 2C), 126.7 (s, CH, 1C), 126.6 (s, CH, 1C), 126.6 (s, CH, 1C), 126.3 (s, CH, 1C), 126.0 (s, CH, 1C), 102.5 (d, *J* = 6.8 Hz, CH, 1C), 83.2 (s, Cq, 1C), 57.3 (d, *J* = 2.7 Hz, CH_3_, 1C), 26.6 (s, CH_2_, 1C), 24.8 (s, CH_2_, 1C), 14.7 (s, CH_3_, 1C), 14.1 (s, CH_3_, 1C).

**31P{1H} NMR** (242.98 MHz, CD_2_Cl_2_, 300 K): *δ*[ppm] = –13.9 (s).

**IR (ATR):** ν[cm^-1^] = 3059, 2971, 2876, 2820, 1584, 1435, 1334, 1254, 1176, 1103, 1064, 993, 964, 920, 897, 848, 818, 778, 745, 696, 637.

**Mass spectrometry (MS-ESI, +)**: *calcd.* as [M–OMe]^+^ C_42_H_37_NP^+^: 586.2658, *found:* 586.2668.

**m.p.** [°C]: 193 (decomp.).

**2-(2,6-diethyl-4-methylphenyl)-4-(diphenylphosphino)-3-methoxy-1,1-diphenylisoindoline (4c)**

According to **GP6**. The product was obtained as a colorless solid. Colorless Yield: 81 %
(6.14 g, 9.72 mmol). single crystals suitable for X-Ray crystallography were obtained from the filtrate at –40 °C.

**1H NMR** (600.24 MHz, CD_2_Cl_2_, 300 K): *δ*[ppm] = 7.61 (bd, *J* = 7.7 Hz, 2H), 7.40–7.33 (m, 10H), 7.25–7.20 (m, 3H), 7.16–7.09 (m, 3H), 7.00 (t, *J* = 7.8 Hz, 2H), 6.95–6.91 (m, 2H), 6.87 (bd,
*J* = 7.7 Hz, 2H), 6.80 (d, *J* = 1.8 Hz, 1H), 6.47 (d, *J* = 1.8 Hz, 1H), 5.75 (s, 1H), 3.28 (s, 3H), 2.88–2.79 (m, 1H), 2.64–2.54 (m, 1H), 2.17 (s, 3H), 1.60–1.52 (m, 1H), 1.29–1.20 (m, 1H), 0.86 (t, *J* = 7.6 Hz, 3H), 0.56 (t, *J* = 7.4 Hz, 3H).

**13C{1H} NMR** (150.93 MHz, CD_2_Cl_2_, 300 K): *δ*[ppm] = 149.5 (s, Cq, 1C), 148.2 (d, *J* = 5.9 Hz, Cq, 1C), 145.9 (s, Cq, 1C), 143.7 (s, Cq, 1C), 142.9 (d, *J* = 25.9 Hz, Cq, 1C), 142.2 (s, Cq, 1C), 138.6 (s, Cq, 1C), 137.8 (d, *J* = 11.6 Hz, Cq, 1C), 137.1 (d, *J* = 11.6 Hz, Cq, 1C), 135.9 (s, Cq, 1C), 135.1 (d, *J* = 18.4 Hz, Cq, 1C), 134.5 (d, *J* = 20.5 Hz, CH, 2C), 134.3 (d, *J* =
20.1 Hz, CH, 2C), 132.7 (s, CH, 1C), 131.1 (bs, CH, 2C), 129.4 (bs, CH, 2C), 129.2 (s, CH, 1C), 128.9 (s, CH, 2C), 128.9 (d, *J* = 7.2 Hz, CH, 2C), 128.7 (d, *J* = 7.3 Hz, CH, 2C), 127.7 (bs, CH, 2C), 127.2 (s, CH, 1C), 127.0 (s, CH, 1C), 126.9 (s, CH, 1C), 126.8 (s, CH, 3C), 126.5 (s, CH, 1C), 102.6 (d, *J* = 6.6 Hz, CH, 1C), 83.0 (s, Cq, 1C), 57.3 (d, *J* = 2.6 Hz, CH_3_, 1C), 26.5 (s, CH_2_, 1C), 24.7 (s, CH_2_, 1C), 21.1 (s, CH_3_, 1C), 14.7 (s, CH_3_, 1C), 14.2 (s, CH_3_, 1C).

**31P{1H} NMR** (242.98 MHz, CD_2_Cl_2_, 300 K): *δ*[ppm] = –13.8 (s).

**IR (ATR):** ν[cm^-1^] = 3050, 2973, 2929, 2875, 2825, 1957, 1896, 1818, 1584, 1492, 1459, 1433, 1371, 1334, 1304, 1235, 1187, 1157, 1107, 1065, 999, 965, 931, 896, 862, 795, 766, 744, 716, 696, 634.

**Mass spectrometry (MS-ESI, +)**: *calcd.* as [M–OMe]^+^ C_43_H_39_NP^+^: 600.2815, *found:* 600.2816.

**m.p.** [°C]: 199 (decomp.).

**2-(2,6-di(pentan-3-yl)phenyl)-4-(diphenylphosphino)-3-methoxy-1,1-diphenyl-isoindoline (4d)**

According to **GP6**. The product was obtained as a colorless solid. Yield: 91 % (7.64 g,
10.9 mmol). Colorles single crystals of (**4d**) suitable for X-Ray crystallography were obtained from the diethyl ether filtrate at –40 °C.

**1H NMR** (600.24 MHz, CD_2_Cl_2_, 300 K): *δ*[ppm] = 7.47 (bs, 2H), 7.40–7.19 (m, 12H), 7.19–7.08 (m, 5H), 7.07–6.96 (m, 3H), 6.94 (dd, *J* = 7.7 Hz, *J* = 1.7 Hz, 1H), 6.89 (d, *J* = 7.7 Hz, 1H), 6.86–6.56 (m, 2H), 6.24 (s, 1H), 3.42–3.37 (m, 1H), 3.32 (s, 3H), 1.80–1.72 (m, 1H), 1.68–1.61 (m, 1H), 1.57–1.44 (m, 2H), 1.44–1.35 (m, 1H), 1.25–1.15 (m, 1H), 1.14–1.1.06 (m, 1H), 0.90 (t, *J* = 7.6 Hz, 3H), 0.82 (t, *J* = 7.6 Hz, 3H), 0.79–0.71 (m, 1H), 0.47 (t, *J* = 7.5 Hz, 3H), 0.38 (t, *J* = 7.5 Hz, 3H), –0.72 to –0.82 (m, 1H).

**13C{1H} NMR** (150.93 MHz, CD_2_Cl_2_, 300 K): *δ*[ppm] = 150.7 (s, Cq, 1C), 149.3 (s, Cq, 1C), 148.6 (s, Cq, 1C), 148.1 (d, *J* = 7.2 Hz, Cq, 1C), 143.8 (s, Cq, 1C), 143.8 (d, *J* = 29.5 Hz, Cq, 1C), 138.6 (s, Cq, 1C), 138.6 (d, *J* = 11.1 Hz, Cq, 1C), 137.4 (d, *J* = 11.0 Hz, Cq, 1C), 134.1 (d, *J* = 8.6 Hz, Cq, 1C), 134.0 (d, *J* = 9.7 Hz, CH, 2C), 133.9 (d, *J* = 9.7 Hz, CH, 2C), 133.7 (d, *J* = 3.0 Hz, CH, 1C), 129.3 (bs, CH, 2C), 128.9 (d, *J* = 6.4 Hz, CH, 2C), 128.8 (s, CH, 2C), 128.8 (s, CH, 1C), 128.6 (d, *J* = 7.0 Hz, CH, 2C), 127.8 (s, CH, 2C), 127.8 (bs, CH, 2C) 127.6 (s, CH, 1C), 127.4 (s, CH, 1C), 126.5 (s, CH, 1C), 126.2 (s, CH, 1C), 126.0 (s, CH, 1C), 125.9 (s, CH, 1C), 103.3 (d, *J* = 7.4 Hz, CH, 1C), 82.8 (s, Cq, 1C), 58.0 (d, *J* = 7.5 Hz, CH_3_, 1C), 41.8 (s, CH, 1C), 40.2 (s, CH, 1C), 29.2 (s, CH_2_, 1C), 28.8 (s, CH_2_, 1C), 26.7 (s, CH_2_, 1C), 25.2 (s, CH_2_, 1C), 13.6 (s, CH_3_, 1C), 12.8 (d, *J* = 3.8 Hz, CH_3_, 1C), 11.0 (s, CH_3_, 1C), 10.5 (s, CH_3_, 1C). *2 tertiary carbon signals (1C each) could not be detected.*

**31P{1H} NMR** (242.98 MHz, CD_2_Cl_2_, 300 K): *δ*[ppm] = –15.5 (s).

**IR (ATR):** ν[cm^-1^] = 3057, 2961, 2932, 2871, 2859, 1958, 1737, 1585, 1434, 1376, 1322, 1240, 1186, 1109, 1093, 1054, 965, 929, 896, 843, 819, 794, 778, 757, 742, 717, 694, 635, 618.

**Mass spectrometry (MS-ESI, +)**: *calcd.* as [M–OMe]^+^ C_48_H_49_NP^+^: 670.3597, *found:* 670.3599.

**m.p.** [°C]: 187 (decomp.).

**2-(2,6-di(heptan-4-yl)phenyl)-4-(diphenylphosphino)-3-methoxy-1,1-diphenyl-isoindoline (4e)**

According to **GP6**. The product was obtained as a colorless solid. Yield: 70 % (6.37 g,
8.40 mmol). Colorless single crystals of (**4e**) suitable for X-Ray crystallography were obtained from the diethyl ether filtrate at –40 °C.

**1H NMR** (600.24 MHz, CD_2_Cl_2_, 300 K): *δ*[ppm] = 7.61–7.21 (m, 14H), 7.20–6.90 (m, 5H), 6.88 (d, *J* = 7.6 Hz, 1H), 6.85 –6.47 (m, 2H), 6.20 (s, 1H), 3.54–3.48 (m, 1H), 3.35 (s, 3H), 1.77–1.66 (m, 2H), 1.46–1.37 (m, 2H), 1.36–1.23 (m, 5H), 1.19–1.11 (m, 2H), 1.01–0.87 (m, 2H), 0.85 (t, *J* = 7.3 Hz, 3H), 0.79 (t, *J* = 7.0 Hz, 3H), 0.77–0.68 (m, 1H), 0.65–0.53 (m, 8H), –0.68 to –0.80 (m, 1H).

**13C{1H} NMR** (150.93 MHz, CD_2_Cl_2_, 300 K): *δ*[ppm] = 150.7 (s, Cq, 1C), 150.1 (s, Cq, 1C), 149.2 (s, Cq, 1C), 148.2 (d, *J* = 7.2 Hz, Cq, 1C), 143.9 (d, *J* = 29.5 Hz, Cq, 1C), 143.2 (s, Cq, 1C), 138.7 (d, *J* = 11.3 Hz, Cq, 1C), 138.6 (s, Cq, 1C), 137.6 (d, *J* = 11.3 Hz, Cq, 1C), 134.2 (d, *J* = 17.9 Hz, Cq, 1C), 134.0 (d, *J* = 20.0 Hz, CH, 2C), 133.8 (d, *J* = 19.2 Hz, CH, 2C), 133.7 (s, CH, 1C), 129.3 (bs, CH, 2C), 128.9 (d, *J* = 6.3 Hz, CH, 2C), 128.8 (s, CH, 1C), 128.7 (s, CH, 1C), 128.7 (s, CH, 1C), 128.6 (d, *J* = 7.0 Hz, CH, 2C), 127.8 (s, CH, 2C), 127.6 (s, CH, 1C), 127.4 (s, CH, 1C), 126.5 (s, CH, 1C), 126.2 (s, CH, 1C), 126.1 (s, CH, 1C), 125.8 (s, CH, 1C), 103.2 (d, *J* = 7.0 Hz, CH, 1C), 82.8 (s, Cq, 1C), 58.1 (d, *J* = 7.7 Hz, CH_3_, 1C), 40.5 (s, CH_2_, 1C), 39.8 (s, CH, 1C), 39.6 (s, CH_2_, 1C), 38.7 (s, CH, 1C), 38.0 (s, CH_2_, 1C), 36.7 (s, CH_2_, 1C), 22.0 (d, *J* = 3.8 Hz, CH_2_, 1C), 22.0 (s, CH_2_, 1C), 20.1 (s, CH_2_, 1C), 20.0 (s, CH_2_, 1C), 15.3 (s, CH_3_, 1C), 15.0 (s, CH_3_, 1C), 14.9 (s, CH_3_, 1C), 14.5 (s, CH_3_, 1C). *2 tertiary carbon signals (2C each) could not be detected.*

**31P{1H} NMR** (242.93 MHz, CD_2_Cl_2_, 300 K): *δ*[ppm]= –15.5 (s).

**IR (ATR):** ν[cm^-1^] = 3054, 2955, 2927, 2869, 1959, 1584, 1434, 1376, 1326, 1255, 1097, 1056, 995, 966, 929, 897, 850, 801, 776, 741, 696, 637, 614.

**Mass spectrometry (MS-EI)**: *calcd.* as [M–OMe]^+^ C_52_H_57_NP^+^: 726.4223, *found:* 726.4225.

**m.p.** [°C]: 210 (decomp.).

**2-(2,6-diisopropylphenyl)-4-(diphenylphosphino)-3,5,7-trimethoxy-1,1-diphenylisoindoline (4f)**

According to **GP6**. The product was obtained as an off-white solid. Yield: 66 % (5.59 g,
7.92 mmol). Again, colorless single crystals of (**4f**) suitable for X-Ray crystallography were obtained from the filtrate confirming the connectivity.

**^1^H NMR** (600.24 MHz, CD_2_Cl_2_, 300 K): *δ* [ppm] = 7.68–7.45 (m, 3H), 7.42–7.22 (m, 9H), 7.19–6.80 (m, 9H), 6.78 (dd, *J* = 7.7 Hz, *J* = 1.5 Hz, 1H), 6.54 (bs, 1H), 6.36 (s, 1H), 6.32 (s, 1H), 3.81 (sept, *J* = 6.8 Hz, 1H), 3.45 (s, 3H), 3.41 (s, 3H), 3.39 (s, 3H), 2.17 (sept, *J* = 6.7 Hz, 1H), 1.17 (d, *J* = 6.7 Hz, 3H), 1.11 (d, *J* = 6.9 Hz, 3H), 0.45 (d, *J* = 6.8 Hz, 3H), 0.00 (d, *J* = 6.7 Hz, 3H).

**13C{1H} NMR** (150.93 MHz, CD_2_Cl_2_, 300 K): *δ*[ppm] = 164.4 (d, *J* = 4.6 Hz, Cq, 1C), 157.4 (d, *J* = 1.5 Hz, Cq, 1C), 151.6 (s, Cq, 1C), 149.3 (s, Cq, 1C), 148.7 (d, *J* = 34.2 Hz, Cq, 1C), 147.3 (s, Cq, 1C), 143.2 (s, Cq, 1C), 139.6 (d, *J* = 10.1 Hz, Cq, 1C), 137.1 (d, *J* = 10.3 Hz, Cq, 1C), 137.0 (s, Cq, 1C), 133.3 (d, *J* = 19.2 Hz, CH, 2C), 132.8 (d, *J* = 20.1 Hz, CH, 2C), 130.8 (bs, CH, 2C), 128.6 (d, *J* = 8.9 Hz, Cq, 1C), 128.3 (d, *J* = 6.5 Hz, CH, 2C), 128.0 (s, CH, 1C), 127.9 (d, *J* = 6.4 Hz, CH, 2C), 127.6 (s, CH, 1C), 127.2 (s, CH, 1C), 126.9 (s, CH, 1C), 126.7 (bs, CH, 2C), 126.1 (s, CH, 1C), 124.6 (s, CH, 1C), 124.5 (s, CH, 1C), 110.7 (d, *J* = 22.0 Hz, Cq, 1C), 103.4 (d, *J* = 14.5 Hz, CH, 1C), 97.8 (s, CH, 1C), 82.1 (s, Cq, 1C), 57.8 (d, *J* = 6.5 Hz, CH_3_, 1C), 55.6 (s, CH_3_, 1C), 55.5 (s, CH_3_, 1C), 29.2 (s, CH, 1C), 29.1 (s, CH, 1C), 26.5 (s, CH_3_, 1C), 25.0 (s, CH_3_, 1C), 23.9 (s, CH_3_, 1C), 23.4 (s, CH_3_, 1C). *Two tertiary signals (2C each) could not be detected.*

**31P{1H} NMR** (242.98 MHz, CD_2_Cl_2_, 300 K): *δ*[ppm] = –14.2 (s).

**IR (ATR):** ν[cm^-1^] = 3056, 2961, 2936, 2866, 1595, 1460, 1449, 1434, 1383, 1336, 1211, 1061, 995, 896, 814, 778, 740, 693, 628.

**Mass spectrometry (MS-ESI, +)**: *calcd.* for [M–OMe]^+^ C_46_H_45_NPO_2_^+^: 674.3182, *found*: 674.3185.

**m.p.** [°C]: 169 (decomp.).

**6-(*tert*-butyl)-2-(2,6-diisopropylphenyl)-4-(diphenylphosphino)-3-methoxy-1,1-diphenylisoindoline (4g)**

According to **GP6**. The product was obtained as a colorless solid. Yield: 78 % (6.57 g, 9.36 mmol).

**^1^H NMR** (600.24 MHz, CD_2_Cl_2_, 300 K): *δ* [ppm] = 7.50–7.44 (bs, 2H), 7.40–7.30 (m, 10H), 7.19–7.16 (m, 2H), 7.15–7.07 (m, 7H), 7.02–6.77 (m, 3H), 6.75 (dd, *J* = 7.5 Hz, *J* = 1.9 Hz, 1H), 6.09 (s, 1H), 3.88 (sept, *J* = 6.9 Hz, 1H), 3.33 (s, 3H), 1.97 (sept, *J* = 6.7 Hz, 1H), 1.19 (d, *J* = 6.9 Hz, 3H), 1.07 (s, 9H), 0.88 (d, *J* = 6.7 Hz, 3H), 0.45 (d, *J* = 6.9 Hz, 3H), –0.09 (d, *J* = 6.7 Hz, 3H).

**13C{1H} NMR** (150.93 MHz, CD_2_Cl_2_, 300 K): *δ*[ppm] = 151.7 (s, Cq, 1C), 151.6 (s, Cq, 1C), 149.6 (s, Cq, 1C), 149.5 (s, Cq, 1C), 147.7 (d, *J* = 7.1 Hz, Cq, 1C), 142.9 (s, Cq, 1C), 140.9 (d, *J* = 28.4 Hz, Cq, 1C), 138.8 (s, Cq, 1C), 138.5 (d, *J* = 11.6 Hz, Cq, 1C), 137.7 (d, *J* =
11.4 Hz, Cq, 1C), 134.2 (d, *J* = 20.0 Hz, CH, 2C), 134.0 (d, *J* = 19.9 Hz, CH, 2C), 133.2 (d,
*J* = 17.0 Hz, Cq, 1C), 131.1 (bs, CH, 2C), 130.9 (d, *J* = 2.8 Hz, CH, 1C), 129.7 (bs, CH, 2C) 129.0 (s, CH, 1C), 128.8 (d, *J* = 6.6 Hz, CH, 2C), 128.8 (s, CH, 1C), 128.6 (d, *J* = 7.0 Hz, CH, 2C), 127.5 (s, CH, 2C), 127.4 (bs, CH, 2C), 127.3 (s, CH, 1C), 127.2 (s, CH, 1C), 126.4 (s, CH, 1C), 124.6 (s, CH, 1C), 124.5 (s, CH, 1C), 124.3 (s, CH, 1C), 102.5 (d, *J* = 7.5 Hz CH, 1C), 82.9 (s, Cq, 1C), 57.9 (d, *J* = 4.9 Hz, CH_3_, 1C), 35.2 (s, Cq, 1C), 31.3 (s, CH_3_, 3C), 29.1 (s, CH, 1C), 29.0 (s, CH, 1C), 26.2 (s, CH_3_, 1C), 25.0 (s, CH_3_, 1C), 23.5 (s, CH_3_, 1C), 23.4 (s, CH_3_, 1C).

**31P{1H} NMR** (242.98 MHz, CDCl_3_, 300 K): *δ*[ppm] = –14.2 (s).

**IR (ATR):** ν[cm^-1^] = 3055, 2960, 2866, 1584, 1434, 1382, 1364, 1330, 1256, 1156, 1117, 1058, 977, 929, 907, 882, 813, 771, 741, 693, 647.

**Mass spectrometry (MS-ESI, +)**: *calcd.* for [M–OMe]^+^ C_48_H_49_NP^+^: 670.3597, *found*: 670.3598.

**m.p.** [°C]: 212 (decomp.).

**GP 7: Synthesis of phosphine isoindolium tetrafluoroborate salts**

Methoxyisoindoline phosphine **4** (10.0 mmol, 1.00 equiv.) was suspended in anhydrous degassed diethyl ether (125 mL) and the suspension was cooled to –78 °C. Then, tetrafluoroboric acid diethyl ether complex (1.36 mL, 1.62 g, 10.0 mmol, 1.00 equiv.) was added dropwise at –78 °C and the mixture was allowed to warm to rt. After stirring for 1 h at rt, the supernatant was removed and the solid residue was washed with anhydrous degassed diethyl ether (5 x 40 mL) using a syringe. The product was extracted with degassed acetonitrile and filtrated by syringe filtration. The resulting product was dried *in vacuo*to give 4-(diphenylphosphino)-1,1-diphenyl-1*H*-isoindol-2-ium tetrafluoroborates as yellow to orange solids.

**2-(2,6-diisopropylphenyl)-4-(diphenylphosphino)-1,1-diphenyl-1*H*-isoindol-2-ium tetrafluoroborate (5a)**

According to **GP7**. The product was obtained as an orange solid. Single crystals were obtained from a saturated benzene solution. Yield: 98 % (6.88 g, 9.80 mmol).

**^1^H NMR** (600.24 MHz, CD_2_Cl_2_, 300 K): *δ*[ppm] = 9.23 (s, 1H), 7.93 (t, *J* = 7.7 Hz, 1H), 7.53–7.45 (m, 13H), 7.45–7.42 (m, 1H), 7.38–7.32 (m, 5H), 7.14 (d, *J* = 7.8 Hz, 2H), 7.00–6.95 (m, 4H), 1.89 (sept, *J* = 6.7 Hz, 2H), 0.73 (d, *J* = 6.9 Hz, 6H), 0.23 (d, *J* = 6.6 Hz, 6H).

**^13^C NMR** (150.93 MHz, CD_2_Cl_2_, 300 K) *δ*[ppm] = 173.0 (d, *J* = 12.4 Hz, CH, 1C), 157.6 (s, Cq, 1C), 146.5 (s, Cq, 2C), 145.8 (d, *J* = 25.3 Hz, Cq, 1C), 138.9 (s, CH, 1C), 135.5 (s, CH, 1C), 134.8 (d, *J* = 20.7 Hz, CH, 4C), 133.8 (d, *J* = 8.0 Hz, Cq, 2C), 133.1 (s, Cq, 1C), 132.9 (s, CH, 1C), 131.9 (d, *J* = 21.5 Hz, Cq, 1C), 131.3 (s, CH, 4C), 131.0 (s, CH, 4C), 130.6 (s, Cq, 2C), 130.4 (s, CH, 4C), 129.9 (d, *J* = 7.0 Hz, CH, 4C), 129.7 (s, CH, 4C), 127.2 (s, CH, 1C), 125.8 (s, CH, 2C), 97.0 (s, Cq, 1C), 30.7 (s, CH, 2C), 26.0 (s, CH_3_, 2C), 21.5 (s, CH_3_, 2C).

**^31^P NMR** (242.98 MHz, CD_2_Cl_2_, 300 K): *δ* [ppm] = –13.1 (s).

**IR (ATR):** ν[cm^-1^] = 3065, 2969, 2931, 2872, 1597, 1544, 1436, 1360, 1325, 1297, 1256, 1223, 1167, 1059, 1028, 1000, 934, 805, 751, 700, 657, 613.

**Mass spectrometry (MS-ESI, +)**: *calcd.* for [M–BF_4_]^+^ C_44_H_41_NP^+^: 614.2971, *found:* 614.2972.

**m.p.** [°C]: 251 (decomp.).

**2-(2,6-diethylphenyl)-4-(diphenylphosphino)-1,1-diphenyl-1*H*-isoindol-2-ium tetrafluoroborate (5b)**

According to **GP7**. The product was obtained as an orange solid. Yield: 98 % (6.60 g,
9.80 mmol).

**1H NMR** (600.24 MHz, CD_2_Cl_2_, 300 K): *δ*[ppm] = 9.17 (s, 1H), 7.92 (t, *J* = 7.7 Hz, 1H), 7.52–7.46 (m, 12H), 7.43–7.38 (m, 2H), 7.35–7.31 (m, 5H), 7.08–7.05 (m, 2H), 7.04–7.00 (m, 4H), 1.62–1.54 (m, 2H), 1.39–1.32 (m, 2H), 0.52 (t, *J* = 7.5 Hz, 6H).

**13C{1H} NMR** (150.93 MHz, CD_2_Cl_2_, 300 K): *δ*[ppm] = 173.4 (d, *J* = 8.8 Hz, CH, 1C), 157.8 (s, Cq, 1C), 146.1 (d, *J* = 24.9 Hz, Cq, 1C), 141.7 (s, Cq, 2C), 138.8 (s, CH, 1C), 135.1 (s, CH, 1C), 134.8 (d, *J* = 19.9 Hz, CH, 4C), 134.6 (s, Cq, 1C), 133.8 (bd, *J* = 6.4 Hz, Cq, 2C), 132.4 (s, CH, 1C), 131.9 (d, *J* = 20.3 Hz, Cq, 1C), 131.4 (s, CH, 2C), 131.0 (s, CH, 2C), 130.4 (s, CH, 4C), 130.3 (s, Cq, 2C), 129.9 (bs, CH, 4C), 129.5 (s, CH, 4C), 127.8 (s, CH, 2C), 126.9 (s, CH, 1C), 96.8 (s, Cq, 1C), 25.1 (s, CH_2_, 2C), 14.1 (s, CH_3_, 2C).

**^31^P NMR** (242.98 MHz, CD_2_Cl_2_, 300 K): *δ* [ppm] = –11.8 (s).

**IR (ATR):** ν[cm^-1^] = 3070, 2977, 2934, 2878, 1593, 1534, 1435, 1362, 1216, 1051, 814, 776, 744, 698, 671, 653.

**Mass spectrometry (MS-ESI, +)**: *calcd.* for [M–BF_4_]^+^ C_42_H_37_NP^+^: 586.2658, *found*: 586.2663.

**m.p.** [°C]: 251 (decomp.).

**2-(2,6-diethyl-4-methylphenyl)-4-(diphenylphosphino)-1,1-diphenyl-1*H*-isoindol-2-ium tetrafluoroborate (5c)**

According to **GP7**. The product was obtained as an orange solid. Yield: 95 % (6.53 g,
9.50 mmol).

**1H NMR** (600.24 MHz, CD_2_Cl_2_, 300 K): *δ*[ppm] = 9.12 (s, 1H), 7.91 (t, *J* = 7.8 Hz, 1H), 7.53–7.45 (m, 12H), 7.38 (d, *J* = 7.6 Hz, 1H), 7.36–7.31 (m, 5H), 7.05–7.00 (m, 4H), 6.85 (s, 2H), 2.31 (s, 3H), 1.57–1.49 (m, 2H), 1.35–1.28 (m, 2H), 0.51 (t, *J* = 7.5 Hz, 6H).

**13C{1H} NMR** (150.93 MHz, CD_2_Cl_2_, 300 K): *δ*[ppm] = 173.3 (d, *J* = 8.5 Hz, CH, 1C), 157.7 (s, Cq, 1C), 145.8 (d, *J* = 25.0 Hz, Cq, 1C), 143.0 (s, Cq, 1C), 141.3 (s, Cq, 2C), 138.6 (s, CH, 1C), 135.0 (s, CH, 1C), 134.8 (d, *J* = 20.0 Hz, CH, 4C), 133.8 (bs, Cq, 2C), 132.2 (s, Cq, 1C), 131.9 (d, *J* = 19.9 Hz, Cq, 1C) 131.3 (s, CH, 2C), 131.0 (s, CH, 2C), 130.4 (s, Cq, 2C), 130.4 (s, CH, 4C), 129.9 (bs, CH, 4C), 129.4 (s, CH, 4C), 128.5 (s, CH, 2C), 126.9 (s, CH, 1C), 96.6 (s, Cq, 1C), 24.9 (s, CH_2_, 2C), 21.3 (s, CH_3_, 1C), 14.2 (s, CH_3_, 2C).

**^31^P NMR** (242.98 MHz, CD_2_Cl_2_, 300 K): *δ* [ppm] = –11.8 (s).

**IR (ATR):** ν[cm^-1^] = 3058, 2972, 2935, 2878, 1604, 1582, 1536, 1451, 1436, 1360, 1217, 1190, 1053, 861, 801, 747, 698, 657.

**Mass spectrometry (MS-ESI, +)**: *calcd.* for [M–BF_4_]^+^ C_43_H_39_NP^+^: 600.2815, *found*: 600.2818.

**m.p.** [°C]: 170 (decomp.).

**2-(2,6-di(pentan-3-yl)phenyl)-4-(diphenylphosphino)-1,1-diphenyl-1*H*-isoindol-2-ium tetrafluoroborate (5d)**

According to **GP7**. The product was obtained as an orange solid. Yield: 98 % (7.43 g,
9.80 mmol).

**1H NMR** (600.24 MHz, CD_2_Cl_2_, 300 K): *δ*[ppm] = 9.36 (s, 1H), 8.05 (t, *J* = 7.8 Hz, 1H), 7.72–7.65 (m, 6H), 7.64–7.60 (m, 7H), 7.53–7.48 (m, 5H), 7.43 (d, *J* = 7.8 Hz, 1H), 7.21 (d,
*J* = 7.8 Hz, 2H), 7.10–7.06 (m, 4H), 1.84–1.78 (m, 2H), 1.48–1.38 (m, 2H), 1.30–1.19 (m, 4H), 0.61 (t, *J* = 7.4 Hz, 6H), 0.45 (t, *J* = 7.4 Hz, 6H), 0.32–0.24 (m, 2H).

**13C{1H} NMR** (150.93 MHz, CD_2_Cl_2_, 300 K): *δ*[ppm] = 173.4 (d, *J* = 11.0 Hz, CH, 1C), 158.0 (s, Cq, 1C), 146.0 (d, *J* = 25.3 Hz, Cq, 1C), 144.4 (s, Cq, 2C), 139.1 (s, CH, 1C), 135.7 (s, Cq, 1C), 135.0 (s, CH, 1C), 134.9 (d, *J* = 20.8 Hz, CH, 4C), 133.6 (d, *J* = 7.4 Hz, Cq, 1C), 132.4 (s, CH, 1C), 131.2 (s, CH, 2C), 131.2 (s, CH, 2C), 131.0 (d, *J* = 21.5 Hz, Cq, 1C), 130.9 (s, Cq, 2C), 130.2 (s, CH, 4C), 130.0 (d, *J* = 6.4 Hz, CH, 4C), 129.9 (s, CH, 4C), 127.1 (s, CH, 1C), 126.9 (s, CH, 2C), 97.2 (s, Cq, 1C), 43.3 (s, CH, 2C), 28.9 (s, CH_2_, 2C), 26.9 (s, CH_2_, 2C), 12.2 (s, CH_3_, 2C), 11.2 (s, CH_3_, 2C).

**^31^P NMR** (242.98 MHz, CD_2_Cl_2_, 300 K): *δ* [ppm] = –13.0 (s).

**IR (ATR):** ν[cm^-1^] = 3071, 2964, 2935, 2875, 1634, 1594, 1581, 1529, 1461, 1434, 1360, 1218, 1162, 1051, 1001, 974, 954, 806, 768, 740, 703, 676, 658.

**Mass spectrometry (MS-ESI, +)**: *calcd.* for [M–BF_4_]^+^ C_48_H_49_NP^+^: 670.3597, *found*: 670.3599.

**m.p.** [°C]: 120 (decomp.).

**2-(2,6-di(heptan-4-yl)phenyl)-4-(diphenylphosphino)-1,1-diphenyl-1*H*-isoindol-2-ium tetrafluoroborate (5e)**

According to **GP7**. The product was obtained as an orange solid. Yield: 90 % (7.32 g,
9.00 mmol).

**1H NMR** (600.24 MHz, CD_2_Cl_2_, 300 K): *δ*[ppm] = 9.29 (s, 1H), 7.91 (t, *J* = 7.7 Hz, 1H), 7.56–7.50 (m, 6H), 7.50–7.45 (m, 3H), 7.44–7.39 (m, 4H), 7.37–7.31 (m, 5H), 7.28 (d, *J* = 7.9 Hz, 1H), 7.09 (d, *J* = 7.9 Hz, 2H), 6.92 (d, *J* = 8.1 Hz, 4H), 1.80–1.72 (m, 2H), 1.31–1.24 (m, 2H), 1.09–0.99 (m, 4H), 0.87–0.80 (m, 2H), 0.78–0.69 (m, 10H), 0.59 (t, *J* = 7.2 Hz, 6H), 0.41–0.33 (m, 2H), 0.05 to –0.02 (m, 2H).

**13C{1H} NMR** (150.93 MHz, CD_2_Cl_2_, 300 K): *δ*[ppm] = 173.2 (d, *J* = 13.3 Hz, CH, 1C), 157.9 (d, *J* = 3.5 Hz, Cq, 1C), 145.3 (d, *J* = 25.0 Hz, Cq, 1C), 145.0 (s, Cq, 2C), 139.2 (s, CH, 1C), 135.4 (s, CH, 1C), 135.0 (s, Cq, 1C), 134.6 (d, *J* = 20.8 Hz, CH, 4C), 133.6 (d, *J* = 8.4 Hz, Cq, 2C), 132.5 (s, CH, 1C), 131.4 (d, *J* = 23.0 Hz, Cq, 1C), 131.1 (d, *J* = 16.6 Hz, CH, 4C), 130.8 (s, Cq, 2C), 130.3 (s, CH, 4C), 130.0 (d, *J* = 7.6 Hz, CH, 4C), 129.9 (s, CH, 4C), 127.3 (s, CH, 1C), 126.9 (s, CH, 2C), 97.2 (s, Cq, 1C), 41.6 (s, CH, 2C), 39.3 (s, CH_2_, 2C), 37.4 (s, CH_2_, 2C), 21.0 (s, CH_2_, 2C), 20.6 (s, CH_2_, 2C), 14.9 (s, CH_3_, 2C), 14.2 (s, CH_3_, 2C).

**^31^P NMR** (242.98 MHz, CD_2_Cl_2_, 300 K): *δ* [ppm] = –14.0 (s).

**IR (ATR):** ν[cm^-1^] = 3069, 2958, 2931, 2871, 1594, 1534, 1452, 1436, 1360, 1210, 1054, 804, 746, 699, 657.

**Mass spectrometry (MS-ESI, +)**: *calcd.* for [M–BF_4_]^+^ C_52_H_57_NP^+^: 726.4223, *found*: 726.4234.

**m.p.** [°C]: 155 (decomp.).

**2-(2,6-diisopropylphenyl)-4-(diphenylphosphino)-5,7-dimethoxy-1,1-diphenyl-1*H*-isoindol-2-ium tetrafluoroborate (5f)**

According to **GP7**. The product was obtained as a dark-red solid. Yield: 89 % (6.78 g,
8.90 mmol).

**^1^H NMR** (600.24 MHz, CD_2_Cl_2_, 300 K): *δ* [ppm] = 8.36 (s, 1H), 7.50–7.45 (m, 4H), 7.45–7.38 (m, 9H), 7.31 (t, *J* = 7.5 Hz, 4H), 7.11–7.09 (m, 3H), 6.94 (d, *J* = 7.9 Hz, 4H), 3.85 (s, 3H), 3.65 (s, 3H), 1.92 (sept, *J* = 6.8 Hz, 2H), 0.87 (d, *J* = 6.8 Hz, 6H), 0.24 (d, *J* = 6.8 Hz, 6H).

**13C{1H} NMR** (150.93 MHz, CD_2_Cl_2_, 300 K): *δ*[ppm] = 174.0 (d, *J* = 13.0 Hz, CH, 1C), 166.5 (d, *J* = 8.5 Hz, Cq, 1C), 157.4 (s, Cq, 1C), 146.3 (s, Cq, 2C), 137.1 (d, *J* = 1.5 Hz, Cq, 1C), 135.2 (bd, *J* = 8.0 Hz, Cq, 2C), 134.6 (d, *J* = 11.0 Hz, Cq, 1C), 133.4 (d, *J* = 20.2 Hz, CH, 4C), 132.9 (s, Cq, 1C), 132.7 (s, CH, 1C), 130.8 (s, CH, 2C), 130.7 (bs, CH, 4C), 130.1 (s, CH, 2C), 129.6 (d, *J* = 7.0 Hz, CH, 4C), 129.5 (s, Cq, 2C), 129.0 (s, CH, 4C), 125.7 (s, CH, 2C), 106.0 (s, CH, 1C), 96.3 (s, Cq, 1C), 57.4 (s, CH_3_, 1C), 57.1 (s, CH_3_, 1C), 30.6 (s, CH, 2C), 26.5 (s, CH_3_, 2C), 21.7 (s, CH_3_, 2C).

**31P{1H} NMR** (121.65 MHz, CD_2_Cl_2_, 300 K): *δ*[ppm] = –17.9 (s).

**IR (ATR):** ν[cm^-1^] = 3014, 2973, 2956, 1589, 1547, 1467, 1436, 1366, 1313, 1277, 1219, 1189, 1124, 1060, 971, 944, 856, 814, 763, 740, 711, 699, 658.

**Mass spectrometry (MS-ESI, +)**: *calcd.* for [M–BF_4_]^+^ C_46_H_45_NPO_2_^+^: 674.3182, *found*: 674.3195.

**m.p.** [°C]: 212 (decomp.).

**6-(tert-butyl)-2-(2,6-diisopropylphenyl)-4-(diphenylphosphino)-1,1-diphenyl-1*H*-isoindol-2-ium tetrafluoroborate (5g)**

According to **GP7**. The product was obtained as an orange solid. Yield: 80 % (6.06 g, 8.00 mmol).

**^1^H NMR** (600.24 MHz, CD_2_Cl_2_, 300 K): *δ* [ppm] = 8.99 (s, 1H), 7.53–7.44 (m, 13H), 7.42 (d,
 *J* = 1.3 Hz, 1H), 7.37–7.34 (m, 4H), 7.23 (d, *J* = 1.3 Hz, 1H), 7.12 (d, *J* = 7.8 Hz, 2H), 6.98–6.94 (m, 4H), 1.88 (sept, *J* = 6.7 Hz, 2H), 1.18 (s, 9H), 0.72 (d, *J* = 6.9 Hz, 6H), 0.23 (d, *J* =
6.7 Hz, 6H).

**13C{1H} NMR** (121.65 MHz, CD_2_Cl_2_, 300 K): *δ*[ppm] = 172.0 (bs, CH, 1C), 164.6 (s, Cq, 1C), 158.3 (s, Cq, 1C), 146.6 (s, Cq, 2C), 134.8 (bs, CH, 4C), 134.1 (bs, Cq, 2C), 133.5 (bs, CH, 1C), 133.1 (s, Cq, 1C), 132.8 (s, CH, 1C), 131.2 (s, CH, 2C), 131.1 (s, CH, 2C), 131.0 (s, Cq, 2C), 130.3 (s, CH, 4C), 130.0 (s, CH, 4C), 129.6 (s, CH, 4C), 129.6 (s, Cq, 1C), 128.5 (s, CH, 2C), 124.2 (s, CH, 1C), 96.5 (s, Cq, 1C), 37.0 (s, Cq, 1C), 30.7 (s, CH_3_, 3C), 30.7 (s, CH, 2C), 26.1 (s, CH_3_, 2C), 21.5 (s, CH_3_, 2C). *One quaternary carbon signal (1C) could not be detected.*

**31P{1H} NMR** (242.98 MHz, CD_2_Cl_2_, 300 K): *δ*[ppm] = –11.3 (s).

**IR (ATR):** ν[cm^-1^] = 3066, 2964, 2930, 2871, 1587, 1522, 1436, 1402, 1352, 1222, 1053, 933, 883, 807, 747, 700, 650, 628.

**Mass spectrometry (MS-ESI, +)**: *calcd.* for [M–BF_4_]^+^ C_48_H_49_NP^+^: 670.3597, *found*: 670.3600.

**m.p.** [°C]: 150 (decomp.).

**Synthesis of Cyclobutenone imine phosphine (6) rearrangement product**

In a glove box, the phosphine isoindolium salt **(5a)** (384 mg, 0.55 mmol, 1.00 equiv.) and lithium bis(trimethylsilyl)amide (101 mg, 0.60 mmol, 1.00 equiv.) were added to a Schlenk flask. At –­­­­­­­­­78 °C, pre-cooled toluene (12 mL) was added dropwise over 5–10 mins to the mixture while stirring. The suspension was allowed to warm to rt and was stirred for 1.5 h at rt. The solvent was removed under reduced pressure and the crude product was purified by column chromatography (PE/EA 20:1 to 5:1, Alox). The product **(6)** was obtained as a pale yellow powder. Yield: 35 % (118 mg, 0.31 mmol).

**1H NMR** (600.24 MHz, CD_2_Cl_2_, 300 K): *δ*[ppm] = 7.81 (d, *J* = 7.5 Hz, 1H), 7.65 (d, *J* = 7.8 Hz, 4H), 7.44 (t, *J* = 7.6 Hz, 1H), 7.37–7.32 (m, 4H), 7.28–7.22 (m, 4H), 7.22–7.18 (m, 4H), 7.07 (t, *J* = 7.7 Hz, 1H), 7.00–6.95 (m, 3H), 6.89–6.83 (m, 4H), 2.62 (sept, *J* = 6.8 Hz, 2H), 0.91 (d, *J* = 6.8 Hz, 6H), 0.65 (d, *J* = 6.9 Hz, 6H).

**13C{1H} NMR** (150.93 MHz, CD_2_Cl_2_, 300 K): *δ*[ppm] = 163.9 (s, Cq, 1C), 156.0 (d, *J* = 9.0 Hz, Cq, 1C), 148.3 (d, *J* = 33.5 Hz, Cq, 1C), 146.7 (s, Cq, 1C), 143.4 (s, Cq, 2C), 137.7 (d, *J* = 16.3 Hz, Cq, 2C), 137.2 (d, *J* = 3.2 Hz, Cq, 2C), 136.6 (bs, CH, 1C), 135.0 (d, *J* = 26.7 Hz, Cq, 1C), 133.8 (d, *J* = 20.4 Hz, CH, 4C), 133.2 (bs, CH, 1C), 128.8 (s, CH, 2C), 128.6 (d, *J* =
5.9 Hz, CH, 4C), 128.6 (s, CH, 4C), 127.8 (s, CH, 4C), 127.2 (s, CH, 2C), 124.1 (s, CH, 1C), 123.9 (s, CH, 1C), 123.1 (s, CH, 2C), 72.3 (s, Cq, 1C), 28.6 (s, CH, 2C), 22.9 (s, CH_3_, 2C), 22.0 (s, CH_3_, 2C).

**^31^P NMR** (242.98 MHz, CD_2_Cl_2_, 300 K): *δ* [ppm] = –19.2 (s).

**R_f_** = 0.28 (PE/EA 20:1)

**IR (ATR):** ν[cm^-1^] = 3053, 2959, 2923, 2866, 1694, 1584, 1493, 1446, 1433, 1381, 1359, 1327, 1253, 1034, 954, 871, 791, 745, 696, 644, 622.

**Mass spectrometry (MS-APCI, +)**: *calcd.* as [M+H]^+^ C_44_H_41_NP^+^: 614.2971, *found*: 614.2985.

**m.p.** [°C]: 74 (decomp.).

**GP 8: Synthesis of CAArCPhos palladium (II) dichloride complexes (7)**

Phosphine isoindolium salt **5** (0.50 mmol, 1.00 equiv.), sodium acetate (123.1 mg, 1.50 mmol, 3.00 equiv.) and palladium(II) chloride (110.9 mg, 0.63 mmol, 1.25 equiv. or 133.1 mg,
0.75 mmol, 1.50 equiv. in case of **7d**) were suspended in dry degassed toluene (12 mL) under vigorous stirring and were heated under reflux in a pre-heated oil bath. The reaction was stirred for 16 h (in case of **7d**: 3 days) and the suspension was allowed to cool to rt. The suspension was filtrated over Celite and the solid was washed with toluene (and diethyl ether in case of **7a**). The products **7** were extracted with DCM and the solvent was removed *in vacuo* to obtain the palladium carbenes as powders.

**2-(2,6-diisopropylphenyl)-4-(diphenylphosphino)-1,1-diphenyl-isoindol-3-ylidene palladium(II) chloride (7a)**

According to **GP8**. The suspension was filtrated over Celite and the solid was washed with dry toluene (50 mL) and dry diethyl ether (50 mL). The product was extracted with DCM and the solvent was removed *in vacuo* to afford the product **7a** as colorless to beige powder. Yield:
81 % (321 mg, 0.34 mmol). The reaction could be scaled up to 1.85 mmol with a yield of 77 % (1.16 g, 1.42 mmol). Colorless single-crystals of **7a** suitable for X-Ray structure analysis were obtained by slow diffusion from chloroform.

**1H NMR** (600.24 MHz, CD_2_Cl_2_, 300 K): *δ*[ppm] = 7.99–7.91 (m, 4H), 7.83–7.78 (m, 2H), 7.65–7.60 (m, 2H), 7.56–7.50 (m, 4H), 7.38–7.08 (m, 10H), 7.02 (d, *J* = 7.8 Hz, 2H), 6.76 (bs, 2H), 2.23 (sept, *J* = 6.4 Hz, 2H), 1.39 (d, *J* = 6.7 Hz, 1H), 0.07 (d, *J* = 6.7 Hz, 1H).

**13C{1H} NMR** (150.93 MHz, CD_2_Cl_2_, 300 K): *δ*[ppm] = 211.1 (d, *J* = 5.2 Hz, C_carbene_, 1C), 155.6 (d, *J* = 40.9 Hz, Cq, 1C), 149.6 (d, *J* = 17.0 Hz, Cq, 1C), 146.8 (s, Cq, 2C), 137.8 (s, Cq, 1C), 137.1 (d, *J* = 40.9 Hz, Cq, 1C), 136.3 (d, *J* = 5.7 Hz, CH, 1C), 134.6 (d, *J* = 11.7 Hz, CH, 4C), 133.8 (s, Cq, 2C), 132.4 (d, *J* = 3.0 Hz, CH, 2C), 131.5 (d, *J* = 2.3 Hz, CH, 1C), 131.1 (bs, CH, 2C), 130.1 (s, CH, 1C), 130.0 (bs, Cq, 1C), 129.8 (s, CH, 2C), 129.4 (d, *J* = 56.8 Hz, Cq, 1C), 129.2 (d, *J* = 11.8 Hz, CH, 4C), 128.9 (d, *J* = 2.3 Hz, CH, 1C), 128.7 (bs, CH, 4C), 124.7 (s, CH, 2C), 100.0 (s, Cq, 1C), 31.0 (s, CH, 2C), 26.2 (s, CH_3_, 2C), 23.0 (s, CH_3_, 2C).

**^31^P NMR** (242.98 MHz, CD_2_Cl_2_, 300 K): *δ* [ppm] = 52.6 (s).

**IR (ATR):** ν[cm^-1^] = 3057, 2960, 2928, 2866, 1586, 1466, 1452, 1435, 1384, 1319, 1274, 1250, 1184, 1159, 1101, 1054, 998, 973, 936, 858, 802, 786, 772, 739, 702, 689, 663, 636, 619.

**Mass spectrometry (MS-ESI, +)**: *calcd.* for [M–Cl]^+^ C_44_H_40_NPPdCl: 754.1616, found: 754.1670.

**m.p.** [°C]: 298 (decomp.).

**2-(2,6-diethylphenyl)-4-(diphenylphosphino)-1,1-diphenyl-isoindol-3-ylidene palladium(II) chloride (7b)**

According to **GP8**. The suspension was filtrated over Celite and the solid was washed with dry toluene (40 mL). The product was extracted with DCM and the solvent was removed *in vacuo* to afford the product as beige to pale yellow powder. Yield: 46 % (176 mg, 0.22 mmol).

**1H NMR** (600.24 MHz, CD_2_Cl_2_, 300 K): *δ*[ppm] = 7.98–7.24 (m, 4H), 7.85–7.82 (m, 2H), 7.64–7.60 (m, 2H), 7.56–7.52 (m, 4H), 7.36–7.33 (m, 2H), 7.31–7.26 (m, 2H), 7.21–7.18 (m, 4H), 7.00–6.97 (m, 4H), 6.95 (d, *J* = 7.8 Hz, 2H), 2.58–2.50 (m, 2H), 1.84–1.76 (m, 2H), 1.09 (t, *J* = 7.5 Hz, 6H).

**13C{1H} NMR** (150.93 MHz, CD_2_Cl_2_, 300 K): *δ*[ppm] = 210.0 (d, *J* = 5.0 Hz, C_carbene_, 1C), 155.7 (d, *J* = 41.0 Hz, Cq, 1C), 149.6 (d, *J* = 16.6 Hz, Cq, 1C), 142.3 (s, Cq, 2C), 138.4 (s, Cq, 1C), 137.1 (d, *J* = 41.2 Hz, Cq, 1C), 136.4 (d, *J* = 5.5 Hz, CH, 1C), 134.5 (d, *J* = 11.6 Hz, CH, 4C), 132.8 (s, Cq, 2C), 132.4 (d, *J* = 2.9 Hz, CH, 2C), 131.6 (d, *J* = 2.3 Hz, CH, 1C), 130.7 (s. CH, 4C), 129.8 (s, CH, 2C), 129.6 (s, CH, 1C), 129.4 (d, *J* = 56.3 Hz, Cq, 2C), 129.3 (d, *J* = 11.6 Hz, CH, 4C), 128.6 (d, *J* = 2.1 Hz, CH, 1C), 128.4 (s, CH, 4C), 125.7 (s, CH, 2C), 99.8 (s, Cq, 1C), 26.0 (s, CH_3_, 2C), 13.7 (s, CH_2_, 2C).

**^31^P NMR** (242.98 MHz, CD_2_Cl_2_, 300 K): *δ* [ppm] = 53.4 (s).

**IR (ATR):** ν[cm^-1^] = 3057, 2969, 2930, 2873, 1696, 1586, 1470, 1454, 1435, 1375, 1322, 1274, 1185, 1099, 1034, 998, 973, 862, 808, 742, 701, 662, 637, 619.

**Mass spectrometry (MS-ESI, +)**: *calcd.* for [M–Cl]^+^ C_42_H_36_NPPdCl: 810.2242, found: 810.2333.

**m.p.** [°C]: 286 (decomp.).

**2-(2,6-diethyl-4-methylphenyl)-4-(diphenylphosphino)-1,1-diphenyl-isoindol-3-ylidene palladium(II) chloride (7c)**

According to **GP8**. The suspension was filtrated over Celite and the solid was washed with dry toluene (40 mL). The product was extracted with DCM and the solvent was removed *in vacuo* to afford the product as a beige to pale-green powder. Yield: 50 % (190 mg, 0.25 mmol).

**1H NMR** (600.24 MHz, CD_2_Cl_2_, 300 K): *δ*[ppm] = 7.98–7.90 (m, 4H), 7.84–7.80 (m, 2H), 7.64–7.60 (m, 2H), 7.56–7.51 (m, 4H), 7.37–7.32 (m, 2H), 7.26–7. 32 (m, 2H, *overlap with residual toluene*), 7.21–7.16 (m, 3H, *overlap with residual toluene*), 7.00–6.96 (m, 4H), 6.76 (s, 2H), 2.33 (s, 3H), 2.20–2.13 (m, 2H), 1.47–1.39 (m, 2H), 0.75 (t, *J* = 7.5 Hz, 6H).

**13C{1H} NMR** (150.93 MHz, CD_2_Cl_2_, 300 K): *δ*[ppm] = 209.9 (d, *J* = 5.1 Hz, C_carbene_, 1C), 155.8 (d, *J* = 40.9 Hz, Cq, 1C), 149.6 (d, *J* = 16.8 Hz, Cq, 1C), 141.9 (s, Cq, 2C), 139.4 (s, Cq, 1C), 137.0 (d, *J* = 41.4 Hz, Cq, 1C), 136.3 (d, *J* = 5.5 Hz, CH, 1C), 136.0 (s, Cq, 1C), 134.5 (d, *J* = 11.6 Hz, CH, 4C), 134.5 (bs, CH, 1C), 132.9 (s, Cq, 2C), 132.4 (d, *J* = 2.9 Hz, CH, 2C), 131.5 (d, *J* = 2.4 Hz, CH, 1C), 130.7 (s, CH, 4C), 129.8 (s, CH, 2C), 129.4 (d, *J* = 56.3 Hz, Cq, 2C), 129.2 (d, *J* = 11.8 Hz, CH, 4C), 128.4 (s, CH, 4C), 126.5 (s, CH, 2C), 99.7 (s, Cq, 1C), 25.9 (s, CH_3_, 2C), 21.5 (s, CH_3_, 1C), 13.8 (s, CH_2_, 2C).

**^31^P NMR** (242.98 MHz, CD_2_Cl_2_, 300 K): *δ* [ppm] = 53.2 (s).

**IR (ATR):** ν[cm^-1^] = 3058, 3024, 2971, 2931, 2874, 1604, 1584, 1493, 1470, 1435, 1376, 1332, 1274, 1186, 1159, 1100, 1030, 998, 973, 855, 800, 779, 722, 694, 637.

**Mass spectrometry (MS-ESI, +)**: *calcd.* for [M–Cl]^+^ C_43_H_38_NP^108^PdCl: 742.1464, found: 742.1483.

**m.p.** [°C]: 295 (decomp.).

**2-(2,6-di(pentan-3-yl)phenyl)-4-(diphenylphosphino)-1,1-diphenyl-isoindol-3-ylidene palladium(II) chloride (7d)**

According to **GP8**. The suspension was filtrated over Celite and the solid was washed with dry toluene (100 mL). The product was obtained as a pale yellow powder. Yield: 38 % (161 mg, 0.24 mmol).

**1H NMR** (600.24 MHz, CD_2_Cl_2_, 300 K): *δ*[ppm]= 7.97–7.90 (m, 4H), 7.80–7.74 (m, 2H), 7.64–7.59 (m, 2H), 7.55–7.50 (m, 4H), 7.35–7.11 (m, 10H, *overlap with residual toluene*), 6.94 (d, *J* = 7.8 Hz, 2H), 6.80 (bs, 2H), 2.25–2.16 (m, 2H), 2.00–1.92 (m, 2H), 1.82–1.73 (m, 2H), 1.13–1.03 (m, 2H), 0.76 (t, *J* = 7.4 Hz, 6H), 0.36 (t, *J* = 7.4 Hz, 6H), 0.14–0.03 (m, 2H).

**13C{1H} NMR** (150.93 MHz, CD_2_Cl_2_, 300 K): *δ*[ppm]= 212.2 (d, *J* = 5.2 Hz, C_carbene_, 1C), 155.5 (d, *J* = 41.2 Hz, Cq, 1C), 149.8 (d, *J* = 16.9 Hz, Cq, 1C), 144.8 (s, Cq, 2C), 139.0 (s, Cq, 1C), 137.1 (d, *J* = 41.0 Hz, Cq, 1C), 136.2 (d, *J* = 5.3 Hz, CH, 1C), 134.7 (bs, Cq, 2C), 134.5 (d, *J* = 11.5 Hz, CH, 4C), 132.3 (d, *J* = 2.9 Hz, CH, 2C), 131.6 (d, *J* = 2.4 Hz, CH, 1C), 130.7 (bs, CH, 2C), 129.7 (s, CH, 2C), 129.5 (bs, CH, 2C), 129.5 (d, *J* = 57.1 Hz, Cq, 2C), 129.3 (s, CH, 1C), 129.2 (d, *J* = 11.8 Hz, CH, 4C), 129.1 (bs, CH, 2C), 128.9 (d, *J* = 2.2 Hz, CH, 1C), 128.8 (bs, CH, 1C), 128.7 (s, CH, 1C), 126.7 (s, CH, 2C), 99.8 (s, Cq, 1C), 41.9 (s, CH, 2C), 25.7 (s, CH_2_, 2C), 24.8 (s, CH_2_, 2C), 11.8 (s, CH_3_, 2C), 9.7 (s, CH_3_, 2C).

**^31^P NMR** (242.98 MHz, CD_2_Cl_2_, 300 K): *δ* [ppm] = 51.3 (s).

**IR (ATR):** ν[cm^-1^] = 3059, 2959, 2933, 2872, 1630, 1585, 1451, 1436, 1378, 1273, 1249, 1185, 1158, 1100, 999, 974, 905, 786, 773, 735, 698, 637, 618.

**Mass spectrometry (MS-ESI, +)**: *calcd.* for [M–Cl]^+^ C_48_H_48_NPPdCl: 810.2242, found: 810.2333.

**m.p.** [°C]: 293 (decomp.).

**2-(2,6-diisopropylphenyl)-4-(diphenylphosphino)-5,7-dimethoxy-1,1-diphenyl-isoindol-3-ylidene palladium(II) chloride (7f)**

According to **GP8**. The suspension was filtrated over Celite and the solid was washed with dry toluene (60 mL). The product was extracted with DCM and the solvent was removed *in vacuo*. The solid was washed with THF (5 x 1 mL) to afford the product **7f** as a beige powder. Yield:
56 % (234 mg, 0.29 mmol).

**1H NMR** (600.24 MHz, CD_2_Cl_2_, 300 K): *δ*[ppm] = 7.79–7.74 (m, 2H), 7.64–7.56 (m, 8H), 7.35–7.26 (m, 4H), 7.15–7.04 (m, 2H), 6.95–6.85 (m, 3H), 6.84–6.80 (m, 1H), 6.77–6.66 (m, 4H), 3.80 (s, 3H), 3.62 (s, 3H), 2.04 (sept, *J* = 6.7 Hz, 2H), 1.21 (d, *J* = 6.9 Hz, 6H), 0.07 (d, *J* = 6.6 Hz, 6H).

**13C{1H} NMR** (150.93 MHz, CD_2_Cl_2_, 300 K): *δ*[ppm] = 203.5 (d, *J* = 5.0 Hz, C_carbene_, 1C), 163.9 (s, Cq, 1C), 161.2 (s, Cq, 1C), 155.6 (d, *J* = 36.8 Hz, Cq, 1C), 146.6 (s, Cq, 2C), 135.7 (s, Cq, 1C), 134.1 (m, CH, 4C), 133.6 (s, CH, 2C), 131.8 (bs, CH, 2C), 131.3 (s, Cq, 2C), 130.7 (s, CH, 1C), 130.2 (s, CH, 2C), 130.0 (bs, CH, 2C), 129.7 (m, CH, 4C), 129.5 (d, *J* = 17.4 Hz, Cq, 1C), 129.0 (bs, CH, 2C), 128.2 (bs, CH, 2C), 125.3 (s, CH, 2C), 125.2 (d, *J* = 62.2 Hz, Cq, 2C), 106.5 (s, Cq, 1C), 104.2 (s, CH, 1C), 100.9 (s, Cq, 1C), 57.6 (s, CH_3_, 1C), 57.6 (s, CH_3_, 1C), 30.5 (s, CH, 2C), 26.4 (s, CH_3_, 2C), 22.7 (s, CH_3_, 2C).

**^31^P NMR** (242.98 MHz, CD_2_Cl_2_, 300 K): *δ* [ppm] = 58.6 (s).

**IR (ATR):** ν[cm^-1^] = 2959, 1691, 1621, 1571, 1501, 1464, 1438, 1348, 1227, 1104, 1054, 998, 946, 911, 811, 748, 715, 689, 628.

**Mass spectrometry (MS-ESI, +)**: *calcd.* for [M–Cl]^+^ C_46_H_44_NPO_2_PdCl: 814.1828, found: 814.1834.

**m.p.** [°C]: 292 (decomp.).

**2-(2,6-diisopropylphenyl)-4-(diphenylphosphino)-1,1-diphenyl-isoindol-3-ylidene palladium(II) chloride (7g)**

According to **GP8**. The suspension was filtrated over Celite and the solid was washed with dry toluene (100 mL). The product was extracted with DCM and the solvent was removed *in vacuo* to afford the product (**7g**) as a beige powder. Yield: 25 % (104 mg, 0.12 mmol). Yellow single-crystals of (**7g**) were obtained from DCM.

**1H NMR** (600.24 MHz, CD_2_Cl_2_, 300 K): *δ*[ppm] = 8.00–7.95 (m, 4H), 7.83 (d, *J* = 8.0 Hz, 1H), 7.66–7.62 (m, 2H), 7.58–7.54 (m, 4H), 7.40–7.11 (m, 10H, *overlap with residual toluene*), 7.05 (d, *J* = 7.7 Hz, 2H), 6.80 (bs, 2H), 2.27 (sept, *J* = 6.7 Hz, 2H), 1.43 (d, *J* = 6.9 Hz, 6H), 1.28 (s, 9H), 0.11 (d, *J* = 6.6 Hz, 6H).

**13C{1H} NMR** (150.93 MHz, CD_2_Cl_2_, 300 K): *δ*[ppm] = 209.9 (d, *J* = 5.3 Hz, C_carbene_, 1C), 161.6 (d, *J* = 4.9 Hz, Cq, 1C), 153.8 (d, *J* = 41.2 Hz, Cq, 1C), 149.4 (d, *J* = 17.7 Hz, Cq, 1C), 146.8 (s, Cq, 2C), 137.8 (s, Cq, 1C), 136.5 (d, *J* = 40.8 Hz, Cq, 1C), 134.5 (d, *J* = 11.6 Hz, CH, 4C), 134.0 (bs, Cq, 2C), 132.3 (d, *J* = 2.8 Hz, CH, 2C), 131.0 (bs, CH, 2C), 129.9 (s, CH, 1C), 129.6 (s, CH, 2C), 129.6 (d, *J* = 56.4 Hz, Cq, 2C), 129.1 (d, *J* = 11.8 Hz, CH, 4C), 128.7 (bs, CH, 4C), 128.5 (d, *J* = 2.3 Hz, CH, 1C), 126.3 (bs, CH, 1C), 124.6 (s, CH, 2C), 100.0 (s, Cq, 1C), 36.5 (s, Cq, 1C), 31.3 (s, CH_3_, 3C), 30.8 (s, CH, 2C), 26.2 (s, CH_3_, 2C), 23.0 (s, CH_3_, 2C). *One tertiary carbon resonance (2C) could not be detected.*

**^31^P NMR** (242.98 MHz, CD_2_Cl_2_, 300 K): *δ* [ppm] = 52.9 (s).

**IR (ATR):** ν[cm^-1^] = 3059, 2963, 2931, 2869, 1584, 1459, 1435, 1384, 1364, 1259, 1179, 1101, 1054, 999, 977, 929, 888, 802, 770, 746, 704, 691, 662.

**Mass spectrometry (MS-ESI, +)**: *calcd.* for [M–Cl]^+^ C_48_H_48_NP^108^PdCl: 812.2246, found: 812.2259.

**m.p.** [°C]: 298 (decomp.).

**GP 8: Catalytic screening of catalysts 7 in the Suzuki-Miyaura cross-coupling**

In 10 mL PTFE screw-cap vials, the catalyst **7** (3 mol%), 1-bromo-2-methoxynaphthalene **8** (23.7 mg, 1.00 equiv., 0.10 mmol), o-tolyl boronic acid **9**(40.8 mg, 3.00 equiv., 0.30 mmol), caesium carbonate (97.7 mg, 3.00 equiv., 0.30 mmol) and hexamethylbenzene **11** (5.4 mg, 0.33 equiv., 0.03 mmol) were suspended in dry deg. toluene (1.0 mL). The reaction was stirred vigorously in a pre-heated aluminium block at reflux for 2 d and the reaction mixture was extracted with DCM and filtrated over Celite. The solvent was removed *in vacuo* and the NMR yield was determined using the internal standard. The analytical data of product 2-methoxy-1-(*o*-tolyl)naphthalene **11** was in accordance with the literature.^[22]^

**GP 9: Catalytic screening of catalysts 7 in Miyaura-borylation reaction**

In 10 mL PTFE screw-cap vials, the catalyst **7** (5 mol%), 4-bromo-anisole (18.7 mg, 1.00 equiv., 0.10 mmol), bis(pinacolato)diboron (1.50 equiv., 0.15 mmol), potassium acetate (3.00 equiv., 0.30 mmol) and hexamethylbenzene (5.4 mg, 0.33 equiv., 0.03 mmol) were dissolved in dry deg. 1,4-dioxane (1.0 mL). The reaction was stirred in a pre-heated aluminium block at reflux for 18 h and the reaction mixture was extracted with DCM and filtrated over Celite. The solvent was removed *in vacuo* and the NMR yield was determined using the internal standard. The analytical data of 2-(4-methoxyphenyl)-4,4,5,5-tetramethyl-1,3,2-dioxaborolane was in accordance with the literature.^[23]^

1. **NMR spectra**


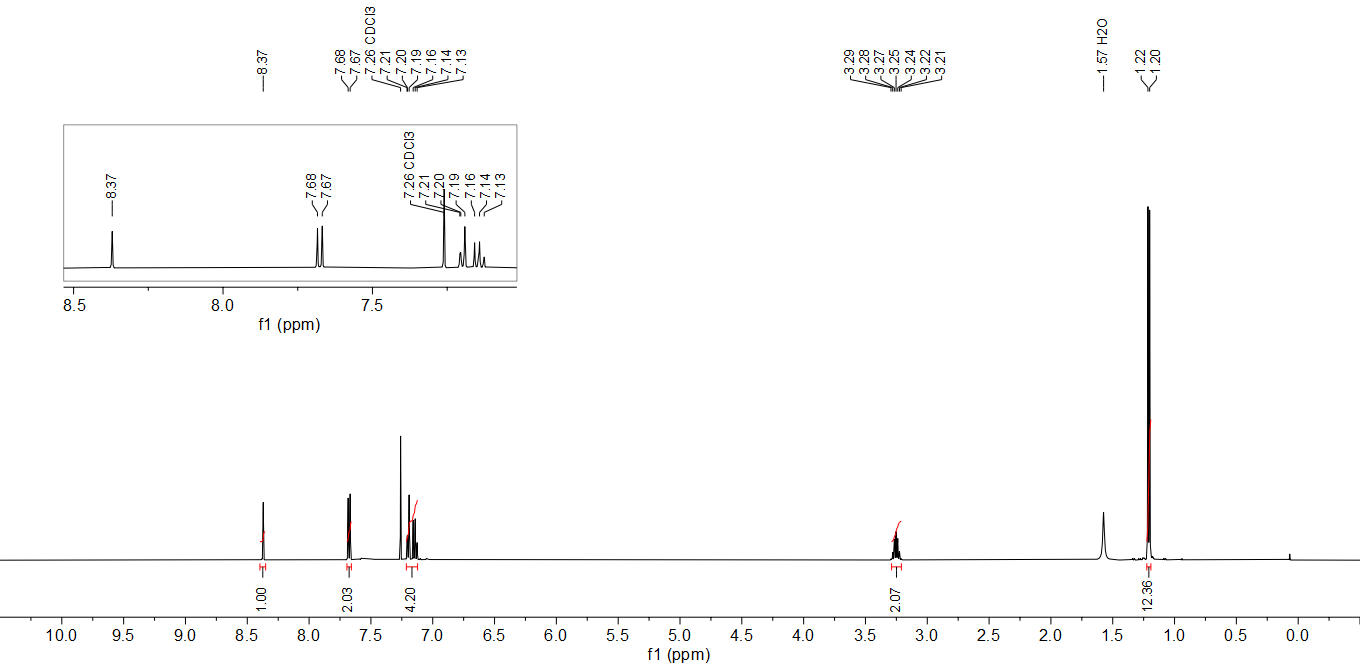


Figure S1: ^1^H NMR spectrum of imine **1a.**


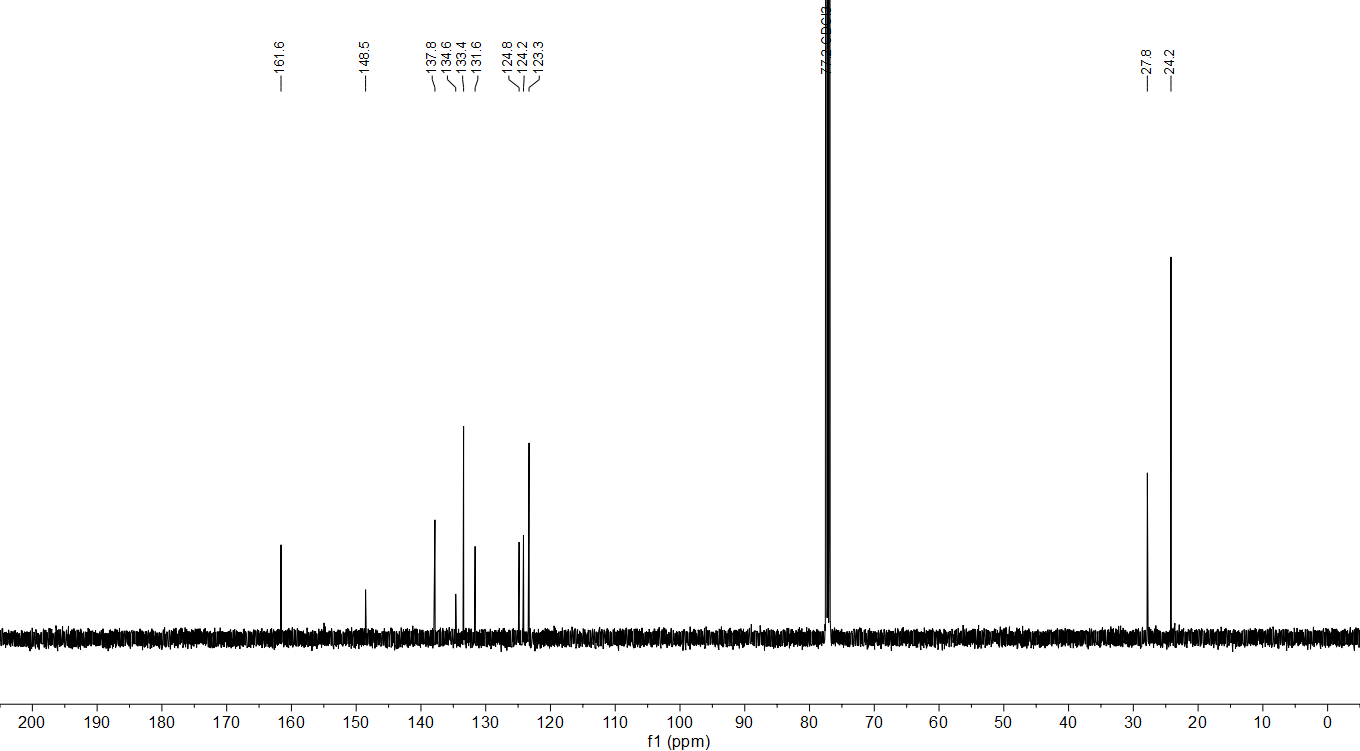


Figure S2: ^13^C{^1^H] NMR spectrum of imine **1a.**


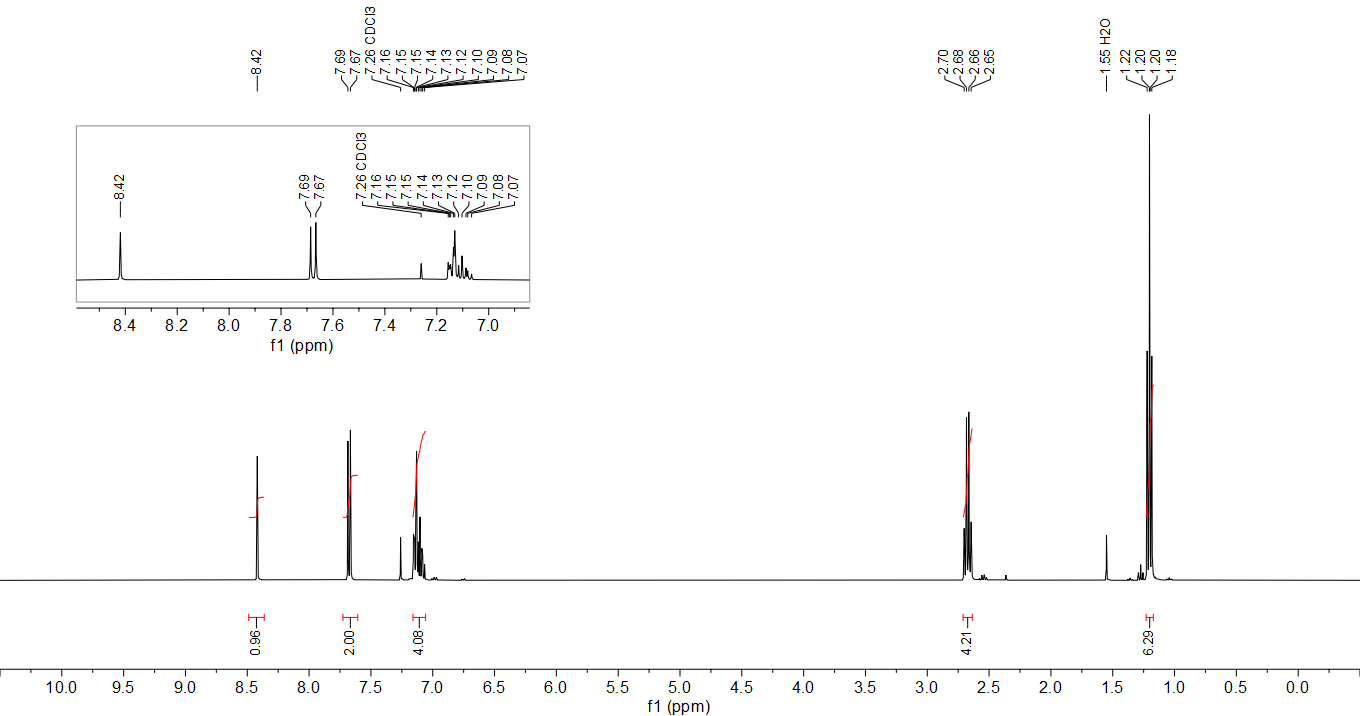


Figure S3: ^1^H NMR spectrum of imine **1b.**


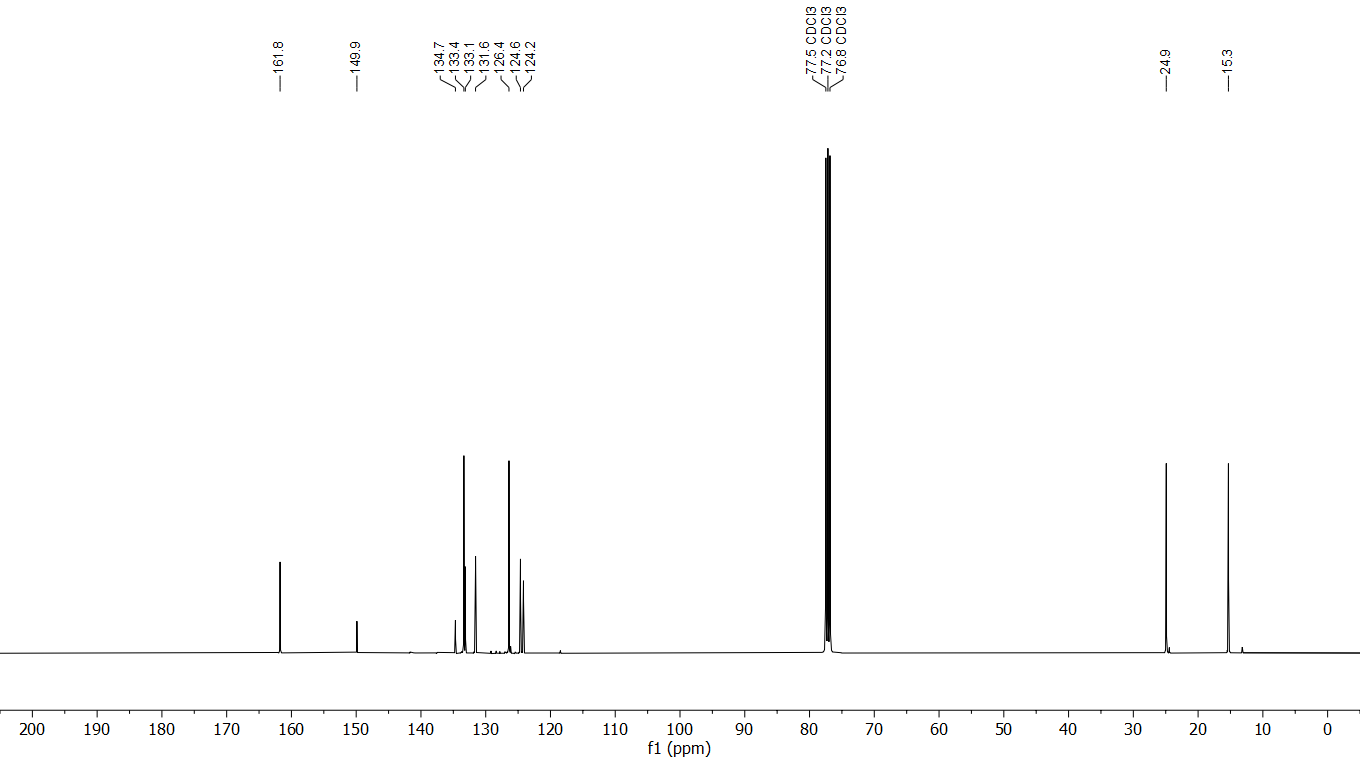


Figure S4: ^13^C{^1^H] NMR spectrum of imine **1b.**


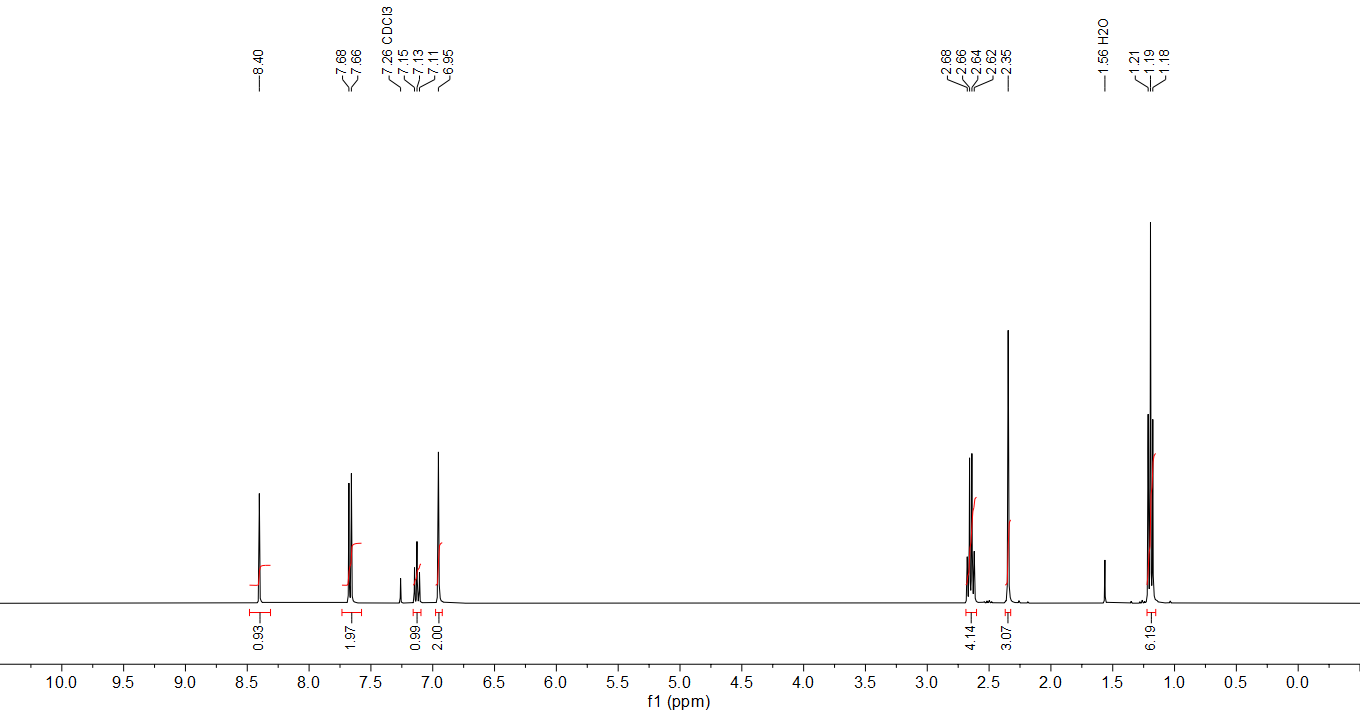


Figure S5: ^1^H NMR spectrum of imine **1c.**


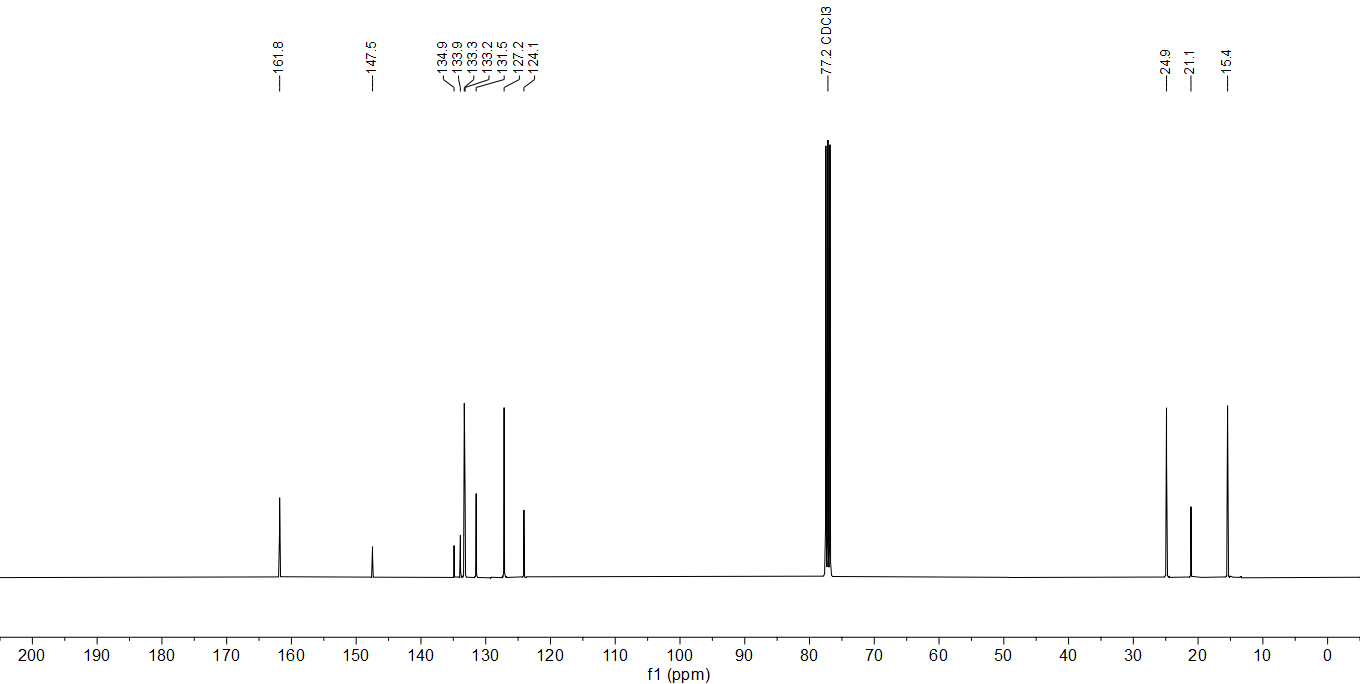


Figure S6: ^13^C{^1^H] NMR spectrum of imine **1c.**


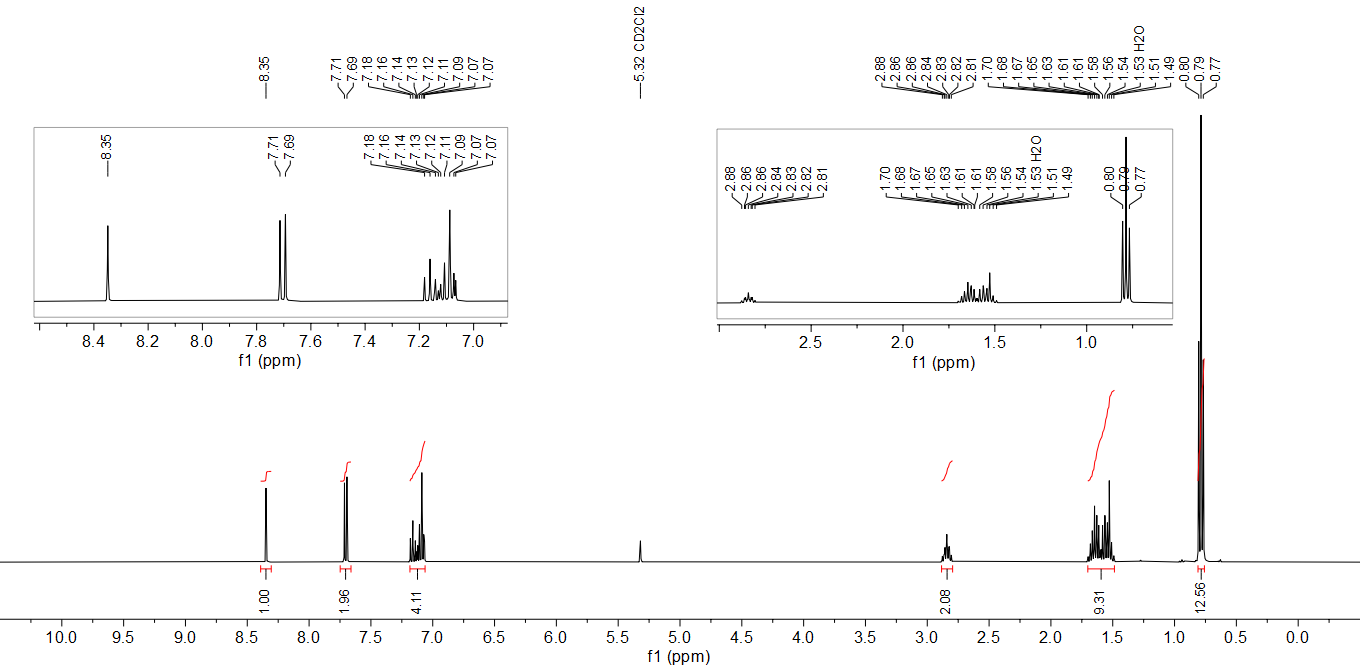


Figure S7: ^1^H NMR spectrum of imine **1d.**


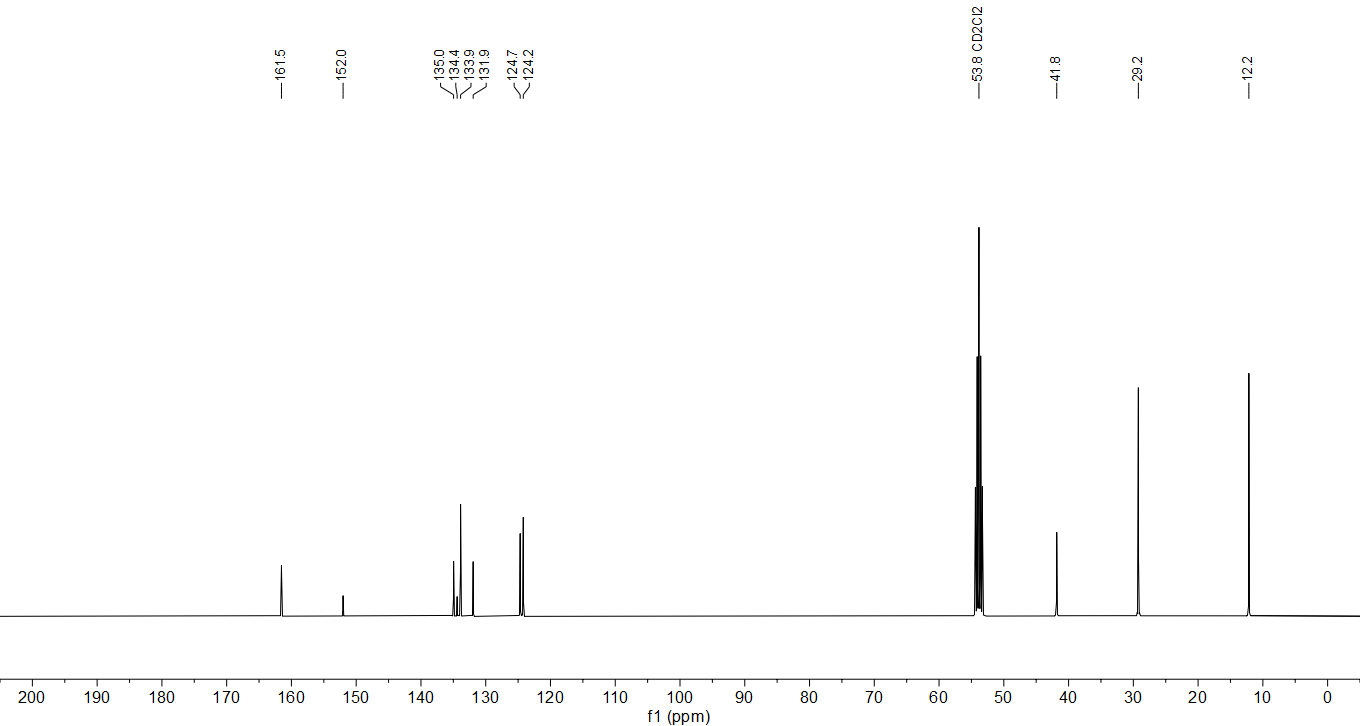


Figure S8: ^13^C{^1^H] NMR spectrum of imine **1d.**


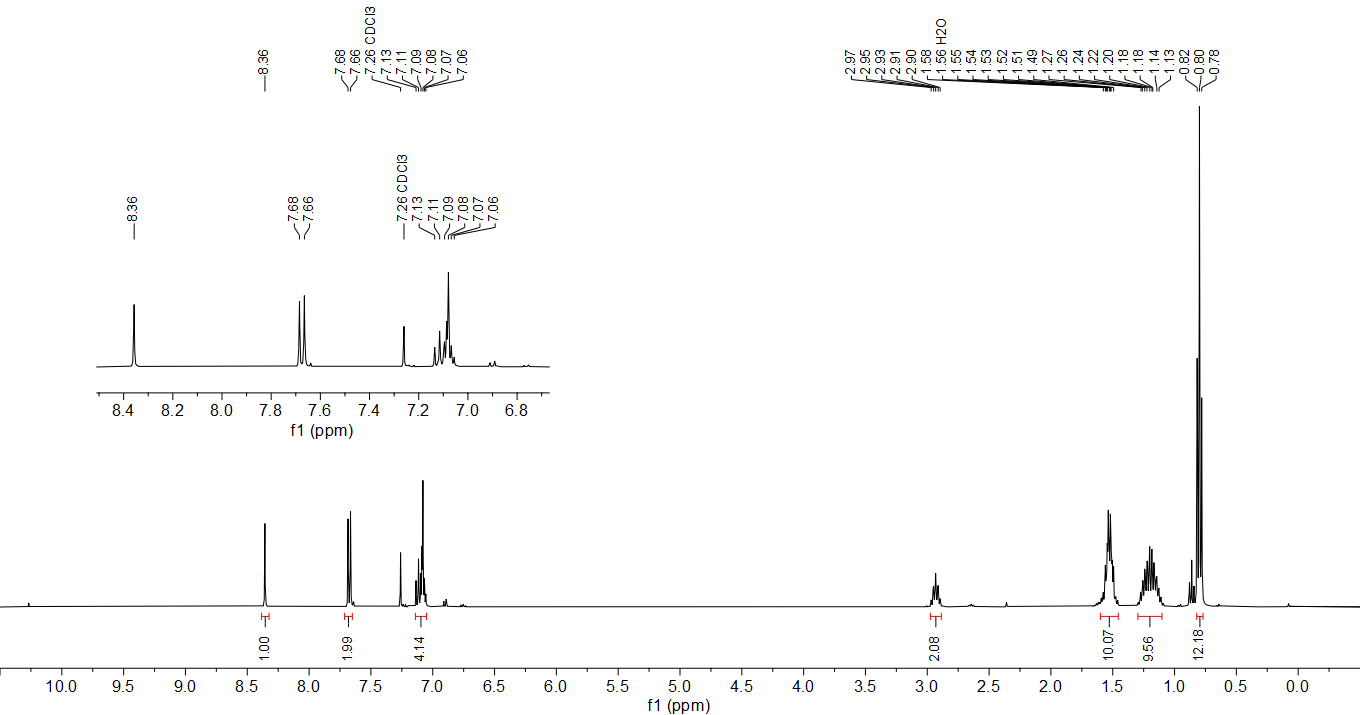


Figure S9: ^1^H NMR spectrum of imine **1e.**


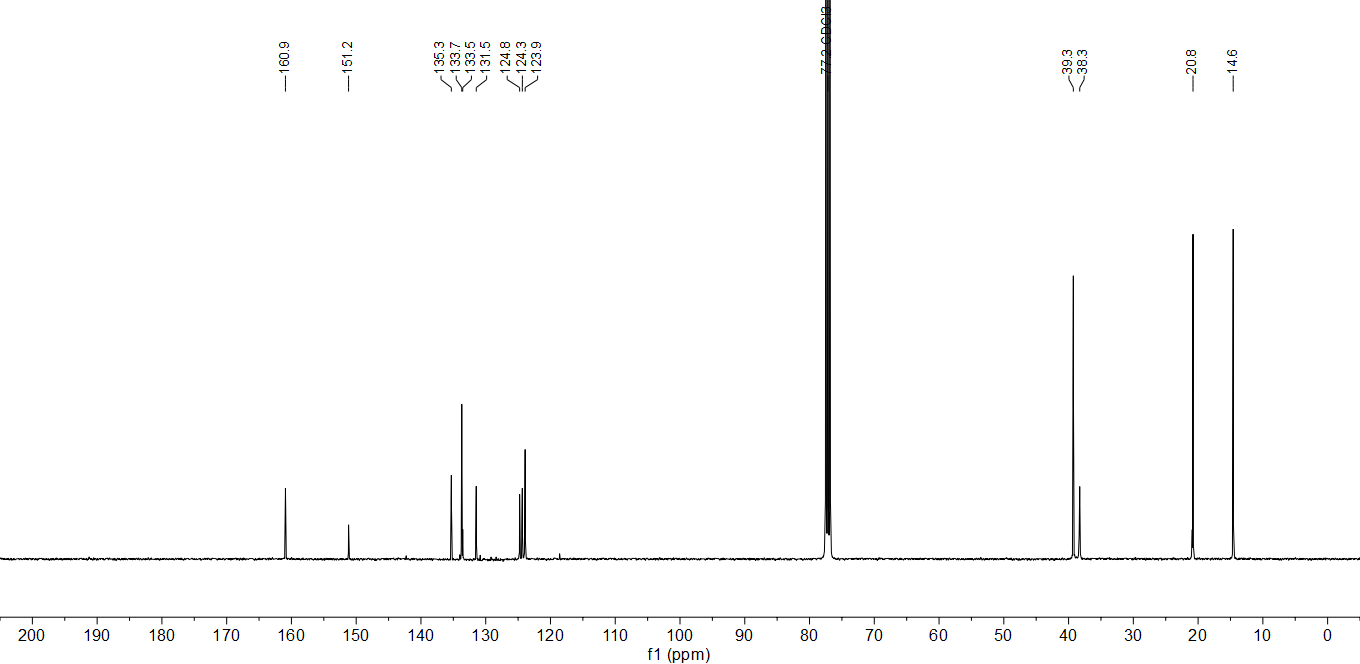


Figure S10: ^13^C{^1^H] NMR spectrum of imine **1e.**


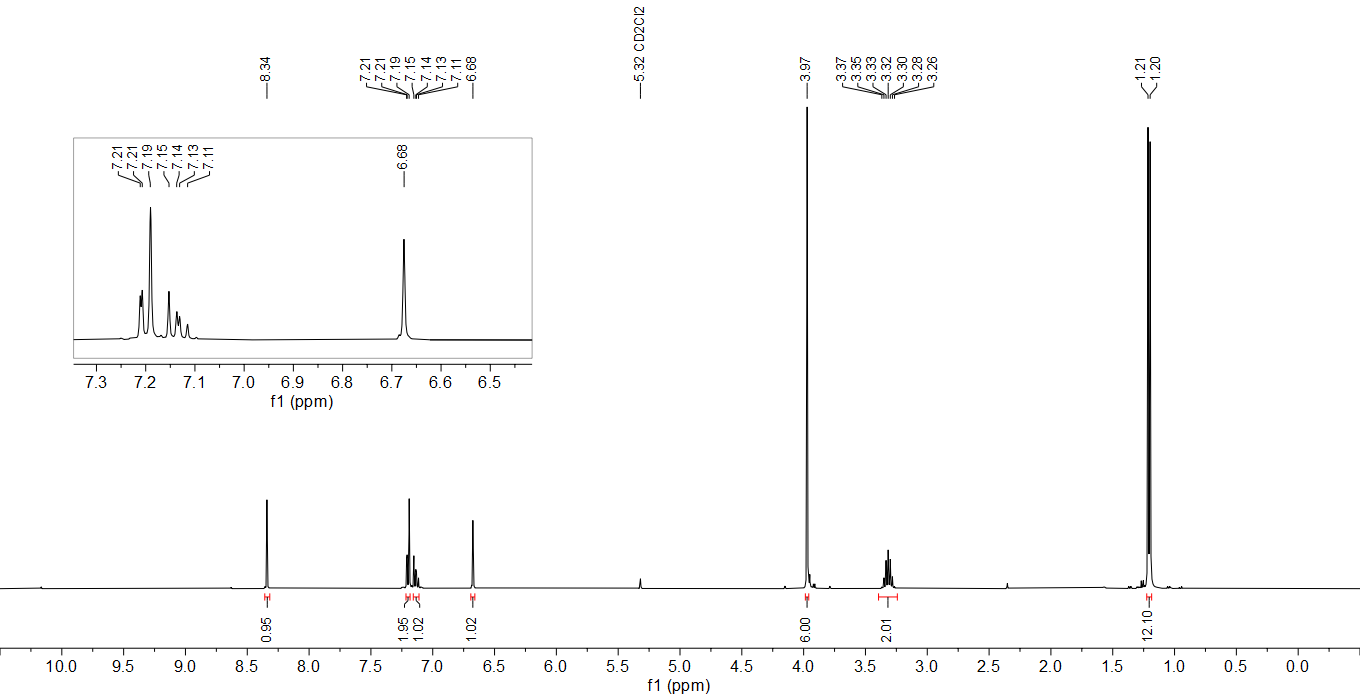


Figure S11: ^1^H NMR spectrum of imine **1f.**


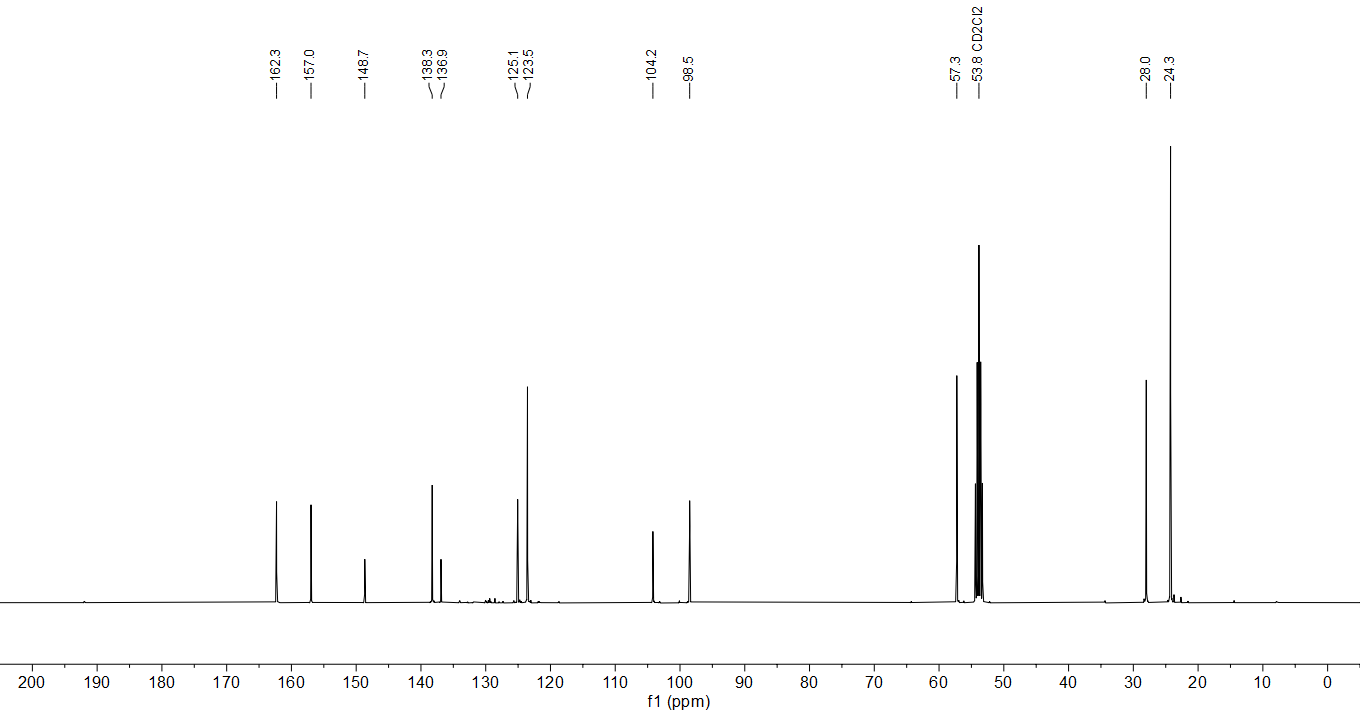


Figure S12:  ^13^C{^1^H] NMR spectrum of imine **1f.**


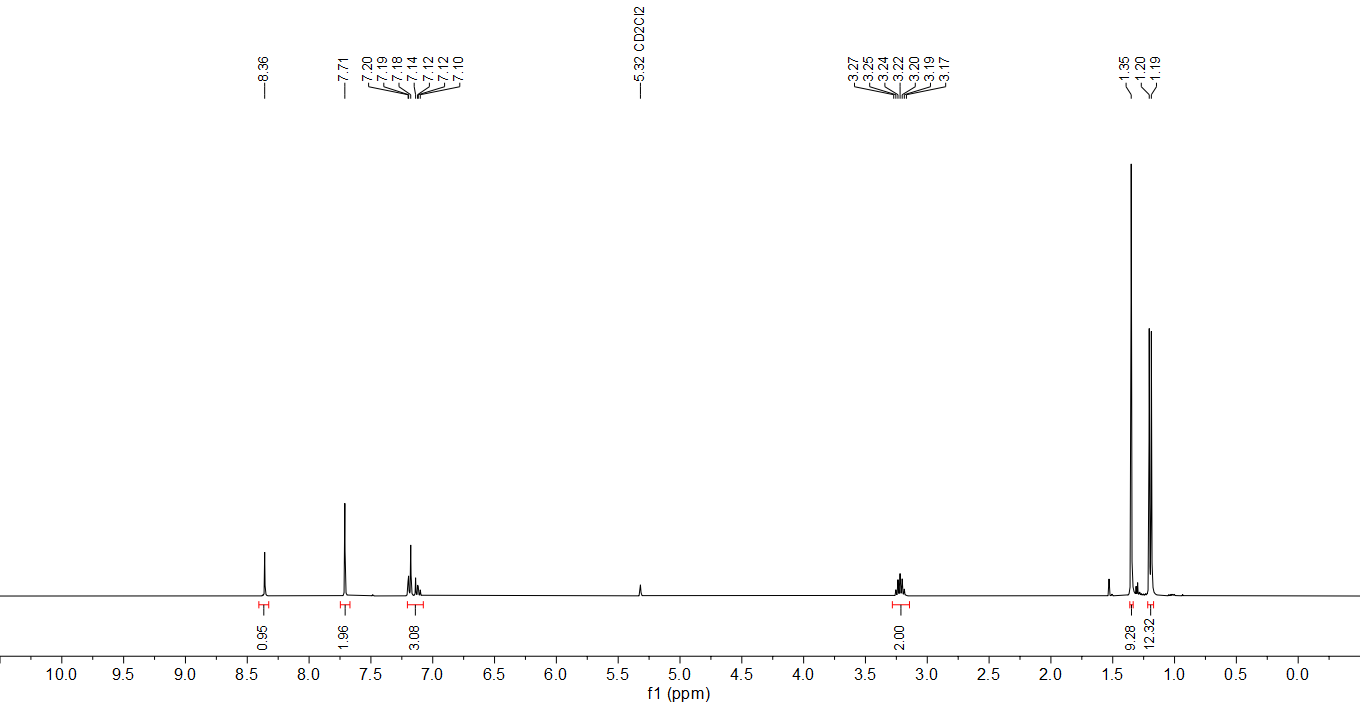


Figure S13: ^1^H NMR spectrum of imine **1g.**


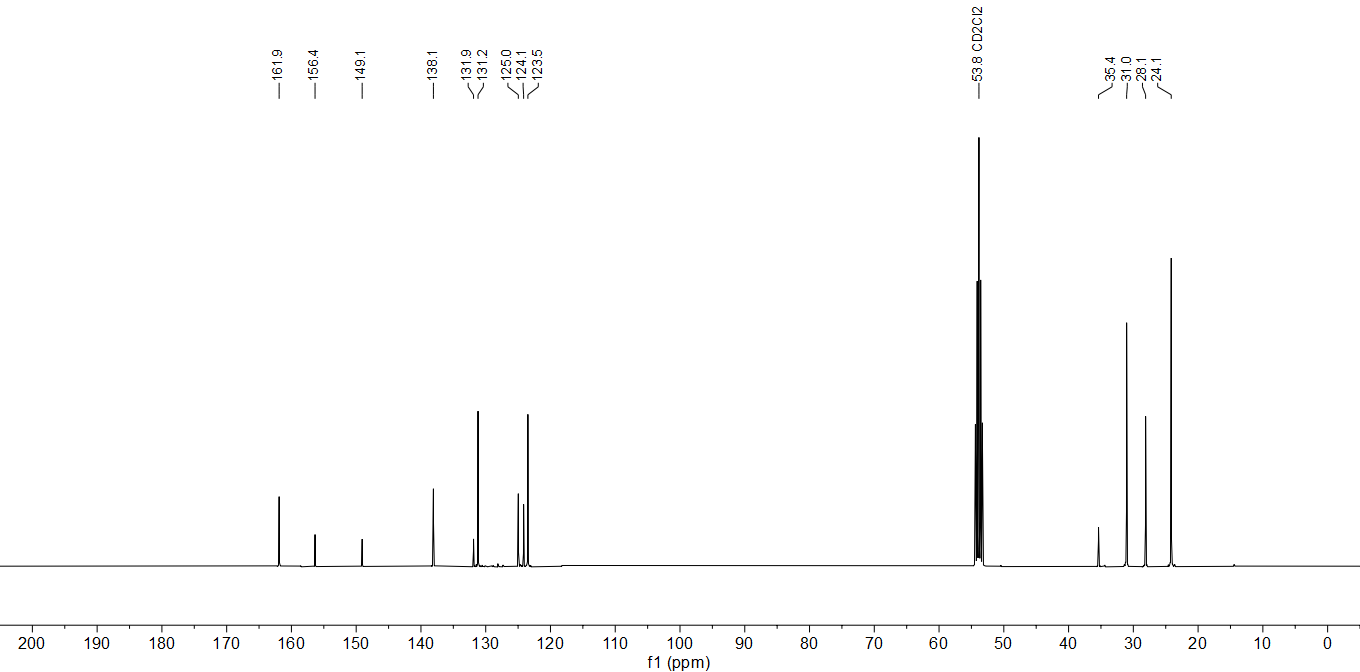


Figure S14: ^13^C{^1^H] NMR spectrum of imine **1g.**


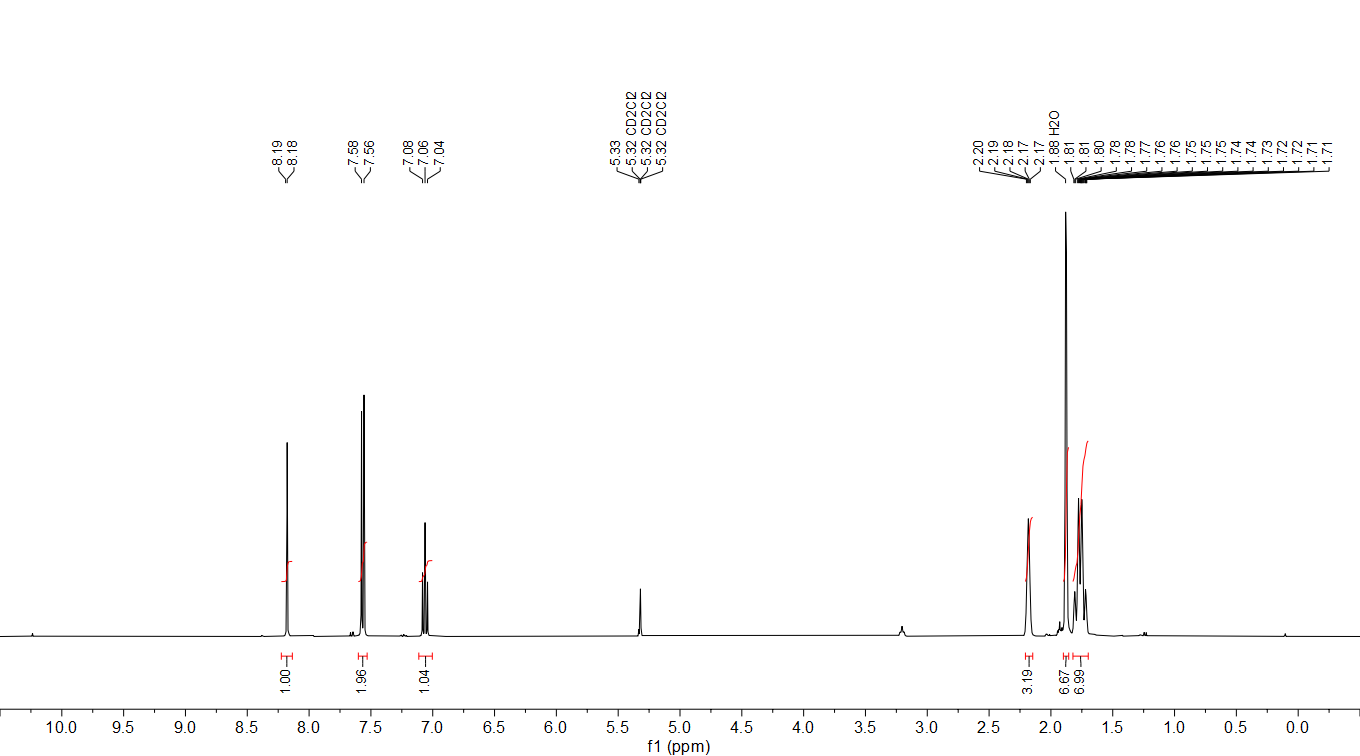


Figure S15: ^1^H NMR spectrum of imine **1h.**


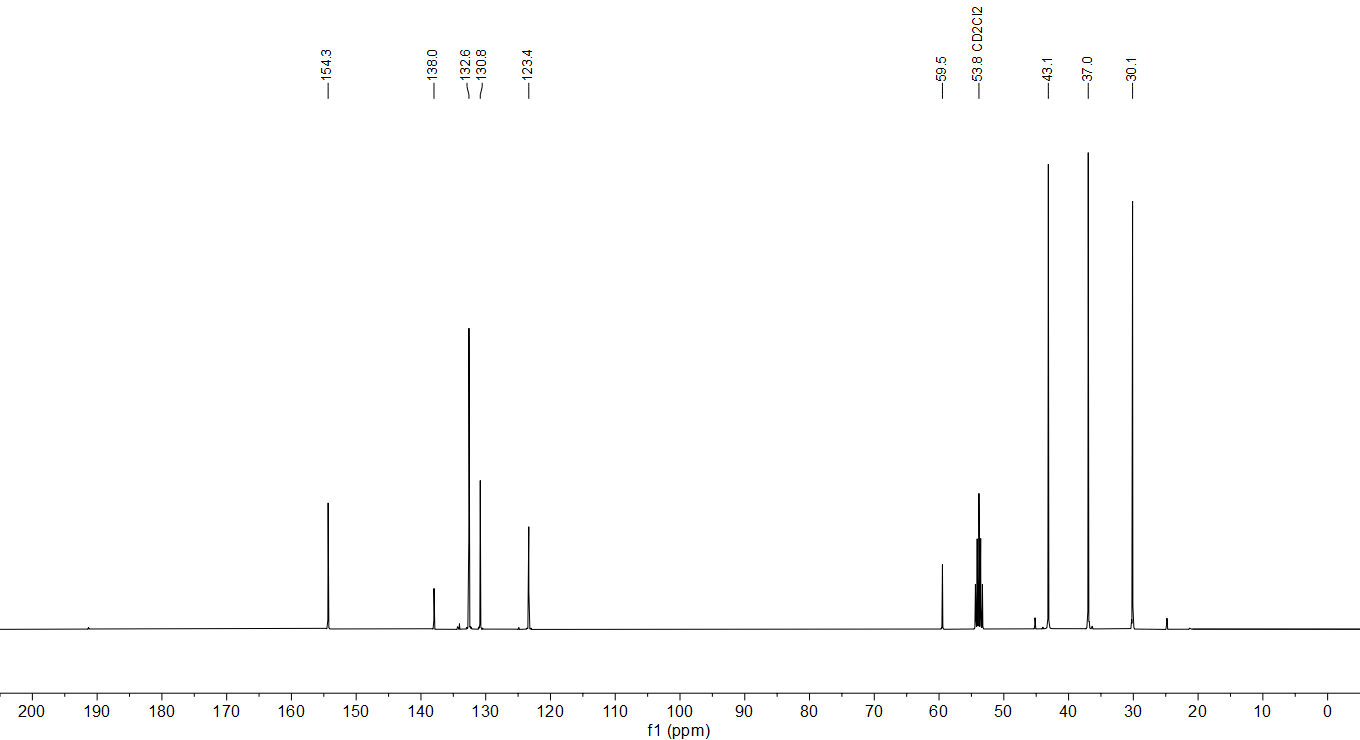


Figure S16: ^13^C{^1^H] NMR spectrum of imine **1h.**


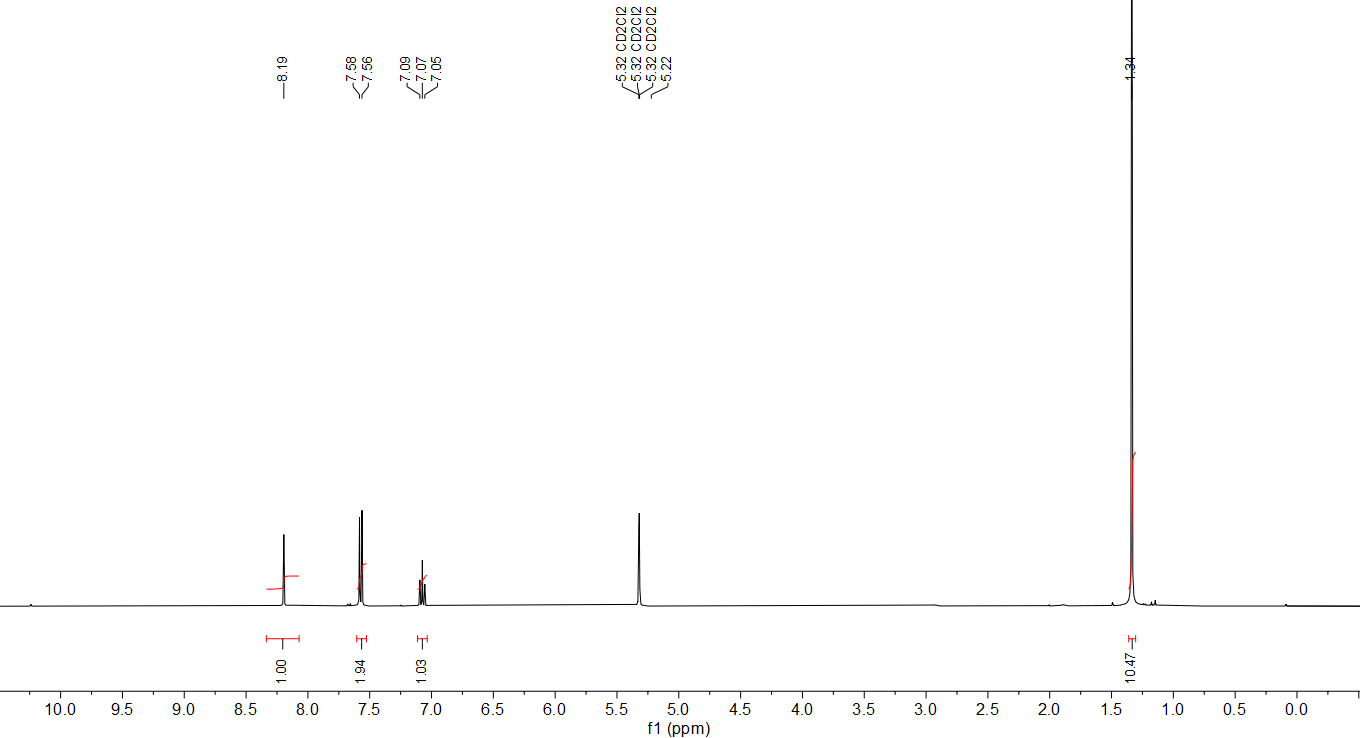


Figure S17: ^1^H NMR spectrum of imine **1i.**


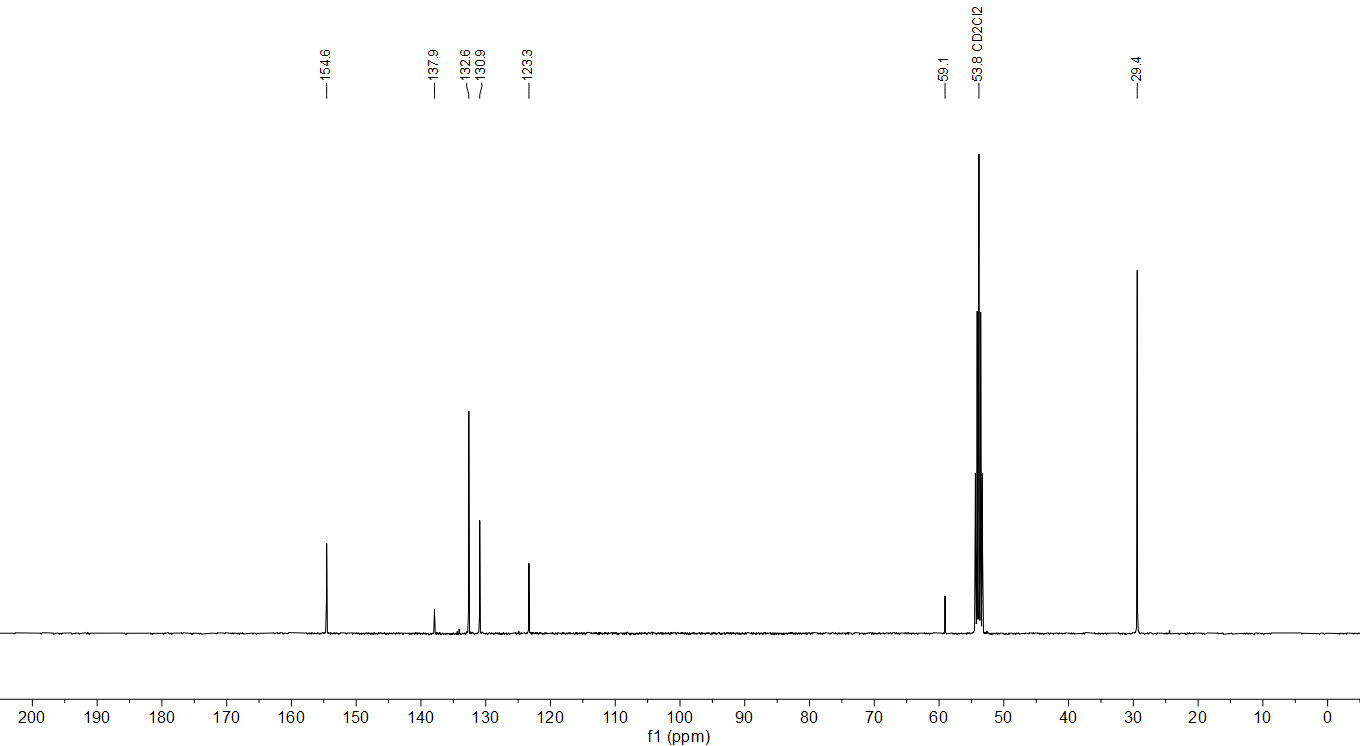


Figure S18: ^13^C{^1^H] NMR spectrum of imine **1i.**


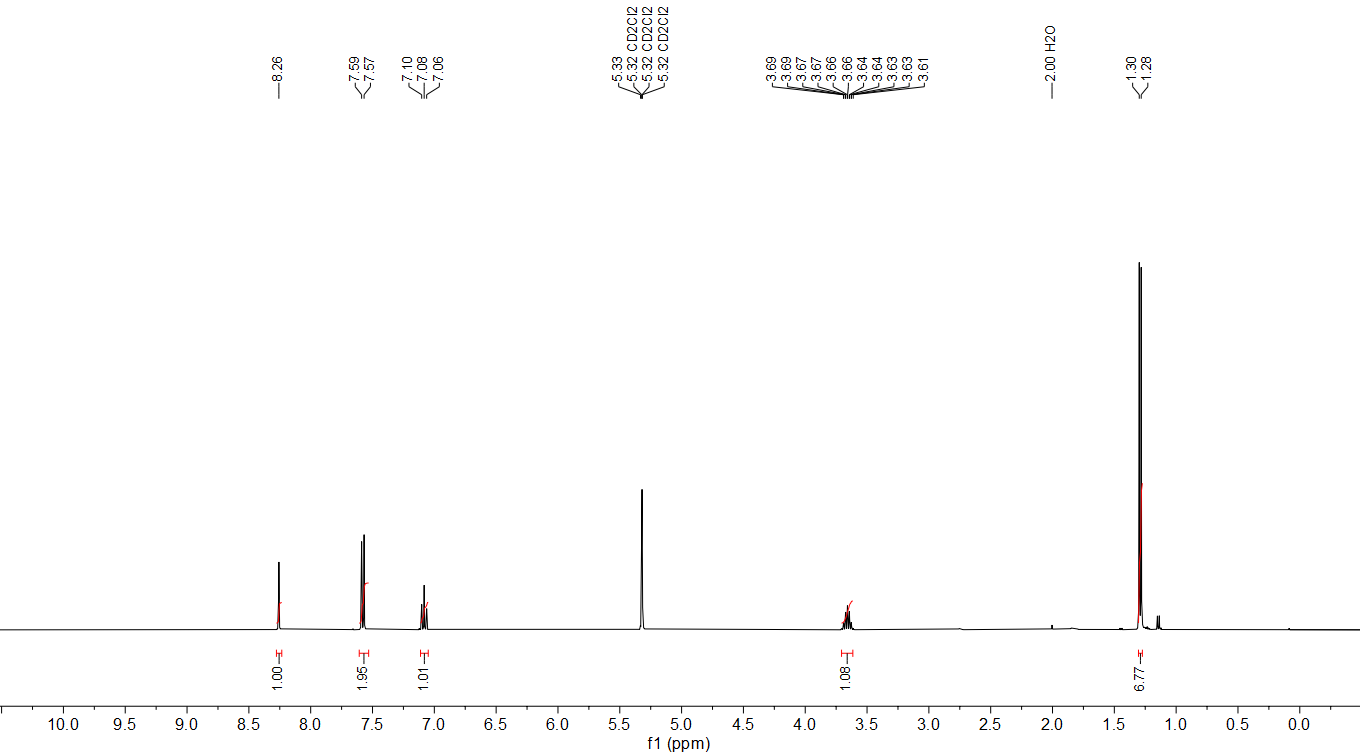


Figure S19: ^1^H NMR spectrum of imine **1j.**


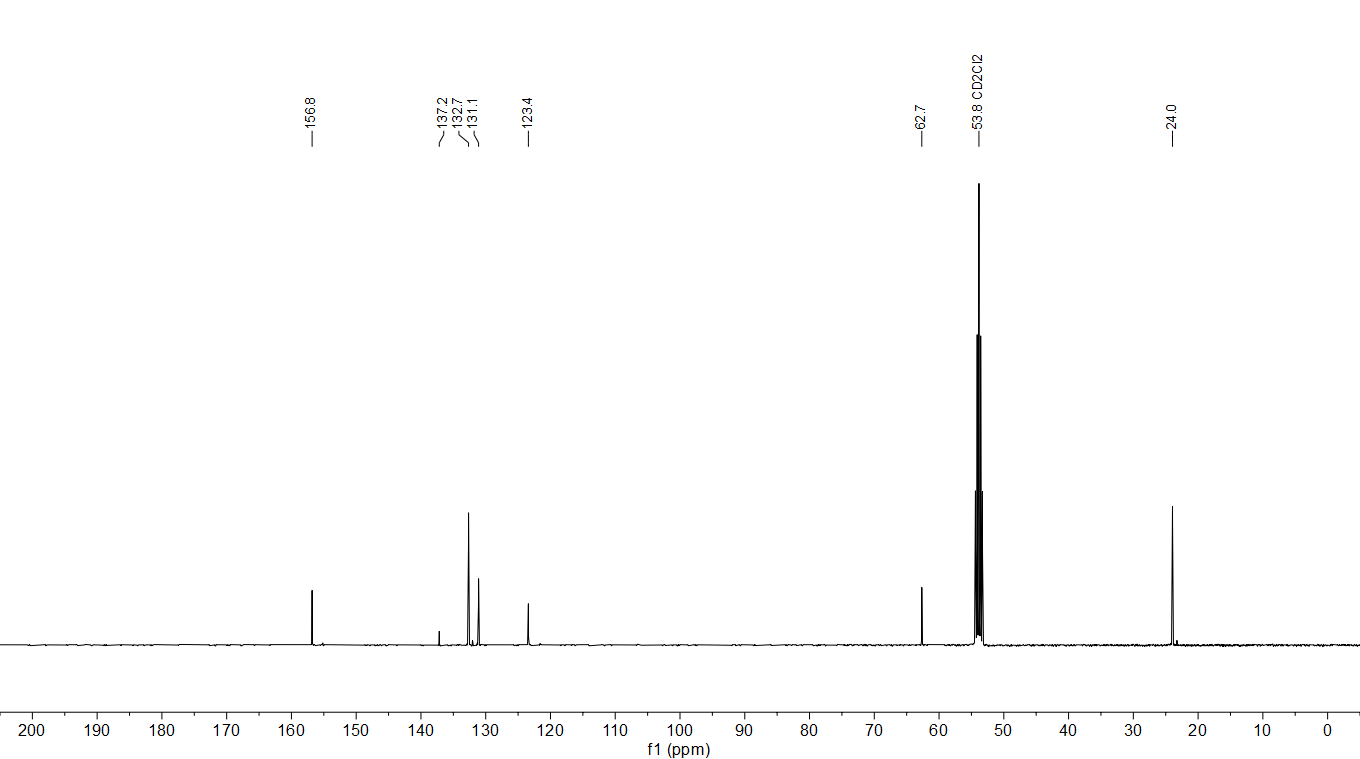


Figure S20: ^13^C{^1^H] NMR spectrum of imine **1j.**


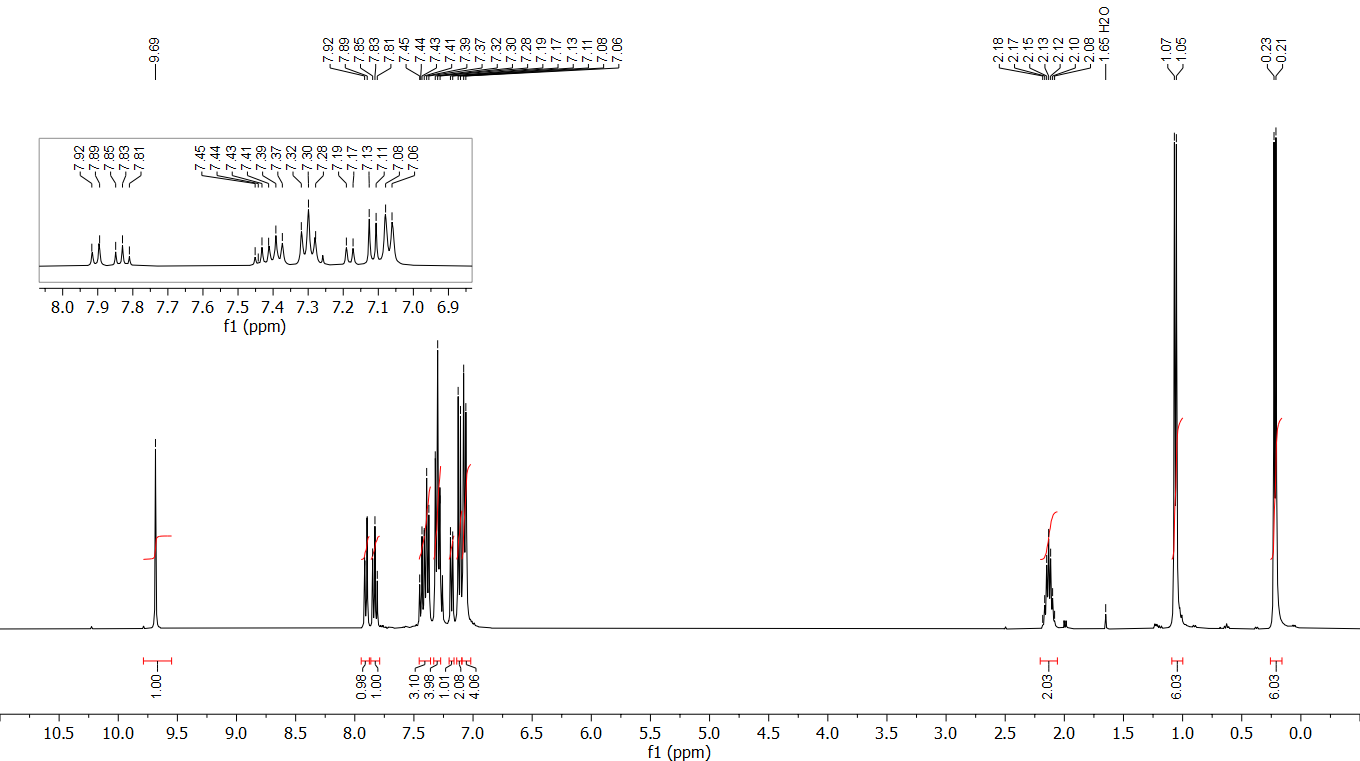


Figure S21: ^1^H NMR spectrum of isoindolium salt **2a.**


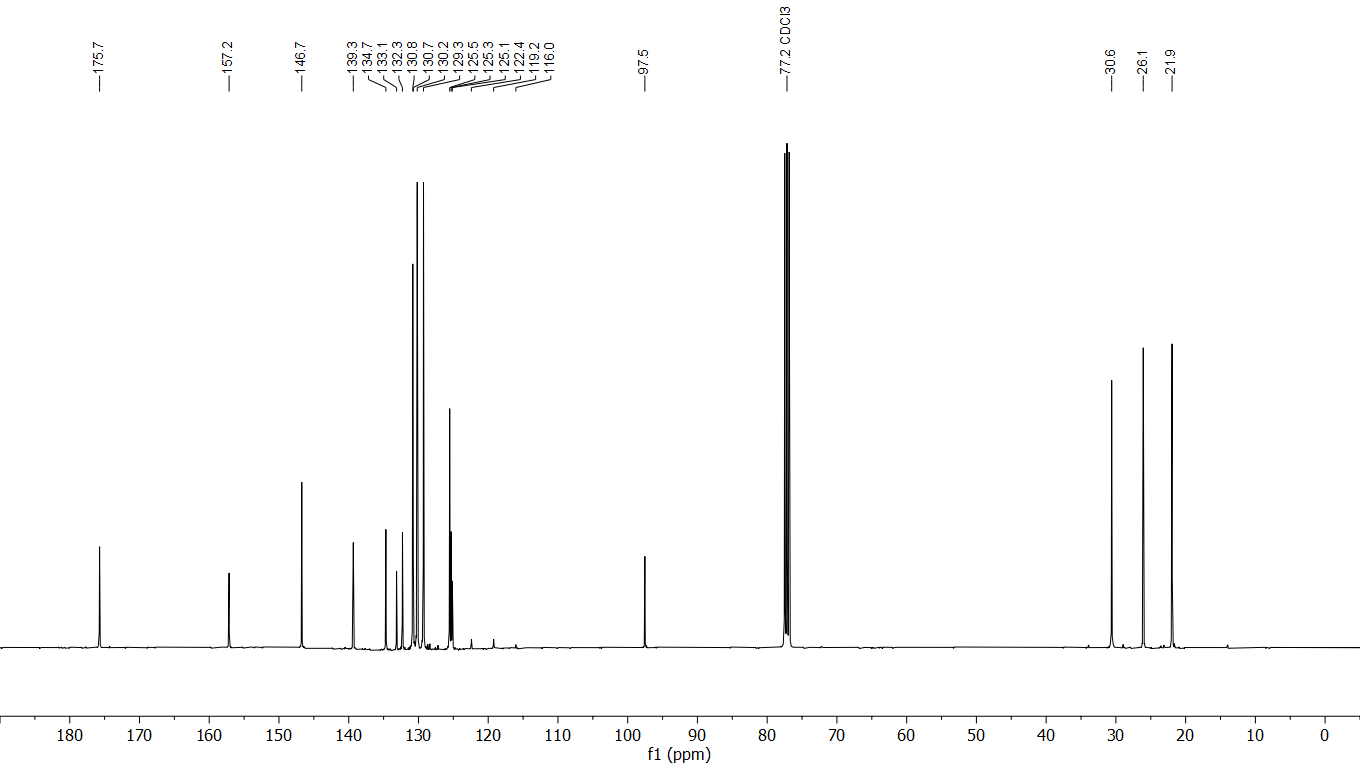


Figure S22: ^13^C{^1^H] NMR spectrum of isoindolium salt **2a.**


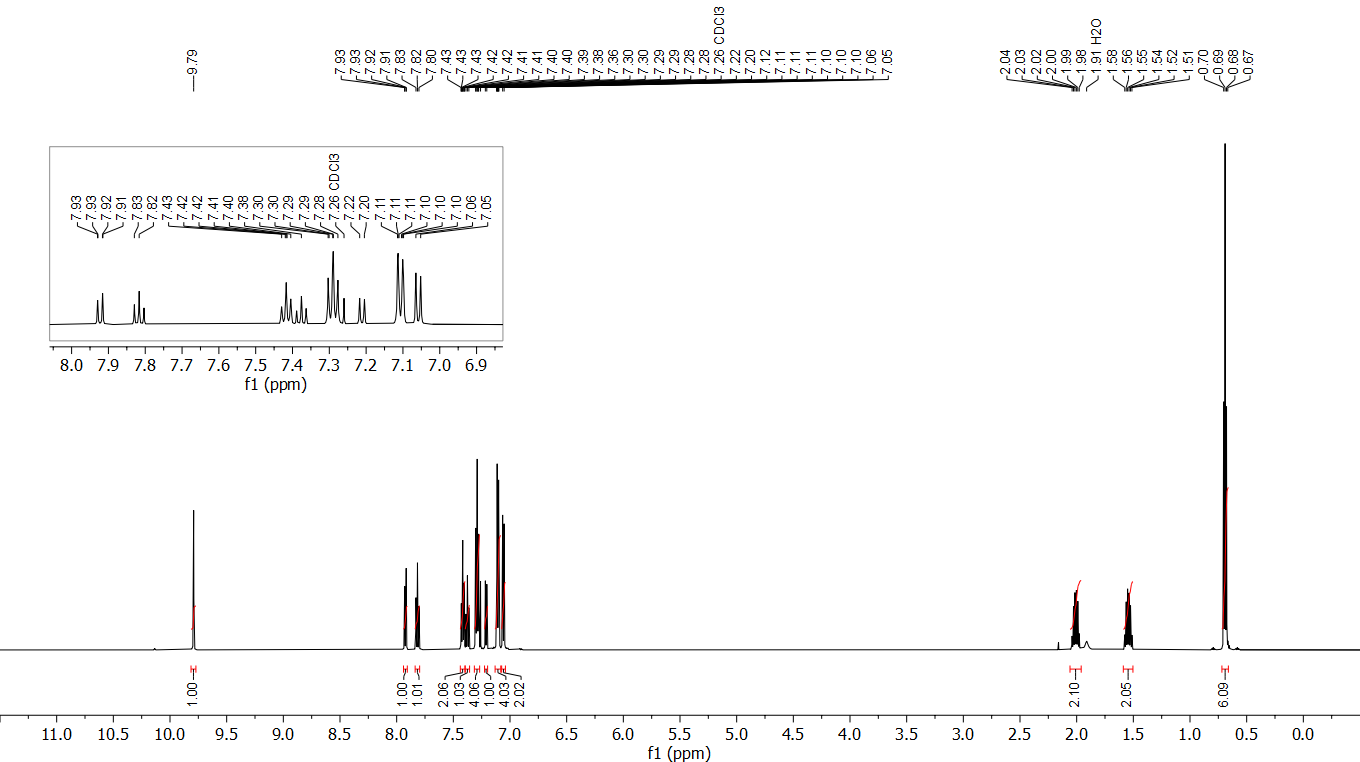


Figure S23: ^1^H NMR spectrum of isoindolium salt **2b.**


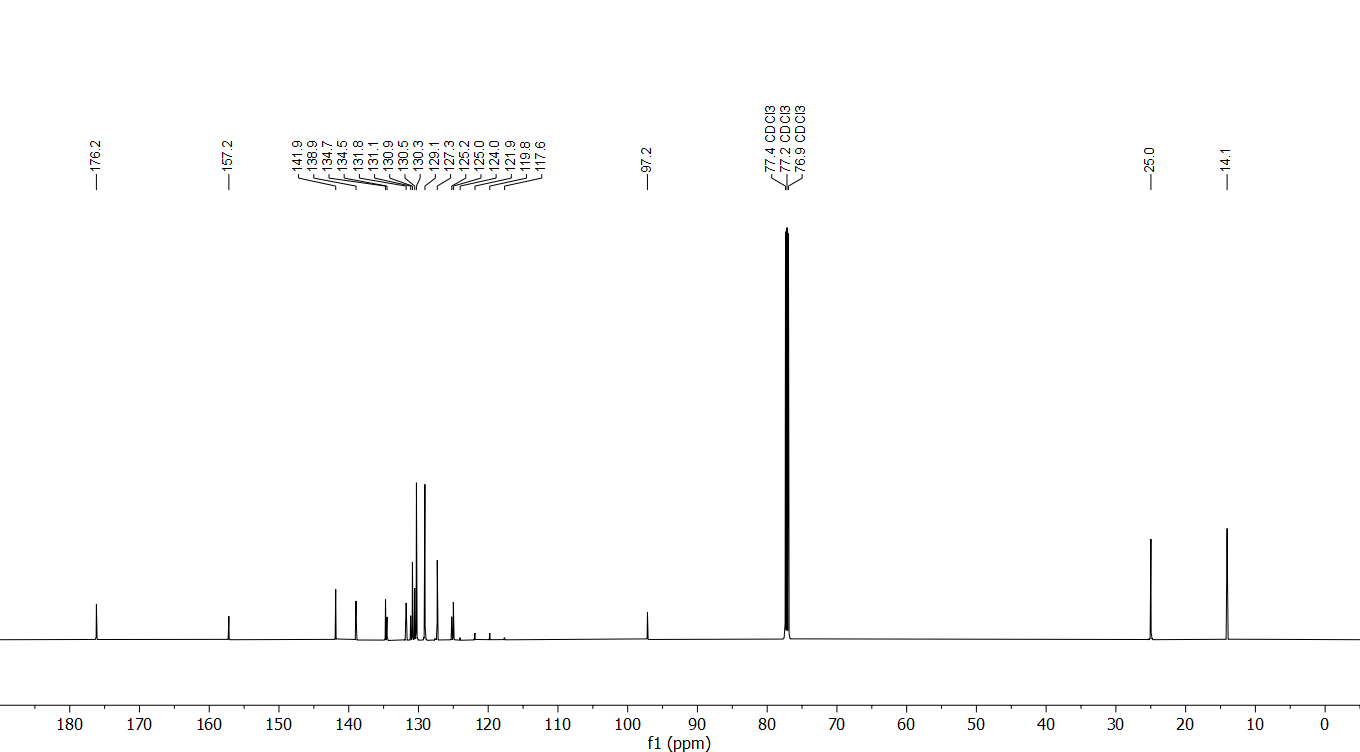


Figure S24: ^13^C{^1^H] NMR spectrum of isoindolium salt **2b.**


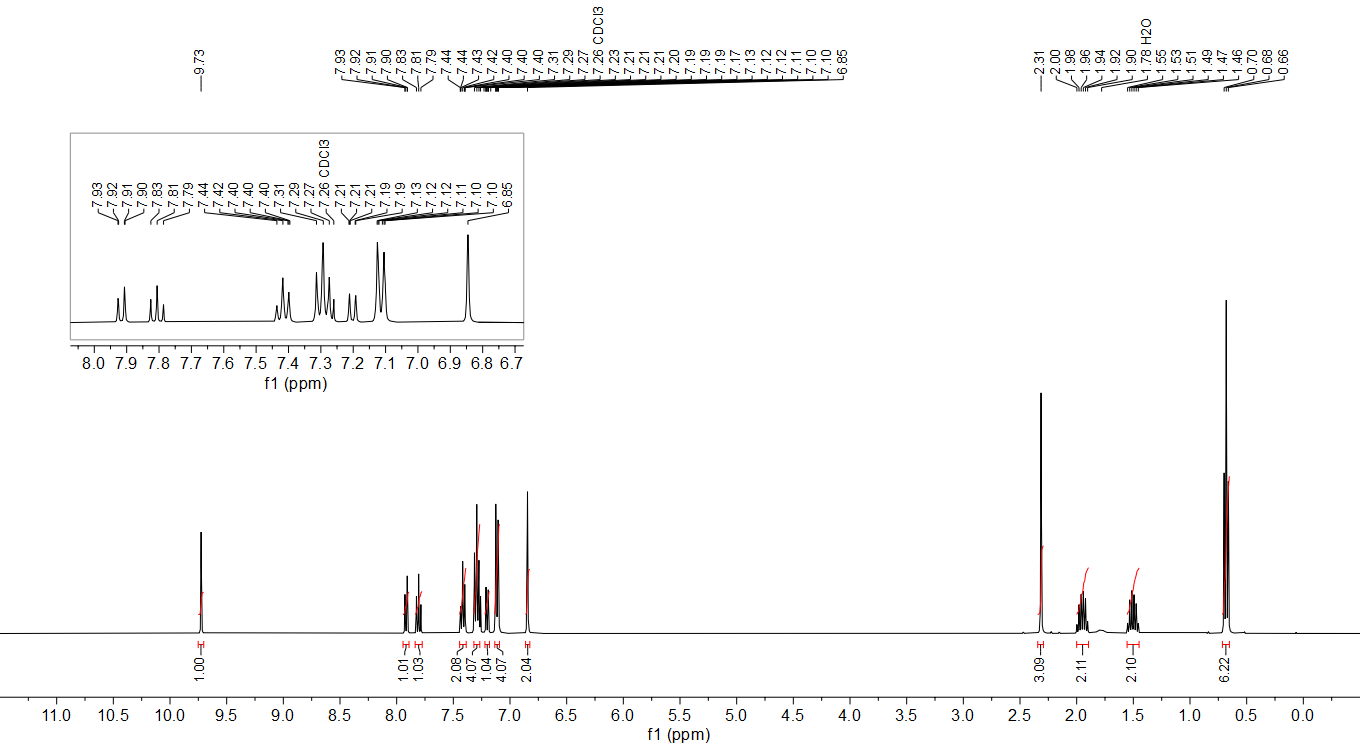


Figure S25: ^1^H NMR spectrum of isoindolium salt **2c.**


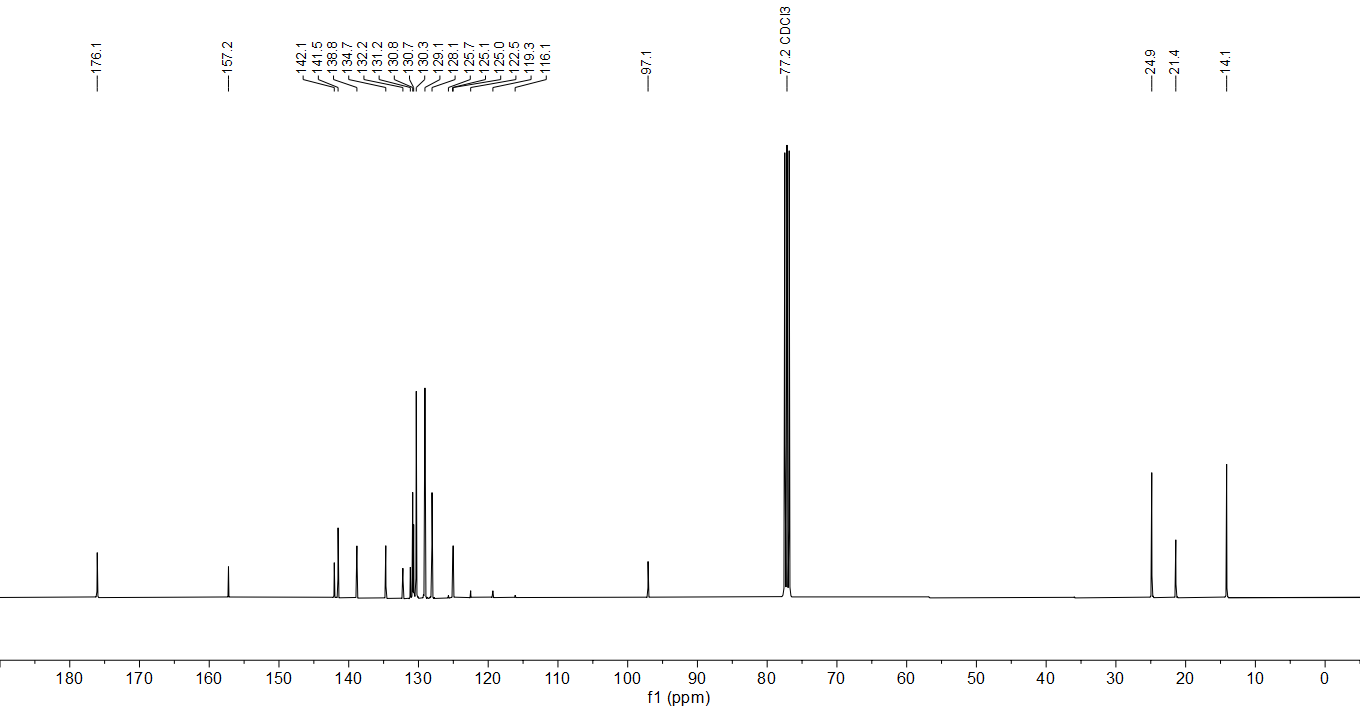


Figure S26: ^13^C{^1^H] NMR spectrum of isoindolium salt **2c.**


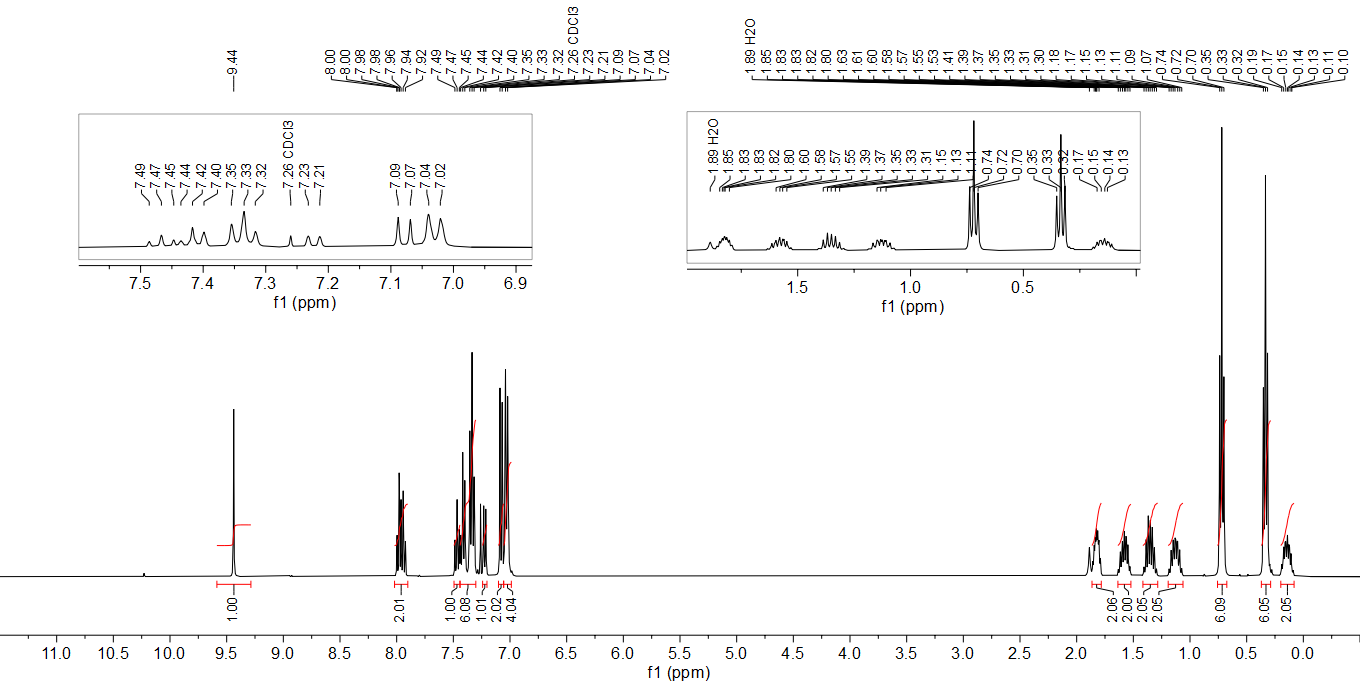


Figure S27: ^1^H NMR spectrum of isoindolium salt **2d.**


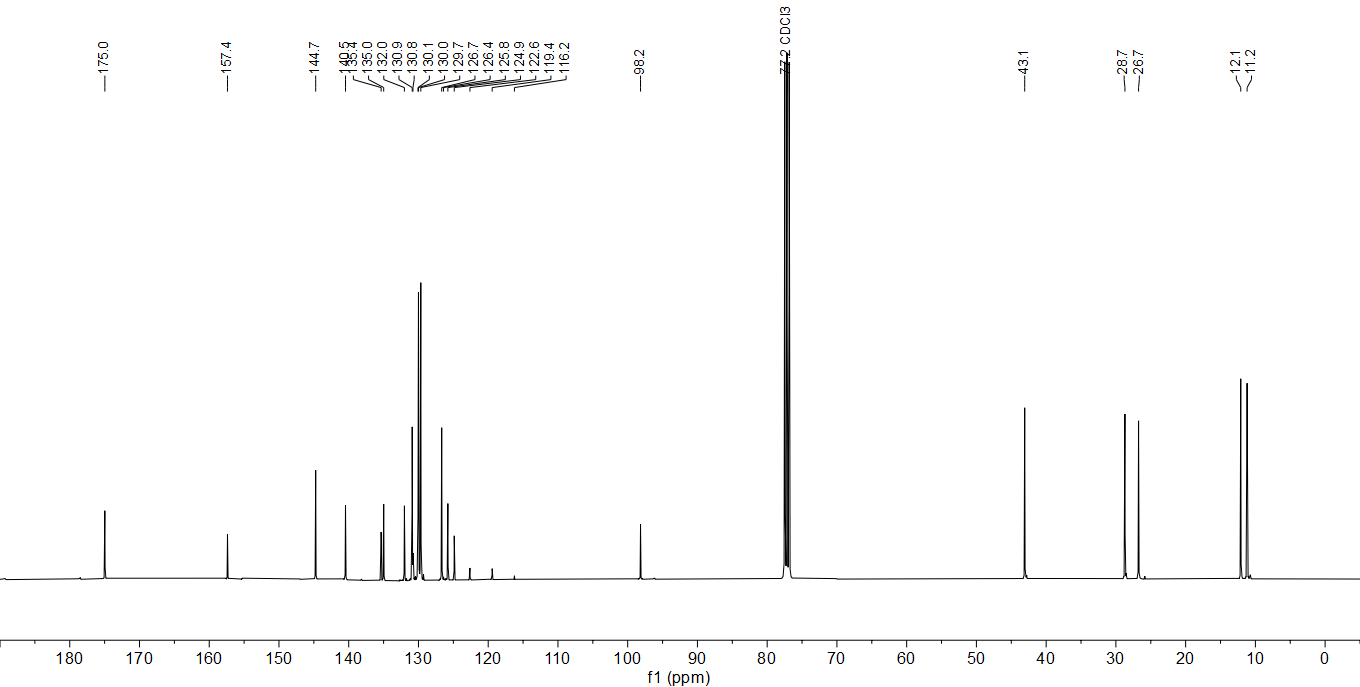


Figure S28: ^13^C{^1^H] NMR spectrum of isoindolium salt **2d.**


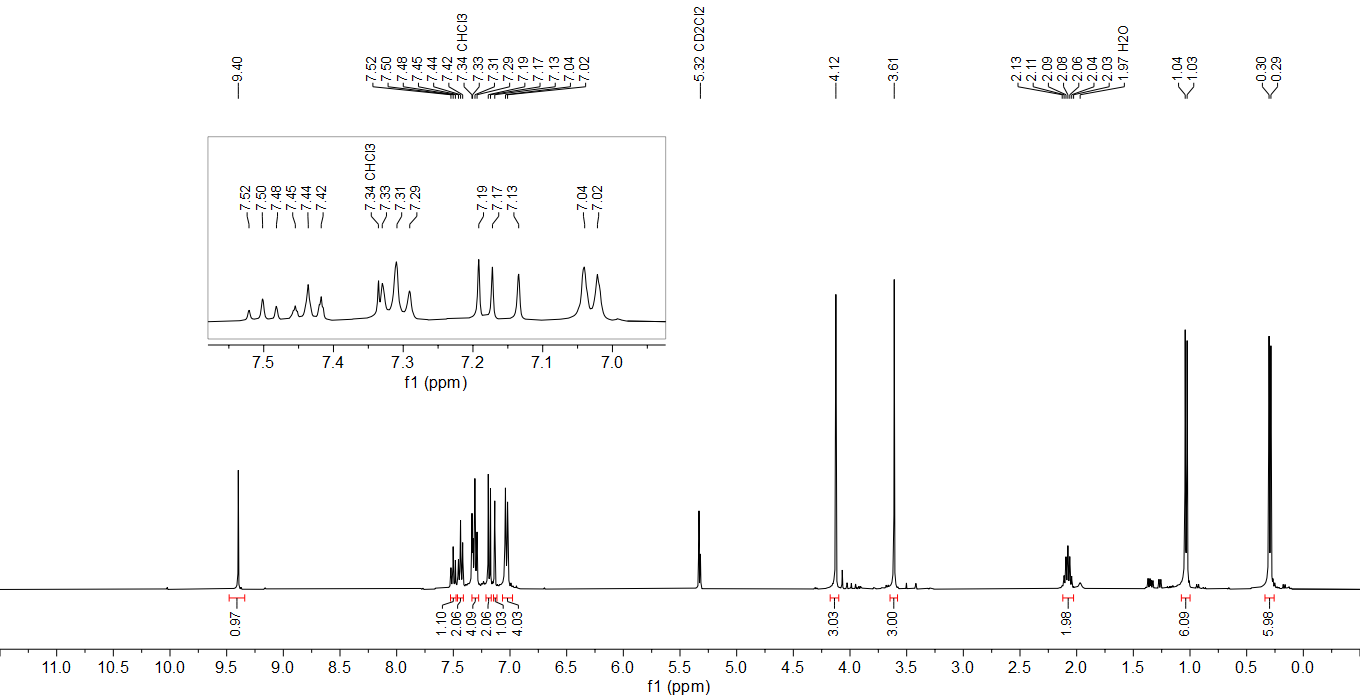


Figure S29: ^1^H NMR spectrum of isoindolium salt **2f.**


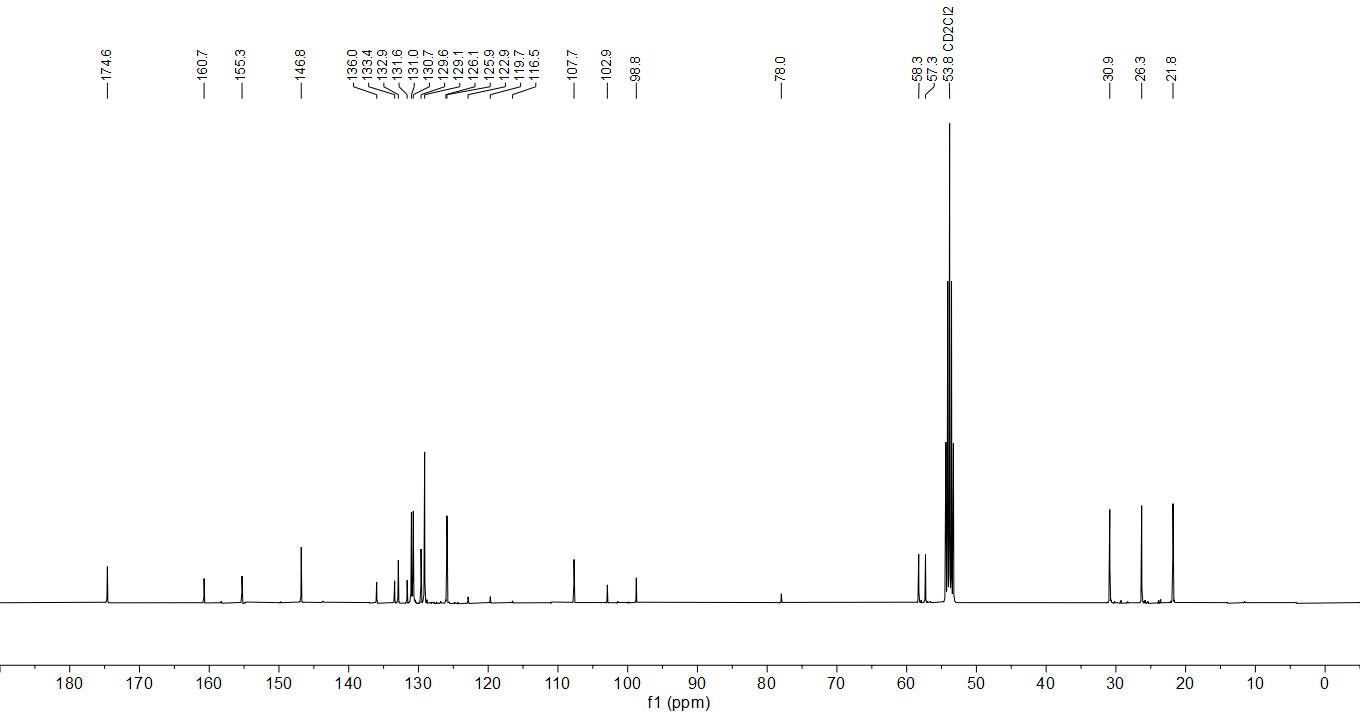


Figure S30: ^13^C{^1^H] NMR spectrum of isoindolium salt **2f.**


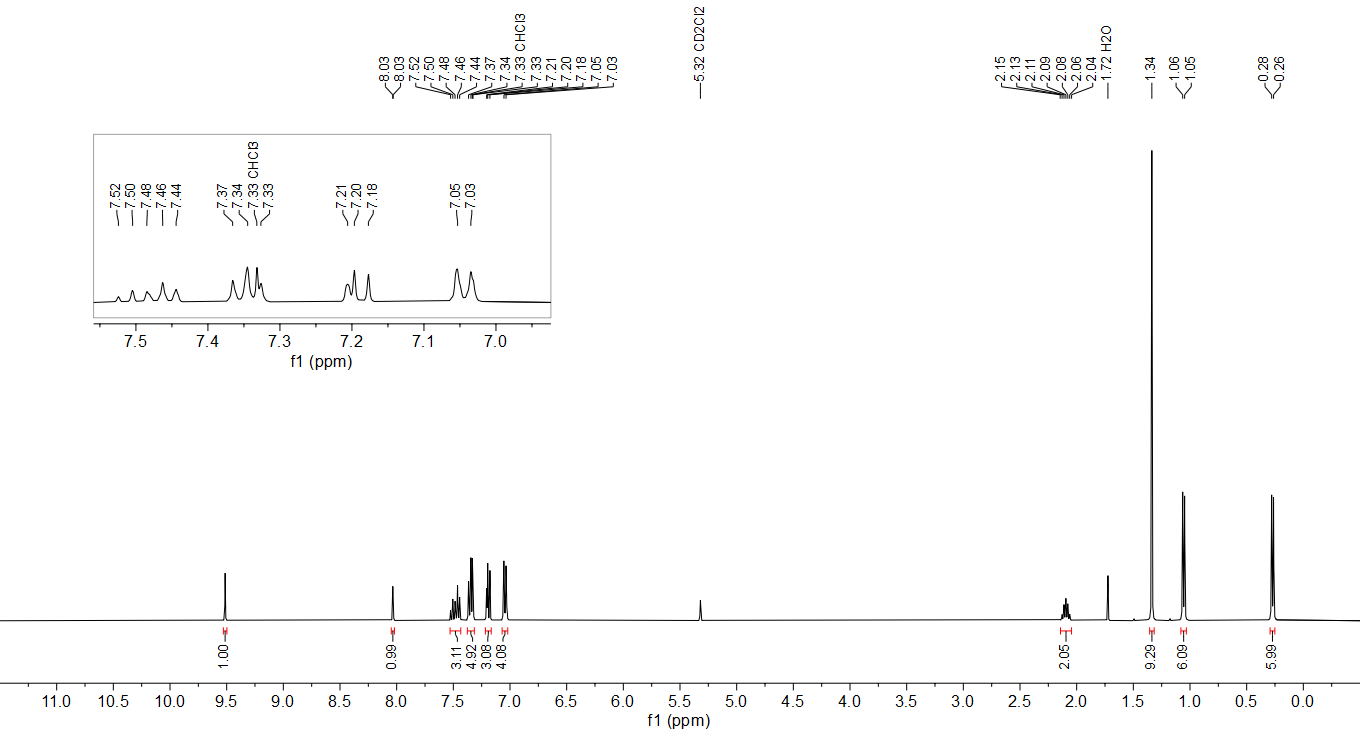


Figure S31: ^1^H NMR spectrum of isoindolium salt **2g.**


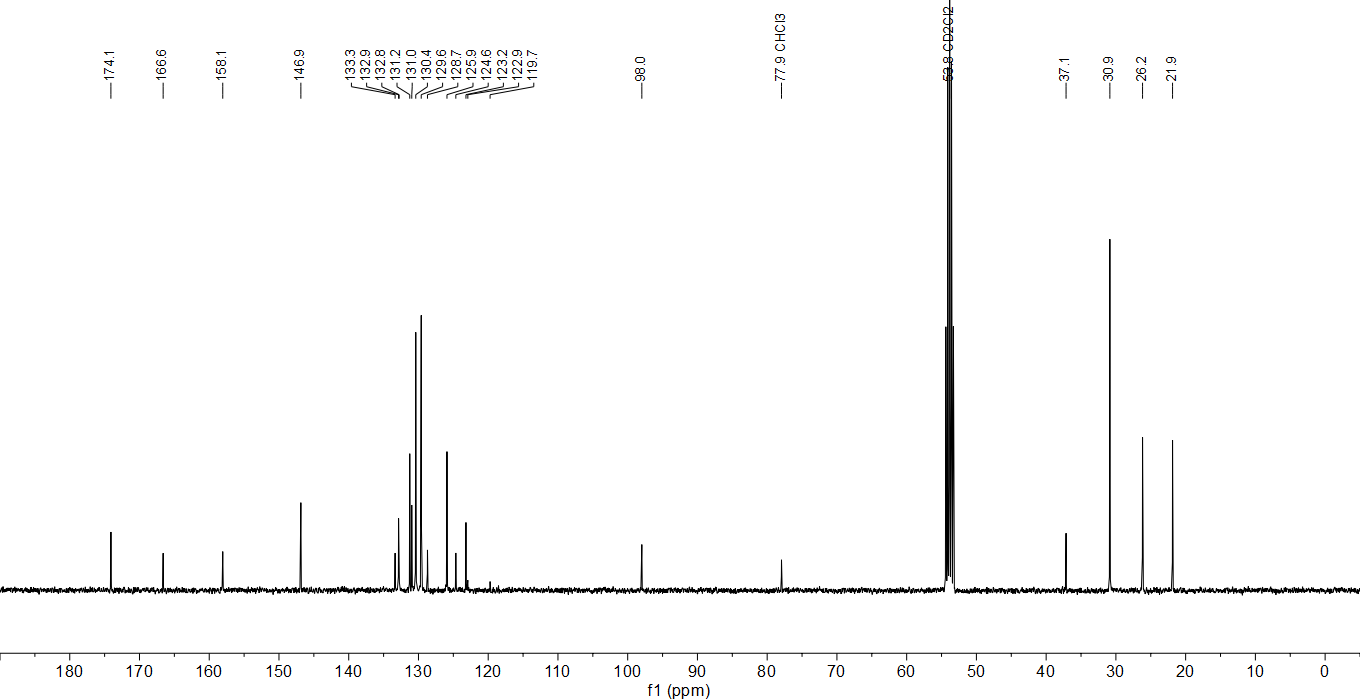


Figure S32: ^13^C{^1^H] NMR spectrum of isoindolium salt **2g.**


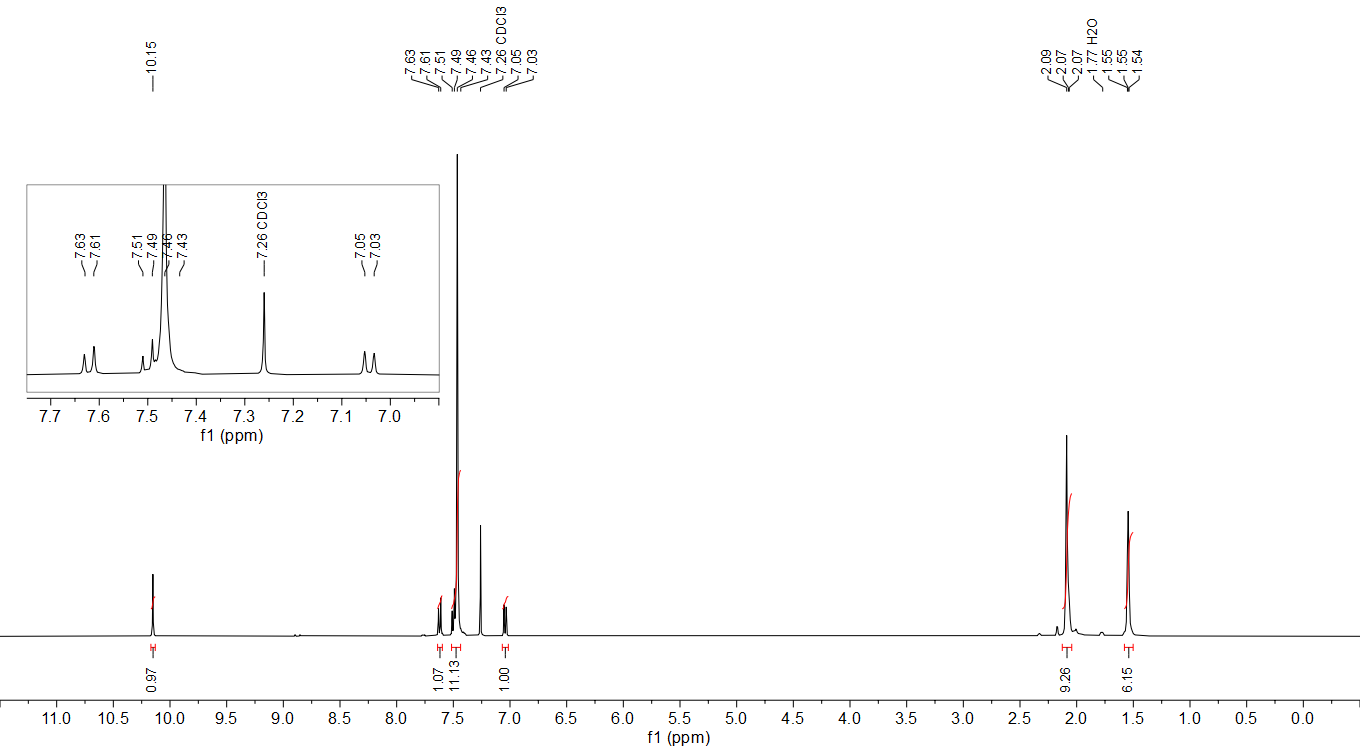


Figure S33: ^1^H NMR spectrum of isoindolium salt **2h.**


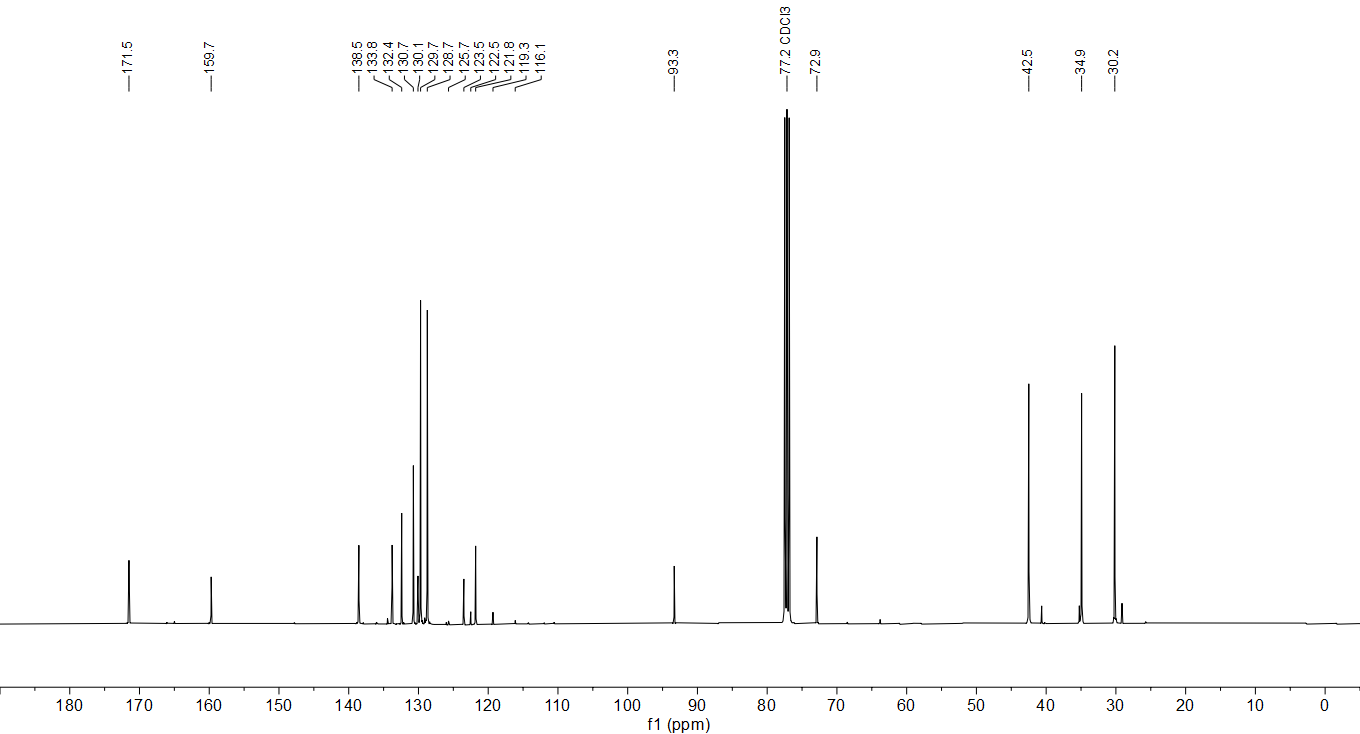


Figure S34: ^13^C{^1^H] NMR spectrum of isoindolium salt **2h.**


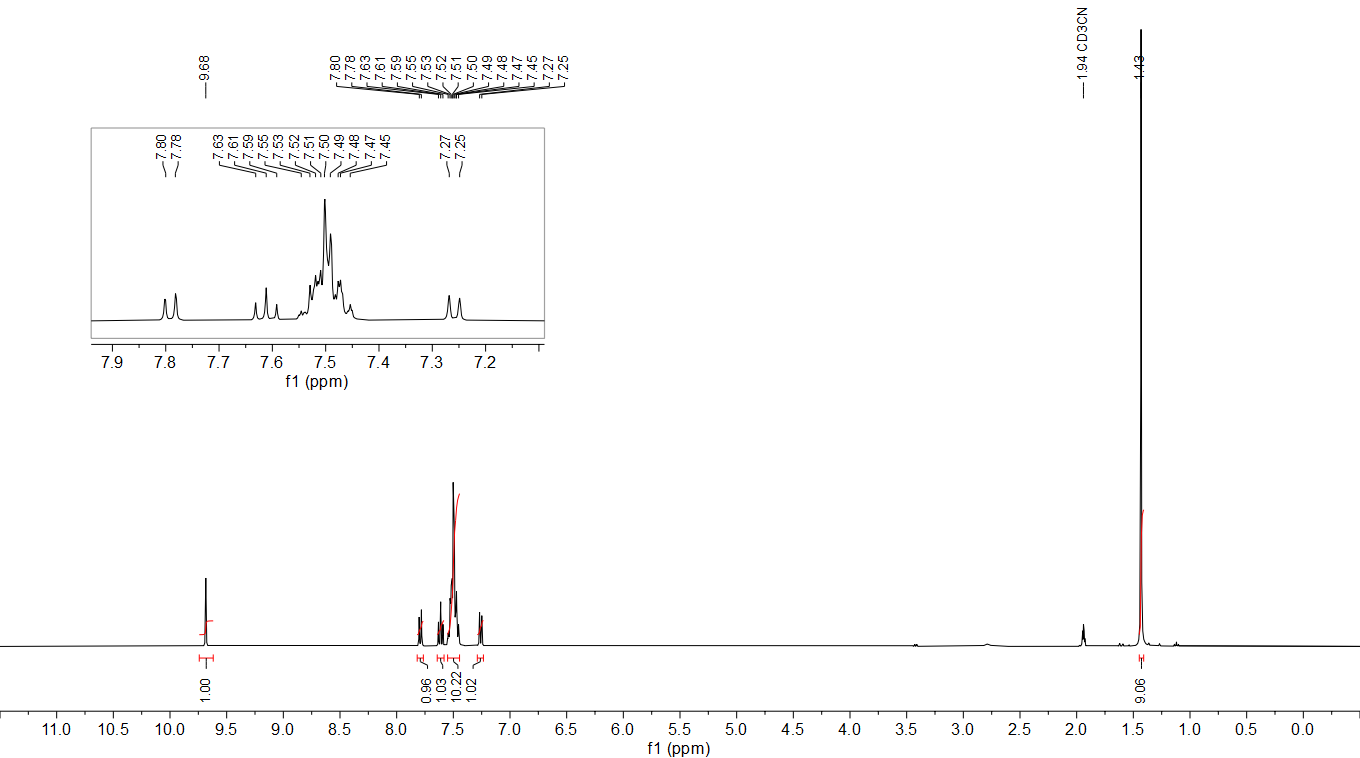


Figure S35: ^1^H NMR spectrum of isoindolium salt **2i.**


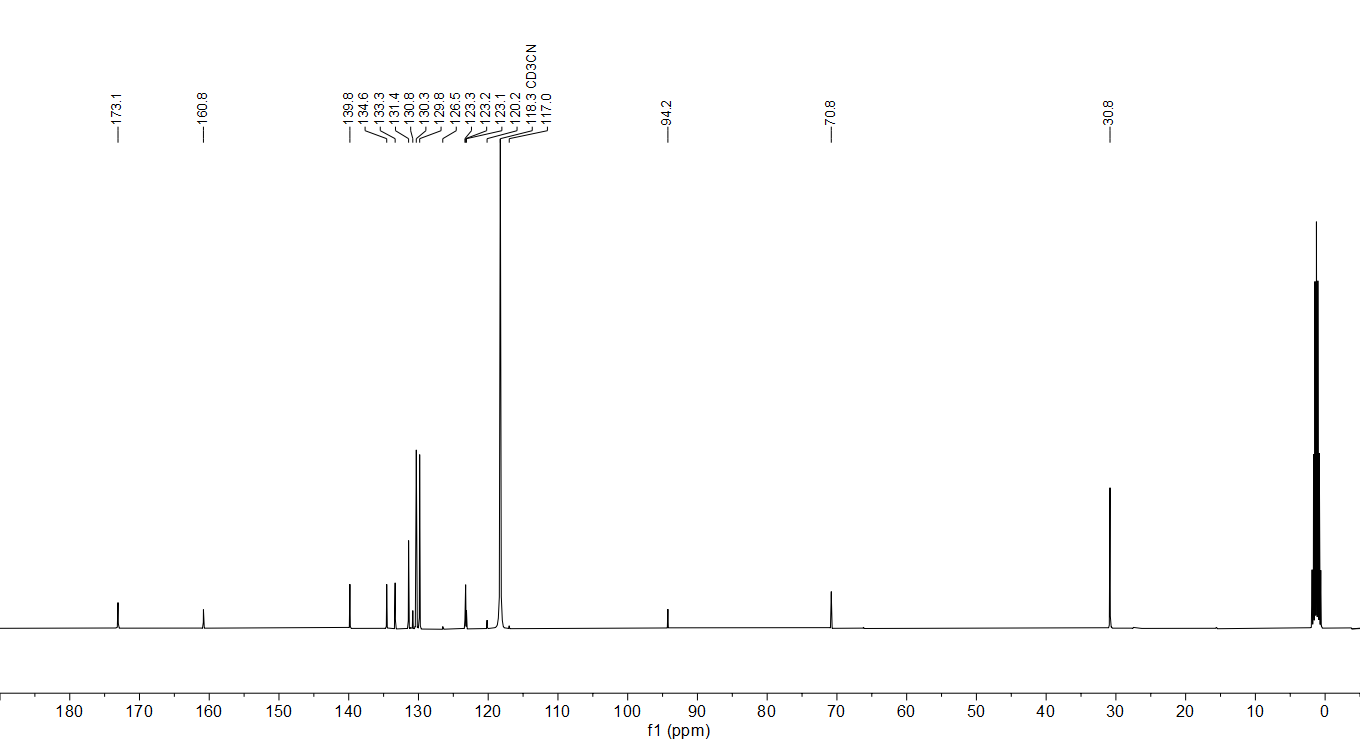


Figure S36: ^13^C{^1^H] NMR spectrum of isoindolium salt **2i.**


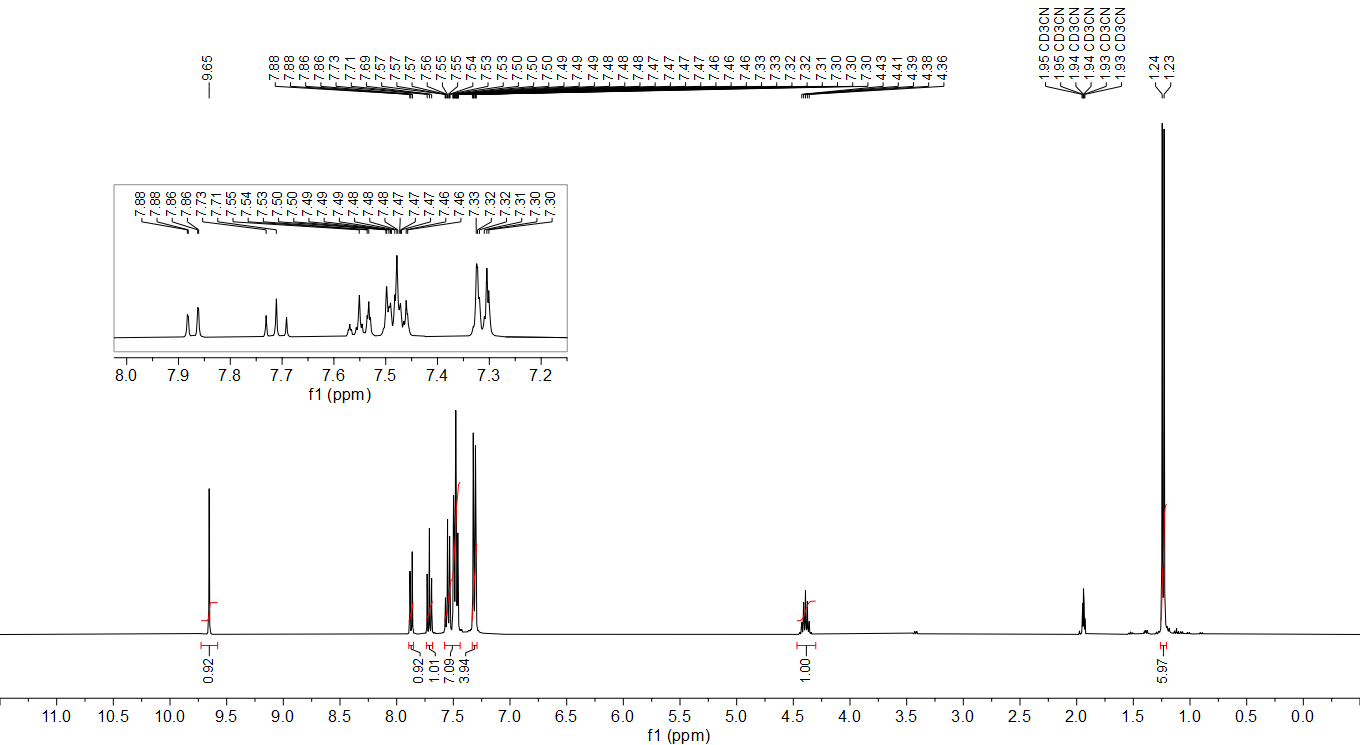


Figure S37: ^1^H NMR spectrum of isoindolium salt **2j.**


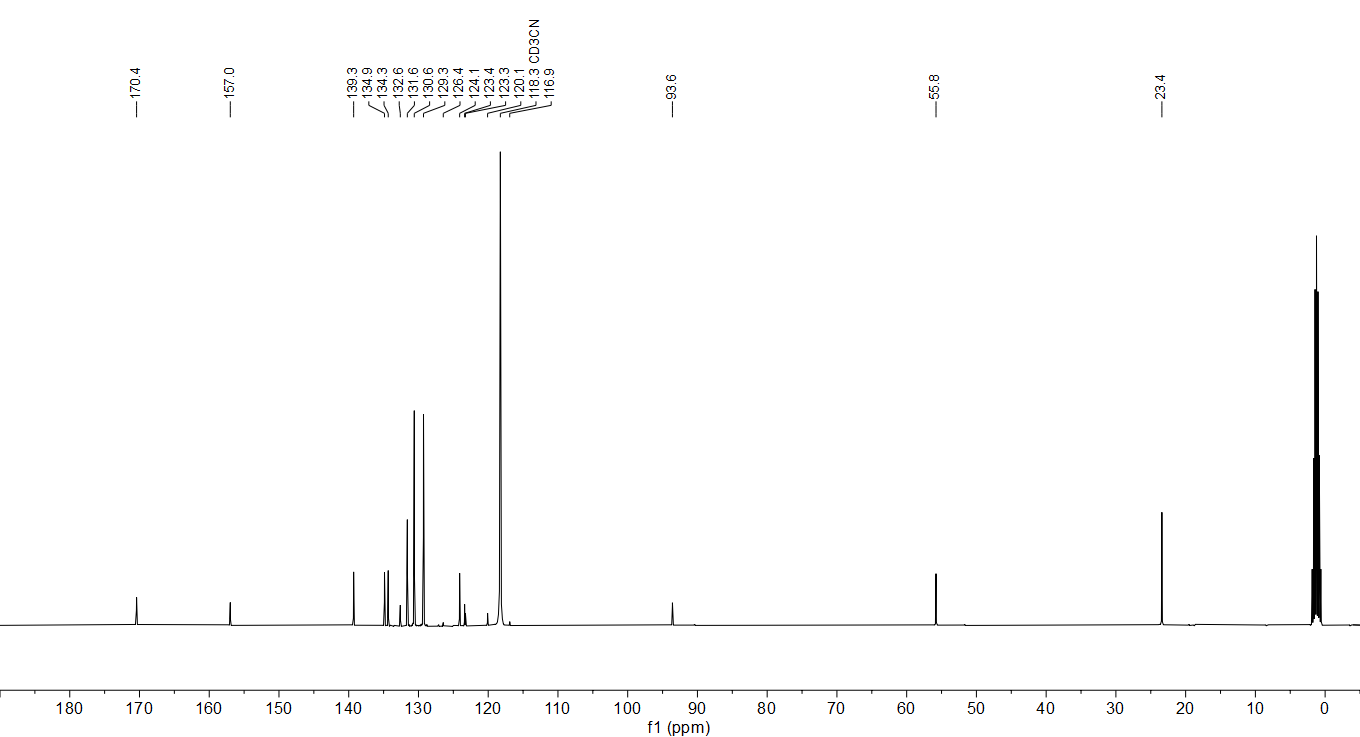


Figure S38: ^13^C{^1^H] NMR spectrum of isoindolium salt **2j.**


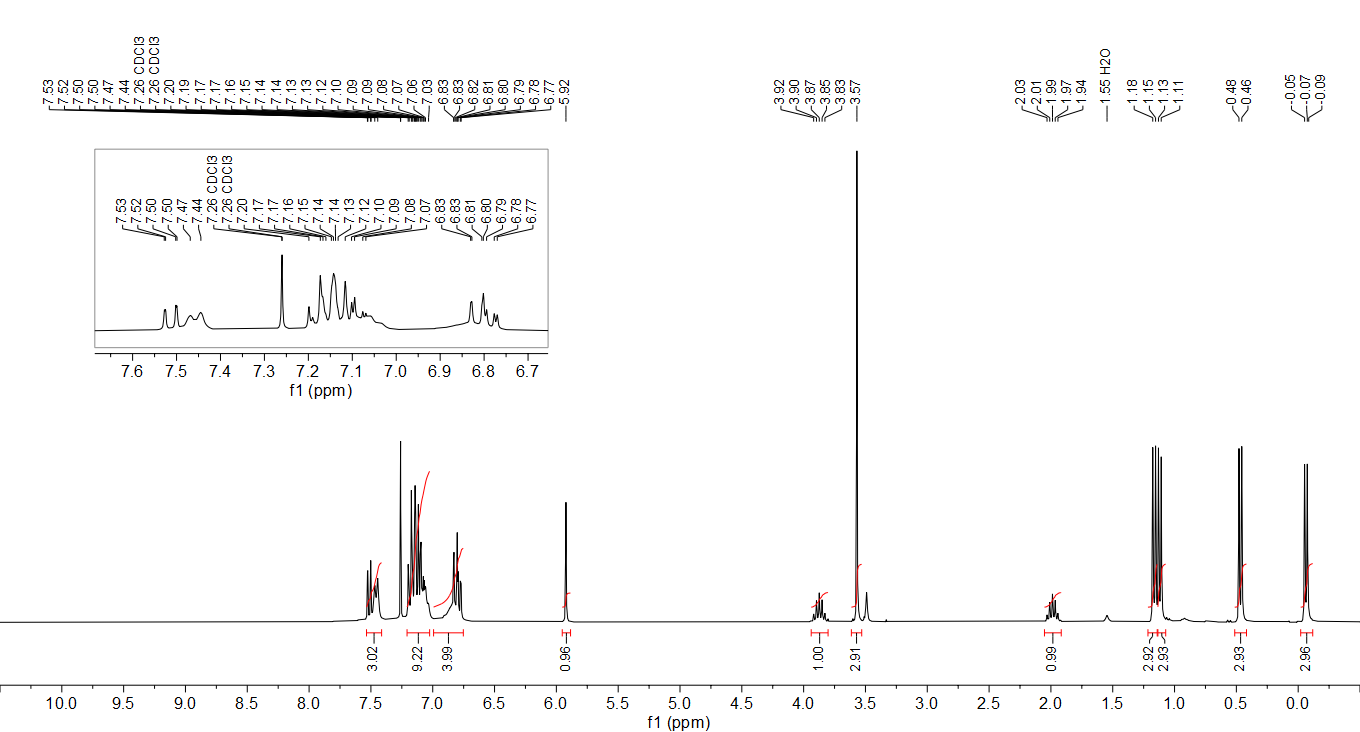


Figure S39: ^1^H NMR spectrum of methoxyisoindoline **3a.**


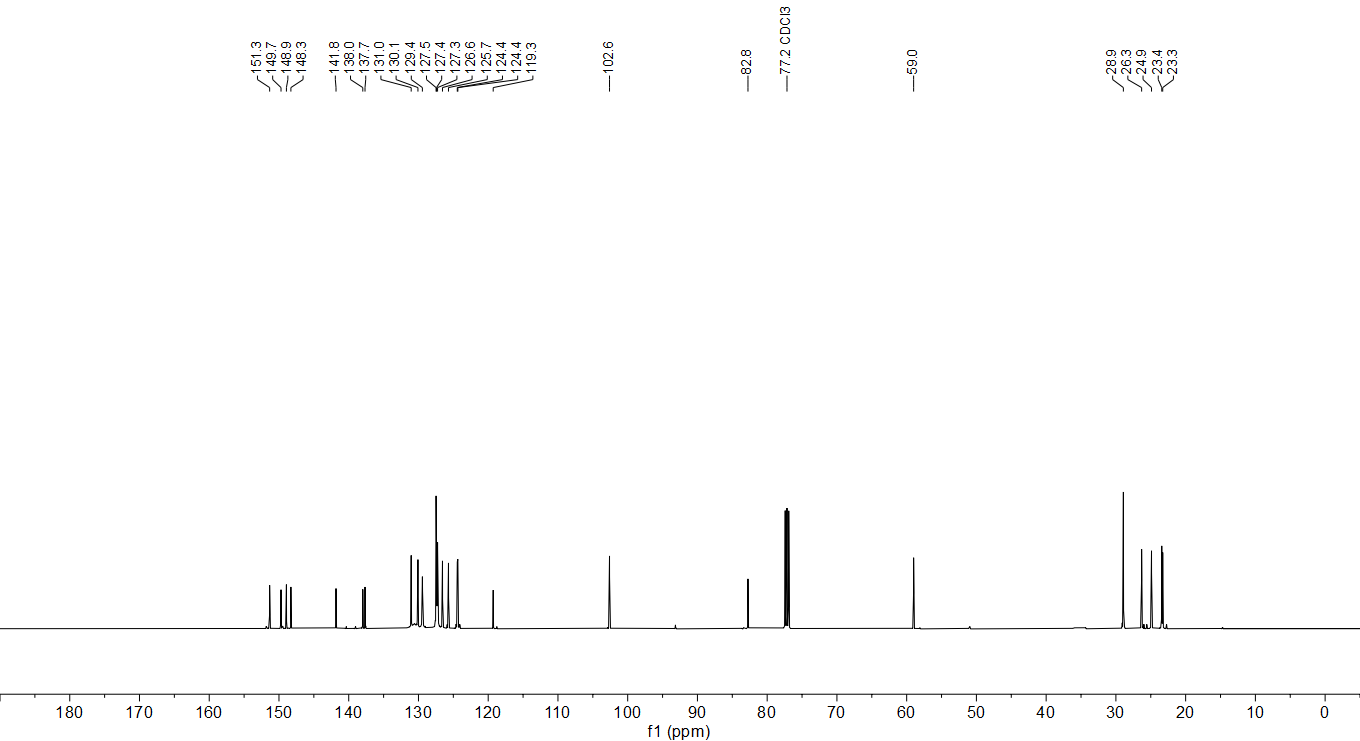


Figure S40: ^13^C{^1^H} NMR spectrum of compound **3a**.


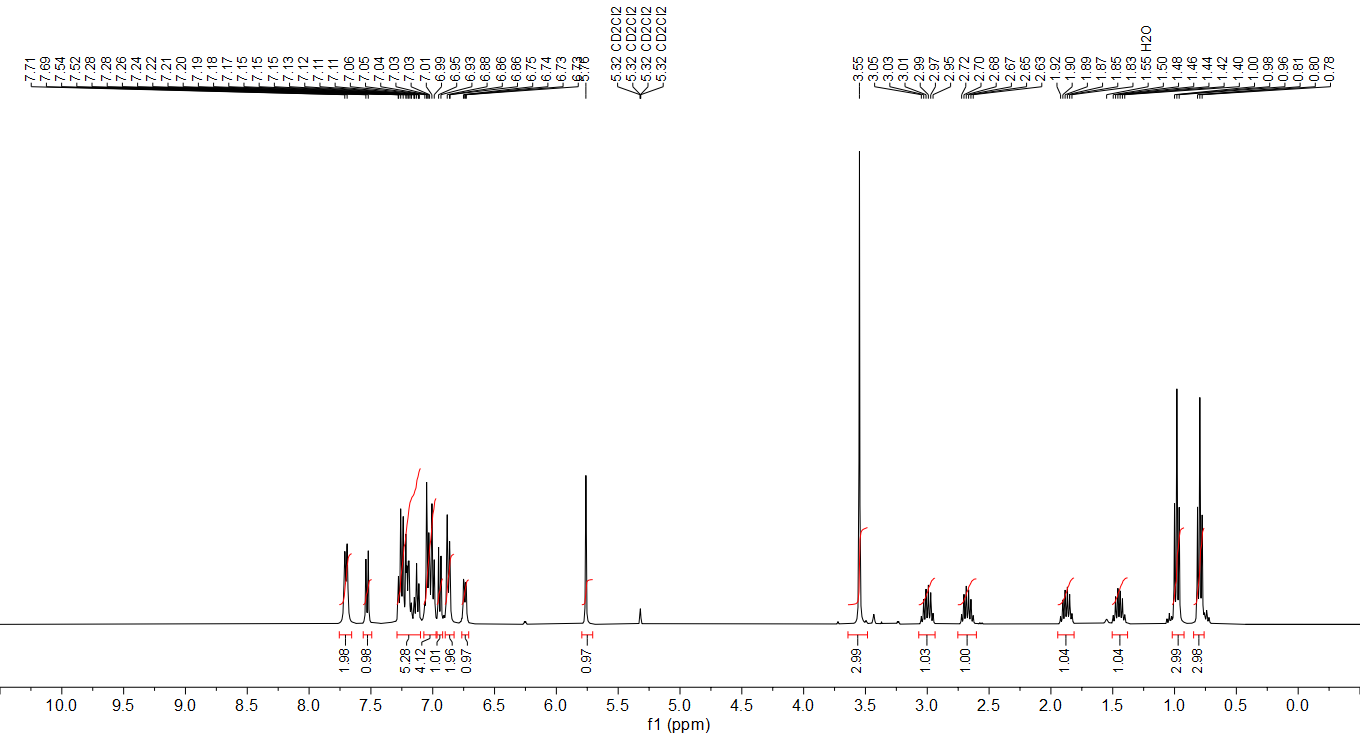


Figure S41: ^1^H NMR spectrum of methoxyisoindoline **3b.**


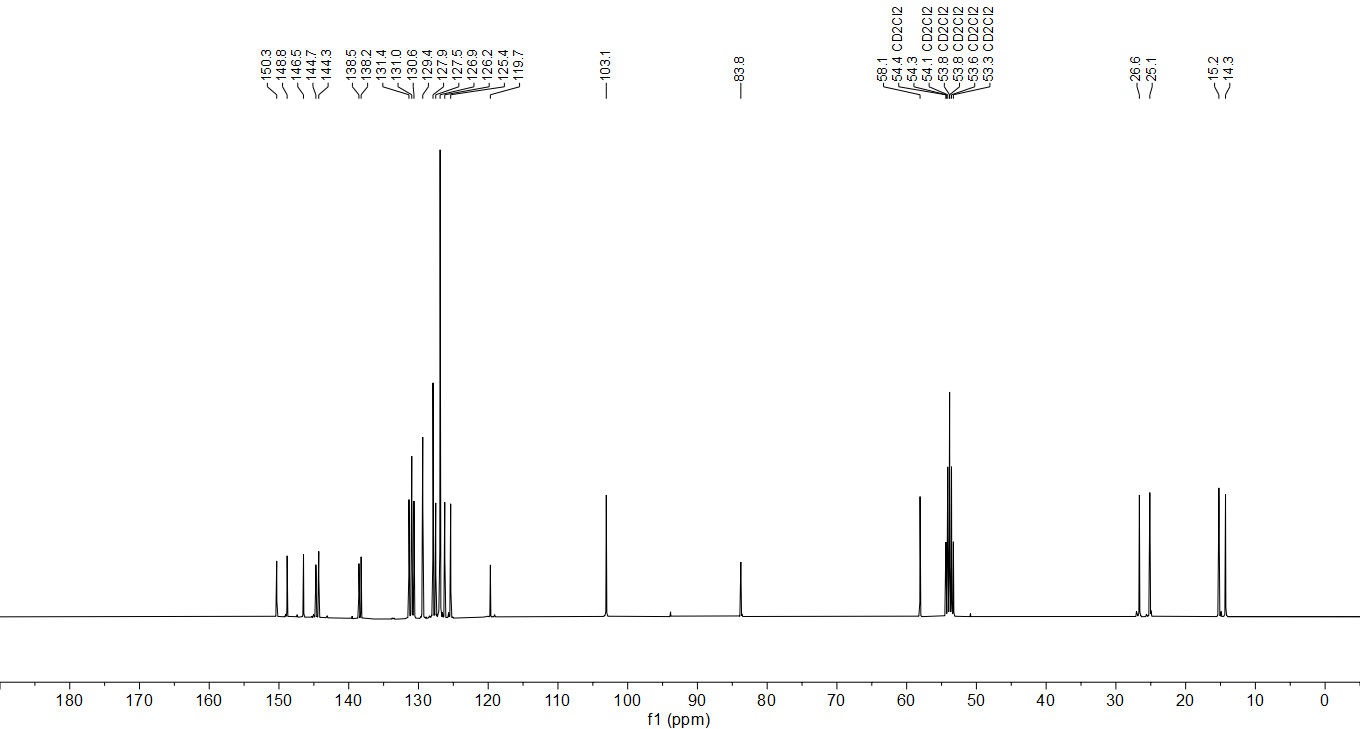


Figure S42: ^13^C{^1^H} NMR spectrum of compound **3b**.


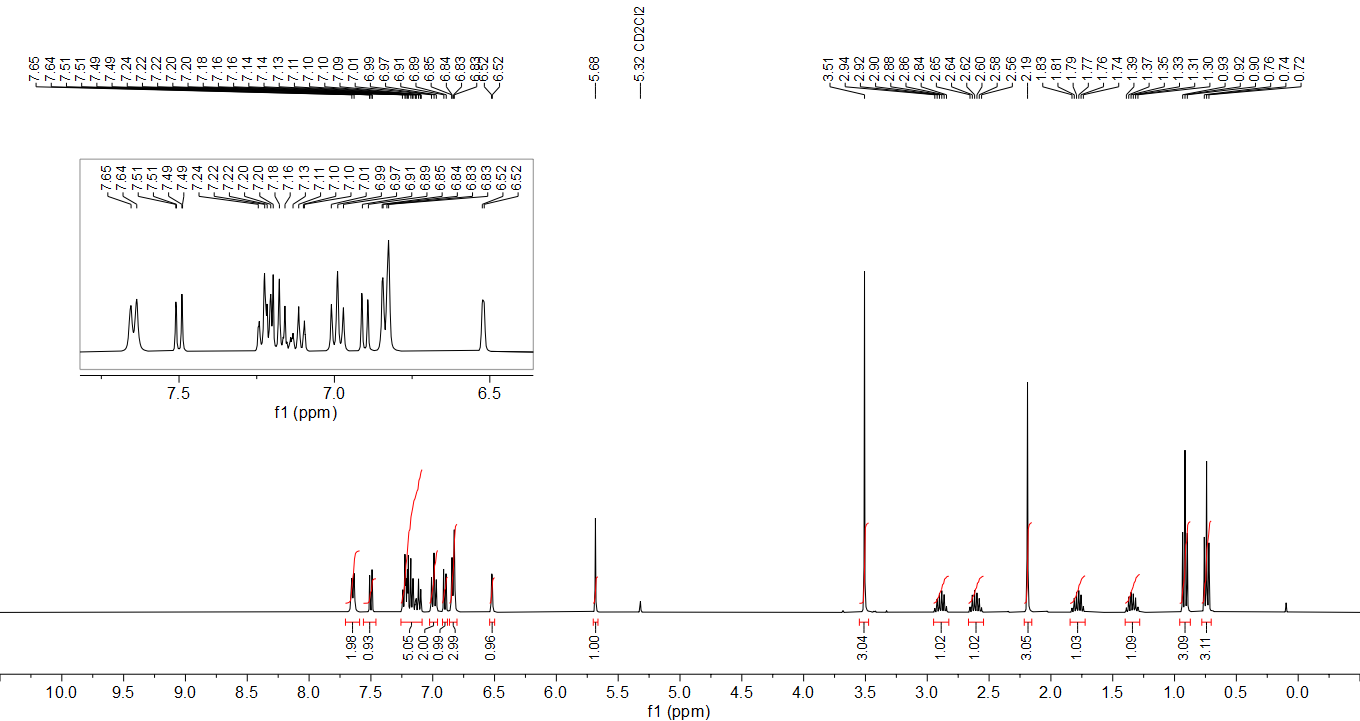


Figure S43: ^1^H NMR spectrum of methoxyisoindoline **3c**.


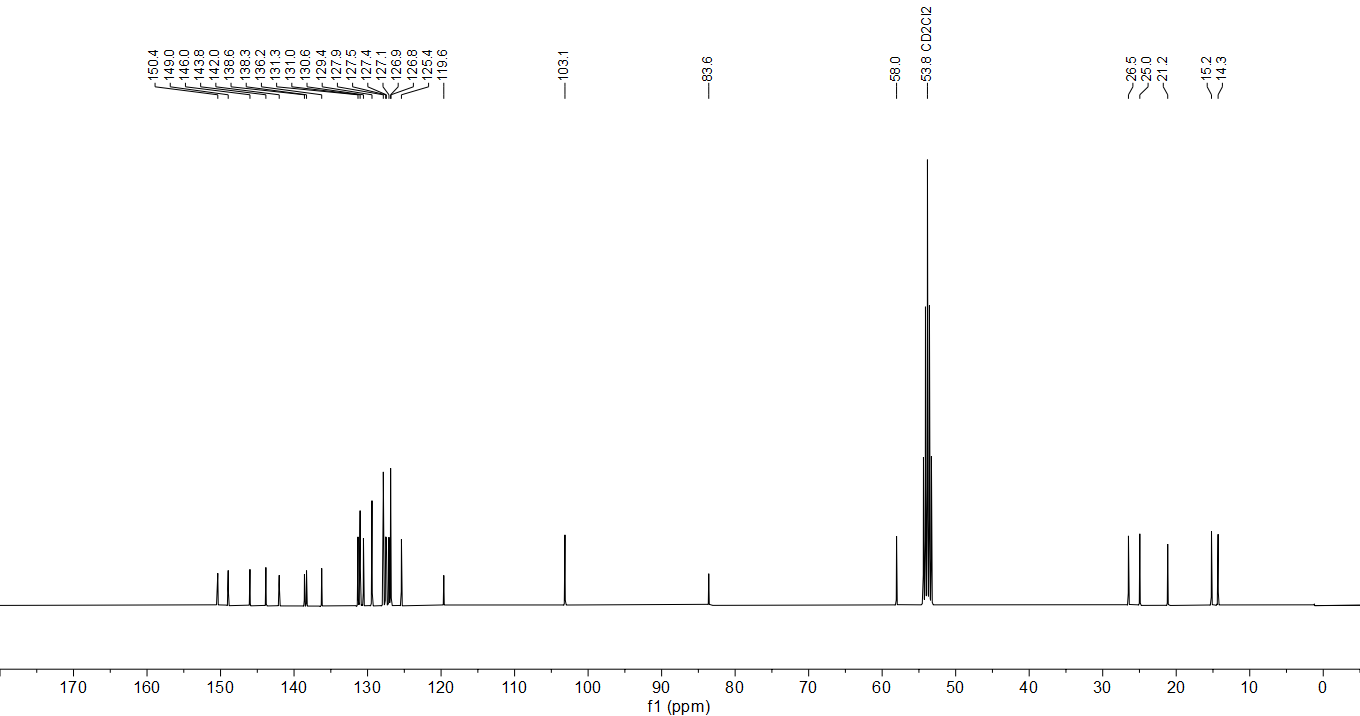


Figure S44: ^13^C{^1^H} NMR spectrum of compound **3c**.


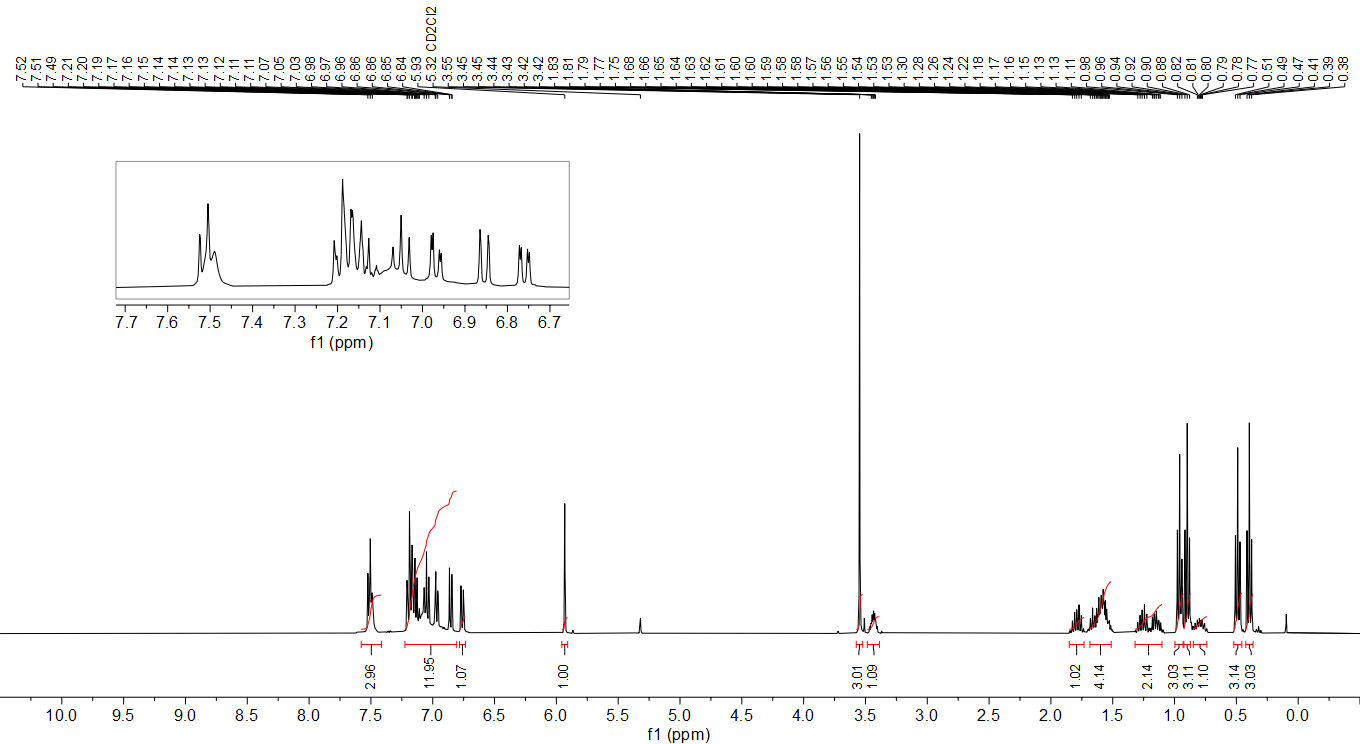


Figure S45: ^1^H NMR spectrum of methoxyisoindoline **3d**.


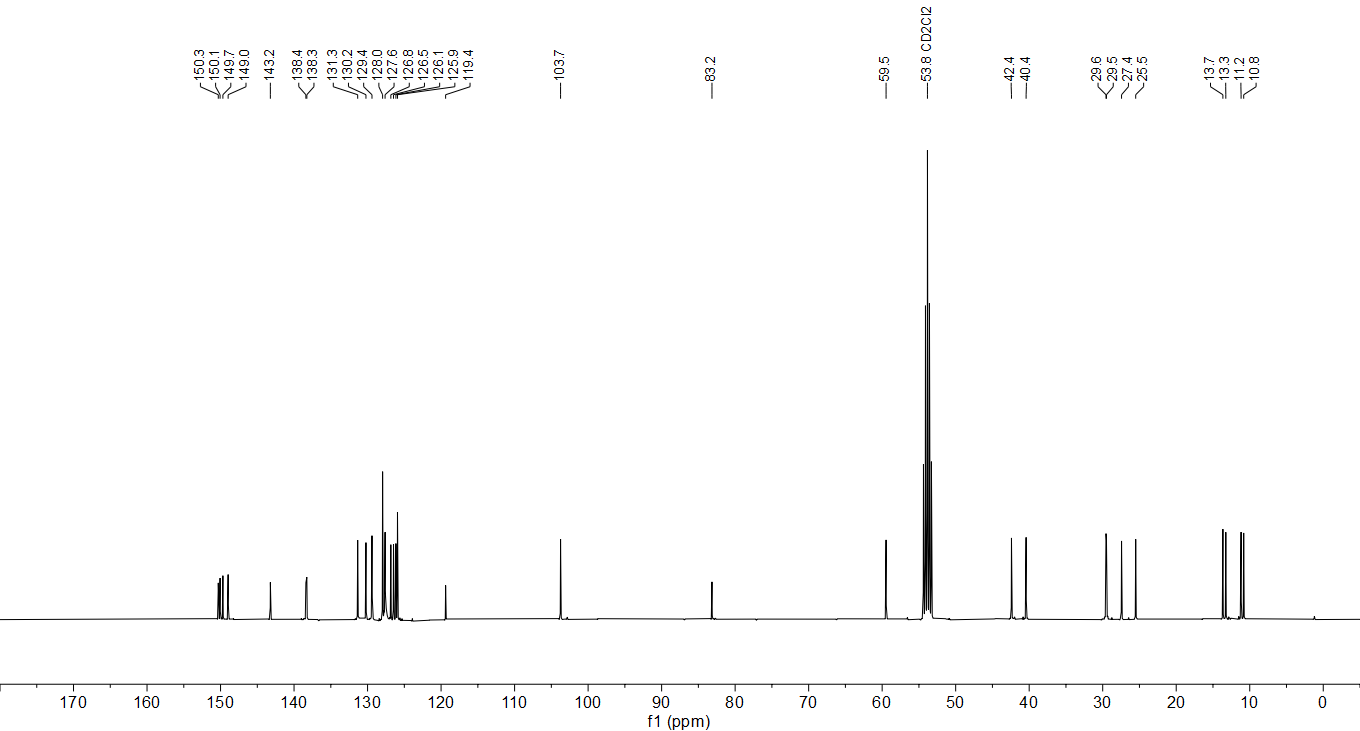


Figure S46: ^13^C{^1^H} NMR spectrum of compound **3d**.


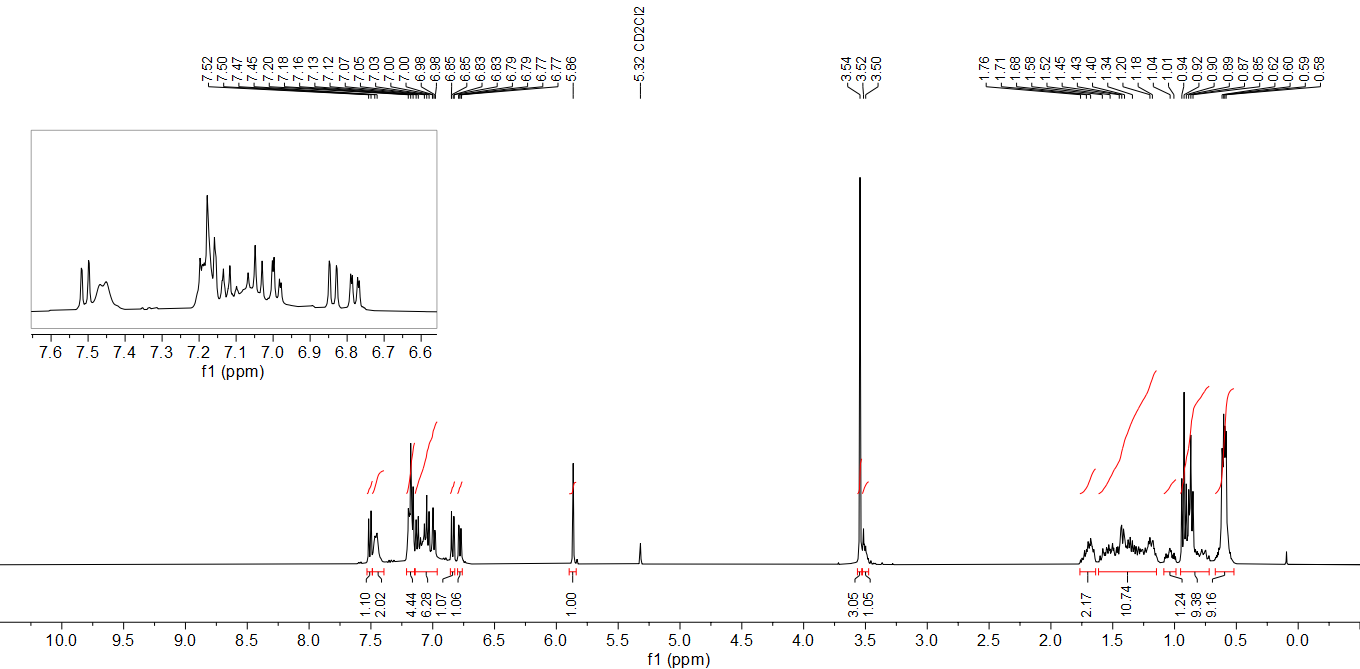


Figure S47: ^1^H NMR spectrum of methoxyisoindoline **3e**.


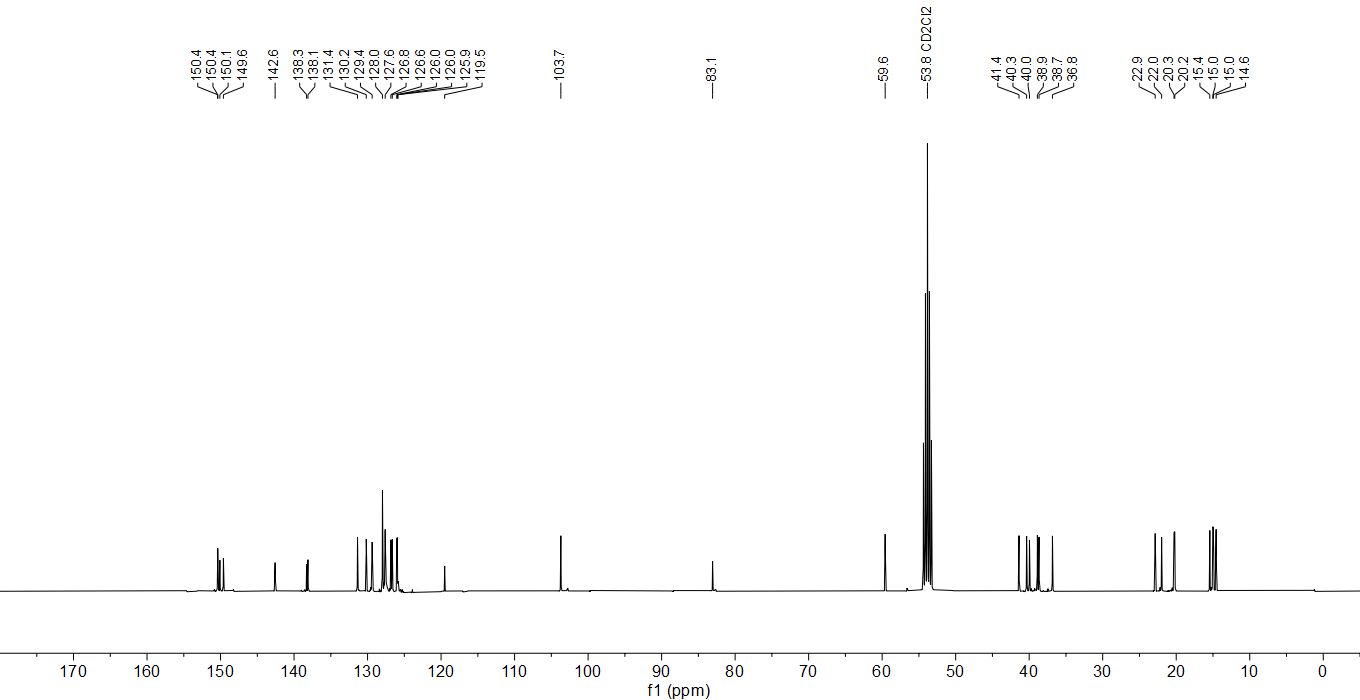


Figure S48: ^13^C{^1^H} NMR spectrum of compound **3e**.


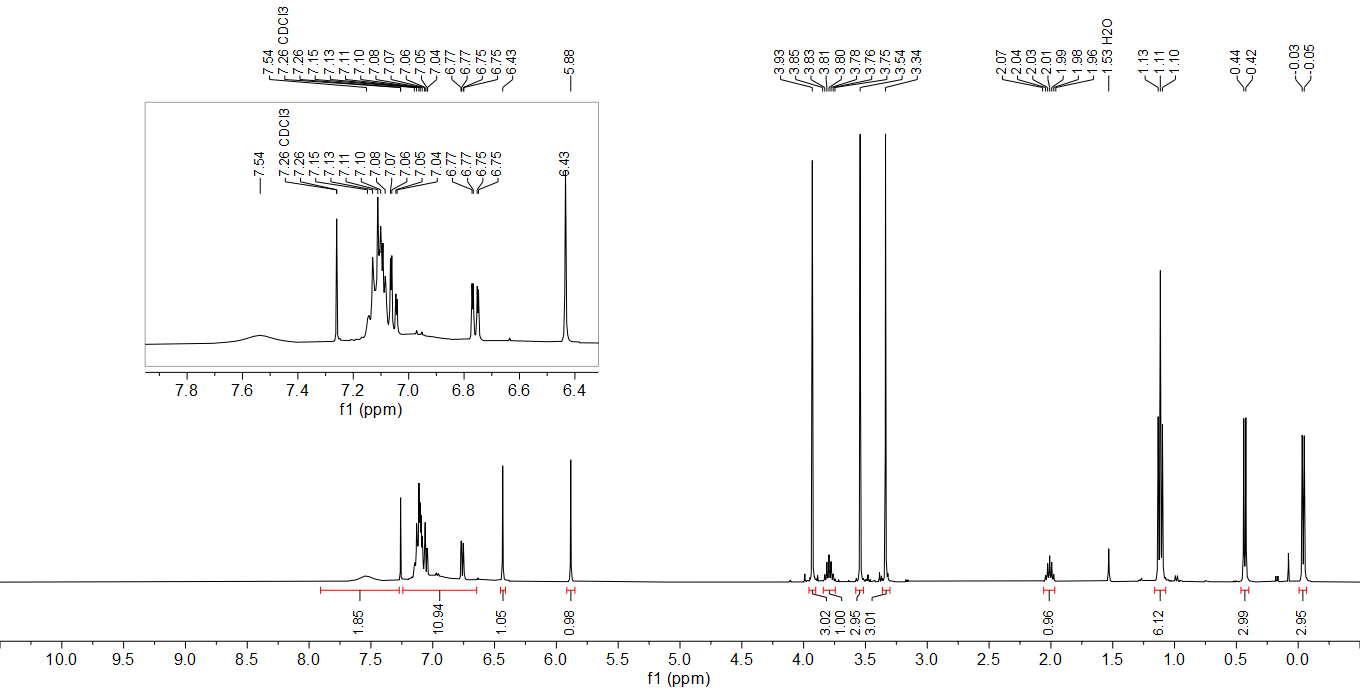


Figure S49: ^1^H NMR spectrum of methoxyisoindoline **3f**.


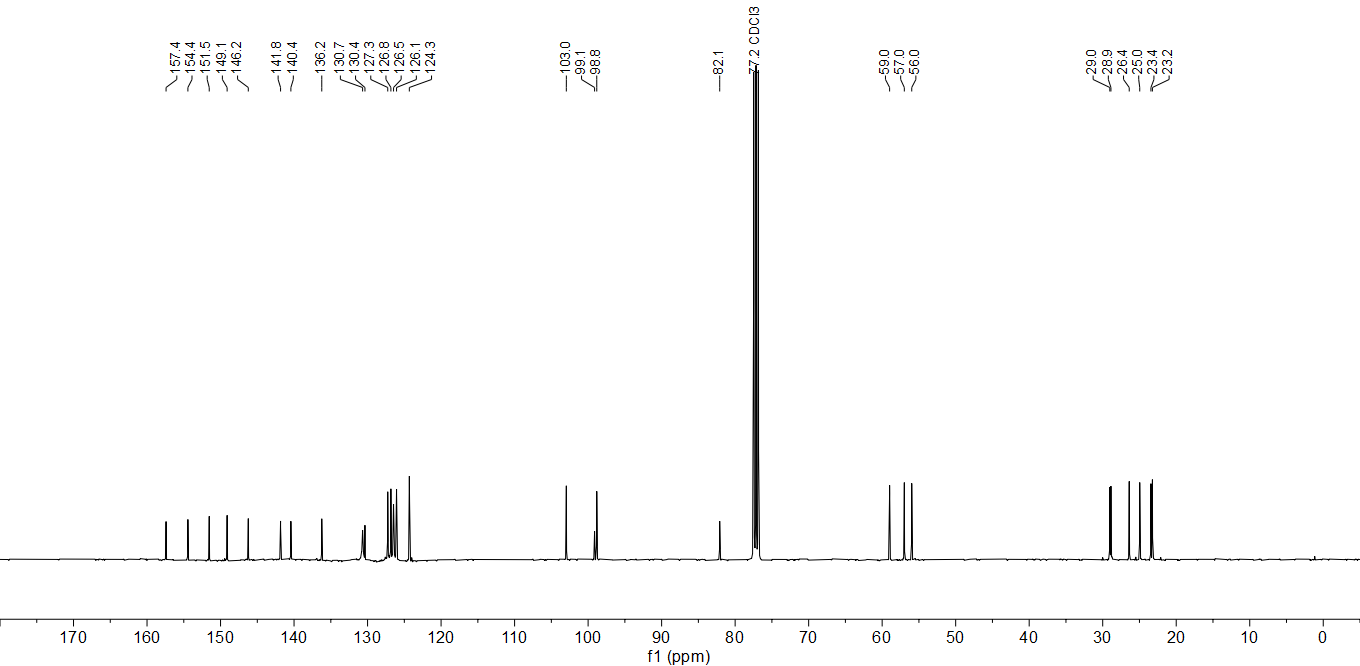


Figure S50: ^13^C{^1^H} NMR spectrum of compound **3f**.


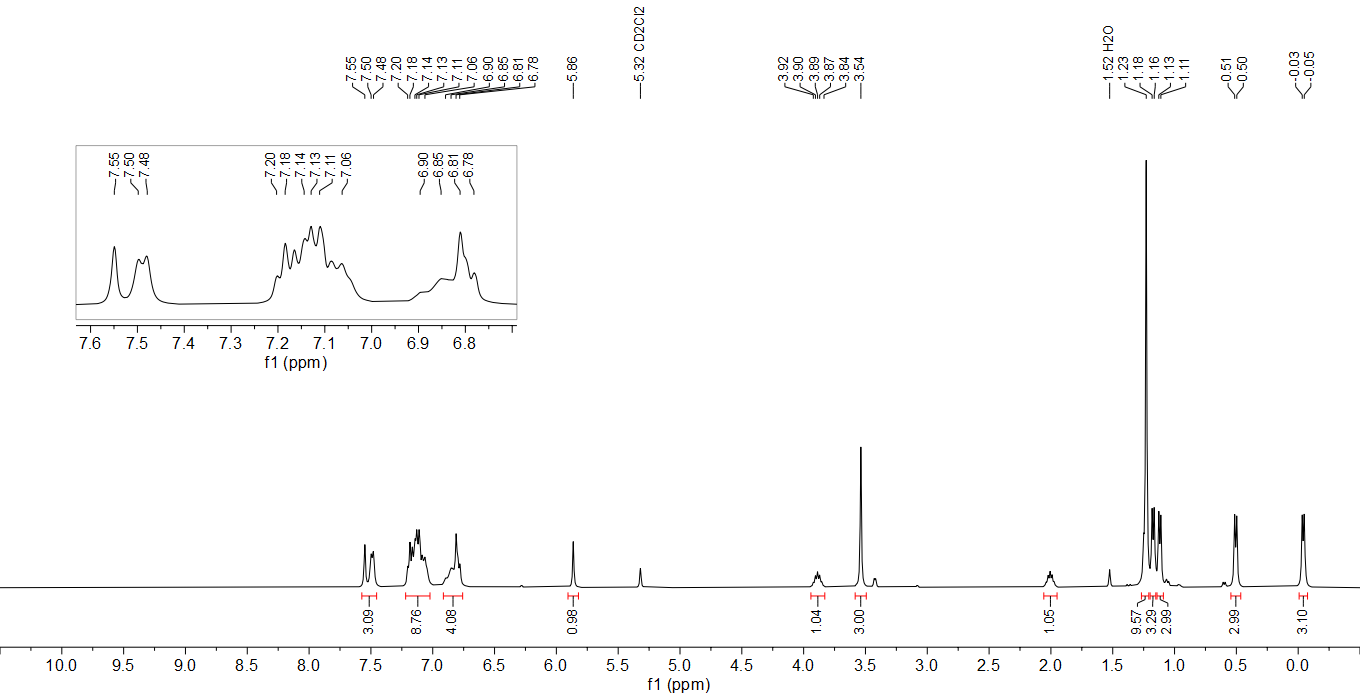


Figure S51: ^1^H NMR spectrum of methoxyisoindoline **3g**.


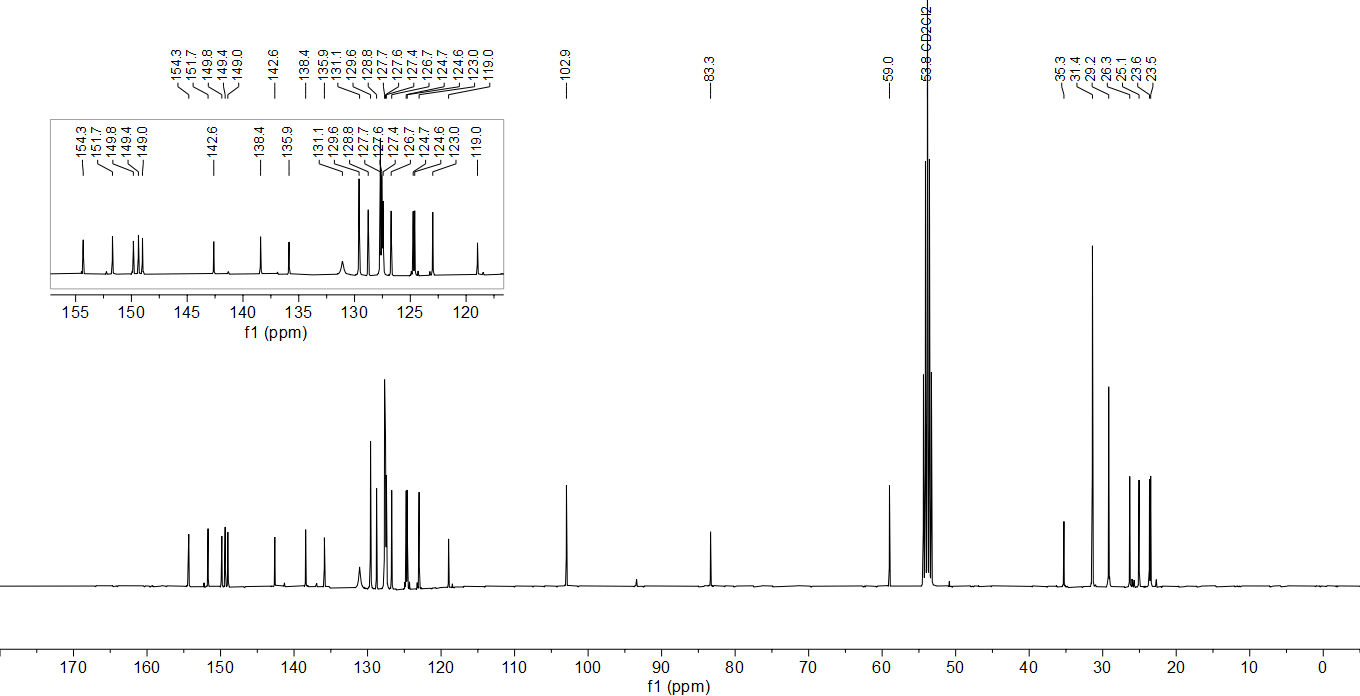


Figure S52: ^13^C{^1^H} NMR spectrum of compound **3g**.


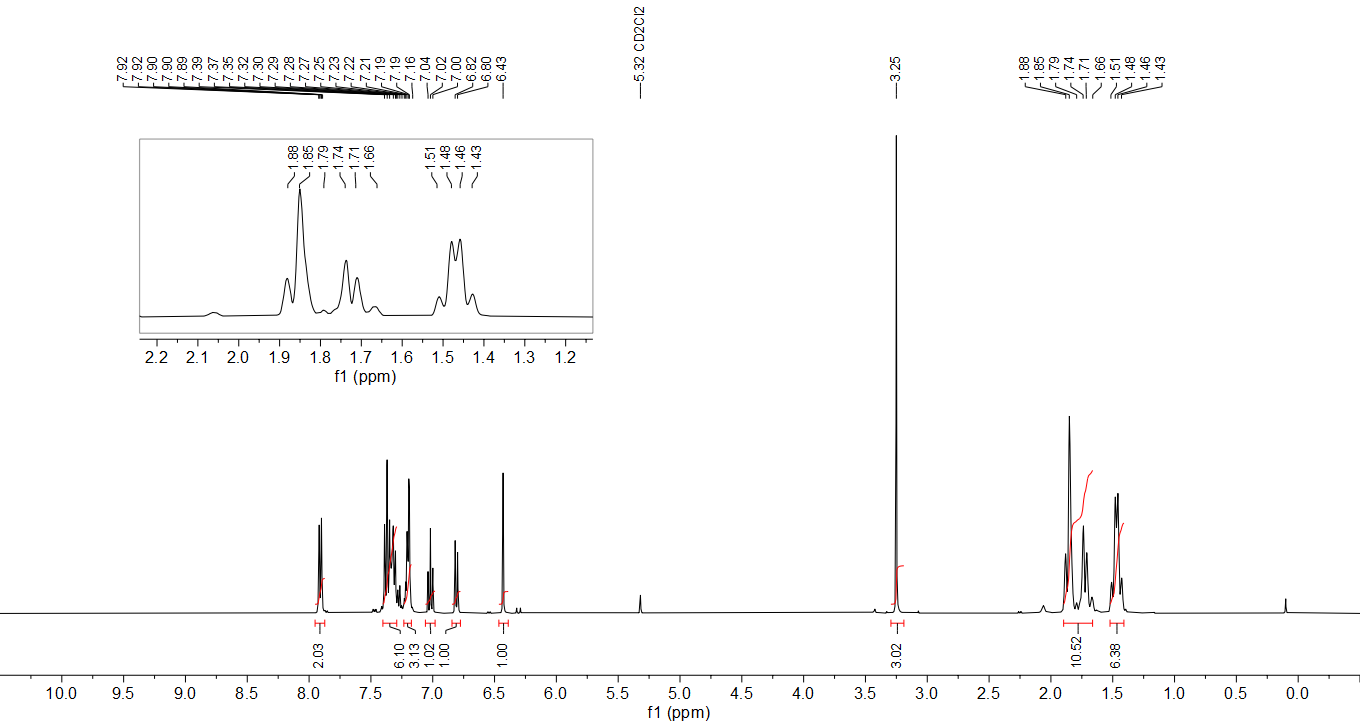


Figure S53: ^1^H NMR spectrum of methoxyisoindoline **3h**.


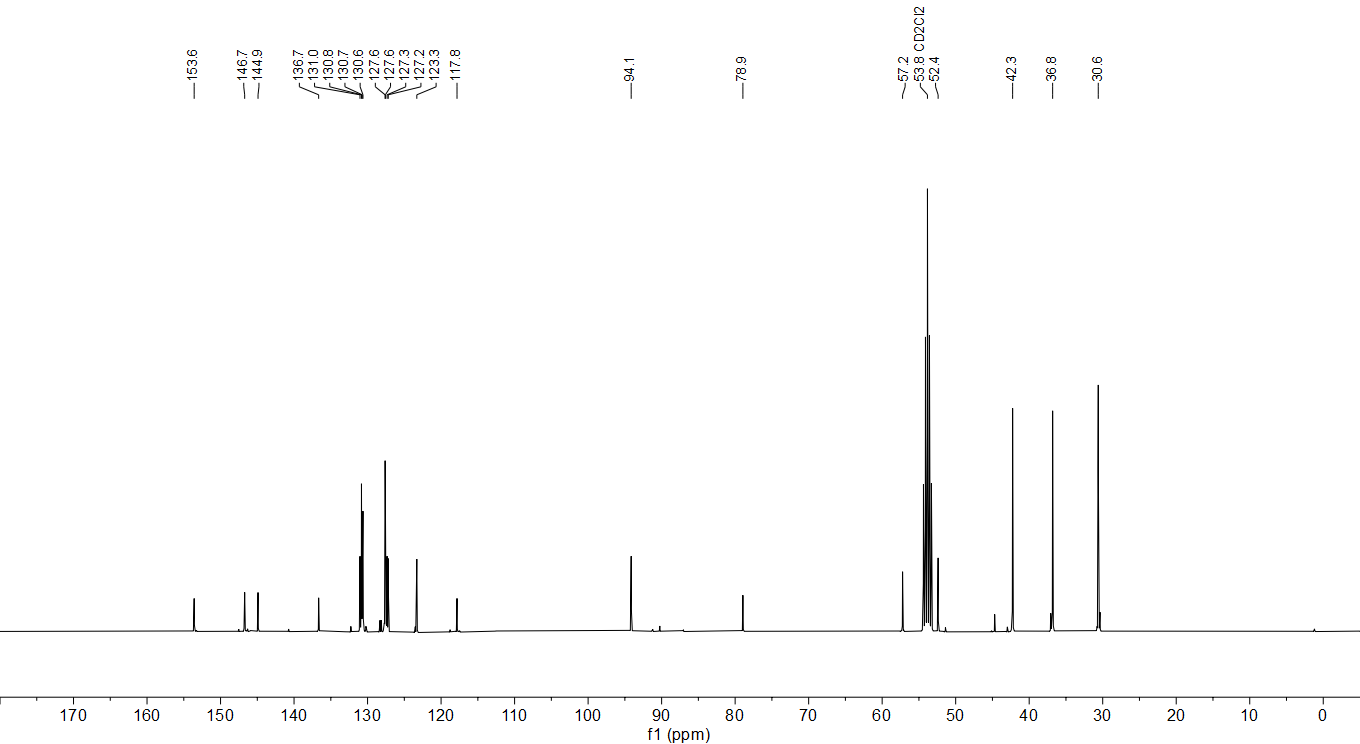


Figure S54: ^13^C{^1^H} NMR spectrum of compound **3h**.


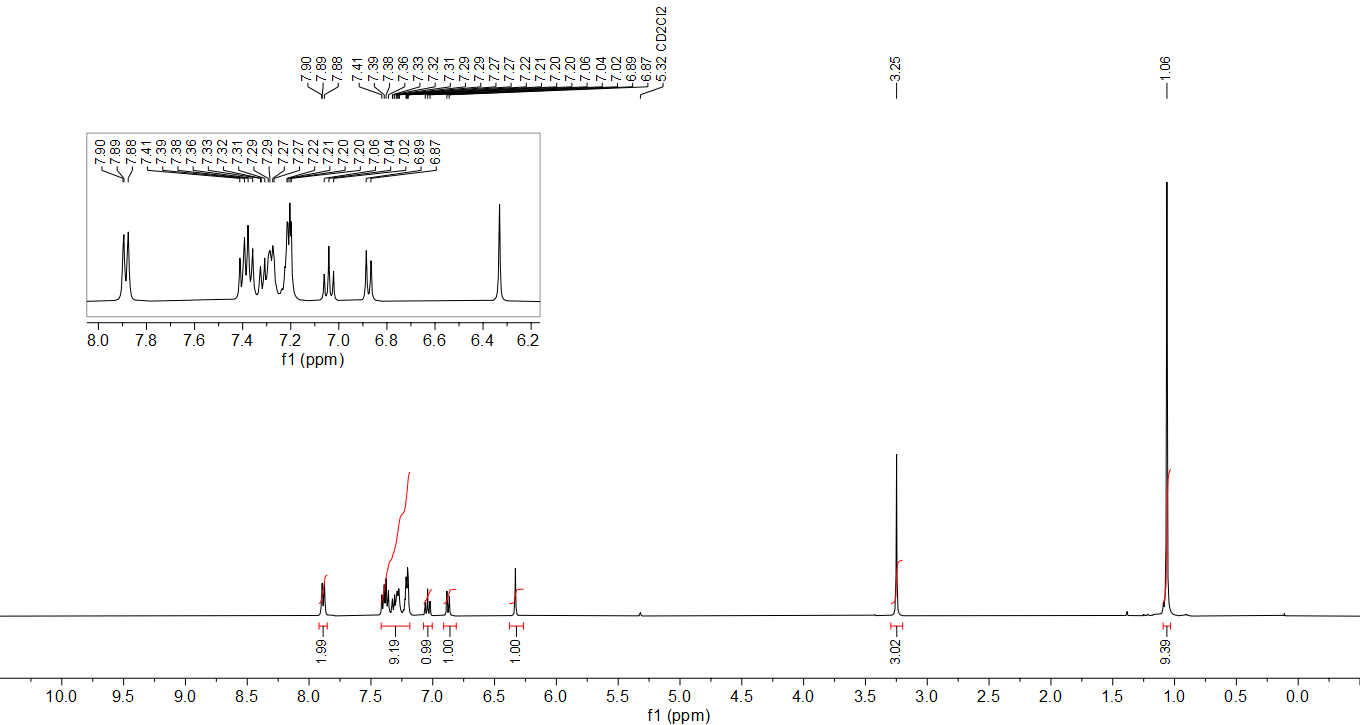


Figure S55: ^1^H NMR spectrum of methoxyisoindoline **3i**.


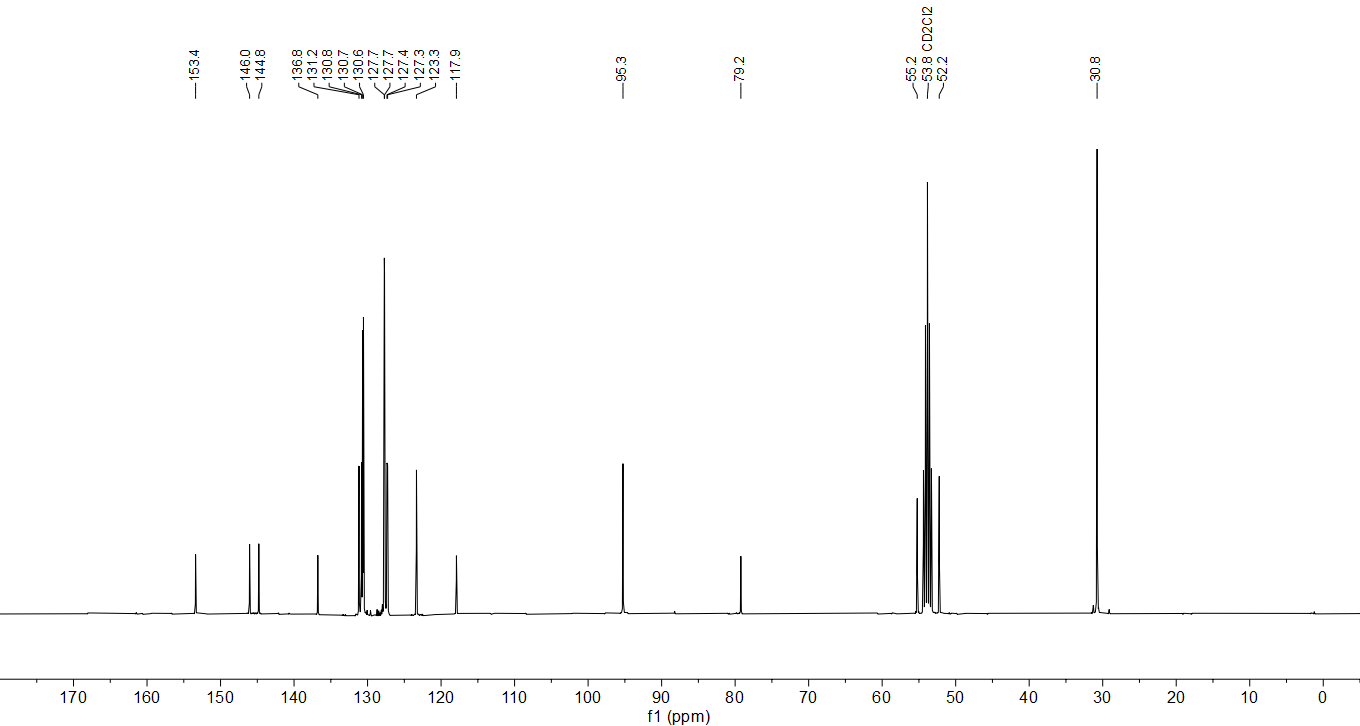


Figure S56: ^13^C{^1^H} NMR spectrum of compound **3i**.


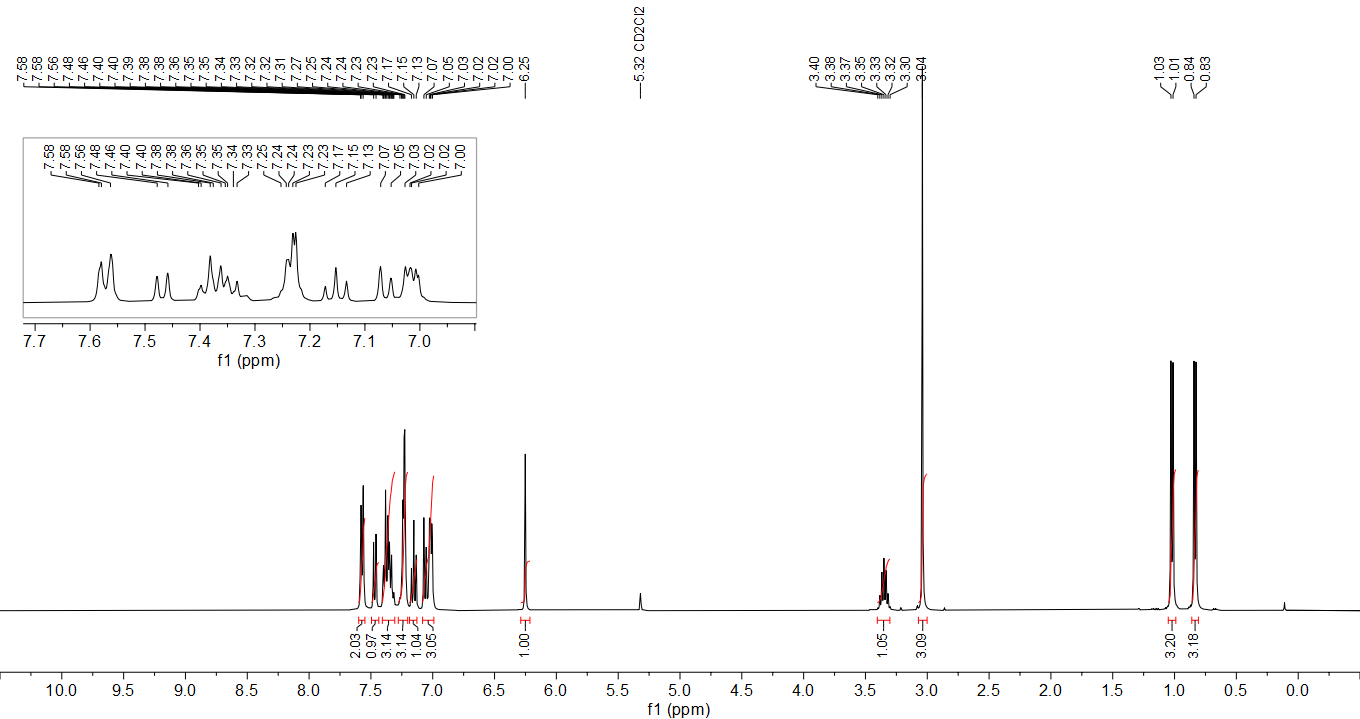


Figure S57: ^1^H NMR spectrum of methoxyisoindoline **3j**.


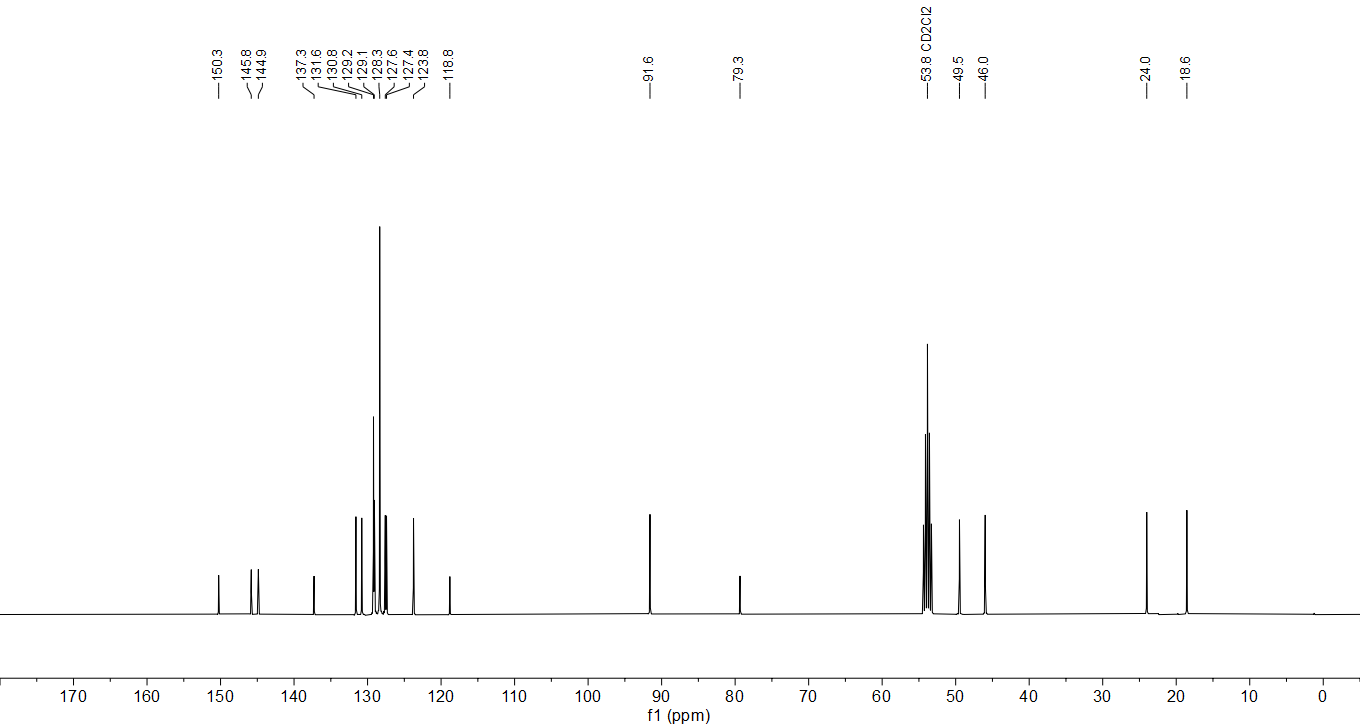


Figure S58: ^13^C{^1^H} NMR spectrum of methoxyisoindoline **3j**.


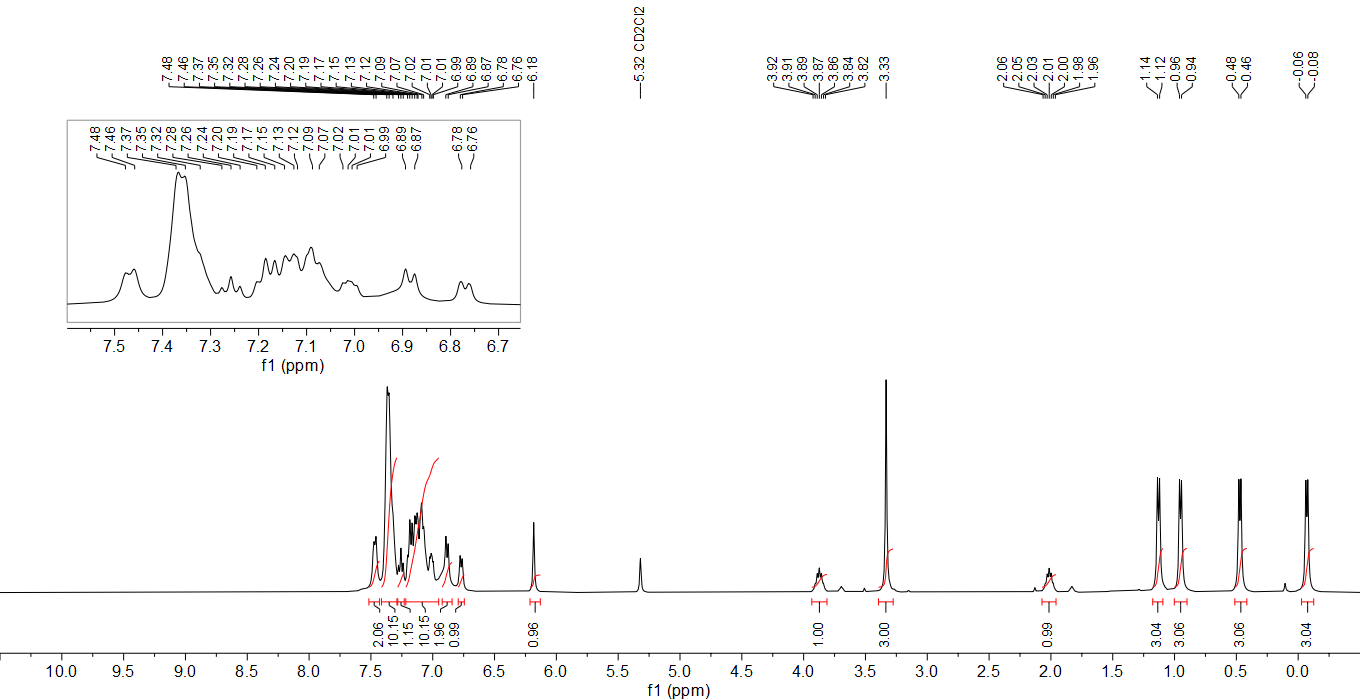


Figure S59: ^1^H NMR spectrum of methoxyisoindoline phosphine **4a**.


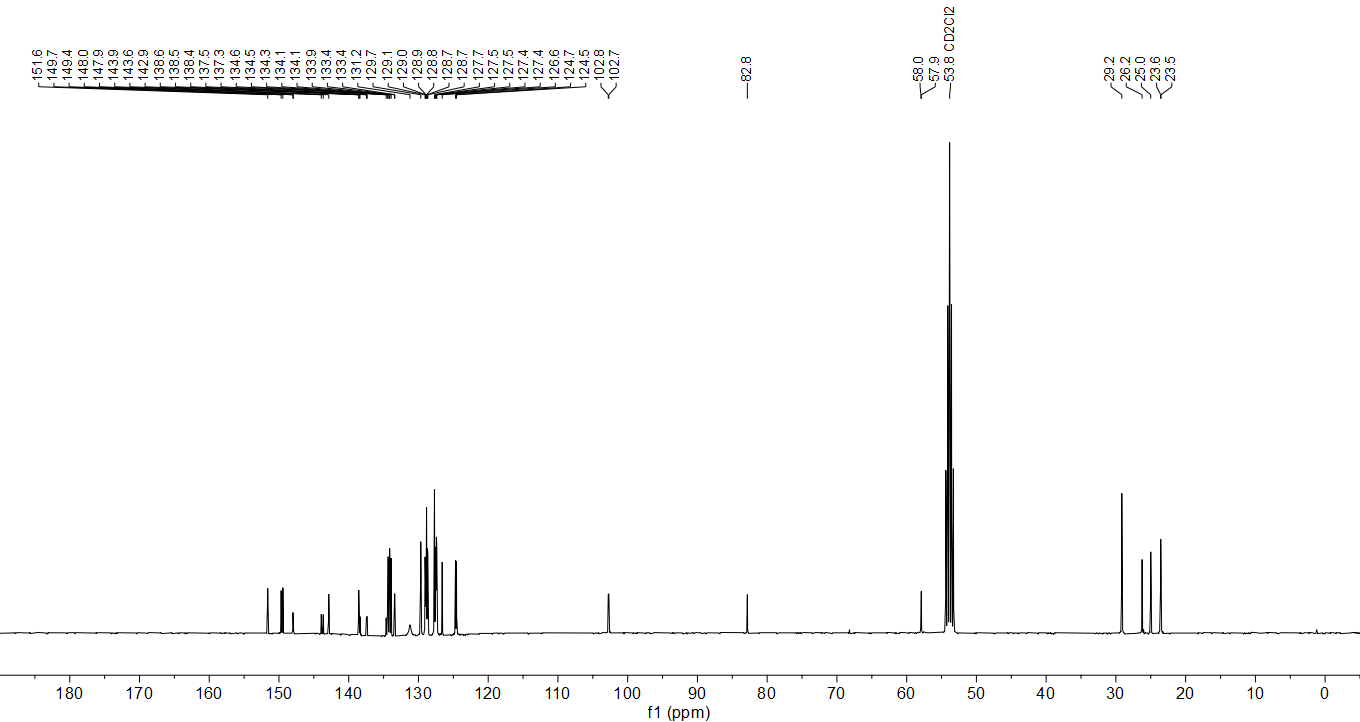


Figure S60: ^13^C{^1^H} NMR spectrum of methoxyisoindoline phosphine **4a**.


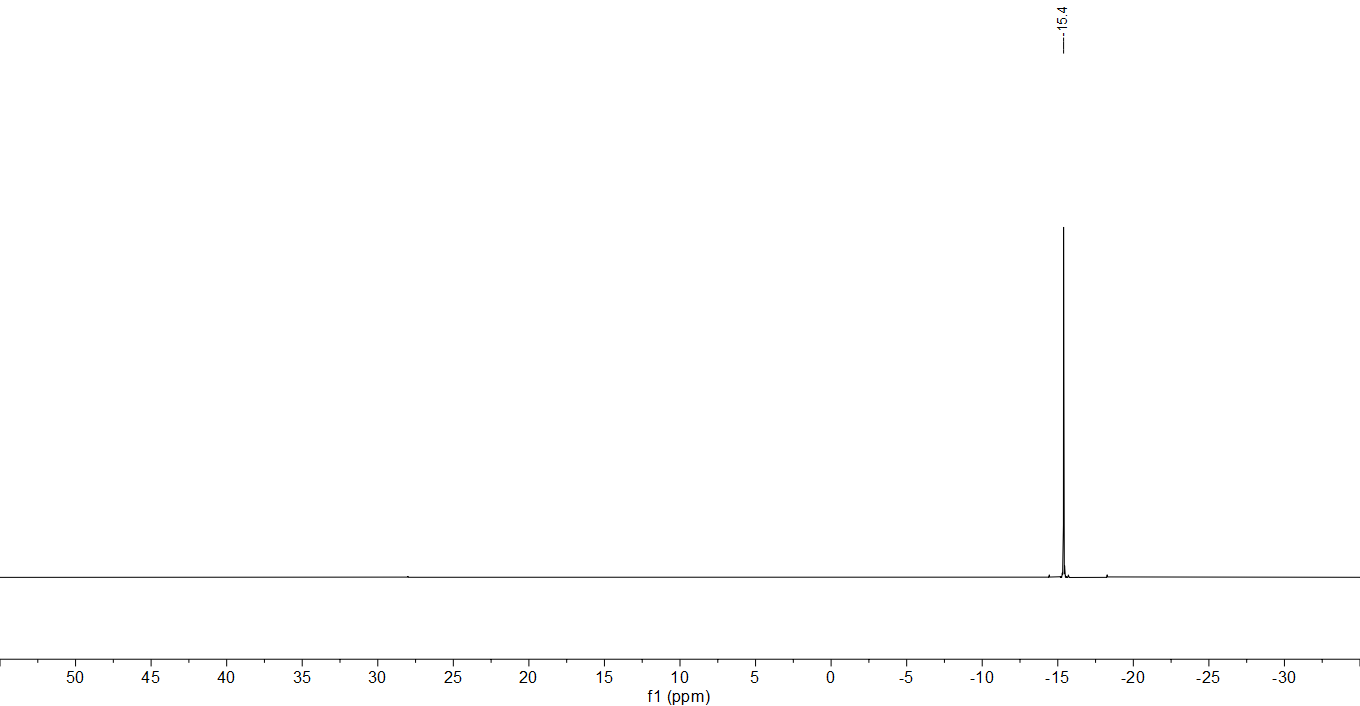


Figure S61: ^31^P{^1^H} NMR spectrum of methoxyisoindoline phosphine **4a**.


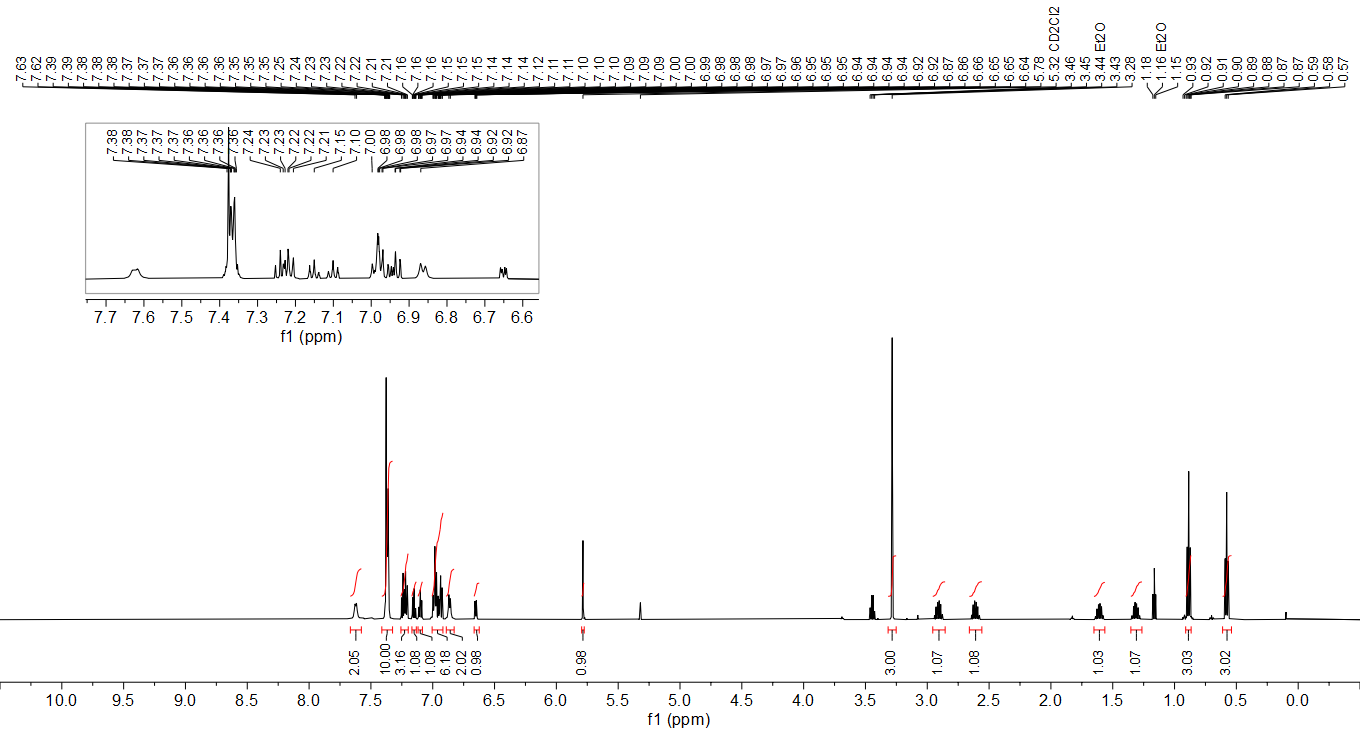


Figure S62: ^1^H NMR spectrum of methoxyisoindoline phosphine **4b**.


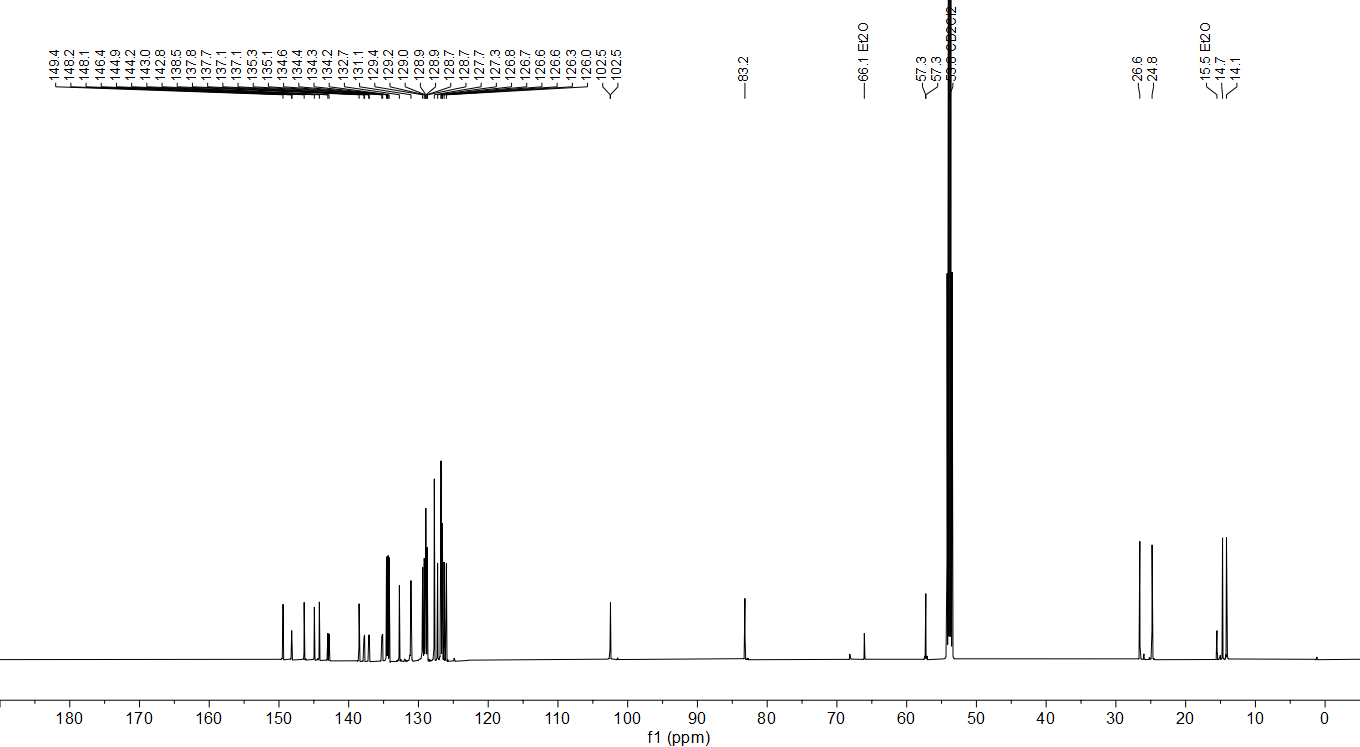


Figure S63: ^13^C{^1^H} NMR spectrum of methoxyisoindoline phosphine **4b**.


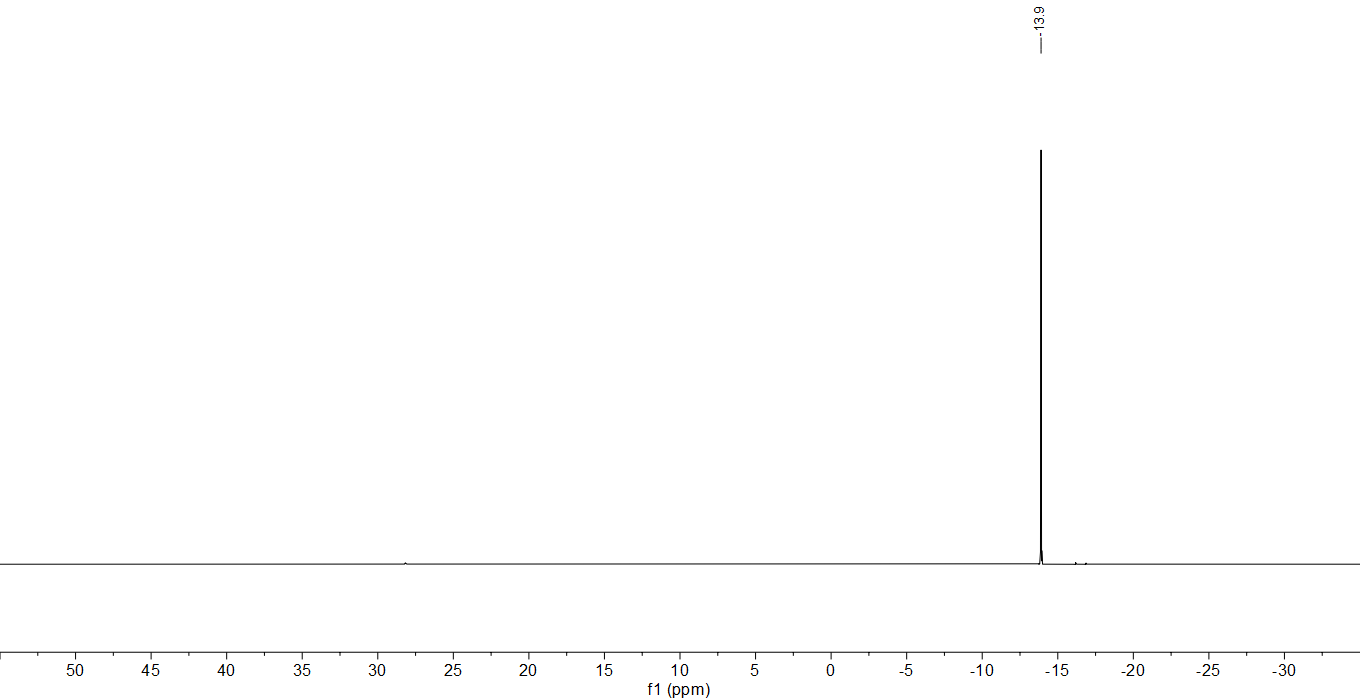


Figure S64: ^31^P{^1^H} NMR spectrum of methoxyisoindoline phosphine **4b**.


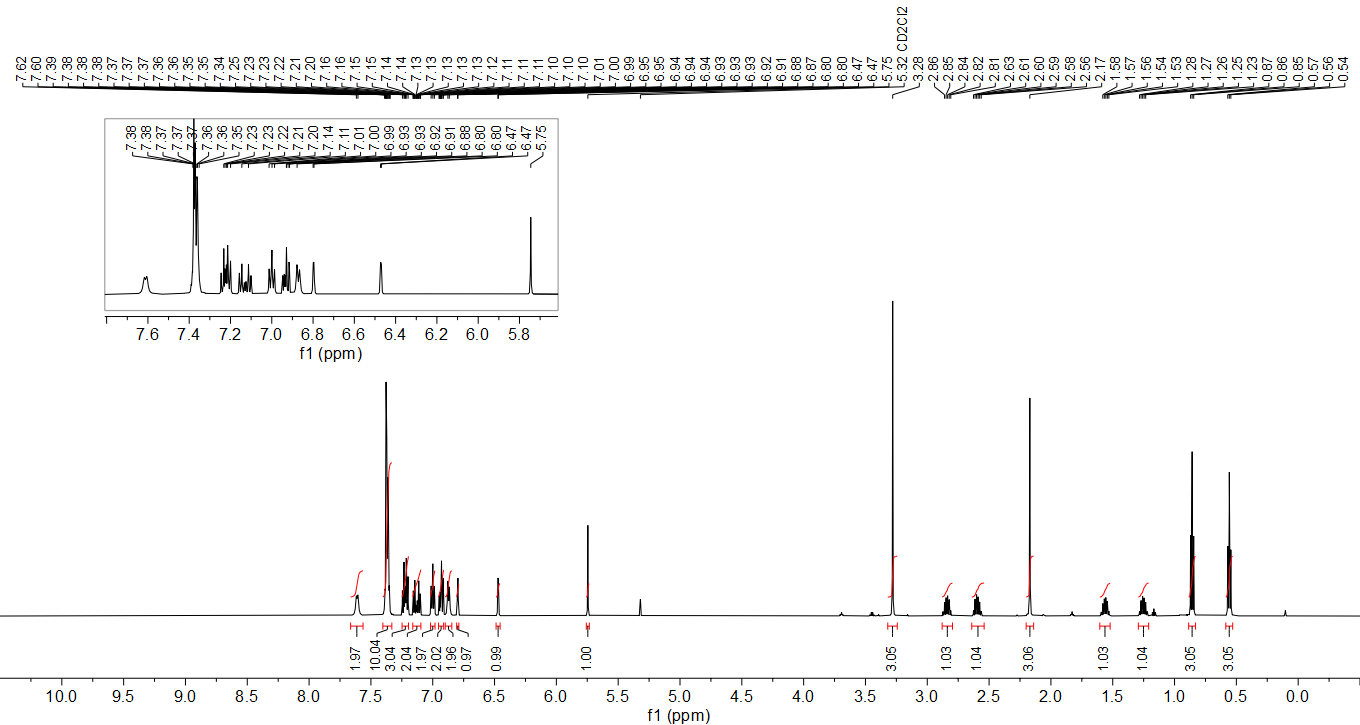


Figure S65: ^1^H NMR spectrum of methoxyisoindoline phosphine **4c**.


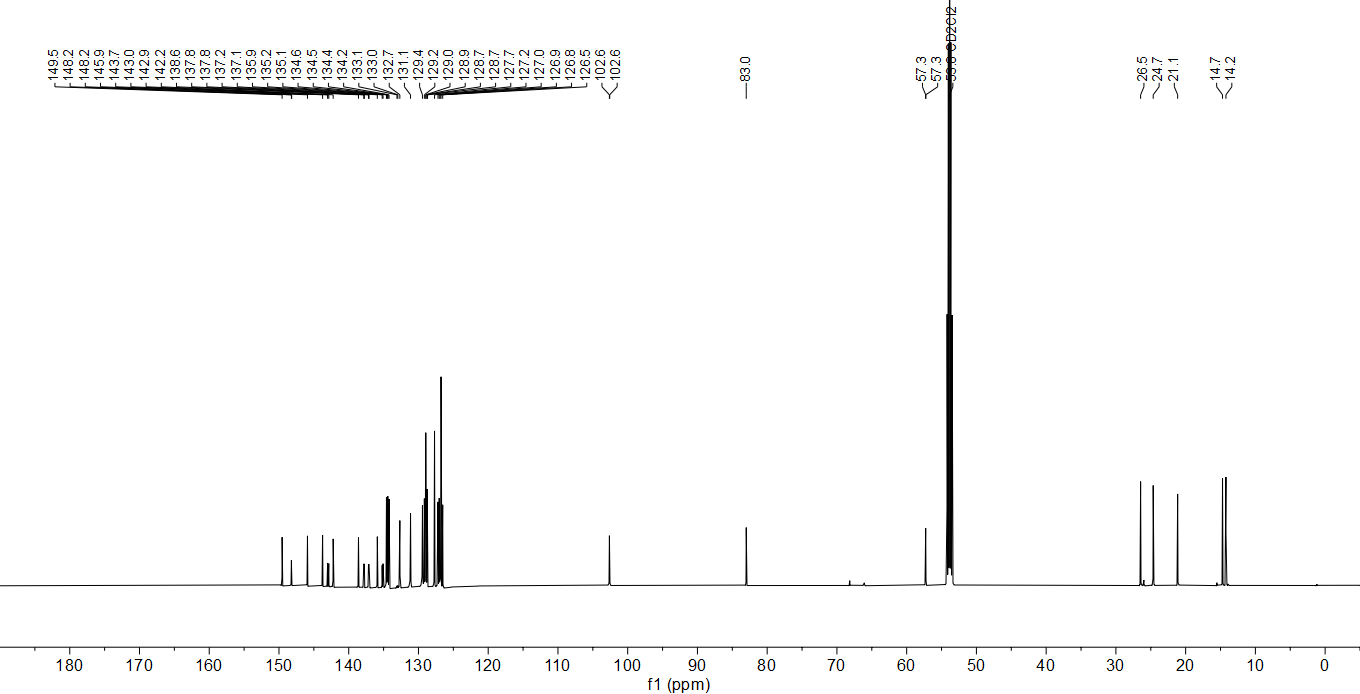


Figure S66: ^13^C{^1^H} NMR spectrum of methoxyisoindoline phosphine **4c**.


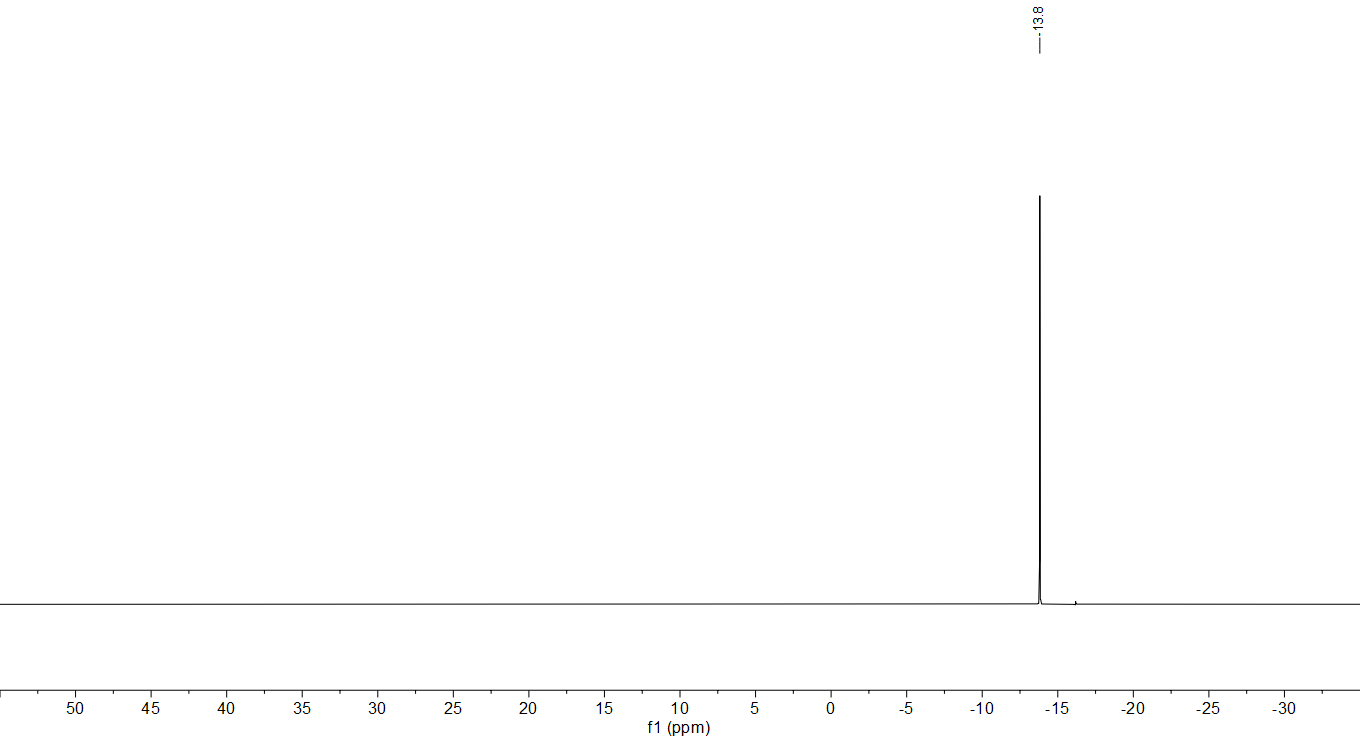


Figure S67: ^31^P{^1^H} NMR spectrum of methoxyisoindoline phosphine **4c**.


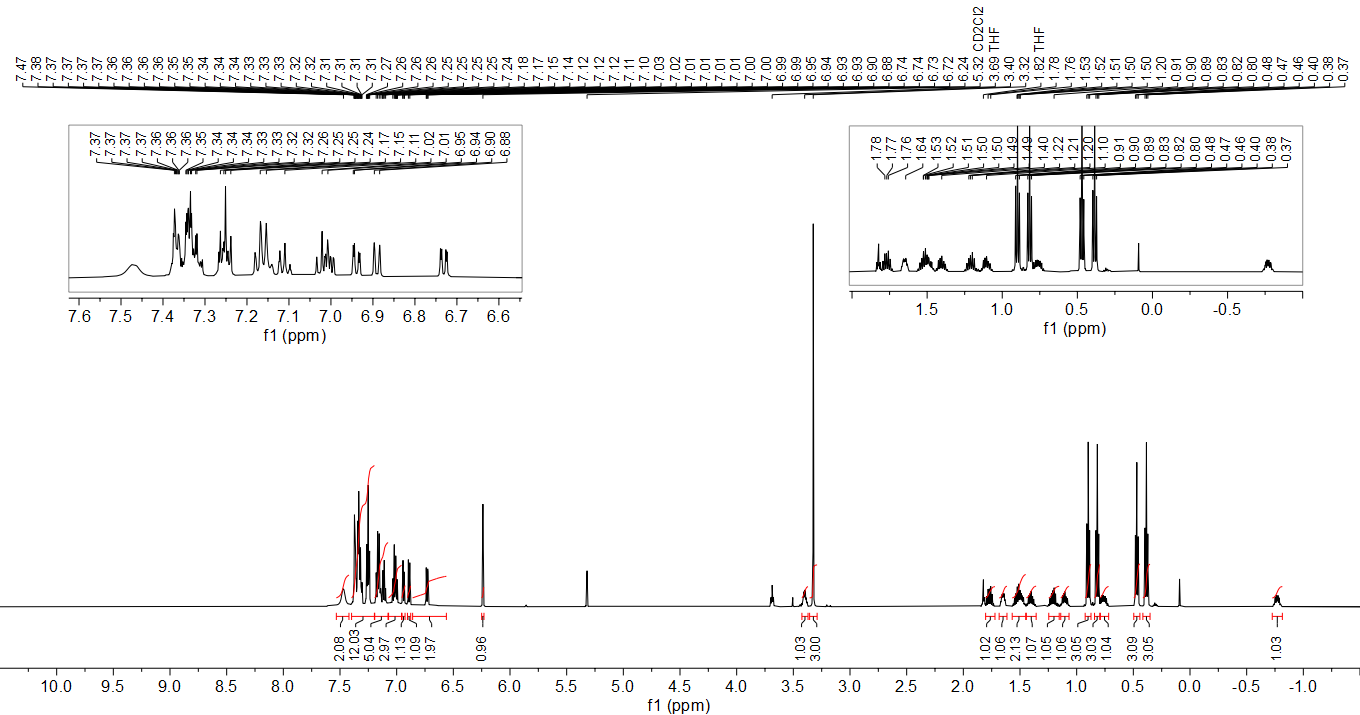


Figure S68: ^1^H NMR spectrum of methoxyisoindoline phosphine **4d**.


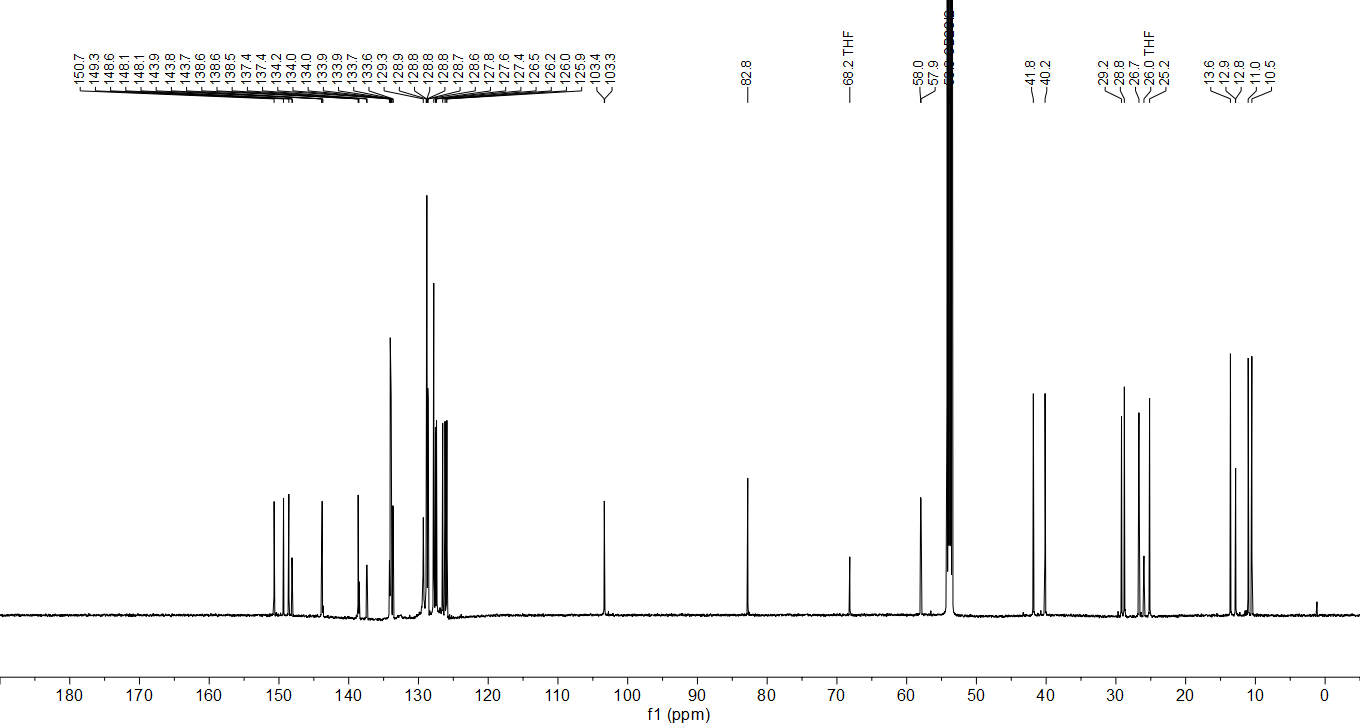


Figure S69: ^13^C{^1^H} NMR spectrum of methoxyisoindoline phosphine **4d**.


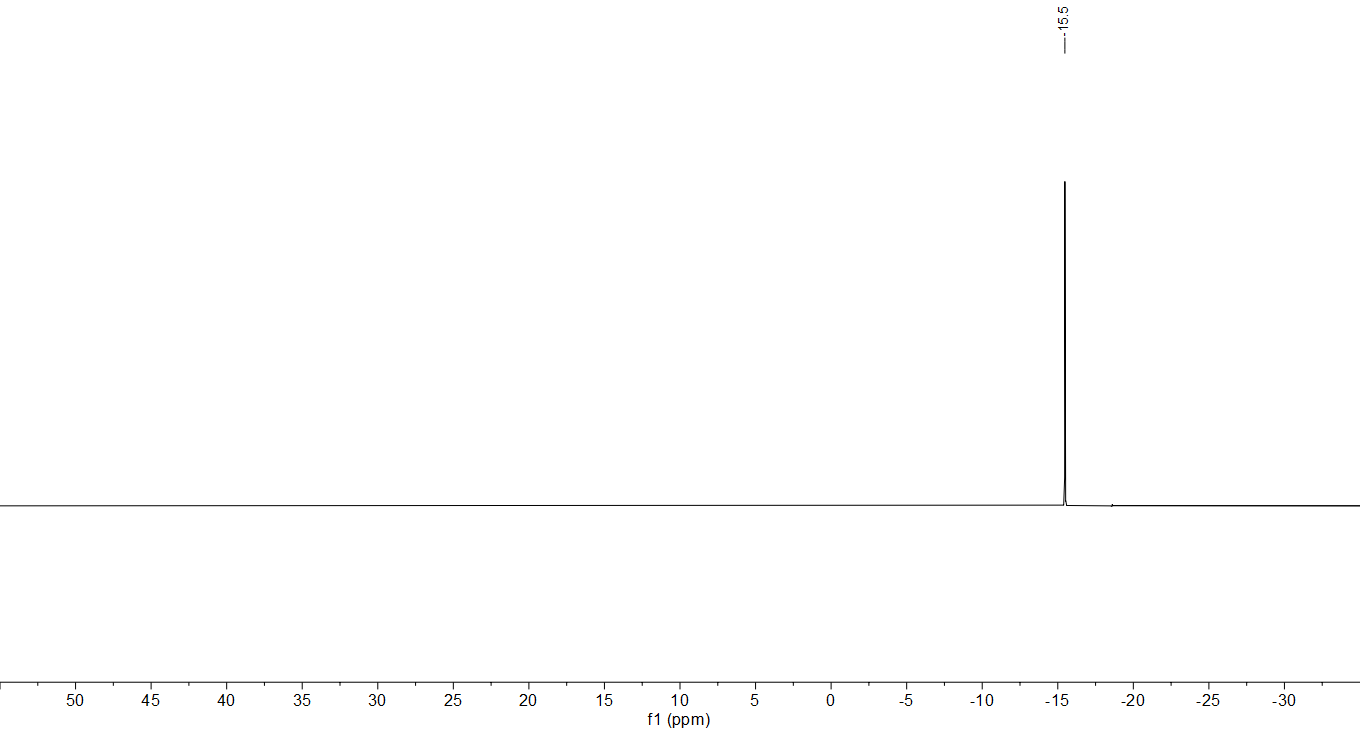


Figure S70: ^31^P{^1^H} NMR spectrum of methoxyisoindoline phosphine **4d**.


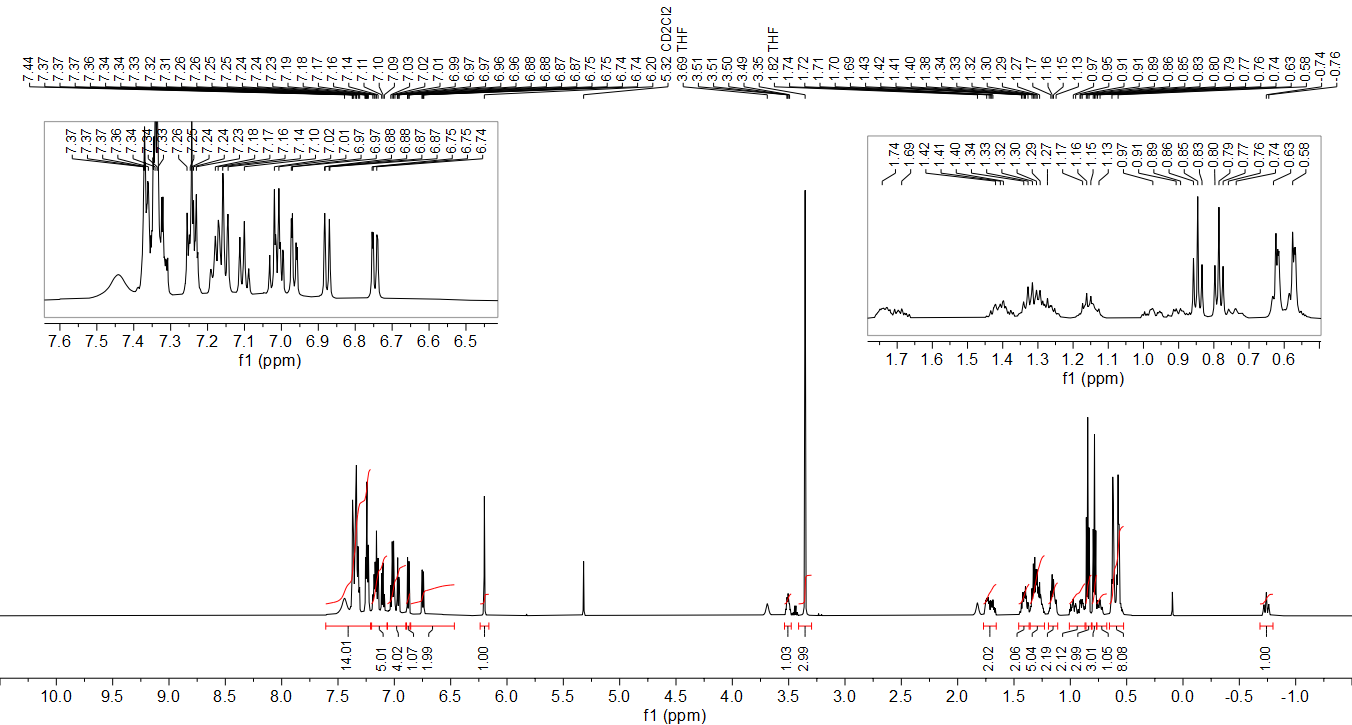


Figure S71: ^1^H NMR spectrum of methoxyisoindoline phosphine **4e**.


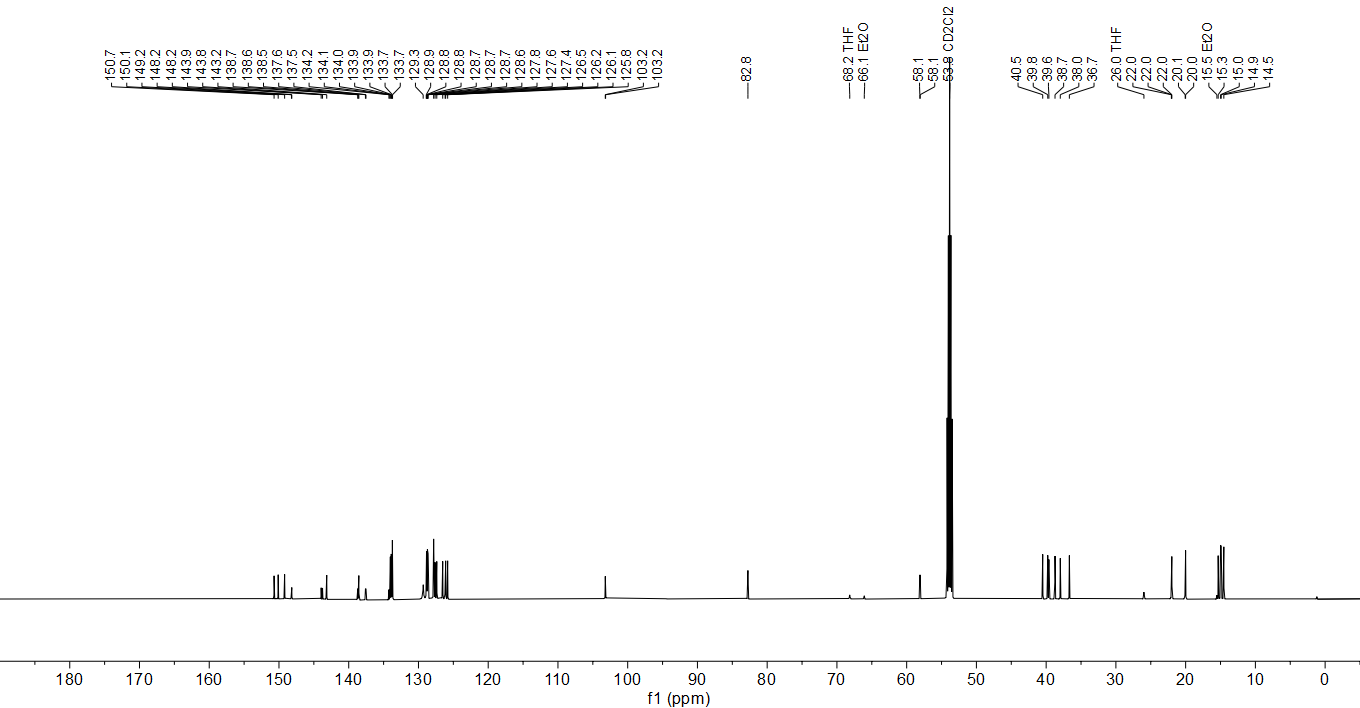


Figure S72: ^13^C{^1^H} NMR spectrum of methoxyisoindoline phosphine **4e**.


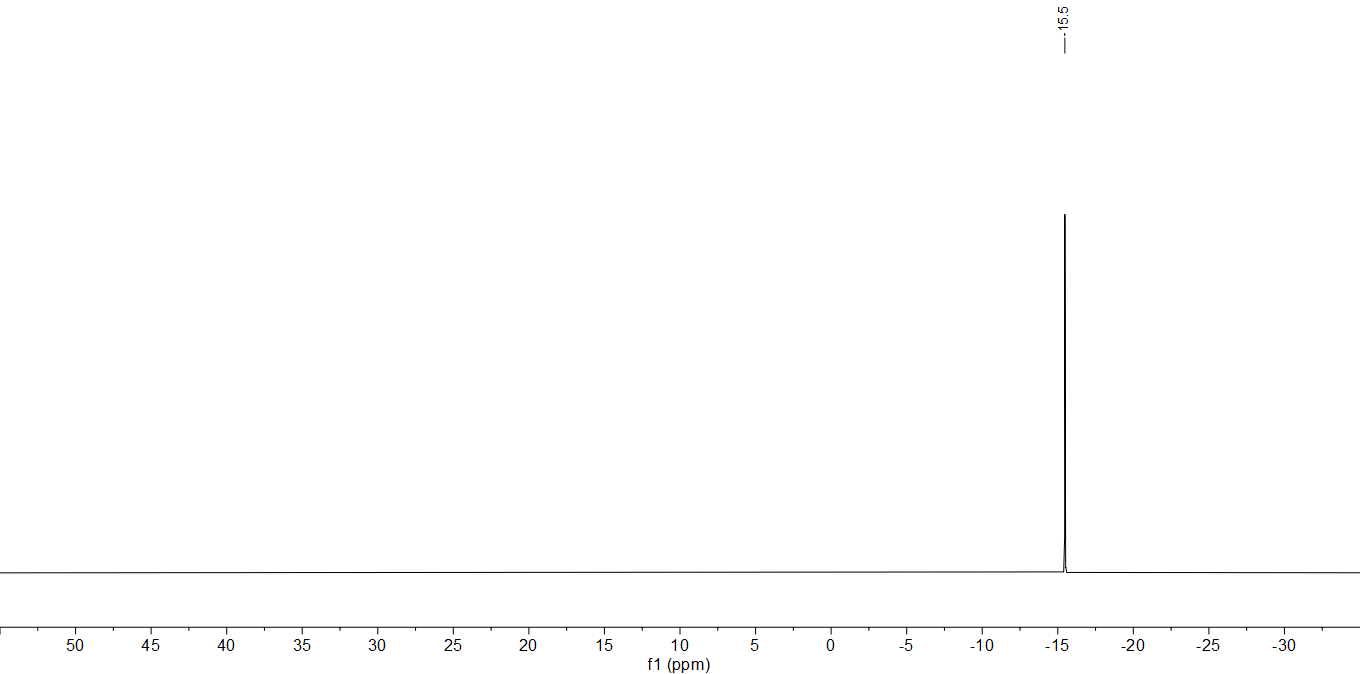


Figure S73: ^31^P{^1^H} NMR spectrum of methoxyisoindoline phosphine **4e**.


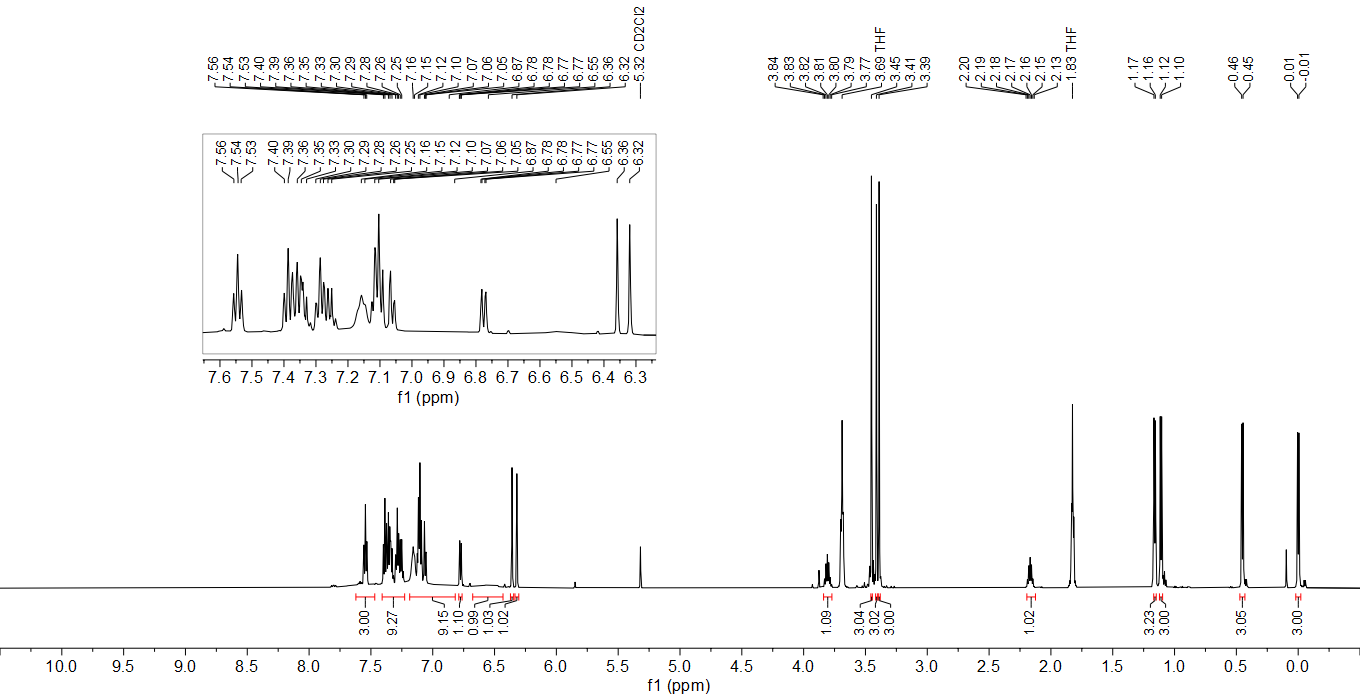


Figure S74: ^1^H NMR spectrum of methoxyisoindoline phosphine **4f**.


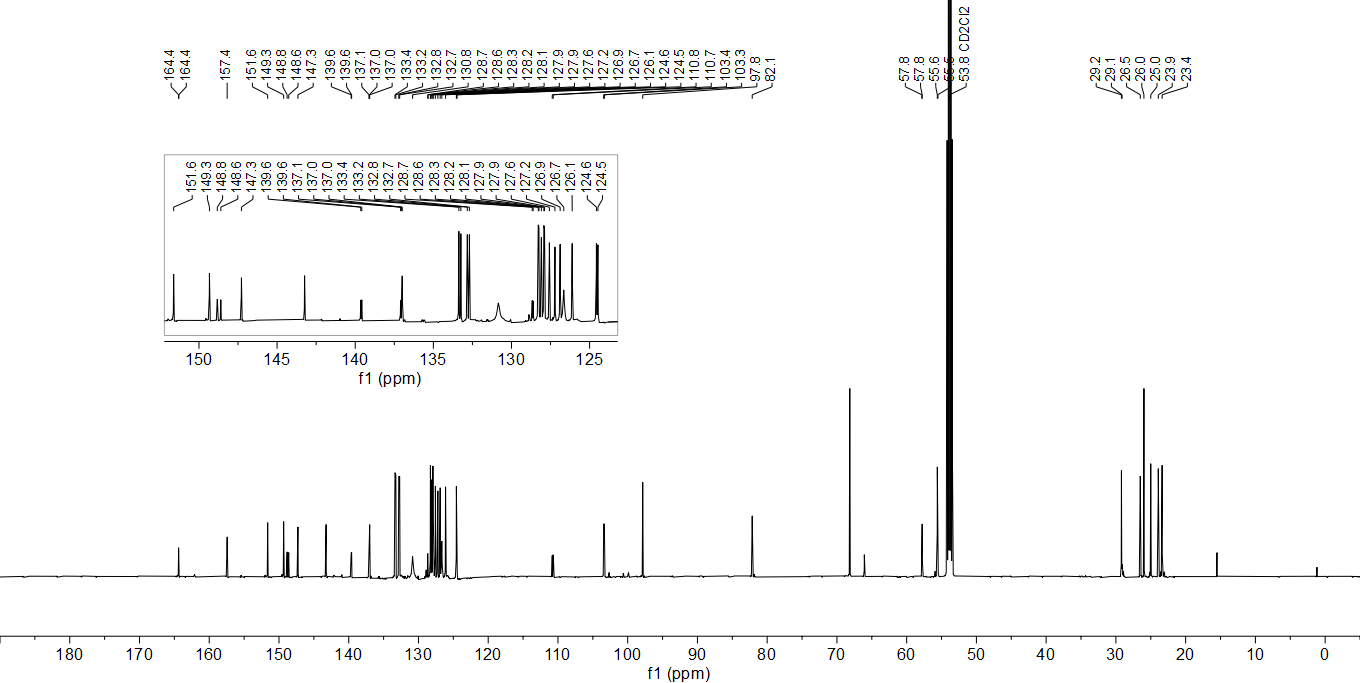


Figure S75: ^13^C{^1^H} NMR spectrum of methoxyisoindoline phosphine **4f**.


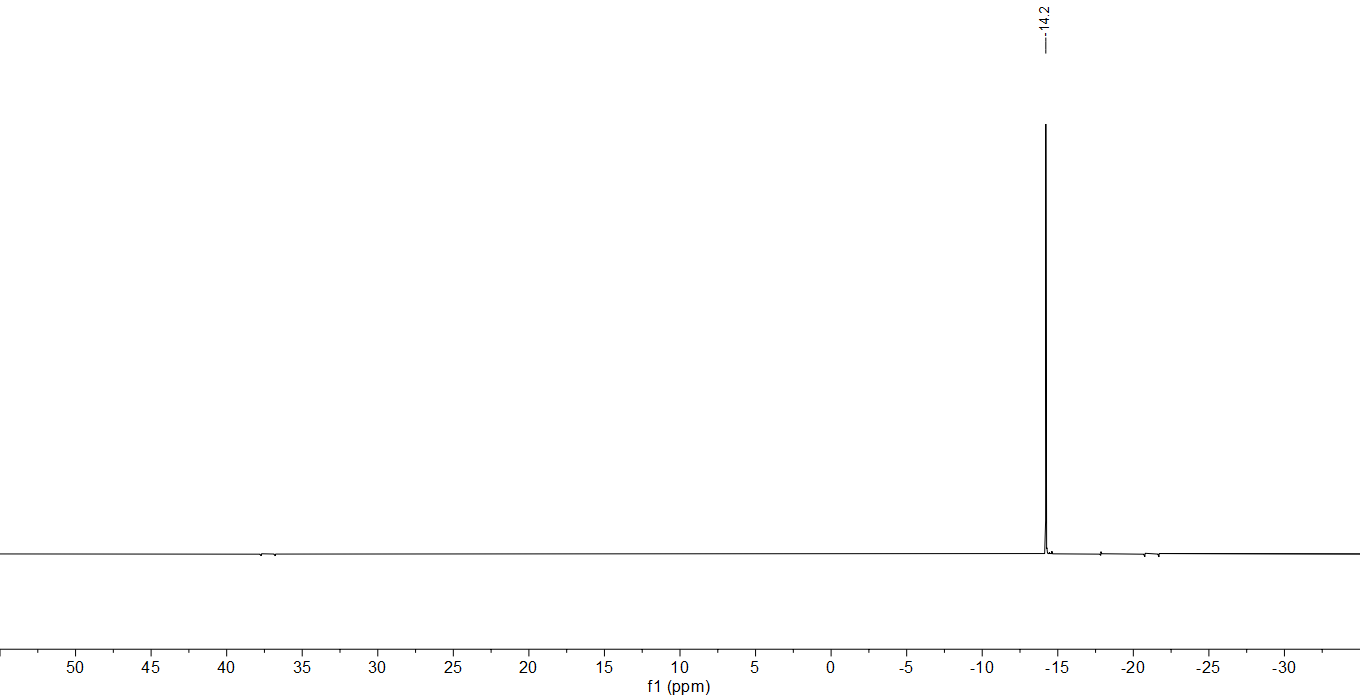


Figure S76: ^31^P{^1^H} NMR spectrum of methoxyisoindoline phosphine **4f**.


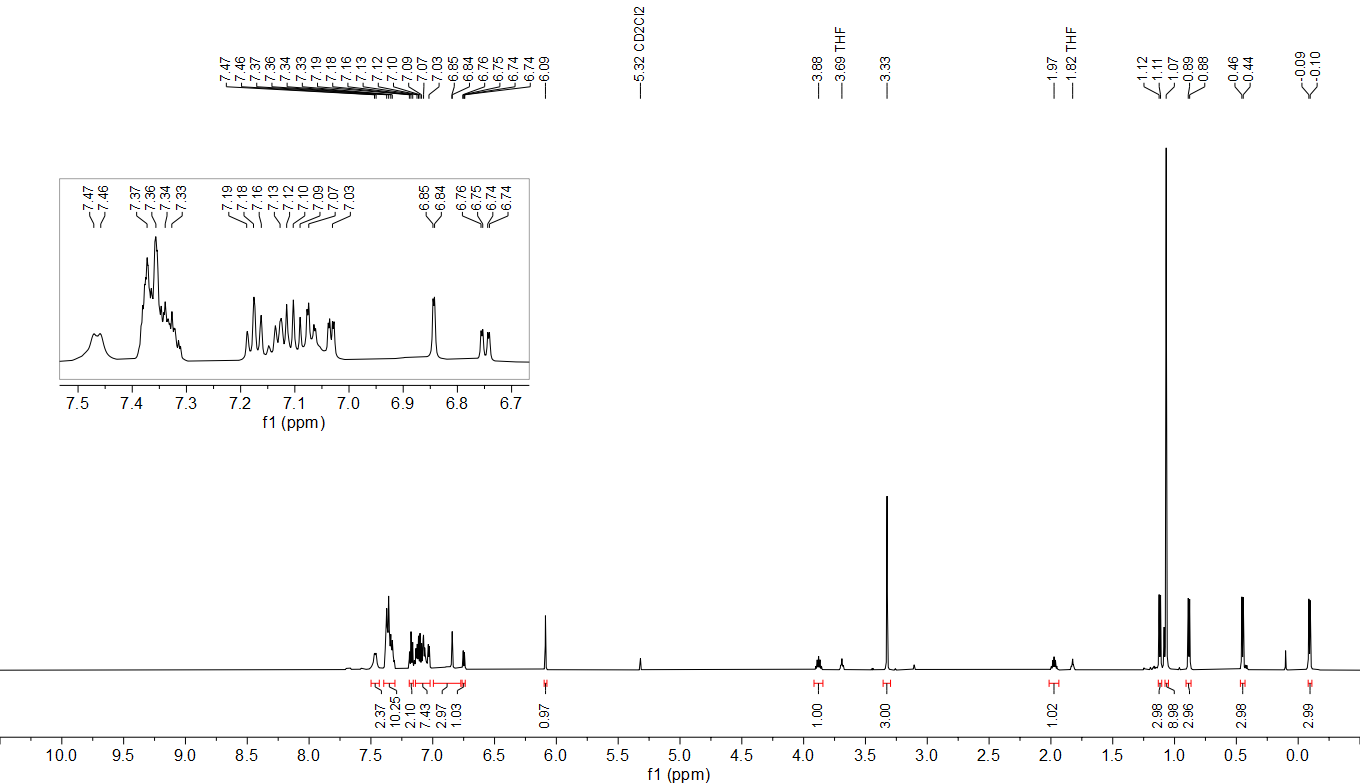


Figure S77: ^1^H NMR spectrum of methoxyisoindoline phosphine **4g**.


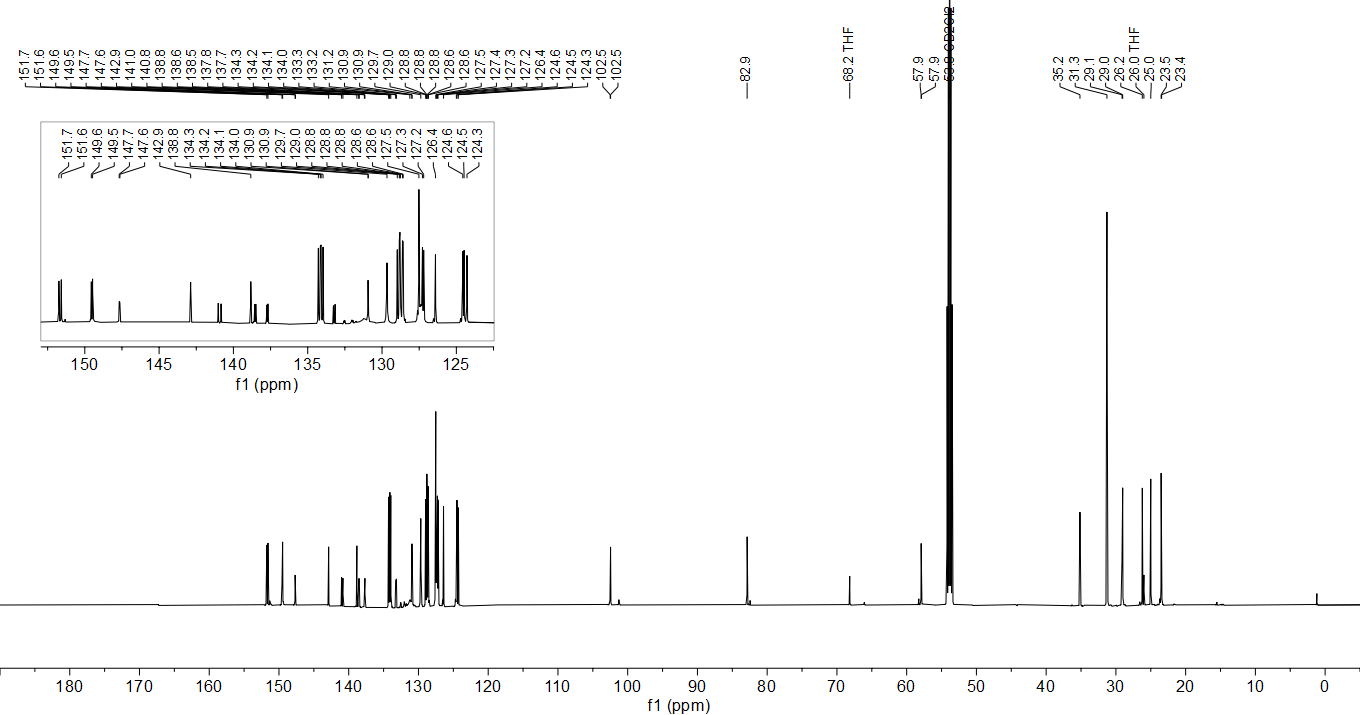


Figure S78: ^13^C{^1^H} NMR spectrum of methoxyisoindoline phosphine **4g**.


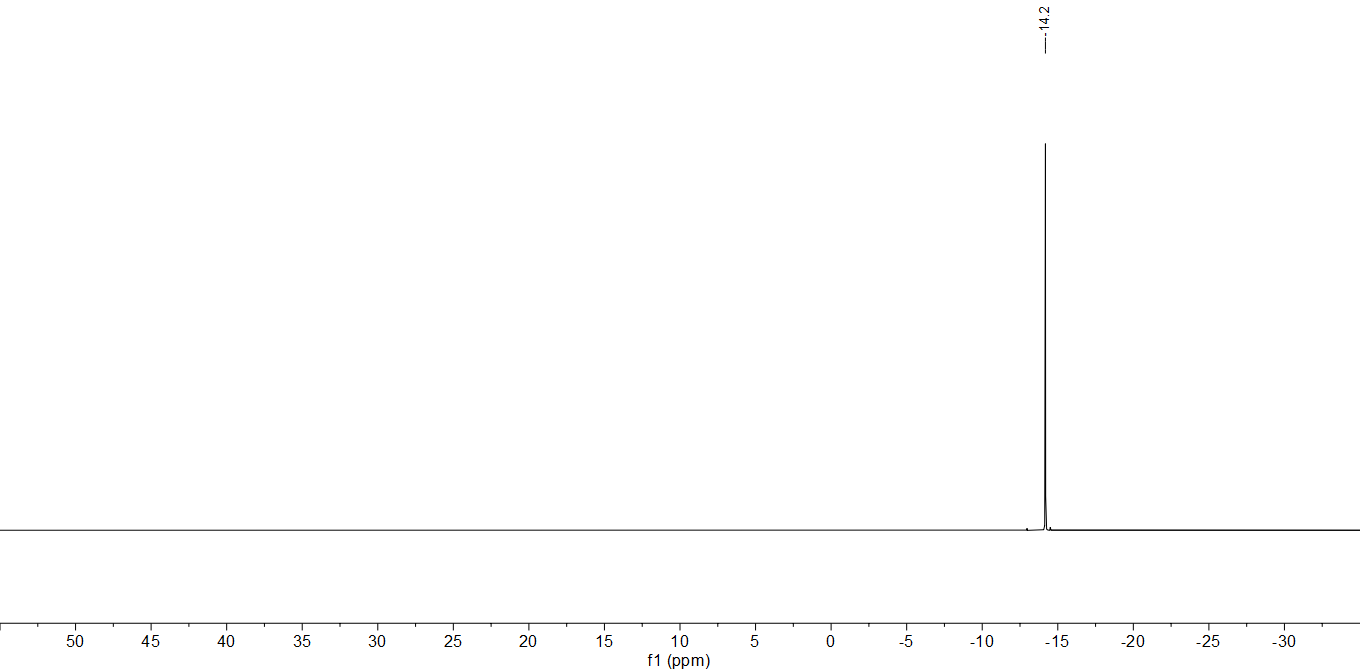


Figure S79: ^31^P{^1^H} NMR spectrum of methoxyisoindoline phosphine **4g**.


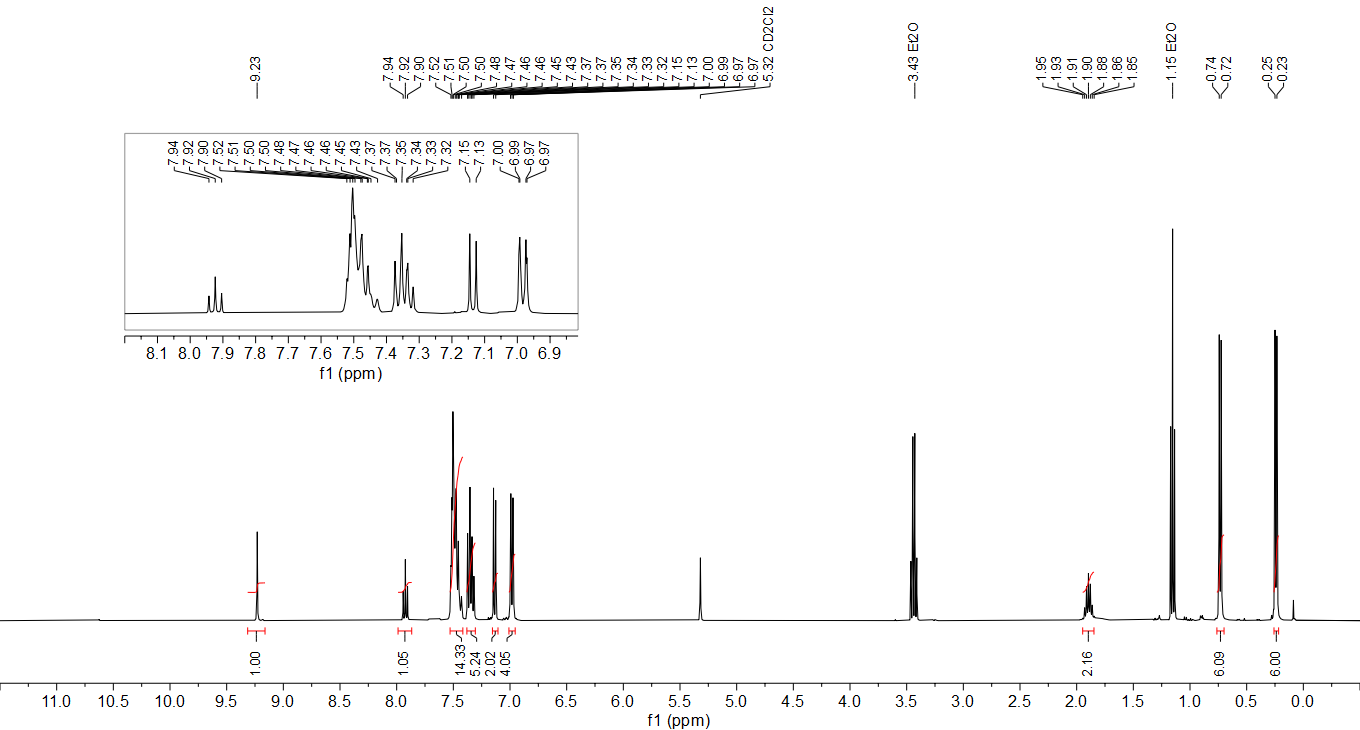


Figure S80: ^1^H NMR spectrum of phosphine isoindolium tetrafluoroborate **5a**.


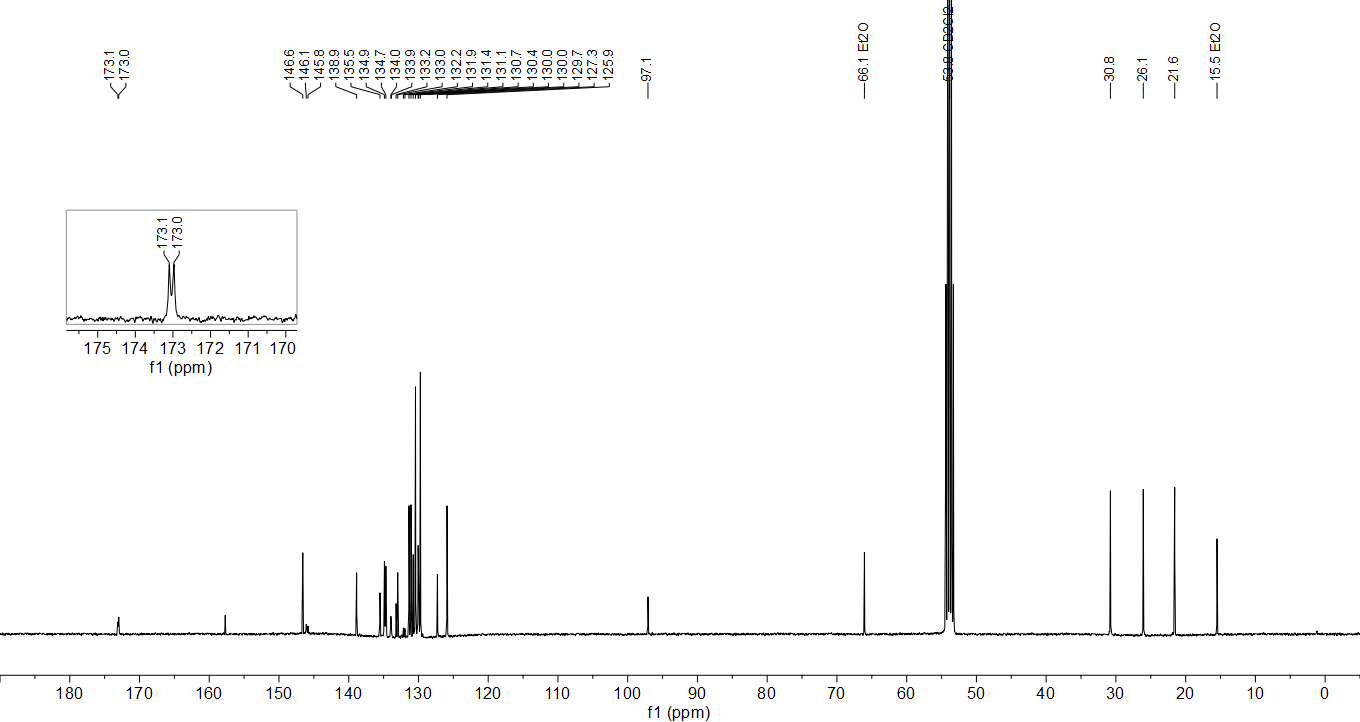


Figure S81: ^13^C{^1^H} NMR spectrum of phosphine isoindolium tetrafluoroborate **5a**.


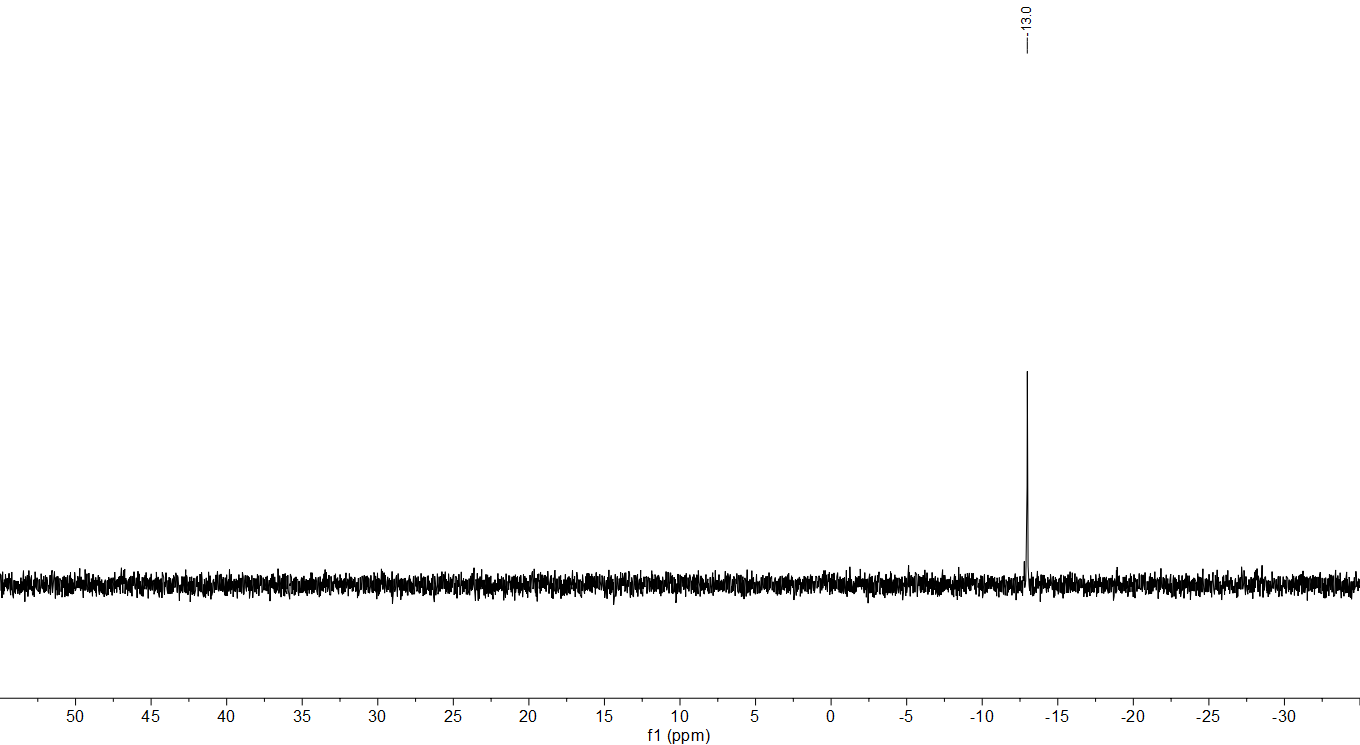


Figure S82: ^31^P{^1^H} NMR spectrum of phosphine isoindolium tetrafluoroborate **5a**.


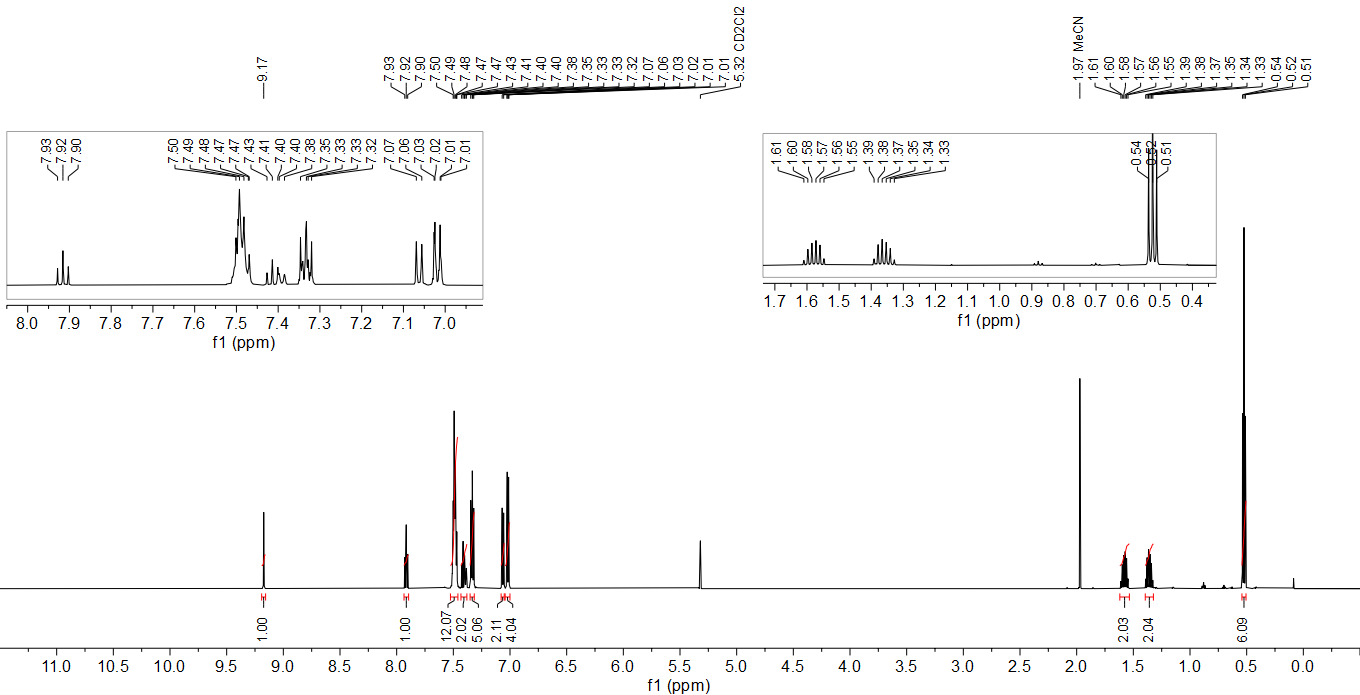


Figure S83: ^1^H NMR spectrum of phosphine isoindolium tetrafluoroborate **5b**.


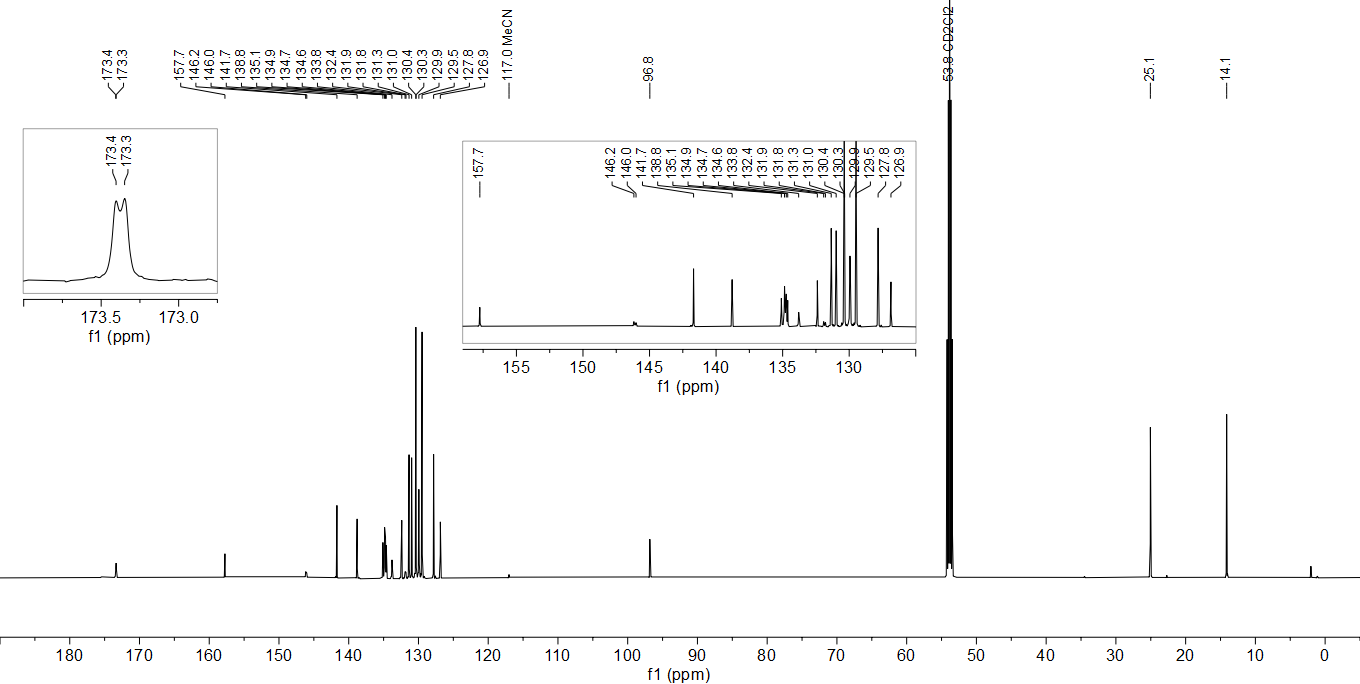


Figure S84: ^13^C{^1^H} NMR spectrum of phosphine isoindolium tetrafluoroborate **5b**.


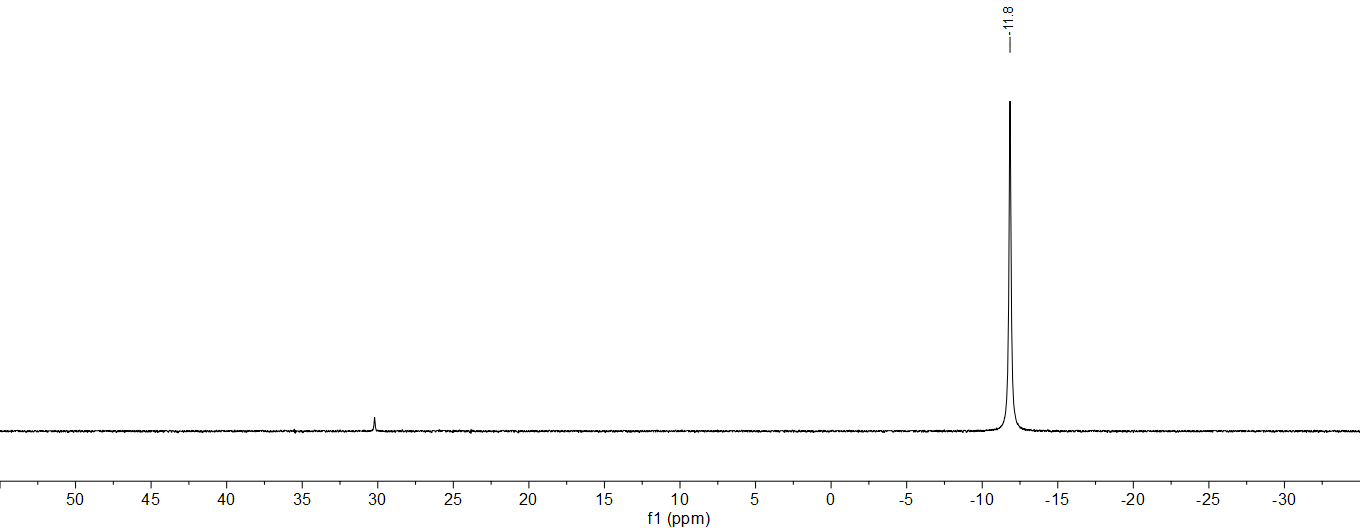


Figure S85: ^31^P{^1^H} NMR spectrum of phosphine isoindolium tetrafluoroborate **5b**.


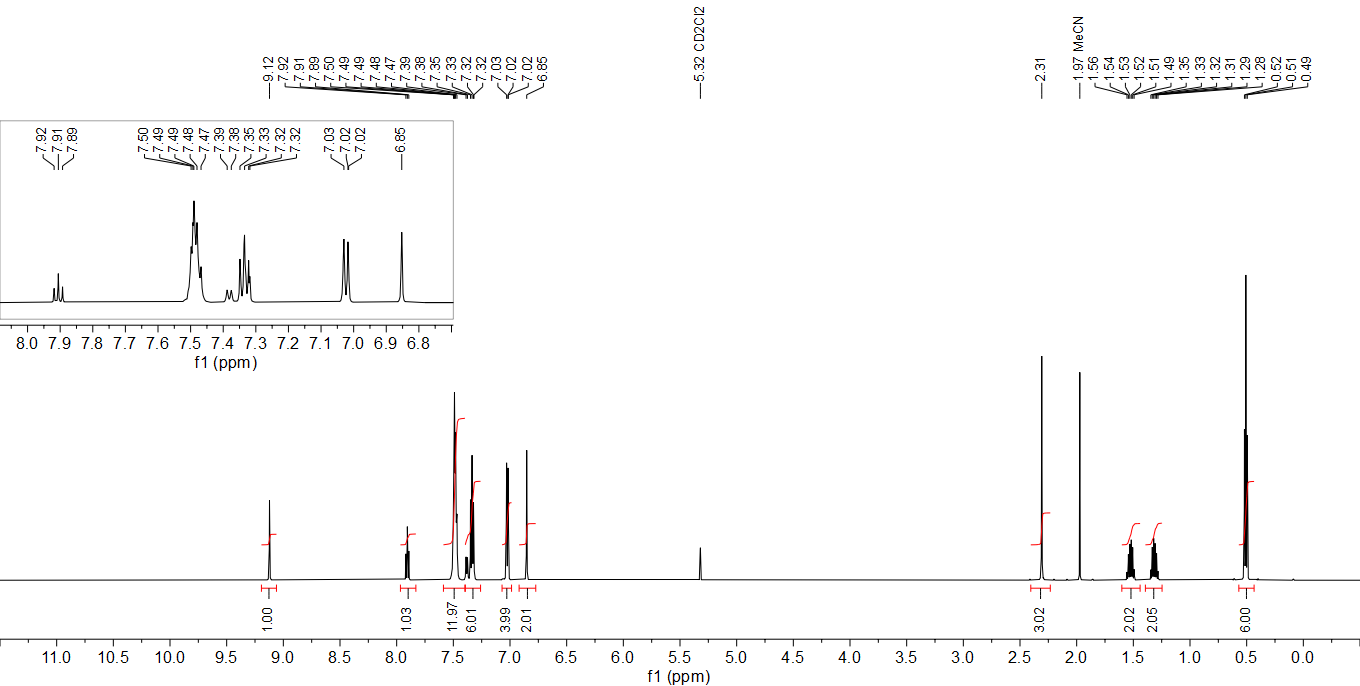


Figure S86: ^1^H NMR spectrum of phosphine isoindolium tetrafluoroborate **5c**.


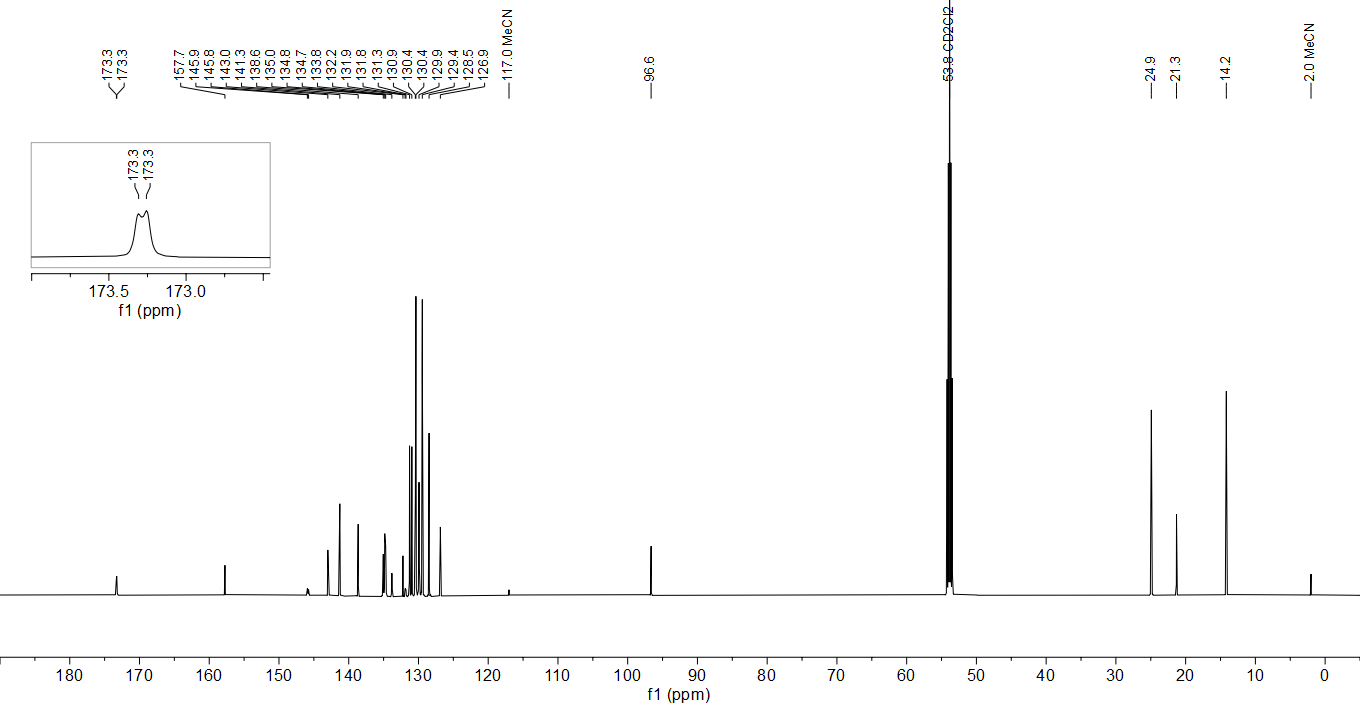


Figure S87: ^13^C{^1^H} NMR spectrum of phosphine isoindolium tetrafluoroborate **5c**.


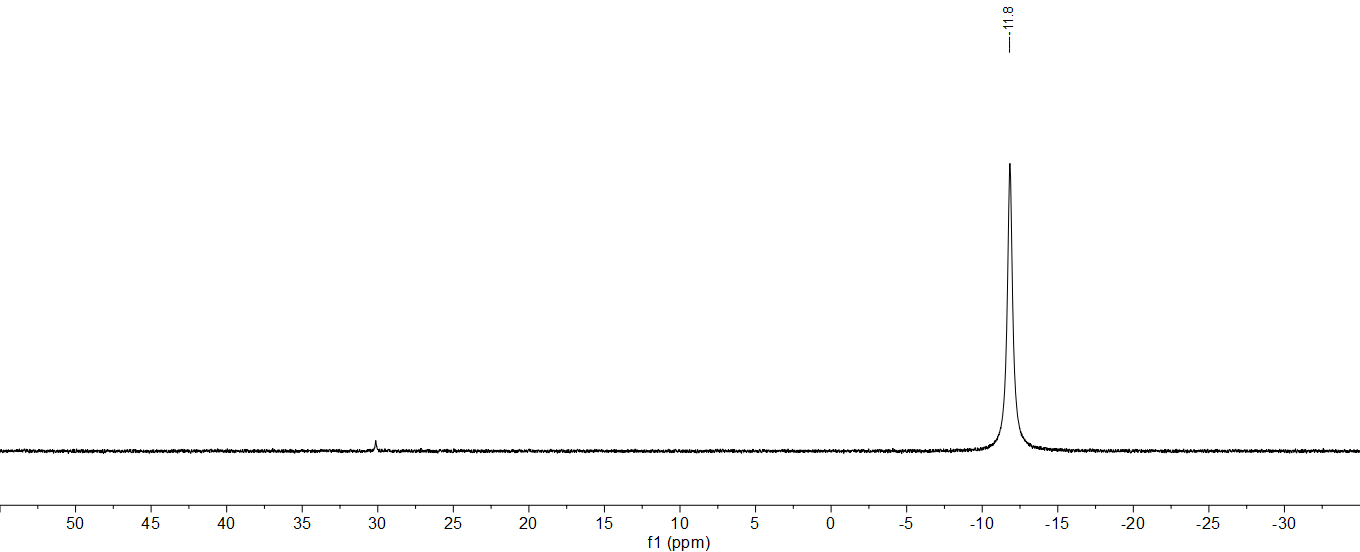


Figure S88: ^31^P{^1^H} NMR spectrum of phosphine isoindolium tetrafluoroborate **5c**.


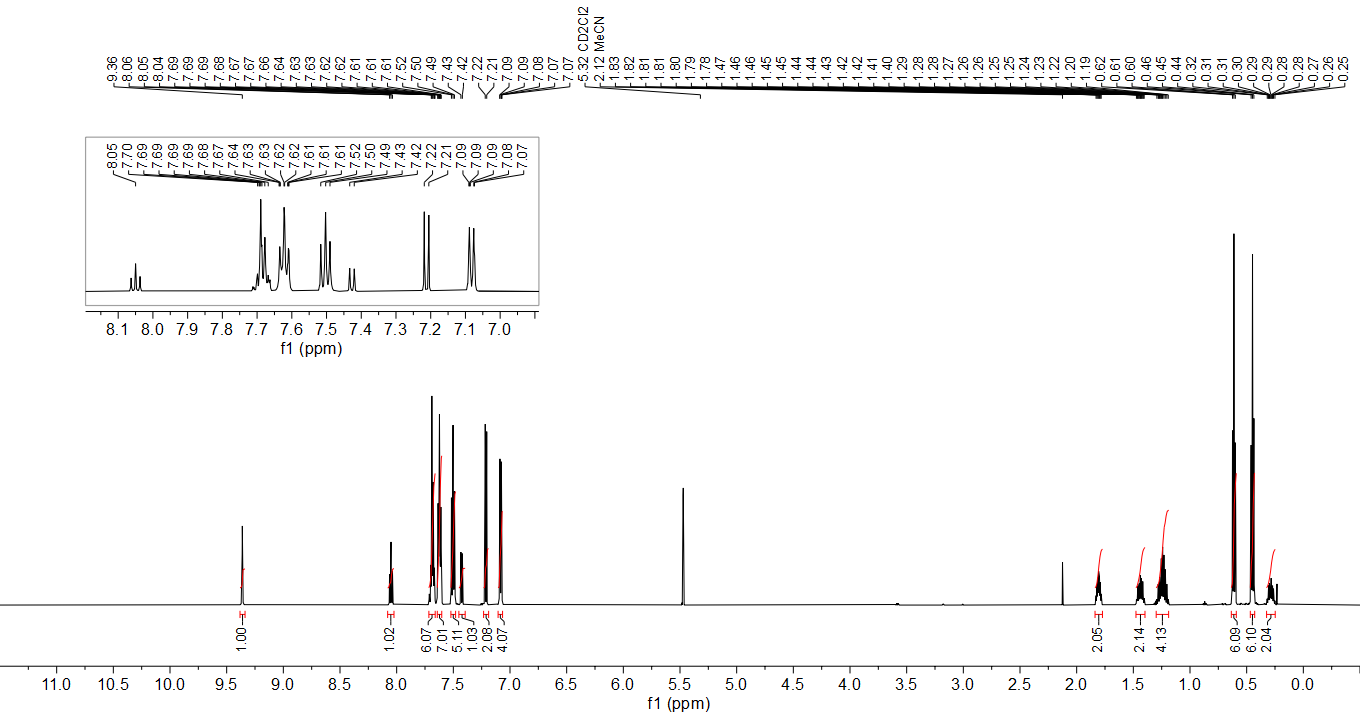


Figure S89: ^1^H NMR spectrum of phosphine isoindolium tetrafluoroborate **5d**.


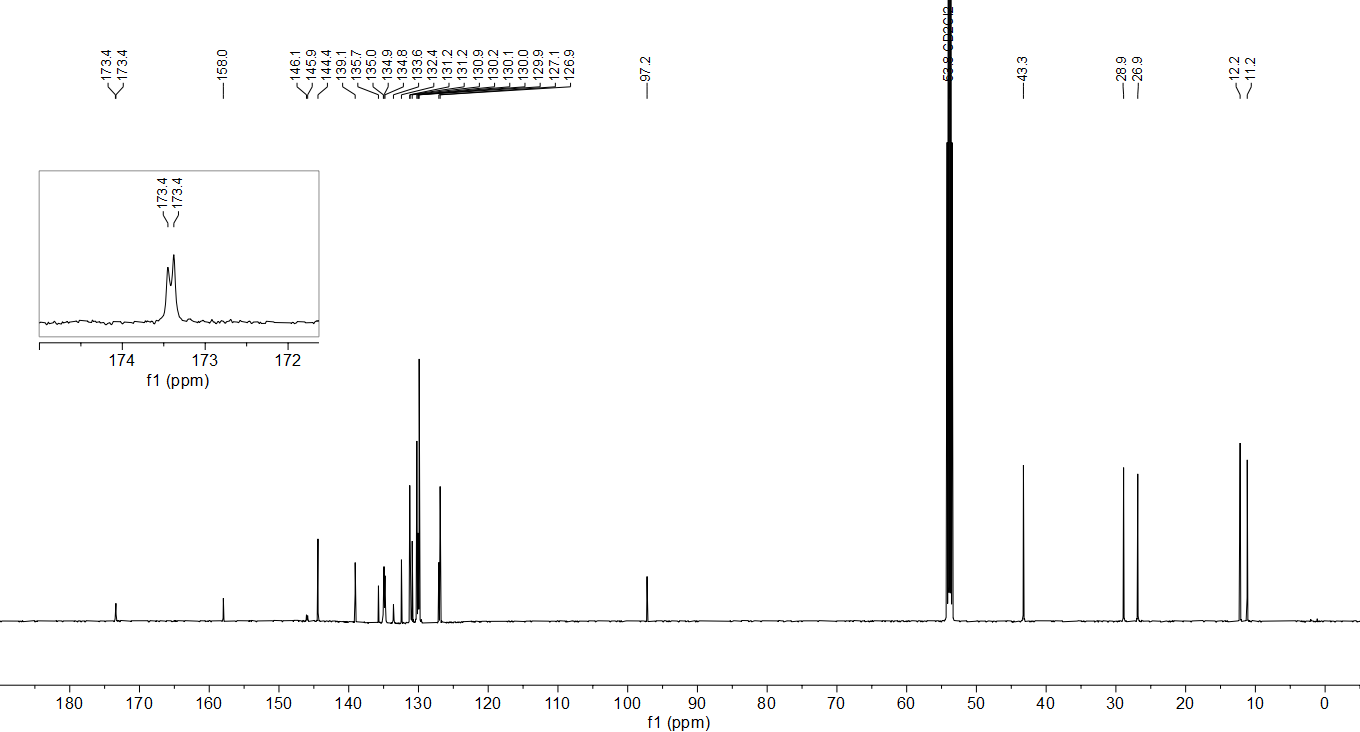


Figure S90: ^13^C{^1^H} NMR spectrum of phosphine isoindolium tetrafluoroborate **5d**.


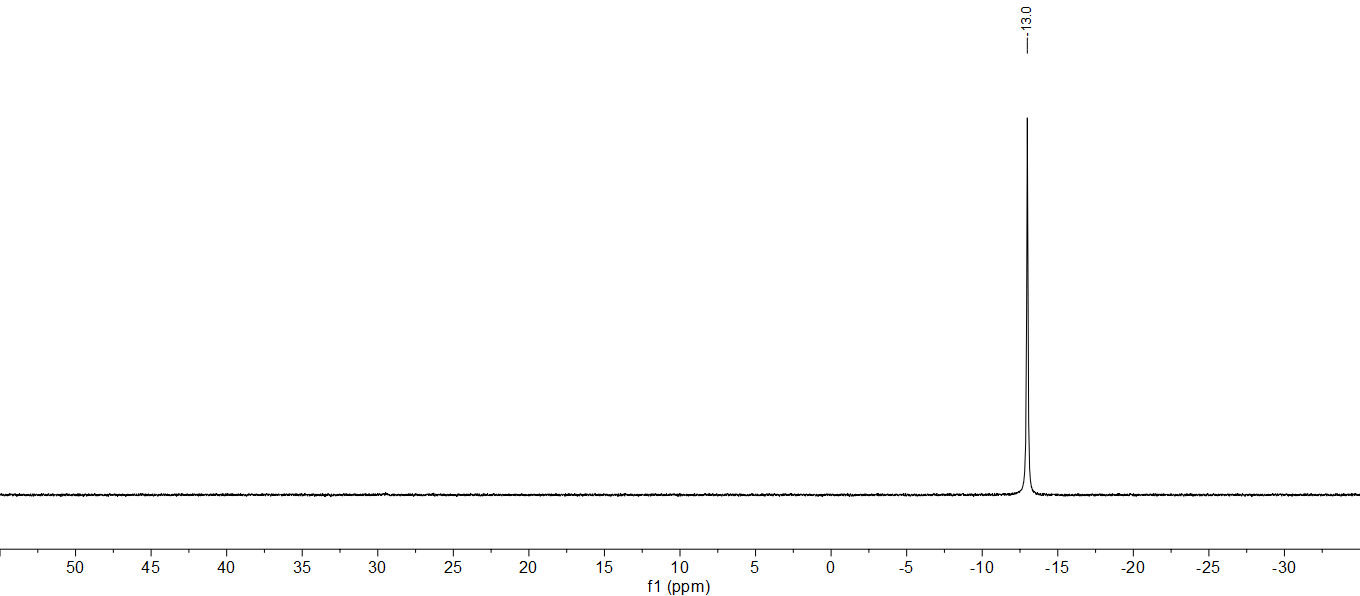
Figure S91: ^13^P{^1^H} NMR spectrum of phosphine isoindolium tetrafluoroborate **5d**.


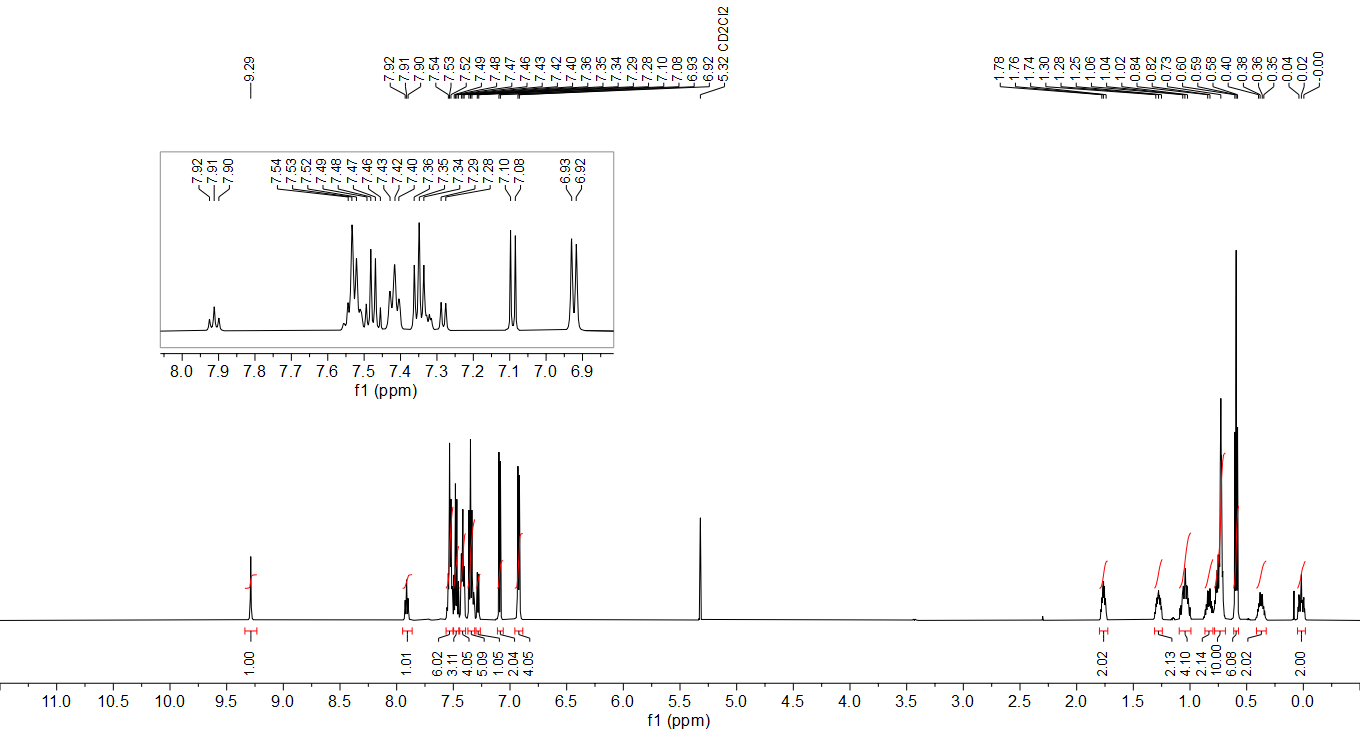


Figure S92: ^1^H NMR spectrum of phosphine isoindolium tetrafluoroborate **5e**.


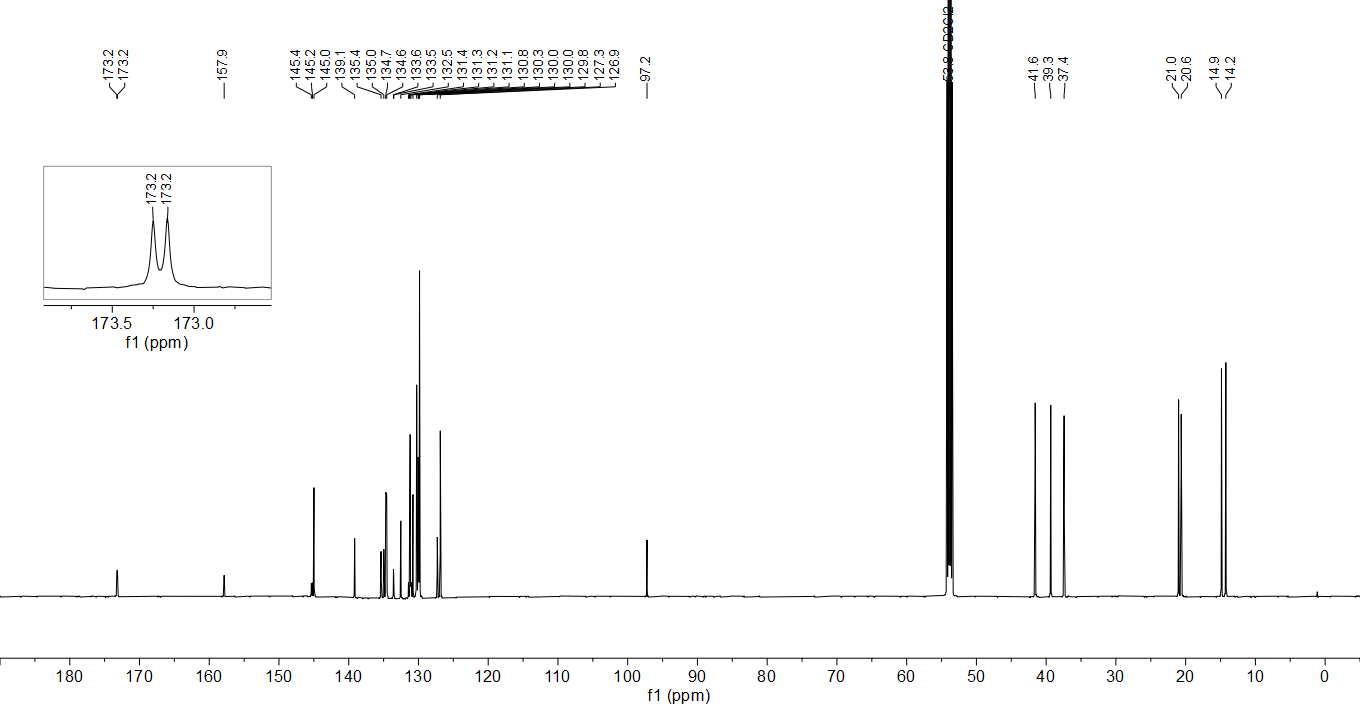


Figure S93: ^13^C{^1^H} NMR spectrum of phosphine isoindolium tetrafluoroborate **5e**.


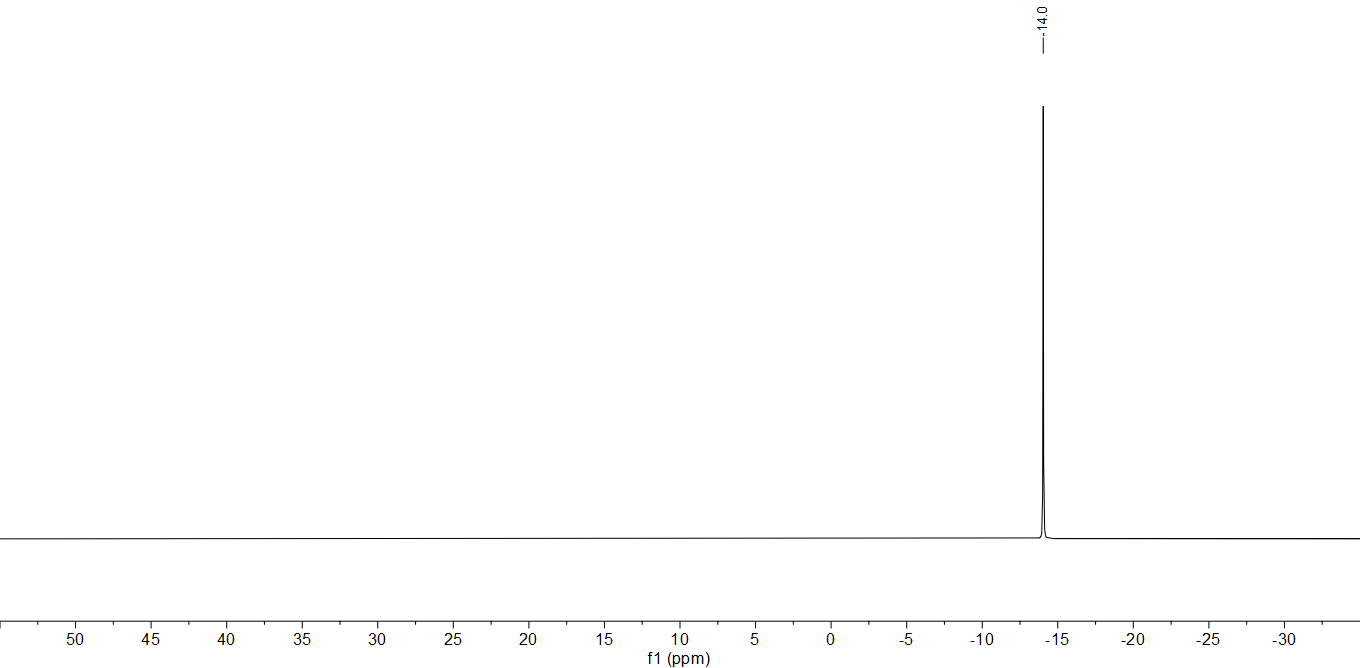


Figure S94: ^13^P{^1^H} NMR spectrum of phosphine isoindolium tetrafluoroborate **5e**.


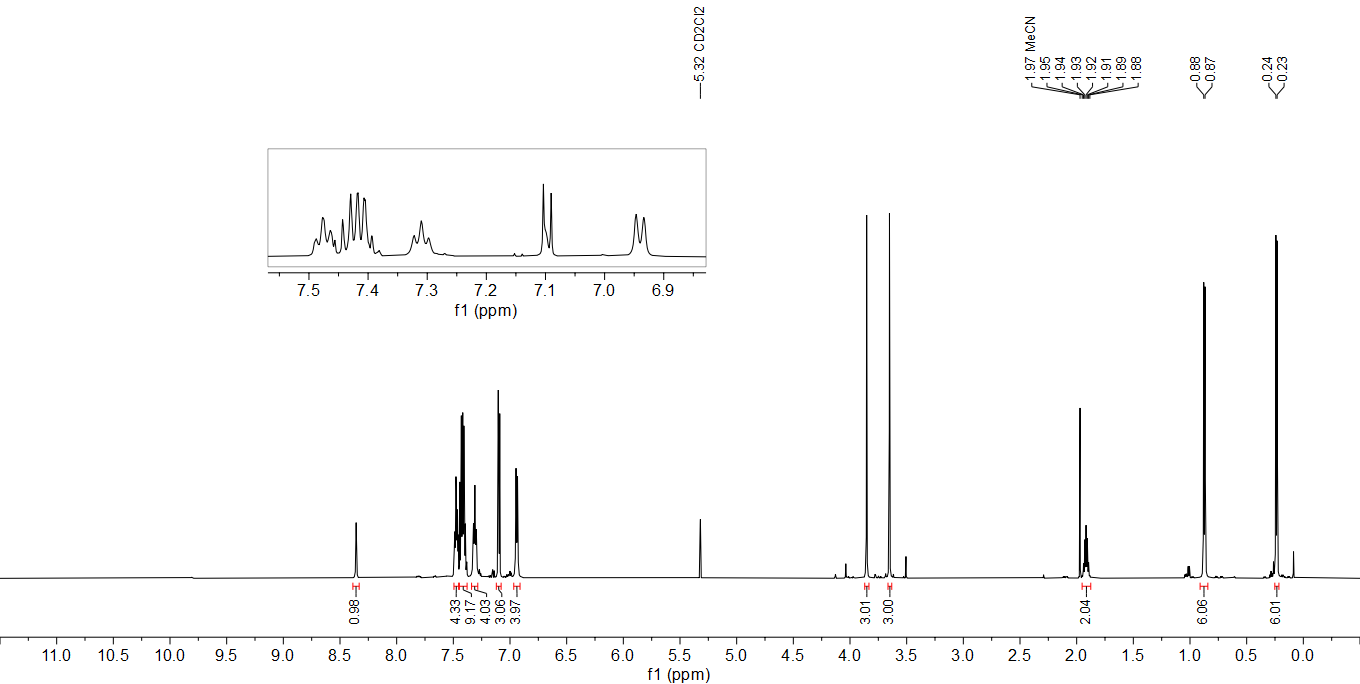


Figure S95: ^1^H NMR spectrum of phosphine isoindolium tetrafluoroborate **5f**.


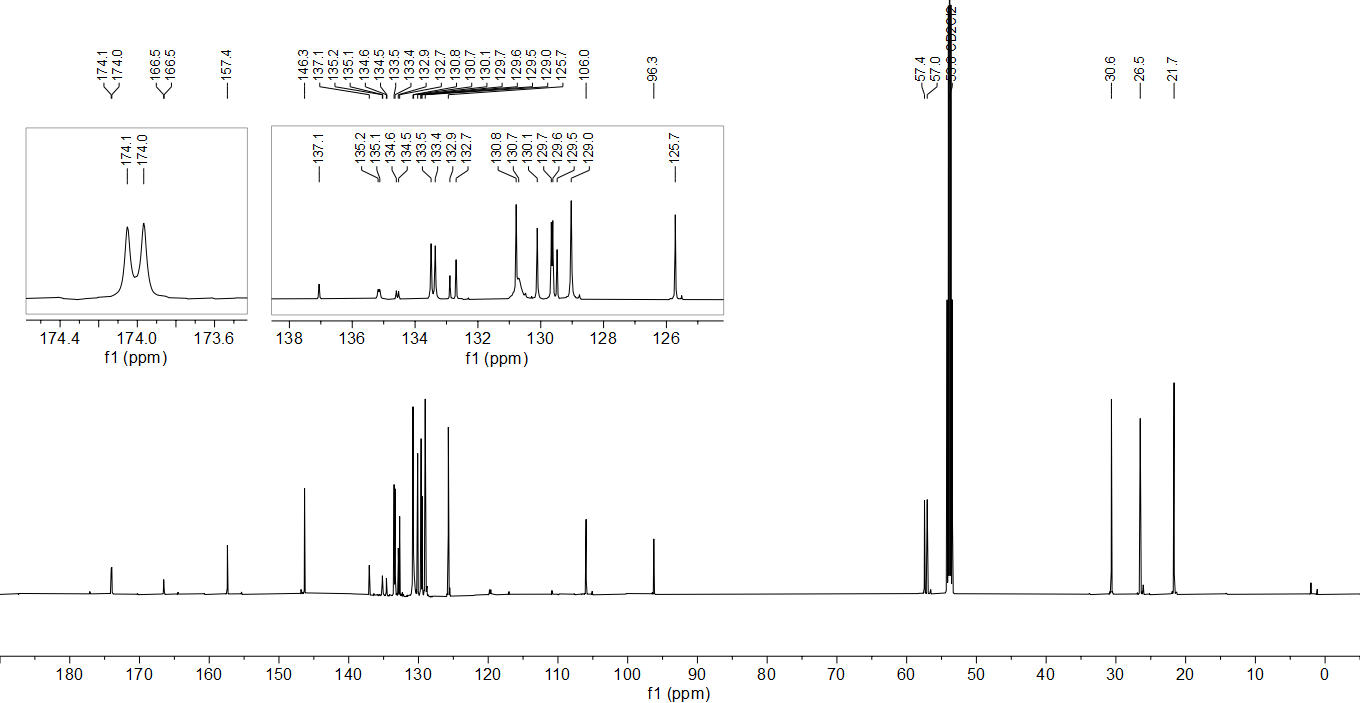


Figure S96: ^13^C{^1^H} NMR spectrum of phosphine isoindolium tetrafluoroborate **5f**.


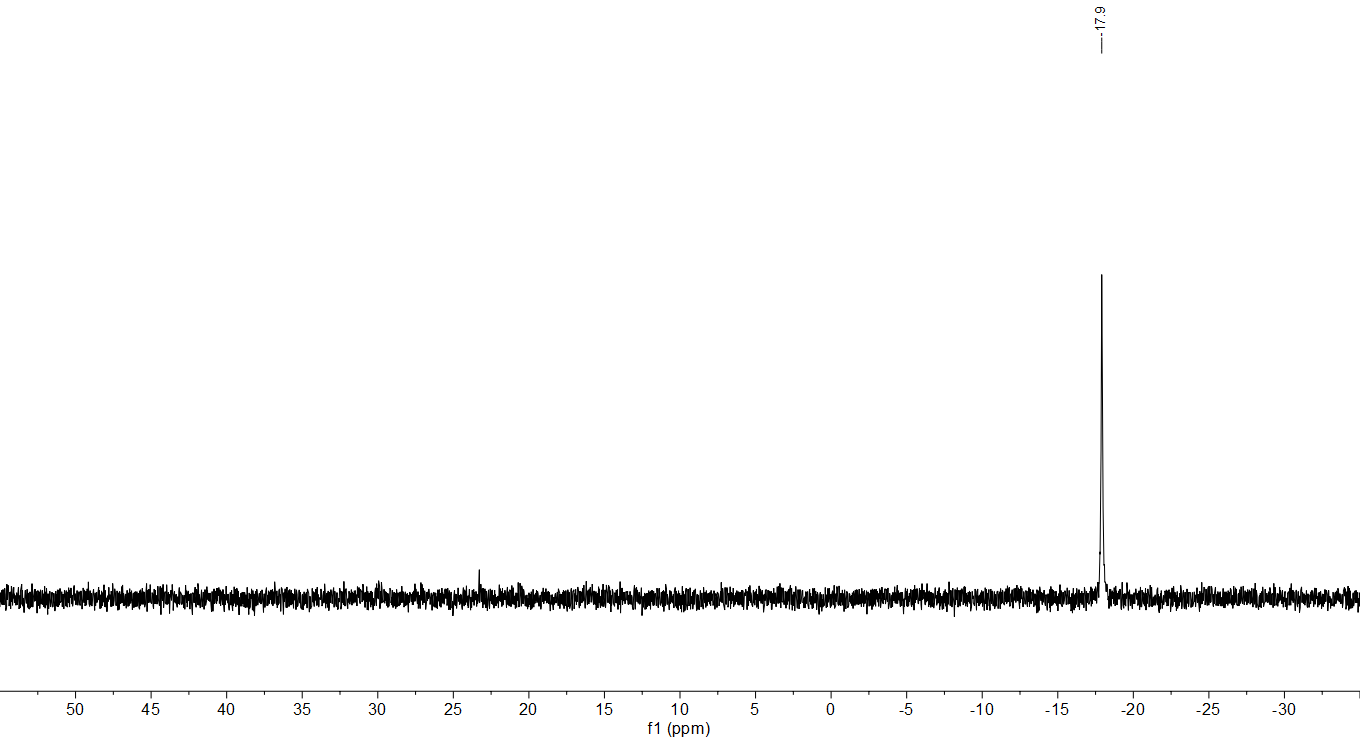


Figure S97: ^31^P{^1^H} NMR spectrum of phosphine isoindolium tetrafluoroborate **5f**.


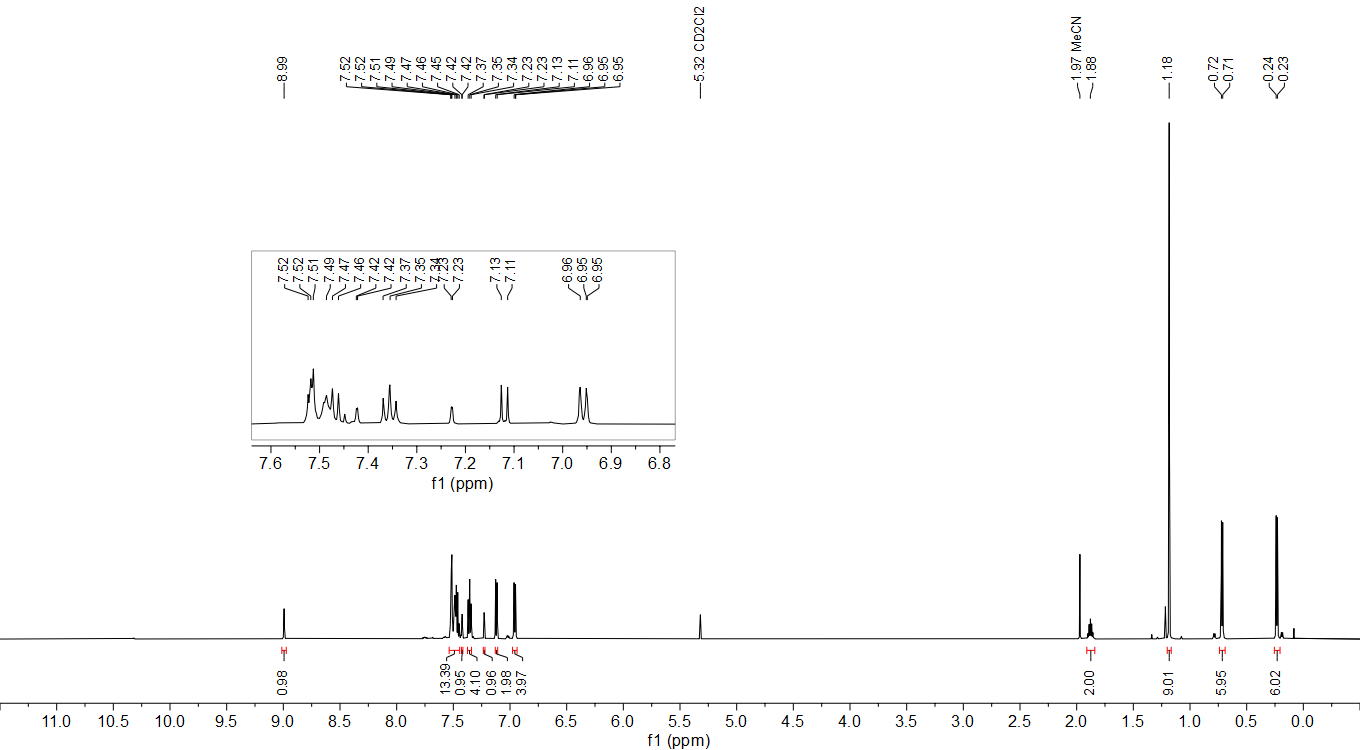


Figure S98: ^1^H NMR spectrum of phosphine isoindolium tetrafluoroborate **5g**.


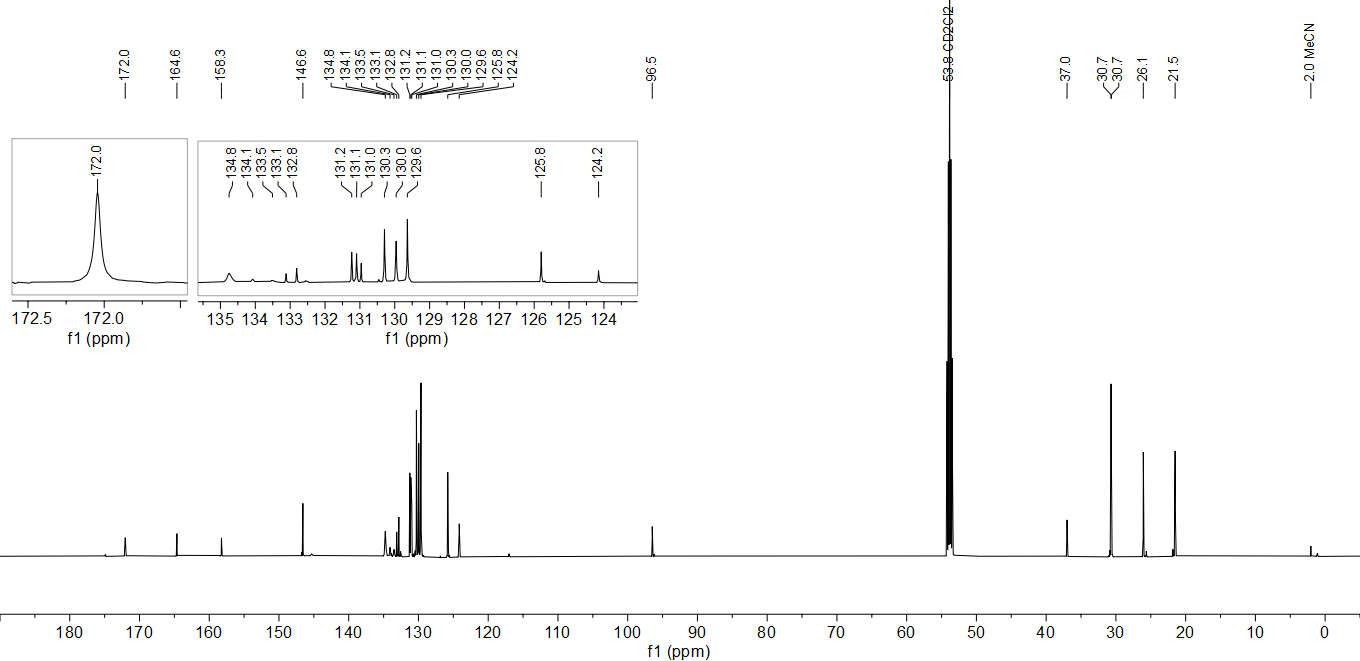


Figure S99: ^13^C{^1^H} NMR spectrum of phosphine isoindolium tetrafluoroborate **5g**.


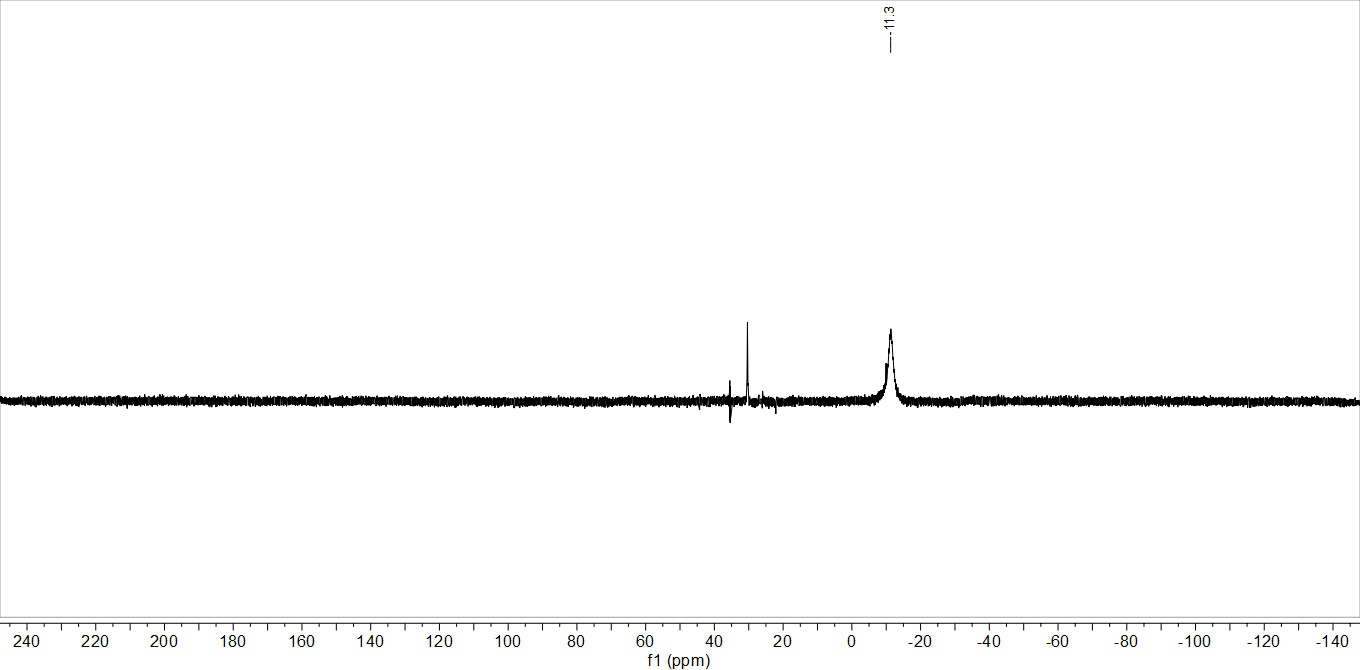


Figure S100: ^31^P{^1^H} NMR spectrum of phosphine isoindolium tetrafluoroborate **5g**.

Figure S101: ^1^H NMR spectrum of benzocyclobutenone imine-phosphine **6**.

Figure S102: ^13^C{^1^H} NMR spectrum of benzocyclobutenone imine-phosphine **6**.

Figure S103: ^31^P{^1^H} NMR spectrum of benzocyclobutenone imine-phosphine **6**.

Figure S104: ^1^H NMR spectrum of (CAArCPhos)PdCl_2_ **7a**.

Figure S105: ^13^C{^1^H} NMR spectrum of (CAArCPhos)PdCl_2_ **7a**.

Figure S106: ^31^P{^1^H} NMR spectrum of (CAArCPhos)PdCl_2_ **7a**.

Figure S107: ^1^H NMR spectrum of (CAArCPhos)PdCl_2_ **7b**.

Figure S108: ^13^C{^1^H} NMR spectrum of (CAArCPhos)PdCl_2_ **7b**.

Figure S109: ^31^P{^1^H} NMR spectrum of (CAArCPhos)PdCl_2_ **7b**.

Figure S110: ^1^H NMR spectrum of (CAArCPhos)PdCl_2_ **7c**.

Figure S111: ^13^C{^1^H} NMR spectrum of (CAArCPhos)PdCl_2_ **7c**.

Figure S112: ^31^P{^1^H} NMR spectrum of (CAArCPhos)PdCl_2_ **7c**.

Figure S113: ^1^H NMR spectrum of (CAArCPhos)PdCl_2_ **7d**.

Figure S114: ^13^C{^1^H} NMR spectrum of (CAArCPhos)PdCl_2_ **7d**.

Figure S115: ^31^P{^1^H} NMR spectrum of (CAArCPhos)PdCl2 **7d**.

Figure S116: ^1^H NMR spectrum of (CAArCPhos)PdCl_2_ **7f**.

Figure S117: ^13^C{^1^H} NMR spectrum of (CAArCPhos)PdCl_2_ **7f**.

Figure S118: ^31^P{^1^H} NMR spectrum of (CAArCPhos)PdCl2 **7f**.

Figure S119: ^1^H NMR spectrum of (CAArCPhos)PdCl_2_ **7g**.

Figure S120: ^13^C{^1^H} NMR spectrum of (CAArCPhos)PdCl_2_ **7g**.

Figure S121: ^31^P{^1^H} NMR spectrum of (CAArCPhos)PdCl2 **7g**.

1. **Density functional theory calculations**

**For quantum-chemical calculations** we used the Orca 5.0.4 program package.^[24]^ We used density fitting with the corresponding def2/J auxiliary bases^[25]^ and the RIJCOSX approximation^[26]^ in their default settings in Orca. The CPCM model was used to account for the polarity of the toluene solvent. We preoptimized structures and explored the reaction mechanism with the r2SCAN‑3c composite method.^[27]^ Transition states were localized by the nudged elastic band method.^[28]^ To obtain final electronic energies we reoptimized all structures with the wB97M-D4 hybrid density functional^[29]^ and def2-TZVP bases and the corresponding electronic core potential (ECP) on palladium.^[30]^ From these structures we obtained Gibbs energies at 384 K through Orcas default QRRHO-model^[31]^ from analytical frequencies calculated at the same level of theory as the electronic energies.

It seems energetically unfavorable for **INT1** to generate a free coordination site by chloride or acetate substitution and simultaneously form an acetate-proton bond since this would require a strong distortion of the square planar geometry of Pd(II) in **INT1**. However, this is not necessary as **TS1** has a reasonable energy of 25.5 kcal/mol and features only slightly distorted square planar palladium (Cl-Pd-O 149°). Instead Pd binds loosely (C-Pd A 2.42 A) by the side that is associated with an occupied dz^2^-orbital in square planar d8-Pd^II^. Considering the electrophilic nature of iminium ions or CAArC’s p-orbitals suggests the approach of palladium without major distortion from a square planar geometry is possible due to a Z-ligand interaction with the Pd-dz^2^-orbital instead of a more repulsive interaction that would be expected for a L-ligand with the Pd-dz^2^-orbital. The transition state is late with respect to the proton transfer (C-H 1.96 A, H-O 1.00 A) and more associated with the shortening Pd-C bond but still concerted. Due to this unusual feature, we performed additional nudged elastic band calculations zoomed in on the breakage of the C-H bond during deprotonation to see if we missed a transition state due insufficient number of images/too large step sizes. But these runs did only show a monotonously increasing energy on the reaction path as the deprotonation is happening.

We also performed the calculation with **INT1’** bearing the full substituents as **5a**. But in this case due to steric hinderance the end of deprotonation leads not to the transition state but rather the energy keeps rising until an almost flat energy region is reached. A first transition state **TS1’** in this region is associated with movement of the formed acetic acid away from the bulky Dipp group. After this has eased steric congestion and **INT2’** has been formed a second transition state **TS2**’ is associated with shortening the Pd-C distance (C-Pd A 2.45 A) leading to the product **7a**.

**XYZ coordinates of calculated stationary points**

**INT1**

C 0.416954 1.617811 -0.150216

P 1.847903 0.602821 0.279554

C 1.651355 -0.927557 -0.666415

C 5.038482 0.174720 0.919542

C 4.573625 1.130028 -0.060914

C 3.280471 1.422147 -0.498057

C 3.164083 2.416690 -1.454955

C 4.287553 3.095207 -1.933806

C 5.560771 2.818768 -1.462323

C 5.688963 1.821621 -0.512533

C 6.911935 1.345747 0.222672

C 7.962095 0.737357 -0.701236

C 7.491208 2.446835 1.109260

N 6.311510 0.282522 1.079281

C 7.092041 -0.533421 1.998112

H -0.457331 1.150880 0.300746

H 0.272509 1.680954 -1.227304

H 0.544862 2.608781 0.279031

H 0.767170 -1.447185 -0.302318

H 2.518557 -1.562662 -0.497513

H 1.547081 -0.707298 -1.727692

H 2.191448 2.704196 -1.825751

H 4.151176 3.871191 -2.674223

H 6.416280 3.374495 -1.820090

H 8.311594 1.505722 -1.387714

H 7.542592 -0.083653 -1.280048

H 8.816842 0.377917 -0.131219

H 7.832736 3.264126 0.477548

H 8.343048 2.074574 1.675663

H 6.735161 2.824628 1.795310

H 7.846619 -1.086725 1.444803

H 6.419337 -1.222964 2.497615

H 7.573114 0.106731 2.733164

Pd 2.194593 0.343036 2.472799

H 4.466326 -0.589535 1.447748

O 1.633805 -1.610179 2.377943

C 2.528957 -2.512312 2.202086

O 3.696430 -2.303751 1.848184

C 2.055957 -3.920808 2.483038

H 2.164262 -4.100391 3.553331

H 1.005527 -4.037280 2.229109

H 2.665576 -4.641852 1.945585

Cl 2.718479 0.044997 4.759404

Cl 2.696403 2.606455 2.490187

**TS1**

C -3.844728 1.494639 -0.598193

P -2.399871 0.452460 -0.328811

C -2.776007 -1.080683 -1.217327

C 0.475925 -0.076419 0.422925

C 0.233184 0.835998 -0.702529

C -1.005279 1.193371 -1.215130

C -1.034675 2.116651 -2.250201

C 0.159118 2.653943 -2.734432

C 1.392390 2.313884 -2.186605

C 1.414670 1.394352 -1.152897

C 2.531957 0.896137 -0.279112

C 3.574631 0.090228 -1.046927

C 3.164023 2.041704 0.507950

N 1.760620 -0.004620 0.641172

C 2.447202 -0.758012 1.680817

H -4.682770 1.062380 -0.053971

H -4.082925 1.527312 -1.660873

H -3.652357 2.491739 -0.214985

H -3.598569 -1.589552 -0.718982

H -1.901500 -1.727052 -1.208358

H -3.050516 -0.848345 -2.245683

H -1.973740 2.443215 -2.675156

H 0.119552 3.374947 -3.539447

H 2.297937 2.779017 -2.552816

H 4.059034 0.735685 -1.777888

H 3.109587 -0.743205 -1.571167

H 4.339948 -0.293740 -0.374022

H 3.614191 2.747594 -0.188203

H 3.946599 1.675014 1.169718

H 2.411175 2.562392 1.097142

H 2.992872 -1.593238 1.244451

H 1.703988 -1.116549 2.385425

H 3.145233 -0.110749 2.205678

Pd -1.572896 0.088095 1.703054

H -0.062906 -1.884953 0.954863

O -2.231877 -2.191588 1.652158

C -1.394656 -3.077609 1.570647

O -0.167792 -2.863894 1.136118

C -1.660973 -4.495186 1.961592

H -1.135102 -4.691773 2.896274

H -2.724992 -4.645160 2.108133

H -1.271356 -5.175401 1.207721

Cl -0.558927 -0.334239 3.816262

Cl -2.109425 2.331213 2.131615

**INT2**

C 0.745494 2.825544 0.327368

P 1.744712 1.322011 0.410165

C 0.733213 0.032769 -0.350701

C 4.447082 1.003858 1.385871

C 4.343242 1.384935 -0.025246

C 3.157127 1.595998 -0.696820

C 3.237856 1.956673 -2.035818

C 4.490636 2.083710 -2.637537

C 5.676813 1.850025 -1.940874

C 5.587318 1.492142 -0.608646

C 6.612229 1.138312 0.430101

C 7.391712 -0.111429 0.027345

C 7.537105 2.305447 0.761647

N 5.724455 0.848710 1.612985

C 6.346876 0.487726 2.879515

H -0.129432 2.690576 0.958847

H 0.440073 3.015999 -0.700943

H 1.329196 3.663613 0.701577

H -0.124423 -0.148473 0.293873

H 1.316298 -0.881285 -0.436136

H 0.397373 0.352100 -1.336664

H 2.345174 2.139643 -2.618571

H 4.542776 2.367291 -3.679846

H 6.630178 1.948107 -2.442498

H 7.921022 0.090530 -0.902374

H 6.717087 -0.950869 -0.131555

H 8.129036 -0.380823 0.780486

H 8.118059 2.562946 -0.122198

H 8.228611 2.035056 1.557797

H 6.961950 3.176851 1.069460

H 7.247034 -0.086591 2.682791

H 5.647176 -0.090430 3.467389

H 6.606248 1.386591 3.435163

Pd 2.708943 0.885188 2.388521

Cl 3.575573 0.616785 4.583582

Cl 0.462816 0.781957 3.139999

**acetic acid**

**INT1’**

H -4.480294 -1.513404 -0.389772

P -2.440264 0.454192 -0.101430

C -3.791461 -1.669775 -1.207846

C 0.729456 -0.321729 0.179783

C 0.298793 0.911172 -0.432505

C -0.989111 1.390420 -0.690717

C -1.079877 2.634676 -1.284260

C 0.068128 3.377689 -1.577832

C 1.334581 2.910080 -1.278187

C 1.437170 1.648503 -0.717711

C 2.658276 0.949787 -0.167745

H 4.324555 -3.113094 3.166279

H 1.984128 -1.296201 -1.906041

N 2.013200 -0.342292 0.320262

C 2.740004 -1.556171 0.646217

C -4.023961 -2.681776 -2.121470

H -1.327244 -2.241480 -4.117396

C -1.779400 -1.069974 -2.383602

H -4.897485 -3.309782 -2.014397

H -0.904768 -0.446589 -2.502042

C -2.663740 -0.857549 -1.333504

H -2.048022 3.064699 -1.492302

H -0.045059 4.355168 -2.025611

H 2.211069 3.514138 -1.463841

H -2.102065 -4.993118 1.747033

C -2.608861 -4.329109 1.047576

H -3.675407 -4.336580 1.250093

H -2.414613 -4.693448 0.040236

Cl -1.700690 -0.876630 4.247079

Cl -1.413558 1.967848 2.465514

C 3.962600 -2.911507 2.168017

C 2.778078 -0.932427 3.136361

C 3.174977 -1.785080 1.950590

Pd -2.115290 -0.199875 2.032520

H 0.120339 -1.181322 0.475626

O -2.833066 -2.054857 1.638253

C -2.023328 -2.942843 1.191358

O -0.843371 -2.742329 0.879946

H 4.089737 4.197635 3.918789

C 3.817272 3.538544 3.106004

C 2.560629 2.947262 3.075510

H 1.839520 3.143439 3.856714

C 2.215008 2.106976 2.034555

H 1.216196 1.693174 2.023026

C 3.124276 1.819861 1.019218

H 5.685354 3.759641 2.079079

C 4.365706 2.440026 1.034836

H 5.074767 2.279428 0.238880

C 4.711012 3.290630 2.079211

H 6.703234 0.039451 -3.615317

C 5.880799 0.203249 -2.932714

C 4.708211 0.787449 -3.380713

H 4.605650 1.078907 -4.416780

C 3.654528 1.003099 -2.501937

H 2.748096 1.457086 -2.872507

C 3.766013 0.647866 -1.164186

H 6.894621 -0.651734 -1.243838

C 4.941234 0.037556 -0.726754

H 5.049060 -0.248283 0.309477

C 5.989244 -0.182915 -1.603230

H 4.876908 -4.654823 1.334157

C 4.263720 -3.784966 1.141367

C 3.747366 -3.571224 -0.124727

H 3.945598 -4.287713 -0.908782

C 2.970014 -2.455665 -0.402956

C 2.350742 -2.313692 -1.781376

C 1.156493 -3.265161 -1.906255

C 3.353234 -2.566132 -2.906354

H 0.701776 -3.165480 -2.891536

H 1.488564 -4.297948 -1.790091

H 0.396492 -3.072869 -1.151137

H 4.247627 -1.960007 -2.788300

H 3.649983 -3.614191 -2.944985

H 2.897016 -2.320847 -3.865510

H 2.087860 -0.168541 2.794841

C 2.026140 -1.773893 4.170805

C 3.988421 -0.241547 3.762957

H 4.510391 0.385288 3.040783

H 3.669742 0.389997 4.591371

H 4.693972 -0.976952 4.152086

H 1.163686 -2.267208 3.726196

H 2.674842 -2.532505 4.610247

H 1.661605 -1.132812 4.972272

H -6.891879 3.856953 -0.969622

C -6.042036 3.208213 -0.806068

C -5.550605 3.020067 0.476675

H -6.014525 3.518831 1.316158

C -4.462800 2.185508 0.690432

H -4.075467 2.040172 1.688316

C -3.863546 1.537341 -0.384658

H -5.836100 2.697902 -2.881993

C -4.366323 1.721678 -1.672667

H -3.915592 1.206946 -2.510555

C -5.449698 2.558145 -1.881821

H -3.319534 -3.682959 -3.883971

C -3.136177 -2.891334 -3.170330

C -2.018307 -2.084602 -3.300921

**TS1’**

H -5.307304 -1.060319 -0.534662

P -3.172929 0.802537 -0.148265

C -4.600381 -1.235385 -1.334672

C -0.155184 -0.053833 0.274896

C -0.467656 1.224829 -0.396900

C -1.726263 1.742721 -0.700256

C -1.795829 2.972494 -1.333589

C -0.628533 3.669835 -1.648036

C 0.620169 3.165198 -1.324989

C 0.682780 1.925046 -0.711283

C 1.867022 1.211258 -0.113356

H 3.542425 -2.774409 3.251629

H 1.138442 -1.015040 -1.796696

N 1.151145 -0.038449 0.408440

C 1.882051 -1.241051 0.752197

C -4.858839 -2.210499 -2.280373

H -2.083042 -1.860783 -4.185056

C -2.525213 -0.711682 -2.433164

H -5.767076 -2.793806 -2.216630

H -1.613550 -0.135651 -2.498500

C -3.431770 -0.476657 -1.408538

H -2.753038 3.416152 -1.564310

H -0.706711 4.633397 -2.132377

H 1.516243 3.733943 -1.532941

H -3.821867 -4.432988 0.759932

C -2.794521 -4.184074 0.509474

H -2.742519 -4.004805 -0.565181

H -2.120761 -4.991586 0.775105

Cl -2.147785 -0.891007 4.019267

Cl -2.365233 2.146947 2.534926

C 3.142063 -2.596387 2.263447

C 2.080002 -0.556895 3.225574

C 2.371006 -1.457312 2.042600

Pd -2.756594 -0.020547 1.902016

H -1.036137 -2.035654 2.125628

O -3.198831 -1.968838 1.244682

C -2.413457 -2.915744 1.195101

O -1.205571 -2.897678 1.690296

H 3.268614 4.638533 3.834868

C 3.003364 3.943052 3.050296

C 1.762274 3.317680 3.057967

H 1.048068 3.525147 3.842973

C 1.425853 2.429834 2.054027

H 0.442531 1.980016 2.066653

C 2.328116 2.126848 1.036201

H 4.851732 4.173106 1.990510

C 3.551752 2.781633 1.014149

H 4.255093 2.603766 0.215784

C 3.889628 3.679864 2.020856

H 5.934666 0.043661 -3.464308

C 5.110760 0.264592 -2.799550

C 3.954833 0.848524 -3.289128

H 3.866609 1.083601 -4.341018

C 2.898887 1.134239 -2.432799

H 2.004510 1.584723 -2.836074

C 2.986954 0.852684 -1.075386

H 6.093063 -0.508525 -1.053575

C 4.149765 0.249611 -0.595969

H 4.243100 0.021684 0.456005

C 5.200008 -0.043832 -1.448224

H 3.988209 -4.385787 1.451830

C 3.387732 -3.507437 1.256295

C 2.836433 -3.306676 0.003376

H 2.998634 -4.041405 -0.772416

C 2.078560 -2.177596 -0.274116

C 1.448731 -2.046672 -1.648611

C 0.197738 -2.925162 -1.722824

C 2.416760 -2.395014 -2.778392

H -0.241376 -2.879780 -2.719115

H 0.449556 -3.964780 -1.507344

H -0.547420 -2.592180 -1.003706

H 3.354132 -1.852506 -2.679371

H 2.637804 -3.462362 -2.803038

H 1.970740 -2.132836 -3.738314

H 1.334858 0.170811 2.920866

C 1.486501 -1.350410 4.392894

C 3.336755 0.184094 3.684489

H 3.766890 0.785989 2.886533

H 3.099370 0.846754 4.516320

H 4.093077 -0.526009 4.023331

H 0.622275 -1.934252 4.084365

H 2.223242 -2.025804 4.829219

H 1.158279 -0.664904 5.173708

H -7.740636 4.089841 -0.851331

C -6.867797 3.465995 -0.714225

C -6.428264 3.160050 0.564349

H -6.956195 3.541672 1.427172

C -5.308592 2.359846 0.744142

H -4.961425 2.126101 1.739448

C -4.625199 1.863194 -0.360203

H -6.537797 3.196237 -2.819355

C -5.079445 2.161948 -1.645500

H -4.566634 1.760214 -2.509299

C -6.192739 2.965681 -1.820896

H -4.157098 -3.195736 -4.053717

C -3.952648 -2.436620 -3.310929

C -2.789944 -1.688217 -3.385323

**INT2’**

H -5.332622 -0.979612 -0.678854

P -3.147089 0.790228 -0.152092

C -4.592798 -1.169839 -1.444835

C -0.128795 -0.041743 0.263267

C -0.447787 1.242085 -0.394296

C -1.710265 1.757306 -0.680620

C -1.790737 2.995719 -1.296342

C -0.628762 3.700220 -1.614001

C 0.625131 3.195297 -1.310724

C 0.697493 1.949947 -0.709408

C 1.889611 1.232561 -0.132318

H 3.633277 -2.634969 3.285655

H 1.123342 -1.069946 -1.776988

N 1.176238 -0.017161 0.399445

C 1.920604 -1.204051 0.760859

C -4.839566 -2.120884 -2.417522

H -1.956205 -1.855573 -4.170159

C -2.445513 -0.712036 -2.427311

H -5.771373 -2.669390 -2.409027

H -1.510803 -0.170621 -2.438699

C -3.394557 -0.455432 -1.447537

H -2.751255 3.439147 -1.512766

H -0.714928 4.669213 -2.086006

H 1.517507 3.768237 -1.523340

H -3.558172 -4.817980 0.727056

C -2.756375 -4.196734 0.326126

H -3.050425 -3.832252 -0.653432

H -1.851676 -4.794228 0.268647

Cl -2.387220 -0.985271 4.068248

Cl -2.361442 2.057660 2.561798

C 3.226314 -2.488739 2.294589

C 2.072714 -0.479687 3.219663

C 2.418443 -1.380047 2.052780

Pd -2.791073 -0.087145 1.885459

H -1.748268 -2.493607 2.870774

O -3.204371 -2.001747 1.103893

C -2.561311 -3.043284 1.253315

O -1.701488 -3.243106 2.211692

H 3.397483 4.664128 3.773547

C 3.112287 3.966460 2.997965

C 1.850550 3.383364 3.008812

H 1.141170 3.626027 3.788085

C 1.488586 2.493489 2.015989

H 0.491136 2.075086 2.029727

C 2.383799 2.145214 1.005062

H 4.971792 4.117384 1.944133

C 3.629274 2.756899 0.981490

H 4.329126 2.547308 0.188079

C 3.993319 3.657652 1.977250

H 5.840527 -0.005490 -3.598123

C 5.040890 0.230911 -2.909434

C 3.875264 0.821097 -3.367592

H 3.755003 1.045942 -4.418557

C 2.851051 1.127466 -2.480422

H 1.949202 1.584280 -2.859301

C 2.979924 0.859102 -1.123332

H 6.070982 -0.535132 -1.188085

C 4.151120 0.247112 -0.676041

H 4.274762 0.025094 0.374126

C 5.170731 -0.064454 -1.558570

H 4.126854 -4.264921 1.512341

C 3.495900 -3.411251 1.303756

C 2.924383 -3.258704 0.052427

H 3.100025 -4.007891 -0.706423

C 2.128024 -2.161087 -0.243505

C 1.454291 -2.090950 -1.600917

C 0.214200 -2.988731 -1.588619

C 2.384476 -2.470766 -2.751454

H -0.276673 -2.978582 -2.561434

H 0.494131 -4.017857 -1.357549

H -0.498433 -2.644914 -0.841023

H 3.313346 -1.905943 -2.713974

H 2.627221 -3.533615 -2.738988

H 1.895946 -2.260505 -3.703489

H 1.360740 0.261510 2.874286

C 1.383144 -1.279864 4.327761

C 3.304384 0.240973 3.765819

H 3.796213 0.834024 2.996586

H 3.018992 0.910082 4.577201

H 4.026611 -0.475970 4.159602

H 0.518366 -1.817274 3.944810

H 2.065594 -2.000375 4.780292

H 1.035376 -0.606939 5.111381

H -7.737565 4.061784 -0.785426

C -6.860556 3.441191 -0.660715

C -6.426959 3.101105 0.611133

H -6.963622 3.452694 1.481286

C -5.302025 2.304746 0.774967

H -4.960624 2.044524 1.765628

C -4.607340 1.845599 -0.338463

H -6.513700 3.236526 -2.770563

C -5.055637 2.179178 -1.617504

H -4.534102 1.807351 -2.489350

C -6.173877 2.979165 -1.776868

H -4.083973 -3.110412 -4.166325

C -3.889883 -2.369358 -3.402805

C -2.697019 -1.666024 -3.405678

**TS2’**

H -5.384743 -0.650522 -1.310016

P -3.006992 0.678116 -0.292057

C -4.573556 -0.874328 -1.988405

C -0.116863 0.104837 0.360149

C -0.397906 1.315886 -0.417344

C -1.654567 1.778996 -0.779689

C -1.731813 2.966954 -1.487564

C -0.561089 3.652961 -1.817296

C 0.692054 3.180737 -1.450329

C 0.758790 1.987000 -0.752205

C 1.925872 1.298703 -0.088743

H 3.519358 -2.382816 3.653844

H 1.042312 -1.122486 -1.513555

N 1.178867 0.117013 0.560406

C 1.866249 -1.071868 1.027116

C -4.796750 -1.702916 -3.074745

H -1.680665 -1.710847 -4.396080

C -2.268980 -0.644026 -2.637248

H -5.782311 -2.115643 -3.241062

H -1.279477 -0.241780 -2.471945

C -3.309080 -0.332496 -1.768182

H -2.685901 3.381942 -1.776704

H -0.636965 4.583254 -2.363265

H 1.584005 3.743333 -1.690217

H -4.022166 -5.329708 -0.042119

C -3.557154 -4.482748 -0.542012

H -4.269434 -3.967003 -1.176206

H -2.734050 -4.862497 -1.147421

Cl -1.456019 -1.895205 3.217834

Cl -2.130669 1.519916 2.596160

C 3.093642 -2.304755 2.663374

C 2.242342 -0.070185 3.369177

C 2.395517 -1.150896 2.318389

Pd -2.240898 -0.558449 1.381150

H -1.890741 -3.426305 2.012503

O -3.236004 -2.335060 0.438129

C -2.994028 -3.537435 0.470443

O -2.218495 -4.107311 1.357376

H 3.509880 5.121675 3.386849

C 3.208333 4.344311 2.698068

C 1.952791 3.758118 2.810538

H 1.266884 4.078766 3.582583

C 1.567526 2.766092 1.928967

H 0.576015 2.339836 2.015023

C 2.439418 2.319133 0.937344

H 5.034816 4.396057 1.578490

C 3.677713 2.931268 0.807647

H 4.355519 2.635777 0.021819

C 4.062278 3.935959 1.688894

H 5.833299 -0.420502 -3.392419

C 5.041972 -0.087627 -2.734788

C 3.898448 0.493562 -3.255738

H 3.787586 0.615487 -4.324530

C 2.884619 0.923302 -2.408806

H 2.000155 1.372152 -2.835498

C 3.003256 0.787819 -1.031296

H 6.044578 -0.708176 -0.940191

C 4.152471 0.187141 -0.518133

H 4.270011 0.069310 0.548908

C 5.160950 -0.247939 -1.360348

H 3.777298 -4.243491 2.071103

C 3.235172 -3.354442 1.778000

C 2.658319 -3.274238 0.523763

H 2.744357 -4.110734 -0.155206

C 1.967827 -2.138717 0.121404

C 1.319588 -2.141223 -1.252046

C 0.038925 -2.978237 -1.217239

C 2.262733 -2.643627 -2.344912

H -0.421942 -3.013314 -2.204645

H 0.259062 -3.999763 -0.904147

H -0.674326 -2.547257 -0.518450

H 3.215049 -2.118593 -2.320625

H 2.456709 -3.711915 -2.247543

H 1.809328 -2.482818 -3.323729

H 1.527200 0.655238 2.997927

C 1.680290 -0.618209 4.683204

C 3.574921 0.633062 3.634598

H 4.008200 1.048765 2.727807

H 3.434231 1.449088 4.343166

H 4.292465 -0.067844 4.064774

H 0.751389 -1.157624 4.521688

H 2.391099 -1.283747 5.174415

H 1.482122 0.210282 5.364184

H -7.816412 3.617417 0.261813

C -6.895759 3.058871 0.160601

C -6.352790 2.412401 1.260077

H -6.847301 2.462842 2.220064

C -5.169658 1.697946 1.135813

H -4.736598 1.204236 1.992551

C -4.529205 1.628160 -0.096084

H -6.691934 3.475910 -1.936671

C -5.089962 2.262174 -1.206208

H -4.622650 2.176534 -2.177779

C -6.264424 2.982533 -1.074784

H -3.932251 -2.651658 -4.793847

C -3.756924 -2.005220 -3.944655

C -2.494357 -1.476672 -3.723406

**7-red**

H -5.548978 -0.684662 -1.482284

P -2.924665 0.355168 -0.750347

C -4.894666 -0.813484 -2.332125

C -0.186280 -0.001849 0.140080

C -0.403343 1.143692 -0.743029

C -1.623224 1.506053 -1.278633

C -1.659695 2.624168 -2.097779

C -0.482575 3.334965 -2.335774

C 0.738834 2.951964 -1.786223

C 0.770290 1.815417 -0.998687

C 1.868138 1.175476 -0.186622

H 3.323981 -2.409204 3.665270

H 1.209122 -1.209125 -1.674593

N 1.097200 -0.015650 0.417328

C 1.802596 -1.174964 0.930935

C -5.360706 -1.436302 -3.478912

H -2.535408 -1.341554 -5.342265

C -2.728338 -0.564898 -3.357778

H -6.384348 -1.781425 -3.521580

H -1.698733 -0.236225 -3.312264

C -3.577512 -0.366657 -2.272096

H -2.585284 2.952362 -2.549424

H -0.519895 4.211283 -2.968293

H 1.631202 3.531545 -1.977578

C -4.228496 1.389397 -0.052833

H -6.617857 3.605346 -0.970540

C -5.043436 2.159072 -0.880482

H -4.954932 2.083630 -1.955891

Cl -0.851456 -2.616130 2.083622

Cl -3.977567 -2.007743 0.899690

C 2.975805 -2.340392 2.644139

C 1.892666 -0.191753 3.304360

C 2.230368 -1.232885 2.257435

Pd -1.831592 -1.057152 0.610198

C -5.982867 3.013193 -0.326116

H -4.881917 -2.112708 -5.456078

C -4.515317 -1.624042 -4.563677

C -3.198421 -1.190733 -4.501565

H 2.886870 5.084336 3.415443

C 2.695717 4.292440 2.704435

C 1.468424 3.640620 2.695737

H 0.695236 3.924984 3.396021

C 1.226481 2.629083 1.785805

H 0.256450 2.151036 1.780162

C 2.211426 2.225890 0.886863

H 4.617415 4.438279 1.768319

C 3.421874 2.904480 0.877228

H 4.189266 2.645804 0.164919

C 3.663845 3.928639 1.785972

H 6.277053 -0.284268 -2.933687

C 5.384357 -0.009272 -2.388912

C 4.286141 0.500588 -3.059654

H 4.311222 0.622259 -4.133751

C 3.140149 0.852064 -2.357828

H 2.292264 1.236958 -2.903453

C 3.082411 0.714997 -0.977022

H 6.173490 -0.582371 -0.475656

C 4.189462 0.188276 -0.312244

H 4.170953 0.073596 0.761144

C 5.326982 -0.175472 -1.011102

H 3.818747 -4.226262 2.088223

C 3.248729 -3.367089 1.761203

C 2.770841 -3.305471 0.465599

H 2.963216 -4.124627 -0.212419

C 2.045568 -2.209108 0.018840

C 1.535933 -2.213643 -1.412258

C 0.334663 -3.151423 -1.559795

C 2.629197 -2.604088 -2.408233

H -0.028011 -3.137150 -2.588674

H 0.621411 -4.174755 -1.314049

H -0.481340 -2.870411 -0.898121

H 3.538583 -2.029768 -2.250734

H 2.875037 -3.663023 -2.328979

H 2.282107 -2.425456 -3.426407

H 1.189947 0.506726 2.863611

C 1.200761 -0.813218 4.520555

C 3.136554 0.580710 3.746900

H 3.633606 1.069287 2.911174

H 2.864547 1.348458 4.471429

H 3.852067 -0.091574 4.222914

H 0.326436 -1.385556 4.221290

H 1.876241 -1.472197 5.066834

H 0.888754 -0.022604 5.204475

H -6.848046 3.765249 1.485358

C -6.110694 3.101971 1.053982

C -5.301162 2.335426 1.879666

H -5.406972 2.396138 2.953827

C -4.360746 1.478229 1.328714

H -3.742170 0.862668 1.966703

1. **X-Ray single crystal structure analysis^[13]^**

| Manuscript number | **1d** |
| --- | --- |
| Empirical formula | C_23_H_29_Br_2_N |
| Formula weight | 479.29 |
| Temperature | 200(2) K |
| Wavelength | 0.71073 Å |
| Crystal system | orthorhombic |
| Space group | Pbca |
| Z | 8 |
| Unit cell dimensions | a = 14.6930(5) Å α = 90 deg. |
|  | b = 11.1444(4) Å β = 90 deg. |
|  | c = 27.5002(10) Å γ = 90 deg. |
| Volume | 4503.0(3) Å^3^ |
| Density (calculated) | 1.414 g/cm^3^ |
| Absorption coefficient | 3.607 mm^-1^ |
| Crystal shape | plate |
| Crystal size | 0.101 x 0.052 x 0.023 mm^3^ |
| Crystal colour | yellow |
| Theta range for data collection | 1.481 to 22.991 deg |
| Index ranges | -16≤h≤16, -12≤k≤12, -30≤l≤19 |
| Reflections collected | 22835 |
| Independent reflections | 3135 (R(int) = 0.1119) |
| Observed reflections | 1909 (I > 2σ(I)) |
| Absorption correction | Semi-empirical from equivalents |
| Max. and min. transmission | 0.93 and 0.80 |
| Refinement method | Full-matrix least-squares on F^2^ |
| Data/restraints/parameters | 3135 / 216 / 239 |
| Goodness-of-fit on F^2^ | 1.00 |
| Final R indices (I>2sigma(I)) | R1 = 0.045, wR2 = 0.064 |
| Largest diff. peak and hole | 0.46 and -0.41 eÅ^-3^ |

| Manuscript number | **1h** |
| --- | --- |
| Empirical formula | C_17_H_19_Br_2_N |
| Formula weight | 397.15 |
| Temperature | 200(2) K |
| Wavelength | 0.71073 Å |
| Crystal system | monoclinic |
| Space group | P2_1_/c |
| Z | 8 |
| Unit cell dimensions | a = 7.0809(4) Å α = 90 deg. |
|  | b = 27.6587(15) Å β = 90.0666(11) deg. |
|  | c = 16.1716(9) Å γ = 90 deg. |
| Volume | 3167.2(3) Å^3^ |
| Density (calculated) | 1.67 g/cm^3^ |
| Absorption coefficient | 5.11 mm^-1^ |
| Crystal shape | brick |
| Crystal size | 0.324 x 0.194 x 0.164 mm^3^ |
| Crystal colour | colorless |
| Theta range for data collection | 1.5 to 27.5 deg. |
| Index ranges | -9≤h≤9, -35≤k≤35, -20≤l≤20 |
| Reflections collected | 35146 |
| Independent reflections | 7247 (R(int) = 0.0556) |
| Observed reflections | 5061 (I > 2σ(I)) |
| Absorption correction | Semi-empirical from equivalents |
| Max. and min. transmission | 0.52 and 0.34 |
| Refinement method | Full-matrix least-squares on F^2^ |
| Data/restraints/parameters | 7247 / 0 / 361 |
| Goodness-of-fit on F^2^ | 1.02 |
| Final R indices (I>2sigma(I)) | R1 = 0.038, wR2 = 0.066 |
| Largest diff. peak and hole | 0.53 and -0.65 eÅ^-3^ |

| Manuscript number | **2a** |
| --- | --- |
| Empirical formula | C_33_H_31_BrF_3_NO_3_S |
| Formula weight | 658.56 |
| Temperature | 200(2) K |
| Wavelength | 0.71073 Å |
| Crystal system | triclinic |
| Space group | P$\bar{1}$ |
| Z | 2 |
| Unit cell dimensions | a = 10.0516(4) Å α = 72.3558(12) deg. |
|  | b = 10.7022(5) Å β = 72.2432(12) deg. |
|  | c = 14.9828(6) Å γ = 83.6409(13) deg. |
| Volume | 1462.47(11) Å^3^ |
| Density (calculated) | 1.50 g/cm^3^ |
| Absorption coefficient | 1.53 mm^-1^ |
| Crystal shape | brick |
| Crystal size | 0.102 x 0.058 x 0.022 mm^3^ |
| Crystal colour | brown |
| Theta range for data collection | 1.5 to 29.6 deg. |
| Index ranges | -13≤h≤13, -14≤k≤14, -20≤l≤20 |
| Reflections collected | 30376 |
| Independent reflections | 8069 (R(int) = 0.0588) |
| Observed reflections | 5361 (I > 2σ(I)) |
| Absorption correction | Semi-empirical from equivalents |
| Max. and min. transmission | 0.97 and 0.91 |
| Refinement method | Full-matrix least-squares on F^2^ |
| Data/restraints/parameters | 8069 / 0 / 383 |
| Goodness-of-fit on F^2^ | 1.02 |
| Final R indices (I>2sigma(I)) | R1 = 0.046, wR2 = 0.088 |
| Largest diff. peak and hole | 0.41 and -0.43 eÅ^-3^ |

| Manuscript number | **2b** |
| --- | --- |
| Empirical formula | C_31_H_27_BrF_3_NO_3_S |
| Formula weight | 630.50 |
| Temperature | 200(2) K |
| Wavelength | 0.71073 Å |
| Crystal system | orthorhombic |
| Space group | Pca2_1_ |
| Z | 4 |
| Unit cell dimensions | a = 17.9072(9) Å α = 90 deg. |
|  | b = 8.9033(4) Å β = 90 deg. |
|  | c = 17.8549(8) Å γ = 90 deg. |
| Volume | 2846.7(2) Å^3^ |
| Density (calculated) | 1.47 g/cm^3^ |
| Absorption coefficient | 1.57 mm^-1^ |
| Crystal shape | plank |
| Crystal size | 0.145 x 0.065 x 0.042 mm^3^ |
| Crystal colour | colorless |
| Theta range for data collection | 2.3 to 26.0 deg. |
| Index ranges | -22≤h≤19, -10≤k≤10, -20≤l≤20 |
| Reflections collected | 18204 |
| Independent reflections | 5012 (R(int) = 0.0609) |
| Observed reflections | 3818 (I > 2σ(I)) |
| Absorption correction | Semi-empirical from equivalents |
| Max. and min. transmission | 0.94 and 0.84 |
| Refinement method | Full-matrix least-squares on F^2^ |
| Data/restraints/parameters | 5012 / 1 / 363 |
| Goodness-of-fit on F^2^ | 1.02 |
| Final R indices (I>2sigma(I)) | R1 = 0.039, wR2 = 0.068 |
| Largest diff. peak and hole | 0.30 and -0.30 eÅ^-3^ |

| Manuscript number | **2d** |
| --- | --- |
| Empirical formula | C_37_H_39_BrF_3_NO_3_S |
| Formula weight | 714.66 |
| Temperature | 200(2) K |
| Wavelength | 0.71073 Å |
| Crystal system | orthorhombic |
| Space group | Pca2_1_ |
| Z | 8 |
| Unit cell dimensions | a = 13.8505(8) Å α = 90 deg. |
|  | b = 23.3657(13) Å β = 90 deg. |
|  | c = 21.1196(12) Å γ = 90 deg. |
| Volume | 6834.9(7) Å^3^ |
| Density (calculated) | 1.39 g/cm^3^ |
| Absorption coefficient | 1.32 mm^-1^ |
| Crystal shape | brick |
| Crystal size | 0.060 x 0.050 x 0.050 mm^3^ |
| Crystal colour | yellow |
| Theta range for data collection | 0.9 to 23.3 deg. |
| Index ranges | -15≤h≤15, -25≤k≤25, -23≤l≤23 |
| Reflections collected | 37219 |
| Independent reflections | 9815 (R(int) = 0.0791) |
| Observed reflections | 7186 (I > 2σ(I)) |
| Absorption correction | Semi-empirical from equivalents |
| Max. and min. transmission | 0.94 and 0.84 |
| Refinement method | Full-matrix least-squares on F^2^ |
| Data/restraints/parameters | 9815 / 1121 / 885 |
| Goodness-of-fit on F^2^ | 1.04 |
| Final R indices (I>2sigma(I)) | R1 = 0.063, wR2 = 0.150 |
| Largest diff. peak and hole | 0.97 and -0.65 eÅ^-3^ |

| Manuscript number | **2i** |
| --- | --- |
| Empirical formula | C_25_H_25_BrF_3_NO_4_S |
| Formula weight | 572.43 |
| Temperature | 200(2) K |
| Wavelength | 0.71073 Å |
| Crystal system | triclinic |
| Space group | P |
| Z | 2 |
| Unit cell dimensions | a = 8.7689(6) Å α = 77.2893(15) deg. |
|  | b = 10.0916(7) Å β = 82.8901(16) deg. |
|  | c = 15.1785(10) Å γ = 78.6529(16) deg. |
| Volume | 1280.10(15) Å^3^ |
| Density (calculated) | 1.49 g/cm^3^ |
| Absorption coefficient | 1.74 mm^-1^ |
| Crystal shape | column |
| Crystal size | 0.190 x 0.072 x 0.072 mm^3^ |
| Crystal colour | colorless |
| Theta range for data collection | 1.4 to 27.7 deg. |
| Index ranges | -11≤h≤11, -13≤k≤13, -19≤l≤19 |
| Reflections collected | 17727 |
| Independent reflections | 5292 (R(int) = 0.0326) |
| Observed reflections | 4182 (I > 2σ(I)) |
| Absorption correction | Semi-empirical from equivalents |
| Max. and min. transmission | 0.90 and 0.83 |
| Refinement method | Full-matrix least-squares on F^2^ |
| Data/restraints/parameters | 5292 / 0 / 325 |
| Goodness-of-fit on F^2^ | 1.10 |
| Final R indices (I>2sigma(I)) | R1 = 0.035, wR2 = 0.088 |
| Largest diff. peak and hole | 0.45 and -0.46 eÅ^-3^ |

| Manuscript number | **2j** |
| --- | --- |
| Empirical formula | C_24_H_21_BrF_3_NO_3_S |
| Formula weight | 540.39 |
| Temperature | 200(2) K |
| Wavelength | 0.71073 Å |
| Crystal system | monoclinic |
| Space group | P2_1_/n |
| Z | 4 |
| Unit cell dimensions | a = 10.1729(9) Å α = 90 deg. |
|  | b = 15.1907(14) Å β = 94.789(2) deg. |
|  | c = 14.9173(14) Å γ = 90 deg. |
| Volume | 2297.2(4) Å^3^ |
| Density (calculated) | 1.56 g/cm^3^ |
| Absorption coefficient | 1.93 mm^-1^ |
| Crystal shape | brick |
| Crystal size | 0.073 x 0.067 x 0.040 mm^3^ |
| Crystal colour | colorless |
| Theta range for data collection | 1.9 to 26.4 deg. |
| Index ranges | -12≤h≤12, -18≤k≤18, -18≤l≤18 |
| Reflections collected | 23669 |
| Independent reflections | 4682 (R(int) = 0.1091) |
| Observed reflections | 2702 (I > 2σ(I)) |
| Absorption correction | Semi-empirical from equivalents |
| Max. and min. transmission | 0.94 and 0.87 |
| Refinement method | Full-matrix least-squares on F^2^ |
| Data/restraints/parameters | 4682 / 0 / 300 |
| Goodness-of-fit on F^2^ | 1.01 |
| Final R indices (I>2sigma(I)) | R1 = 0.048, wR2 = 0.076 |
| Largest diff. peak and hole | 0.40 and -0.38 eÅ^-3^ |

| Manuscript number | **3a** |
| --- | --- |
| Empirical formula | C_33_H_34_BrNO |
| Formula weight | 540.52 |
| Temperature | 200(2) K |
| Wavelength | 1.54178 Å |
| Crystal system | orthorhombic |
| Space group | Pbca |
| Z | 8 |
| Unit cell dimensions | a = 17.8816(3) Å α = 90 deg. |
|  | b = 16.7750(3) Å β = 90 deg. |
|  | c = 18.1535(3) Å γ = 90 deg. |
| Volume | 5445.39(16) Å^3^ |
| Density (calculated) | 1.32 g/cm^3^ |
| Absorption coefficient | 2.24 mm^-1^ |
| Crystal shape | brick |
| Crystal size | 0.140 x 0.120 x 0.108 mm^3^ |
| Crystal colour | pale brown |
| Theta range for data collection | 4.4 to 68.3 deg. |
| Index ranges | -21≤h≤17, -20≤k≤17, -21≤l≤14 |
| Reflections collected | 30179 |
| Independent reflections | 4886 (R(int) = 0.0212) |
| Observed reflections | 4137 (I > 2σ(I)) |
| Absorption correction | Semi-empirical from equivalents |
| Max. and min. transmission | 0.78 and 0.50 |
| Refinement method | Full-matrix least-squares on F^2^ |
| Data/restraints/parameters | 4886 / 0 / 330 |
| Goodness-of-fit on F^2^ | 1.02 |
| Final R indices (I>2sigma(I)) | R1 = 0.035, wR2 = 0.093 |
| Largest diff. peak and hole | 0.39 and -0.56 eÅ^-3^ |

| Manuscript number | **3b** |
| --- | --- |
| Empirical formula | C_31_H_30_BrNO |
| Formula weight | 512.47 |
| Temperature | 200(2) K |
| Wavelength | 0.71073 Å |
| Crystal system | monoclinic |
| Space group | P2_1_/c |
| Z | 4 |
| Unit cell dimensions | a = 17.239(3) Å α = 90 deg. |
|  | b = 17.238(3) Å β = 101.528(4) deg. |
|  | c = 8.5854(14) Å γ = 90 deg. |
| Volume | 2499.7(7) Å^3^ |
| Density (calculated) | 1.36 g/cm^3^ |
| Absorption coefficient | 1.67 mm^-1^ |
| Crystal shape | brick |
| Crystal size | 0.139 x 0.050 x 0.042 mm^3^ |
| Crystal colour | colorless |
| Theta range for data collection | 1.7 to 26.0 deg. |
| Index ranges | -21≤h≤21, -21≤k≤21, -10≤l≤10 |
| Reflections collected | 25253 |
| Independent reflections | 4926 (R(int) = 0.1036) |
| Observed reflections | 2941 (I > 2σ(I)) |
| Absorption correction | Semi-empirical from equivalents |
| Max. and min. transmission | 0.94 and 0.88 |
| Refinement method | Full-matrix least-squares on F^2^ |
| Data/restraints/parameters | 4926 / 0 / 310 |
| Goodness-of-fit on F^2^ | 1.02 |
| Final R indices (I>2sigma(I)) | R1 = 0.051, wR2 = 0.100 |
| Largest diff. peak and hole | 0.44 and -0.43 eÅ^-3^ |

| Manuscript number | **4a** |
| --- | --- |
| Empirical formula | C_45_H_44_NOP |
| Formula weight | 645.78 |
| Temperature | 200(2) K |
| Wavelength | 0.71073 Å |
| Crystal system | triclinic |
| Space group | P-1 |
| Z | 2 |
| Unit cell dimensions | a = 12.3485(7) Å α = 85.039(2) deg. |
|  | b = 12.7230(8) Å β = 70.800(2) deg. |
|  | c = 13.3256(8) Å γ = 66.281(1) deg. |
| Volume | 1807.58(19) Å^3^ |
| Density (calculated) | 1.19 g/cm^3^ |
| Absorption coefficient | 0.11 mm^-1^ |
| Crystal shape | column |
| Crystal size | 0.142 x 0.068 x 0.050 mm^3^ |
| Crystal colour | colorless |
| Theta range for data collection | 1.6 to 20.4 deg. |
| Index ranges | -12≤h≤12, -12≤k≤12, -13≤l≤13 |
| Reflections collected | 30262 |
| Independent reflections | 3561 (R(int) = 0.0478) |
| Observed reflections | 2822 (I > 2\s(I)) |
| Absorption correction | Semi-empirical from equivalents |
| Max. and min. transmission | 0.75 and 0.72 |
| Refinement method | Full-matrix least-squares on F^2^ |
| Data/restraints/parameters | 3561 / 0 / 438 |
| Goodness-of-fit on F^2^ | 1.07 |
| Final R indices (I>2sigma(I)) | R1 = 0.040, wR2 = 0.094 |
| Largest diff. peak and hole | 0.25 and -0.24 eÅ^-3^ |

| Manuscript number | **4b** |
| --- | --- |
| Empirical formula | C_43_H_40_NOP |
| Formula weight | 617.73 |
| Temperature | 200(2) K |
| Wavelength | 0.71073 Å |
| Crystal system | triclinic |
| Space group | P$\bar{1}$ |
| Z | 2 |
| Unit cell dimensions | a = 9.7108(7) Å α = 102.7458(19) deg. |
|  | b = 12.3485(9) Å β = 100.3364(19) deg. |
|  | c = 15.4959(12) Å γ = 104.7717(17) deg deg. |
| Volume | 1696.9(2) Å^3^ |
| Density (calculated) | 1.21 g/cm^3^ |
| Absorption coefficient | 0.12 mm^-1^ |
| Crystal shape | plank |
| Crystal size | 0.387 x 0.150 x 0.012 mm^3^ |
| Crystal colour | colorless |
| Theta range for data collection | 1.8 to 28.4 deg. |
| Index ranges | -12≤h≤12, -16≤k≤15, -20≤l≤20 |
| Reflections collected | 30994 |
| Independent reflections | 7739 (R(int) = 0.0621) |
| Observed reflections | 4961 (I > 2σ(I)) |
| Absorption correction | Semi-empirical from equivalents |
| Max. and min. transmission | 0.96 and 0.80 |
| Refinement method | Full-matrix least-squares on F^2^ |
| Data/restraints/parameters | 7739 / 0 / 418 |
| Goodness-of-fit on F^2^ | 1.02 |
| Final R indices (I>2sigma(I)) | R1 = 0.052, wR2 = 0.110 |
| Largest diff. peak and hole | 0.36 and -0.28 eÅ^-3^ |

| Manuscript number | **4c** |
| --- | --- |
| Empirical formula | C_44_H_42_NOP |
| Formula weight | 631.75 |
| Temperature | 200(2) K |
| Wavelength | 1.54178 Å |
| Crystal system | triclinic |
| Space group | P$\bar{1}$ |
| Z | 8 |
| Unit cell dimensions | a = 16.1894(9) Å α = 104.666(4) deg. |
|  | b = 22.4890(12) Å β = 105.866(4) deg. |
|  | c = 22.6595(13) Å γ = 106.755(4) deg. deg. |
| Volume | 7088.0(7) Å^3^ |
| Density (calculated) | 1.18 g/cm^3^ |
| Absorption coefficient | 0.94 mm^-1^ |
| Crystal shape | plank |
| Crystal size | 0.112 x 0.028 x 0.016 mm^3^ |
| Crystal colour | colorless |
| Theta range for data collection | 3.0 to 62.3 deg. |
| Index ranges | -18≤h≤17, -12≤k≤24, -25≤l≤24 |
| Reflections collected | 73746 |
| Independent reflections | 21272 (R(int) = 0.2342) |
| Observed reflections | 9753 (I > 2σ(I)) |
| Absorption correction | Semi-empirical from equivalents |
| Max. and min. transmission | 0.99 and 0.84 |
| Refinement method | Full-matrix least-squares on F^2^ |
| Data/restraints/parameters | 21272 / 3796 / 1718 |
| Goodness-of-fit on F^2^ | 1.13 |
| Final R indices (I>2sigma(I)) | R1 = 0.147, wR2 = 0.252 |
| Largest diff. peak and hole | 0.39 and -0.42 eÅ^-3^ |

| Manuscript number | **4d** |
| --- | --- |
| Empirical formula | C_49_H_52_NOP |
| Formula weight | 701.88 |
| Temperature | 200(2) K |
| Wavelength | 0.71073 Å |
| Crystal system | triclinic |
| Space group | P$\bar{1}$ |
| Z | 2 |
| Unit cell dimensions | a = 9.6538(17) Å α = 95.279(4) deg. |
|  | b = 10.478(2) Å β = 102.710(4) deg. |
|  | c = 20.895(4) Å γ = 103.583(4) deg. deg. |
| Volume | 1980.9(6) Å^3^ |
| Density (calculated) | 1.18 g/cm^3^ |
| Absorption coefficient | 0.11 mm^-1^ |
| Crystal shape | brick |
| Crystal size | 0.055 x 0.032 x 0.028 mm^3^ |
| Crystal colour | colorless |
| Theta range for data collection | 1.0 to 21.1 deg. |
| Index ranges | -9≤h≤9, -10≤k≤10, -21≤l≤21 |
| Reflections collected | 19185 |
| Independent reflections | 4321 (R(int) = 0.1183) |
| Observed reflections | 2418 (I > 2σ(I)) |
| Absorption correction | Semi-empirical from equivalents |
| Max. and min. transmission | 0.96 and 0.89 |
| Refinement method | Full-matrix least-squares on F^2^ |
| Data/restraints/parameters | 4321 / 411 / 474 |
| Goodness-of-fit on F^2^ | 1.02 |
| Final R indices (I>2sigma(I)) | R1 = 0.058, wR2 = 0.101 |
| Largest diff. peak and hole | 0.22 and -0.22 eÅ^-3^ |

| Manuscript number | **4e** |
| --- | --- |
| Empirical formula | C_53_H_60_NOP |
| Formula weight | 757.99 |
| Temperature | 200(2) K |
| Wavelength | 0.71073 Å |
| Crystal system | triclinic |
| Space group | P$\bar{1}$ |
| Z | 2 |
| Unit cell dimensions | a = 9.638(2) Å α = 84.878(5) deg. |
|  | b = 11.165(2) Å β = 88.640(5) deg. |
|  | c = 21.770(4) Å γ = 72.522(5) deg. |
| Volume | 2225.6(8) Å^3^ |
| Density (calculated) | 1.13 g/cm^3^ |
| Absorption coefficient | 0.10 mm^-1^ |
| Crystal shape | plate |
| Crystal size | 0.071 x 0.067 x 0.017 mm^3^ |
| Crystal colour | colorless |
| Theta range for data collection | 0.9 to 21.3 deg. |
| Index ranges | -9≤h≤9, -11≤k≤11, -22≤l≤22 |
| Reflections collected | 21048 |
| Independent reflections | 4980 (R(int) = 0.1143) |
| Observed reflections | 3024 (I > 2σ(I)) |
| Absorption correction | Semi-empirical from equivalents |
| Max. and min. transmission | 0.96 and 0.84 |
| Refinement method | Full-matrix least-squares on F^2^ |
| Data/restraints/parameters | 4980 / 435 / 510 |
| Goodness-of-fit on F^2^ | 1.14 |
| Final R indices (I>2sigma(I)) | R1 = 0.111, wR2 = 0.286 |
| Largest diff. peak and hole | 0.44 and -0.35 eÅ^-3^ |

| Manuscript number | **4e** |
| --- | --- |
| Empirical formula | C_53_H_60_NOP |
| Formula weight | 757.99 |
| Temperature | 200(2) K |
| Wavelength | 0.71073 Å |
| Crystal system | triclinic |
| Space group | P$\bar{1}$ |
| Z | 2 |
| Unit cell dimensions | a = 9.638(2) Å α = 84.878(5) deg. |
|  | b = 11.165(2) Å β = 88.640(5) deg. |
|  | c = 21.770(4) Å γ = 72.522(5) deg. |
| Volume | 2225.6(8) Å^3^ |
| Density (calculated) | 1.13 g/cm^3^ |
| Absorption coefficient | 0.10 mm^-1^ |
| Crystal shape | plate |
| Crystal size | 0.071 x 0.067 x 0.017 mm^3^ |
| Crystal colour | colorless |
| Theta range for data collection | 0.9 to 21.3 deg. |
| Index ranges | -9≤h≤9, -11≤k≤11, -22≤l≤22 |
| Reflections collected | 21048 |
| Independent reflections | 4980 (R(int) = 0.1143) |
| Observed reflections | 3024 (I > 2σ(I)) |
| Absorption correction | Semi-empirical from equivalents |
| Max. and min. transmission | 0.96 and 0.84 |
| Refinement method | Full-matrix least-squares on F^2^ |
| Data/restraints/parameters | 4980 / 435 / 510 |
| Goodness-of-fit on F^2^ | 1.14 |
| Final R indices (I>2sigma(I)) | R1 = 0.111, wR2 = 0.286 |
| Largest diff. peak and hole | 0.44 and -0.35 eÅ^-3^ |

| Manuscript number | **4f** |
| --- | --- |
| Empirical formula | C_51_H_58_NO_4_P |
| Formula weight | 779.95 |
| Temperature | 200(2) K |
| Wavelength | 0.71073 Å |
| Crystal system | orthorhombic |
| Space group | P2_1_2_1_2_1_ |
| Z | 4 |
| Unit cell dimensions | a = 9.7464(6) Å α = 90 deg. |
|  | b = 15.4377(10) Å β = 90 deg. |
|  | c = 29.552(2) Å γ = 90 deg. |
| Volume | 4446.5(5) Å^3^ |
| Density (calculated) | 1.16 g/cm^3^ |
| Absorption coefficient | 0.11 mm^-1^ |
| Crystal shape | plank |
| Crystal size | 0.130 x 0.040 x 0.023 mm^3^ |
| Crystal colour | colorless |
| Theta range for data collection | 1.4 to 23.0 deg. |
| Index ranges | -10≤h≤10, -16≤k≤16, -32≤l≤32 |
| Reflections collected | 36795 |
| Independent reflections | 6197 (R(int) = 0.0870) |
| Observed reflections | 4544 (I > 2σ(I)) |
| Absorption correction | Semi-empirical from equivalents |
| Max. and min. transmission | 0.96 and 0.92 |
| Refinement method | Full-matrix least-squares on F^2^ |
| Data/restraints/parameters | 6197 / 0 / 521 |
| Goodness-of-fit on F^2^ | 0.98 |
| Final R indices (I>2sigma(I)) | R1 = 0.044, wR2 = 0.081 |
| Largest diff. peak and hole | 0.20 and -0.19 eÅ^-3^ |

| Manuscript number | **5a** |
| --- | --- |
| Empirical formula | C_44_H_41_BF_4_NP |
| Formula weight | 701.56 |
| Temperature | 200(2) K |
| Wavelength | 0.71073 Å |
| Crystal system | triclinic |
| Space group | P |
| Z | 2 |
| Unit cell dimensions | a = 9.6906(7) Å α = 78.0266(17) deg. |
|  | b = 10.1659(7) Å β = 82.7420(16) deg. |
|  | c = 19.4169(14) Å γ = 84.3094(16) deg. |
| Volume | 1851.0(2) Å^3^ |
| Density (calculated) | 1.26 g/cm^3^ |
| Absorption coefficient | 0.13 mm^-1^ |
| Crystal shape | prism |
| Crystal size | 0.103 x 0.047 x 0.030 mm^3^ |
| Crystal colour | orange |
| Theta range for data collection | 2.1 to 28.4 deg. |
| Index ranges | -12≤h≤12, -13≤k≤13, -25≤l≤25 |
| Reflections collected | 33766 |
| Independent reflections | 8472 (R(int) = 0.0557) |
| Observed reflections | 5436 (I > 2σ(I)) |
| Absorption correction | Semi-empirical from equivalents |
| Max. and min. transmission | 0.96 and 0.93 |
| Refinement method | Full-matrix least-squares on F^2^ |
| Data/restraints/parameters | 8472 / 94 / 510 |
| Goodness-of-fit on F^2^ | 1.02 |
| Final R indices (I>2sigma(I)) | R1 = 0.052, wR2 = 0.100 |
| Largest diff. peak and hole | 0.24 and -0.28 eÅ^-3^ |

| Manuscript number | **6** |
| --- | --- |
| Empirical formula | C_44_H_40_NP |
| Formula weight | 613.74 |
| Temperature | 200(2) K |
| Wavelength | 0.71073 Å |
| Crystal system | monoclinic |
| Space group | P2_1_/n |
| Z | 4 |
| Unit cell dimensions | a = 13.3854(12) Å α = 90 deg. |
|  | b = 13.4738(12) Å β = 92.038(3) deg. |
|  | c = 19.6026(18) Å γ = 90 deg. |
| Volume | 3533.1(6) Å^3^ |
| Density (calculated) | 1.15 g/cm^3^ |
| Absorption coefficient | 0.11 mm^-1^ |
| Crystal shape | irregular |
| Crystal size | 0.133 x 0.058 x 0.040 mm^3^ |
| Crystal colour | yellow |
| Theta range for data collection | 1.8 to 23.6 deg. |
| Index ranges | -15≤h≤15, -15≤k≤15, -22≤l≤21 |
| Reflections collected | 29454 |
| Independent reflections | 5251 (R(int) = 0.0994) |
| Observed reflections | 3061 (I > 2σ(I)) |
| Absorption correction | Semi-empirical from equivalents |
| Max. and min. transmission | 0.96 and 0.86 |
| Refinement method | Full-matrix least-squares on F^2^ |
| Data/restraints/parameters | 5251 / 422 / 440 |
| Goodness-of-fit on F^2^ | 1.01 |
| Final R indices (I>2sigma(I)) | R1 = 0.061, wR2 = 0.125 |
| Largest diff. peak and hole | 0.34 and -0.33 eÅ^-3^ |

| Manuscript number | **7a** |
| --- | --- |
| Empirical formula | C_44_H_40_Cl_2_NPPd ∙ 2 CHCl_3_ |
| Formula weight | 1029.77 |
| Temperature | 200(2) K |
| Wavelength | 0.71073 Å |
| Crystal system | triclinic |
| Space group | P$\bar{1}$ |
| Z | 2 |
| Unit cell dimensions | a = 9.7865(7) Å α = 87.7862(17) deg. |
|  | b = 11.3274(8) Å β = 87.9332(17) deg. |
|  | c = 22.2043(15) Å γ = 70.3104(17) deg. |
| Volume | 2315.1(3) Å^3^ |
| Density (calculated) | 1.48 g/cm^3^ |
| Absorption coefficient | 0.93 mm^-1^ |
| Crystal shape | plank |
| Crystal size | 0.156 x 0.056 x 0.032 mm^3^ |
| Crystal colour | colorless |
| Theta range for data collection | 0.9 to 27.7 deg. |
| Index ranges | -12≤h≤12, -14≤k≤14, -29≤l≤28 |
| Reflections collected | 32901 |
| Independent reflections | 9842 (R(int) = 0.0777) |
| Observed reflections | 6472 (I > 2σ(I)) |
| Absorption correction | Semi-empirical from equivalents |
| Max. and min. transmission | 0.96 and 0.86 |
| Refinement method | Full-matrix least-squares on F^2^ |
| Data/restraints/parameters | 9842 / 72 / 535 |
| Goodness-of-fit on F^2^ | 1.02 |
| Final R indices (I>2sigma(I)) | R1 = 0.056, wR2 = 0.113 |
| Largest diff. peak and hole | 0.90 and -0.68 eÅ^-3^ |

| Manuscript number | **7g** |
| --- | --- |
| Empirical formula | C_44_H_40_Cl_2_NPPd ∙ 2 CHCl_3_ |
| Formula weight | 1029.77 |
| Temperature | 200(2) K |
| Wavelength | 0.71073 Å |
| Crystal system | triclinic |
| Space group | P$\bar{1}$ |
| Z | 2 |
| Unit cell dimensions | a = 9.7865(7) Å α = 87.7862(17) deg. |
|  | b = 11.3274(8) Å β = 87.9332(17) deg. |
|  | c = 22.2043(15) Å γ = 70.3104(17) deg. |
| Volume | 2315.1(3) Å^3^ |
| Density (calculated) | 1.48 g/cm^3^ |
| Absorption coefficient | 0.93 mm^-1^ |
| Crystal shape | plank |
| Crystal size | 0.156 x 0.056 x 0.032 mm^3^ |
| Crystal colour | colorless |
| Theta range for data collection | 0.9 to 27.7 deg. |
| Index ranges | -12≤h≤12, -14≤k≤14, -29≤l≤28 |
| Reflections collected | 32901 |
| Independent reflections | 9842 (R(int) = 0.0777) |
| Observed reflections | 6472 (I > 2σ(I)) |
| Absorption correction | Semi-empirical from equivalents |
| Max. and min. transmission | 0.96 and 0.86 |
| Refinement method | Full-matrix least-squares on F^2^ |
| Data/restraints/parameters | 9842 / 72 / 535 |
| Goodness-of-fit on F^2^ | 1.02 |
| Final R indices (I>2sigma(I)) | R1 = 0.056, wR2 = 0.113 |
| Largest diff. peak and hole | 0.90 and -0.68 eÅ^-3^ |

| Manuscript number | **7g** |
| --- | --- |
| Empirical formula | C_48_H_48_Cl_2_NPPd |
| Formula weight | 847.21 |
| Temperature | 200(2) K |
| Wavelength | 0.71073 Å |
| Crystal system | monoclinic |
| Space group | P2_1_ |
| Z | 2 |
| Unit cell dimensions | a = 10.6007(6) Å α = 90 deg. |
|  | b = 13.2674(7) Å β = 92.5575(12) deg. |
|  | c = 17.7538(10) Å γ = 90 deg. |
| Volume | 2494.5(2) Å^3^ |
| Density (calculated) | 1.15 g/cm^3^ |
| Absorption coefficient | 0.54 mm^-1^ |
| Crystal shape | plank |
| Crystal size | 0.164 x 0.128 x 0.060 mm^3^ |
| Crystal colour | yellow |
| Theta range for data collection | 1.9 to 26.1 deg. |
| Index ranges | -13≤h≤13, -16≤k≤16, -21≤l≤21 |
| Reflections collected | 25134 |
| Independent reflections | 9787 (R(int) = 0.0484) |
| Observed reflections | 8504 (I > 2σ(I)) |
| Absorption correction | Semi-empirical from equivalents |
| Max. and min. transmission | 0.96 and 0.89 |
| Refinement method | Full-matrix least-squares on F^2^ |
| Data/restraints/parameters | 9787 / 462 / 513 |
| Goodness-of-fit on F^2^ | 1.02 |
| Final R indices (I>2sigma(I)) | R1 = 0.044, wR2 = 0.095 |
| Largest diff. peak and hole | 0.81 and -0.43 eÅ^-3^ |

| Manuscript number | **8a** |
| --- | --- |
| Empirical formula | C_44_H_40_Cl_2_NPPt |
| Formula weight | 879.73 |
| Temperature | 200(2) K |
| Wavelength | 0.71073 Å |
| Crystal system | monoclinic |
| Space group | P2_1_/n |
| Z | 4 |
| Unit cell dimensions | a = 16.3602(8) Å α = 90 deg. deg. |
|  | b = 12.8653(7) Å β = 113.2757(11) deg. |
|  | c = 19.2985(10) Å γ = 90 deg. |
| Volume | 3731.3(3) Å^3^ |
| Density (calculated) | 1.57 g/cm^3^ |
| Absorption coefficient | 3.98 mm^-1^ |
| Crystal shape | cuboid |
| Crystal size | 0.124 x 0.105 x 0.075 mm^3^ |
| Crystal colour | yellow |
| Theta range for data collection | 2.0 to 26.1 deg. |
| Index ranges | -20≤h≤19, -15≤k≤15, -23≤l≤23 |
| Reflections collected | 36895 |
| Independent reflections | 7364 (R(int) = 0.0654) |
| Observed reflections | 5613 (I > 2σ(I)) |
| Absorption correction | Semi-empirical from equivalents |
| Max. and min. transmission | 0.79 and 0.72 |
| Refinement method | Full-matrix least-squares on F^2^ |
| Data/restraints/parameters | 7364 / 0 / 446 |
| Goodness-of-fit on F^2^ | 1.02 |
| Final R indices (I>2sigma(I)) | R1 = 0.034, wR2 = 0.064 |
| Largest diff. peak and hole | 1.46 and -1.33 eÅ^-3^ |

## **References**

[15] a) N. Slavov, J. Cvengroš, J.-M. Neudörfl, H.-G. Schmalz, *Angew. Chem. Int. Ed.* **2010**, *49*, 7588–7591.

b) N. Slavov, J. Cvengroš, J.-M. Neudörfl, H.-G. Schmalz, *Angew. Chem.* **2010**, *122*, 7751–7754.

[16] H. Nakagawa, T. Nagano, T. Higuchi, *Org. Lett.* **2001**, *3*, 12, 1805–1807.

[17] G. R. Fulmer, A. J. M. Miller, N. H. Sherden, H. E. Gottlieb, A. Nudelman, B. M. Stoltz, J. E. Bercaw, K. I. Goldberg, *Organometallics* **2010**, *29*, 2176.

[18] G. M. Sheldrick, *Acta Crystallogr.* *Sect. A* **2015**, *71*, 3-8.

[19] G. M. Sheldrick, *Acta Crystallogr.* *Sect. C* **2015**, *71*, 3-8.

[20] Bruker, APEX, APEX2, SMART, SAINT, SAINT-Plus Program for the Solution of Crystal Structures, Bruker AXS Inc., Madison, Wisconsin (USA), **2007**.

[21]  S. Morales, F. G. Guijarro, J. L. García Ruano, M. B. Cid, *J. Am. Chem.*

*Soc.* **2014**, *136*, 1082.

[22]  T. Tu, Z. Sun, W. Fang, M. Xu, Y. Zhou, *Org. Lett.* **2012**, *14*, 4250.

[23]  K. L. Billingsley, S. L. Buchwald, *J. Org. Chem.* **2008**, *73*, 5589.

[24] F. Neese, *Wiley Interdiscip. Rev. Comput. Mol. Sci.* **2022**, *12*, e1606.

[25] F. Weigend, *Phys. Chem. Chem. Phys.* **2006**, *8*, 1057–1065.

[26] B. Helmich-Paris, B. de Souza, F. Neese, R. Izsák, *J. Chem. Phys.* **2021**, *155*, 104109-1–104109-14.

[27] S. Grimme, A. Hansen, S. Ehlert, J.-M. Mewes, *J. Chem. Phys.* **2021**, *154*, 64103.

[28] V. Ásgeirsson, B. O. Birgisson, R. Bjornsson, U. Becker, F. Neese, C. Riplinger, H. Jónsson, *J. Chem. Theory Comput.* **2021**, *17*, 4929–4945.

[29] A. Najibi, L. Goerigk, *J. Comput. Chem.* **2020**, *41*, 2562–2572.

[30] F. Weigend, R. Ahlrichs, *Phys. Chem. Chem. Phys.* **2005**, *7*, 3297–3305.

[31] S. Grimme, *Chem. Eur. J* **2012**, *18*, 9955–9964.
